# Effect of Continuous Electrocardiogram Monitoring on Detection of Undiagnosed Atrial Fibrillation After Hospitalization for Cardiac Surgery: A Randomized Clinical Trial

**DOI:** 10.1001/jamanetworkopen.2021.21867

**Published:** 2021-08-27

**Authors:** Andrew C. T. Ha, Subodh Verma, C. David Mazer, Adrian Quan, Bobby Yanagawa, David A. Latter, Terrence M. Yau, Frédéric Jacques, Craig D. Brown, Rohit K. Singal, Michael H. Yamashita, Tarit Saha, Kevin H. Teoh, Buu-Khanh Lam, Marc W. Deyell, Marnee Wilson, Makoto Hibino, Christopher C. Cheung, Andrew Kosmopoulos, Vinay Garg, Shira Brodutch, Hwee Teoh, Fei Zuo, Kevin E. Thorpe, Peter Jüni, Deepak L. Bhatt, Atul Verma

**Affiliations:** 1 Peter Munk Cardiac Centre, Toronto General Hospital, University Health Network, University of Toronto, Toronto, Ontario, Canada; 2 Department of Cardiac Surgery, St Michael’s Hospital, University of Toronto, Toronto, Ontario, Canada; 3 Department of Anesthesiology, St Michael’s Hospital, University of Toronto, Toronto, Ontario, Canada; 4 University Institute of Cardiology and Respirology of Québec, Quebec City, Quebec, Canada; 5 Division of Cardiac Surgery, New Brunswick, Saint John, New Brunswick, Canada; 6 Division of Surgery, Cardiac Science Program, St Boniface General Hospital, Winnipeg, Manitoba, Canada; 7 Department of Anesthesiology and Perioperative Medicine, Kingston General Hospital, Kingston, Ontario, Canada; 8 Southlake Regional Health Center, University of Toronto, Newmarket, Ontario, Canada; 9 University of Ottawa Heart Institute, Ottawa, Ontario, Canada; 10 Division of Cardiology, St Paul’s Hospital, Vancouver, British Columbia, Canada; 11 Applied Health Research Centre, Li Ka Shing Knowledge Institute of St Michael’s Hospital, Toronto, Ontario, Canada; 12 Heart and Vascular Center, Brigham and Women’s Hospital, Harvard Medical School, Boston, Massachusetts

## Abstract

**Importance:**

Postoperative atrial fibrillation (POAF) occurring after cardiac surgery is associated with adverse outcomes. Whether POAF persists beyond discharge is not well defined.

**Objective:**

To determine whether continuous cardiac rhythm monitoring enhances detection of POAF among cardiac surgical patients during the first 30 days after hospital discharge compared with usual care.

**Design, Setting, and Participants:**

This study is an investigator-initiated, open-label, multicenter, randomized clinical trial conducted at 10 Canadian centers. Enrollment spanned from March 2017 to March 2020, with follow-up through September 11, 2020. As a result of the COVID-19 pandemic, enrollment stopped on July 17, 2020, at which point 85% of the proposed sample size was enrolled. Cardiac surgical patients with CHA_2_DS_2_-VASc (congestive heart failure, hypertension, age ≥75 years, diabetes, prior stroke or transient ischemic attack, vascular disease, age 65-74 years, female sex) score greater than or equal to 4 or greater than or equal to 2 with risk factors for POAF, no history of preoperative AF, and POAF lasting less than 24 hours during hospitalization were enrolled.

**Interventions:**

The intervention group underwent continuous cardiac rhythm monitoring with wearable, patch-based monitors for 30 days after randomization. Monitoring was not mandated in the usual care group within 30 days after randomization.

**Main Outcomes and Measures:**

The primary outcome was cumulative AF and/or atrial flutter lasting 6 minutes or longer detected by continuous cardiac rhythm monitoring or by a 12-lead electrocardiogram within 30 days of randomization. Prespecified secondary outcomes included cumulative AF lasting 6 hours or longer and 24 hours or longer within 30 days of randomization, death, myocardial infarction, ischemic stroke, non–central nervous system thromboembolism, major bleeding, and oral anticoagulation prescription.

**Results:**

Of the 336 patients randomized (163 patients in the intervention group and 173 patients in the usual care group; mean [SD] age, 67.4 [8.1] years; 73 women [21.7%]; median [interquartile range] CHA_2_DS_2_-VASc score, 4.0 [3.0-4.0] points), 307 (91.4%) completed the trial. In the intent-to-treat analysis, the primary end point occurred in 32 patients (19.6%) in the intervention group vs 3 patients (1.7%) in the usual care group (absolute difference, 17.9%; 95% CI, 11.5%-24.3%; *P* < .001). AF lasting 6 hours or longer was detected in 14 patients (8.6%) in the intervention group vs 0 patients in the usual care group (absolute difference, 8.6%; 95% CI, 4.3%-12.9%; *P* < .001).

**Conclusions and Relevance:**

In post–cardiac surgical patients at high risk of stroke, no preoperative AF history, and AF lasting less than 24 hours during hospitalization, continuous monitoring revealed a significant increase in the rate of POAF after discharge that would otherwise not be detected by usual care. Studies are needed to examine whether these patients will benefit from oral anticoagulation therapy.

**Trial Registration:**

ClinicalTrials.gov Identifier: NCT02793895

## Key Points

**Question:**

Can continuous cardiac rhythm monitoring beyond hospital discharge enhance atrial fibrillation (AF) detection among cardiac surgical patients?

**Findings:**

In this randomized clinical trial of 336 cardiac surgical patients with risk factors for stroke, use of continuous cardiac rhythm monitoring with wearable sensors increased the rate of AF detection by 17.9% within 30 days of hospital discharge compared with usual care.

**Meaning:**

Among cardiac surgical patients with risk factors for stroke and AF lasting less than 24 hours postoperatively, continuous cardiac rhythm monitoring significantly improved the rate of AF detection during the first 30 days after hospital discharge compared with usual care.

## Teaser

This randomized clinical trial examines whether continuous cardiac rhythm monitoring enhances detection of postoperative atrial fibrillation (AF) among cardiac surgical patients during the first 30 days after hospital discharge compared with usual care.

## Introduction

Postoperative atrial fibrillation (POAF) after cardiac surgery occurs in 30% to 50% of patients during their hospital stay, with the incidence peaking at 3 to 5 days and decreasing afterwards.^1^ However, the incidence of POAF after discharge from cardiac surgery is not well defined. Most studies^2,3,4,5,6,7,8,9,10,11^ have been limited to the hospitalization phase only and were small, nonrandomized, or included patients with antecedent atrial fibrillation (AF) and used limited monitoring for AF. Among cardiac surgical patients who experienced little to no POAF during hospitalization, their risk of experiencing AF after discharge is unknown.^12,13^

Quantifying the risk of ongoing POAF after hospitalization is an important but unanswered issue because many patients have elevated stroke risk (ie, CHA_2_DS_2_-VASc [congestive heart failure, hypertension, age ≥75 years, diabetes, prior stroke or transient ischemic attack, vascular disease, age 65-74 years, female sex] score ≥2); hence, diagnosing AF in this population may be actionable.^14^ In fact, POAF is associated with a 2-fold increase in early adverse outcomes such as stroke and death,^1,2,3^ and the risk of adverse events is known to persist even during long-term follow-up.^1,2^ Because there are sparse clinical trial data, guidelines provide little direction for clinicians on the optimal duration of monitoring, follow-up, or treatment for patients after hospitalization for cardiac surgery, particularly if they are in sinus rhythm at discharge.^15,16^ Randomized data guiding detection and management of POAF after hospitalization for cardiac surgery are lacking. Accordingly, we conducted a randomized clinical trial comparing continuous 30-day electrocardiography (ECG) monitoring with usual care for the detection of AF or atrial flutter (AFL) after hospitalization for cardiac surgery.

## Methods

### Trial Design and Setting

The Post-Surgical Enhanced Monitoring for Cardiac Arrhythmias and Atrial Fibrillation (SEARCH-AF) trial was an investigator-initiated, prospective, open-label, multicenter, randomized clinical trial at 10 tertiary care cardiac surgical centers in Canada. Each site’s research ethics board approved the study, and all participants provided written informed consent. The trial protocol, protocol amendments, and statistical analysis plan are available in Supplement 1. The statistical analysis plan was written without knowledge of outcome data. This report was prepared in accordance with the Consolidated Standards of Reporting Trials (CONSORT) reporting guideline for randomized trials.

### Study Population

Patients were eligible if they were aged 18 years or older, underwent cardiac surgery (coronary artery bypass grafting [CABG] or valve repair or replacement with or without CABG), had no history of AF or AFL before surgery, had AF or AFL lasting less than 24 hours during hospitalization after surgery, and had a CHA_2_DS_2_-VASC score of greater than or equal to 4 or greater than or equal to 2 plus at least 1 additional factor associated with the risk developing POAF (chronic obstructive pulmonary disease, sleep apnea, impaired renal function, left atrial enlargement, elevated body mass index, or combined CABG with valve repair or replacement). Patients were excluded if they had a presurgical history of AF or AFL, had AF or AFL lasting 24 hours or longer after cardiac surgery, were in AF or AFL at the time of randomization, were hospitalized for 10 days or longer at the time of randomization, or received a mechanical valve or had other reason to be on oral anticoagulation. Race/ethnicity was assessed to examine its potential association with the incidence of POAF. Data on race/ethnicity were collected from review of medical records. Detailed inclusion and exclusion criteria are listed in eTable 1 in Supplement 2.

### Trial Procedures

Patients were randomly assigned in a 1:1 ratio to the monitoring group (continuous 30-day ECG monitoring) or to usual care with no mandated monitoring. Randomization lists were prepared by the trial statistician (K.E.T.) and were computer-generated using random permuted blocks. Randomization was stratified by study site and by the type of cardiac surgery performed (isolated CABG or valve replacement or repair with or without CABG). Randomization occurred between the third postoperative day and discharge from hospital. In each site, study coordinators (with oversight from the site principal investigator) enrolled patients in the trial and administered the trial interventions.

Patients randomized to the monitoring group received up to 30 days of continuous ECG monitoring with a wearable, adhesive patch monitor. Until September 30, 2018, the SEEQ system (Medtronic) was used. Afterward, the CardioSTATsystem (Icentia) was used because SEEQ manufacture was halted. Maximum monitoring duration for a single SEEQ or CardioSTAT patch was 7.5 and 14 days, respectively. Patients in the monitoring group received 4 SEEQ or 2 CardioSTAT patches, which provided up to 30 and 28 days of ECG monitoring, respectively. Before hospital discharge, the patch was applied on the anterior left chest, away from the sternotomy site. Patients were provided instruction on monitor use and were contacted weekly (SEEQ) or biweekly (CardioSTAT) to ensure compliance. Used patches were returned to the manufacturer for analysis. A report including all arrhythmic episodes with ECG strips was provided to site investigators and subsequently forwarded to the patient’s primary care physician, cardiologist, and cardiac surgeon at the end of the 28-day or 30-day monitoring period.

Patients randomized to usual care did not undergo any protocol-mandated ECG monitoring within the first 30 days after randomization. This practice is consistent with current guidelines, which make no recommendations for routine monitoring.^15,16,17^ However, if clinically indicated, patients in the usual care group could undergo standard ECG or Holter monitoring within the first 30 days of randomization. If AF or AFL was documented on a 12-lead ECG, performance of additional monitoring, such as Holter monitoring, was suggested. Any AF or AFL detected by an ECG, Holter, or any other type of monitoring performed in the usual care group was included for end point assessment to avoid negatively biasing the event rate in this group. In both groups, therapeutic decisions, such as initiation of oral anticoagulation therapy, were left to local physicians’ discretion.

Patients were followed at 1 to 3 months and 6 months after discharge from surgery on an in-person basis. A telephone-based follow-up was conducted at 9 months after discharge from surgery. At 6 months, patients in both study groups underwent 14 days of continuous cardiac rhythm monitoring (SEEQ or CardioSTAT patch).

### Outcomes

The primary outcome was documentation of cumulative AF or AFL duration of 6 minutes or longer or documentation of AF or AFL by a single 12-lead ECG within 30 days after randomization. A list of prespecified secondary outcomes is provided in eTable 2 in Supplement 2. Rhythm-based outcomes, major adverse cardiovascular events, and major bleeding were adjudicated by a committee of physicians blinded to treatment assignment.

### Statistical Analysis

The trial was designed to test the hypothesis of superiority of continuous ECG monitoring over usual care in the detection of the primary outcome within 30 days after randomization. The rate of the primary outcome was estimated to be 3% during 30 days in the usual care group.^18^ To detect a 7% absolute increase in primary outcome detection with a strategy of continuous cardiac rhythm monitoring, a total of 388 patients (194 in each group) would provide the trial with 80% power at a 2-sided α = .05. Assuming a 2% attrition rate, the final sample size was 396 patients.

Outcome analyses were performed on the intention-to-treat population, which analyzed patients according to their randomization status. The difference in the primary outcome between the 2 study groups was reported as the absolute rate difference with associated 95% CIs. Comparison of the proportion of patients between the 2 groups with the primary outcome was performed with the Pearson χ^2^ test. The Pearson χ^2^ test was used to compare the proportions between groups for secondary outcomes. A subgroup analysis was performed involving 5 prespecified subgroups (sex, age, CHA_2_DS_2_-VASc score, surgery type [isolated CABG vs valve repair or replacement with or without CABG], and left atrial size) to assess for effect modification between the 2 randomization groups. A Kaplan-Meier curve of the primary outcome was generated for illustrative purposes.

A per-protocol analysis was prespecified and consisted of all randomized patients who received their allocated intervention without significant deviations in the assigned treatment during the first 30 days after randomization. For patients randomized to the continuous cardiac rhythm monitoring group, the per-protocol cohort was defined by patients who wore the sensor for 24 hours or longer within the first 30 days after randomization. For patients randomized to the usual care group, the per-protocol cohort was defined by patients who did not wear a continuous cardiac rhythm monitor (SEEQ or CardioSTAT monitor) during the first 30 days after randomization. Statistical analyses were performed with R statistical software version 4.0.2 (R Project for Statistical Computing).

## Results

### Study Population

**Figure 1.**
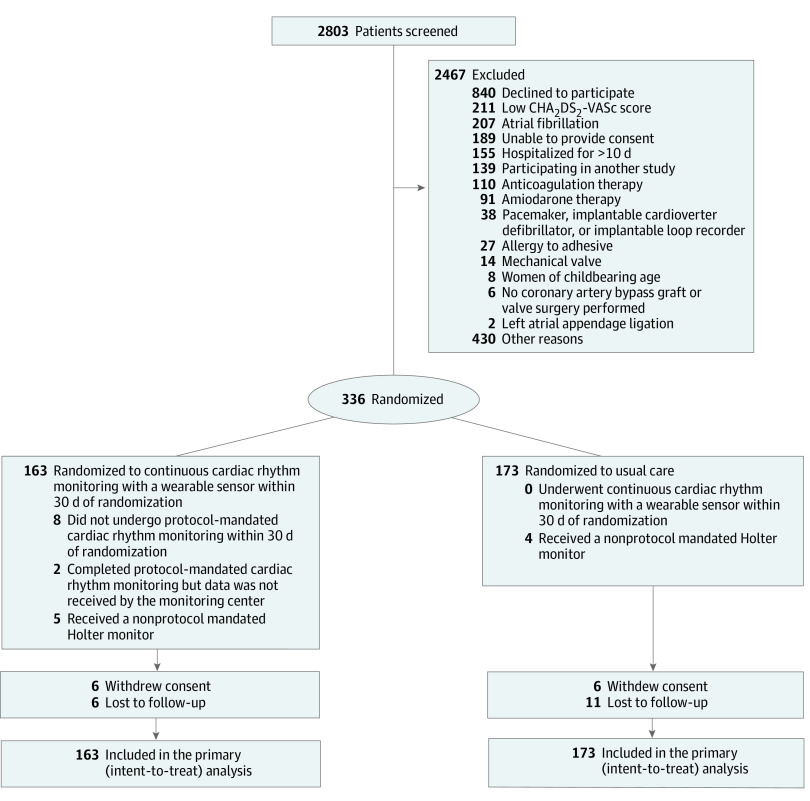
Randomization and Patients in the Post-Surgical Enhanced Monitoring for Cardiac Arrhythmias and Atrial Fibrillation (SEARCH-AF) Trial A total of 2803 patients were screened for the study and 336 patients underwent randomization. In the group that was assigned to receive continuous monitoring, 8 patients did not undergo any monitoring and 2 patients completed the monitoring but the data were not received by the monitoring center. In the usual care group, no patient underwent continuous monitoring and 4 patients underwent non–protocol-mandated Holter monitoring. During follow-up, 6 patients withdrew consent in both groups. There were 6 and 11 patients who were lost to follow-up in the intervention and usual care group, respectively. A total of 163 patients in the monitoring group and 173 patients in the usual care group were included in the primary analysis. A detailed list of reasons for screen failure is provided in eTable 3 in Supplement 2. CHA_2_DS_2_-VASc indicates congestive heart failure, hypertension, age 75 years or older, diabetes, prior stroke or transient ischemic attack, vascular disease, age 65 to 74 years, and female sex.

Between March 2017 and March 2020, 336 patients (mean [SD] age, 67.4 [8.1] years; 73 women [21.7%]) were randomized to continuous monitoring (163 patients) or usual care (173 patients) at 8 Canadian sites (Figure 1). No patients were randomized at 2 sites. As a result of the COVID-19 pandemic, all sites mandated institutional restrictions on surgical procedures and research recruitment. On July 17, 2020, enrollment in the study was terminated, at which time 336 patients were enrolled, accounting for 85% of the planned sample size. The last protocol-mandated clinical follow-up occurred on August 31, 2020, and the last follow-up related to results of cardiac rhythm monitoring occurred on September 11, 2020. The median (IQR) time from surgery to randomization was 5 (4-6) days, and the median (IQR) time from randomization to discharge was 0 (0-1) days.

There was no statistically significant difference in the baseline characteristics of the 2 assigned groups (Table 1). The median (IQR) CHA_2_DS_2_-VASc score was 4.0 points (3.0-4.0 points); 255 patients (75.9%) underwent CABG alone, 39 patients (11.6%) underwent valve repair or replacement alone, and 42 patients (12.5%) underwent both CABG and valve repair or replacement. At hospital discharge, amiodarone was prescribed for 13 patients (7 in the monitoring group and 6 in the usual care group). None of the patients had a presurgical history of AF or AFL by design, and 318 patients (94.6%) had no documented AF during hospitalization. The duration of in-hospital AF in the remaining 18 patients (5.4%) was less than 24 hours.

**Table 1.** Baseline Demographic and Clinical Characteristics

Characteristic	Participants, No. (%) (N = 336)	
	Continuous monitoring (n = 163)	Usual care (n = 173)
Age, mean (SD), y	67.5 (8.1)	67.4 (8.2)
Sex		
Female	35 (21.5)	38 (22.0)
Male	128 (78.5)	135 (78.0)
Race/ethnicity		
White	133 (81.6)	147 (85.0)
Asian	19 (11.7)	19 (11.0)
Black	4 (2.5)	3 (1.7)
First Nations	1 (0.6)	2 (1.2)
Hispanic or Latino	3 (1.8)	1 (0.6)
Other^a^	3 (1.8)	1 (0.6)
Body mass index, mean (SD)^b^	30.8 (6.3)	31.1 (5.8)
Hypertension	151 (92.6)	162 (93.6)
Diabetes	90 (55.2)	86 (49.7)
Smoking history	102 (62.5))	114 (65.9)
Sleep apnea	26 (16.0)	28 (16.2)
Heart failure	10 (6.1)	13 (7.5)
Stroke or transient ischemic attack	17 (10.4)	18 (10.4)
Myocardial infarction	65 (39.9)	60 (34.7)
Percutaneous coronary intervention	24 (14.7)	29 (16.8)
Chronic obstructive pulmonary disease	11 (6.7)	16 (9.2)
Valvular disease^c^		
Aortic	37 (22.7)	35 (20.2)
Mitral	2 (1.2)	2 (1.2)
Aortic and mitral	1 (0.6)	1 (0.6)
CHA_2_DS_2_-VASc score, median (IQR)	4.0 (3.0-4.5)	4.0 (3.0-4.0)
HAS-BLED score, median (IQR)	2.0 (2.0-3.0)	2.0 (2.0-3.0)
Coronary artery bypass surgery only	124 (76.1)	131 (75.5)
Valve repair or replacement only	17 (10.4)	22 (12.7)
Coronary artery bypass plus valve surgery	22 (13.5)	20 (11.6)
Ejection fraction, mean (SD), %	55.1 (10.4)	57.4 (9.8)
Left atrial enlargement^d^	63 (38.7)	64 (37.0)
Atrial fibrillation (<24 h) during hospitalization after cardiac surgery	6 (3.7)	12 (6.9)

Abbreviations: CHA_2_DS_2_-VASc, congestive heart failure, hypertension, age 75 years or older, diabetes, prior stroke or transient ischemic attack, vascular disease, age 65 to 74 years, female sex (1 point each except age ≥75 years and stroke which are 2 points); HAS-BLED, hypertension, abnormal liver or renal function, stroke, bleeding, labile international normalized ratio, elderly (age >65 years), and drugs or alcohol; IQR, interquartile range.

^a^ Other refers to Pacific Islander or multiracial.

^b^ Body mass index is calculated as weight in kilograms divided by height in meters squared.

^c^ The presence of valvular heart disease was defined as having at least a stenotic or regurgitant lesion of at least moderate severity or if the patient had previous bioprosthetic valve replacement or valve repair. Preoperative echocardiographic information within 12 months of surgery was available for 244 patients, and left atrial size was reported for 187 patients.

^d^ Defined as greater than 41 mm diameter or volume greater than or equal to 59 mL or volume index greater than or equal to 29 mL/m^2^.

All study-related follow-up visits were attended by 307 patients (91.4%). The median (IQR) follow-up was 276 (267-293) days in the continuous cardiac rhythm monitoring group and 277 (267-292) days in the usual care group. Among the 163 patients in the monitoring group, 8 patients (4.9%) did not undergo continuous cardiac rhythm monitoring and 2 patients (1.2%) completed continuous cardiac rhythm monitoring but the data were not received by the monitoring center (Figure 1). Of the remaining 153 patients in the intervention group, 79 patients (51.6%) wore the monitor for at least 28 days and 30 patients (19.6%) wore the monitor for 21 to 27 days. Reasons for screen failure are shown in eTable 3 in Supplement 2, and reasons for premature termination of monitoring are listed in eTable 4 in Supplement 2. In the continuous cardiac rhythm monitoring group, 17 patients (10.4%) experienced a device-related adverse event due to skin irritation from the adhesive material of the wearable patch within 30 days of randomization. None of these device-related adverse events resulted in a serious adverse event. Four patients (2.3%) in the usual care group underwent Holter monitoring within 30 days of randomization.

### Primary Outcome

**Figure 2.**
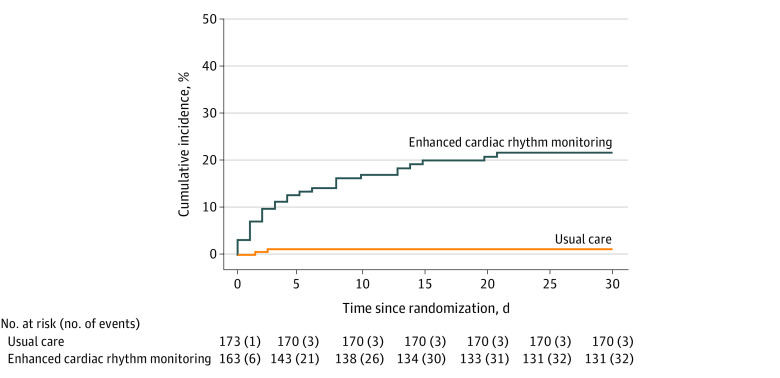
Time to First Event Curves for the Primary End Point Kaplan-Meier estimates of detection of cumulative atrial fibrillation (AF) or atrial flutter (AFL) lasting 6 minutes or longer or documentation of AF or AFL by a single 12-lead electrocardiogram within 30 days after randomization. The primary end point occurred in 32 patients (19.6%) in the monitoring group and in 3 patients (1.7%) in the usual care group for an absolute difference of 17.9% (95% CI, 11.5%-24.3%; *P* < .001).

In the continuous monitoring group, the primary outcome was detected in 32 of 163 patients (19.6%) compared with 3 of 173 patients (1.7%) in the usual care group, with an absolute difference of 17.9% (95% CI, 11.5%-24.3%; *P* < .001) (Table 2 and Figure 2).The number needed to screen to detect the primary outcome was 6 patients (95% CI, 4-9 patients). Of the 32 patients in the monitoring group in whom the primary outcome occurred, 30 of them had at least 1 episode of detected AF or AFL lasting for 6 minutes or longer.

**Table 2.** Primary and Secondary Outcomes by Intention-to-Treat Analysis

Outcome	Participants, No. (%)		Rate difference, % (95% CI)
	Continuous monitoring (n = 163)	Usual care (n = 173)	
Primary outcome: patients with a cumulative duration of AF or AFL lasting ≥6 min or 12-lead ECG demonstrating AF or AFL detected in first 30 d	32 (19.6)	3 (1.7)	17.9 (11.5 to 24.3)^a^
Components of the primary outcome			
Patients with a cumulative duration of AF or AFL lasting ≥6 min in first 30 d	30 (18.4)	0	18.4 (12.5 to 24.4)^a^
Patients with at least 1 episode of 12-lead ECG detected AF or AFL in first 30 d	6 (3.7)	3 (1.7)	1.9 (–1.5 to 5.4)
Secondary outcomes			
Patients with cumulative duration of AF or AFL lasting ≥6 h in first 30 d	14 (8.6)	0	8.6 (4.3 to 12.9)^a^
Patients with cumulative duration of AF or AFL lasting ≥24 h in first 30 d	5 (3.1)	0	3.1 (0.4 to 5.7)
Patients with non–protocol-mandated Holter or event recorder in first 30 d	5 (3.1)	4 (2.3)	0.8 (–2.7 to 4.2)
Patients with prescription of oral anticoagulation in first 45 d	7 (4.3)	4 (2.3)	2.0 (–1.9 to 5.8)
Patients with prescription of oral anticoagulation after 45 d	6 (3.7)	5 (2.9)	0.8 (–3.0 to 4.6)
Patients with major bleeding in first 45 d	0	1 (0.6)	–0.6 (–1.7 to 0.6)
Patients with major bleeding after 45 d	1 (0.6)	3 (1.7)	–1.1 (–3.4 to 1.2)
Patients who experienced death, myocardial infarction, ischemic stroke, or non-CNS thromboembolism in first 45 d	1 (0.6)	1 (0.6)	0.0 (–1.6 to 1.7)
Patients who experienced death, myocardial infarction, ischemic stroke, or non-CNS thromboembolism after 45 d	1 (0.6)	3 (1.7)	–1.1 (–3.4 to 1.2)
Patients who experienced adverse events associated with the use of protocol-mandated monitoring within 30 d after randomization	17 (10.4)	0	10.4 (5.7 to 15.1)^a^

Abbreviations: AF, atrial fibrillation; AFL, atrial flutter; CNS, central nervous system; ECG, electrocardiogram.

^a^ *P* < .001.

**Figure 3.**
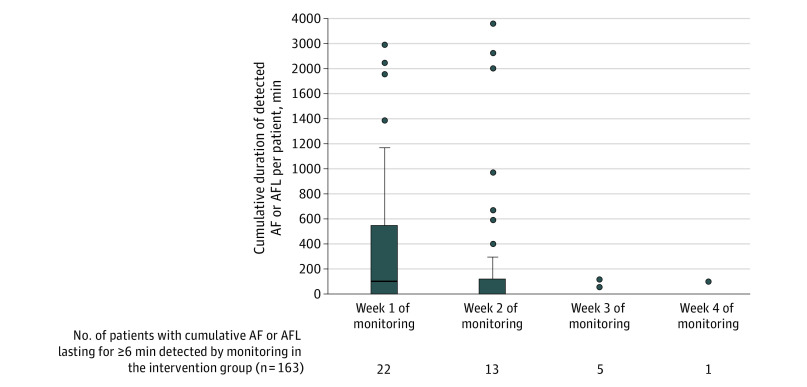
Cumulative Duration of Detected Atrial Fibrillation (AF) or Atrial Flutter (AFL) Episodes (per Patient) Within the First 30 Days of Randomization Box and whisker plots of the cumulative duration of detected AF or AFL durations for patients randomized to continuous cardiac rhythm monitoring within the first 30 days of randomization. The upper and lower ends of the box represent the first and third quartile. The line in the box represents the median. In week 2, the median was 0.2 minutes. In week 1, the whisker above the upper quartile is drawn up to the largest observed point that falls within 1.5 times of the interquartile range (IQR). In week 2, the whisker is 1.5 times of the IQR. The whiskers below the lower quartile are not illustrated because they cross zero. Outliers are presented as circles located outside the whiskers. There were 10 patients who had AF lasting 6 minutes or longer in multiple monitoring weeks.

In the monitoring group, the primary outcome was detected by continuous monitoring in 30 of 32 patients. For the other 2 patients, AF was detected by 12-lead ECG. The first episode of AF or AFL lasting for 6 minutes or longer was detected in 22 patients (73.3%) during the first week of monitoring, in 6 patients (20.0%) during the second week, and in 2 patients (6.7%) during the third week. The cumulative duration of detected AF or AFL episodes and the number of patients with detected AF or AFL lasting for 6 minutes or longer, stratified by monitoring weeks, is shown in Figure 3. Cumulative AF or AFL lasting 6 minutes or longer was detected in 10 patients in multiple monitoring weeks. Additional details of detected AF durations are described in eTable 5 in Supplement 2. Among the 3 patients with detected AF in the usual care group, AF was documented by a 12-lead ECG within 2 days of randomization.

### Secondary Outcomes

Within the first 30 days, cumulative AF or AFL lasting 6 hours or longer was detected in 14 patients (8.6%) in the monitoring group and 0 patients in the usual care group (absolute difference, 8.6%; 95% CI, 4.3%-12.9%; *P* < .001) (Table 2). Cumulative AF or AFL lasting 24 hours or longer within the first 30 days was detected in 5 patients (3.1%) in the monitoring group and 0 patients in the usual care group (absolute difference, 3.1%; 95% CI, 0.4%-5.7%) (Table 2). Other descriptive outcomes of episode durations are provided in eTable 6 in Supplement 2.

During the first 45 days after discharge, a total of 11 patients (3.3%) were prescribed oral anticoagulation therapy (7 patients [4.3%] in the monitoring group and 4 patients [2.3%] in the usual care group) (Table 2). Between 46 days after discharge and the end of follow-up, oral anticoagulation therapy was prescribed for 11 patients (3.3%) (6 patients [3.7%] in the monitoring group and 5 patients [2.9%] in the usual care group) (Table 2).

After discharge, major adverse cardiovascular events occurred in 6 patients (1.8%), 2 patients (1.2%) in the monitoring group and 4 patients (2.3%) receiving usual care (Table 2). Two patients experienced an ischemic stroke during study follow-up, and AF was detected in 1 of those patients.

At 6 months, 189 patients (56.3%) underwent protocol-mandated continuous cardiac rhythm monitoring with a 2-week, patch-based monitor. Among these 189 patients, cumulative AF or AFL lasting for 6 minutes or longer was detected in 3 patients (1.6%) at 6 months after surgery. Of the entire cohort of 336 patients, cumulative AF or AFL lasting for 6 minutes or longer by continuous cardiac rhythm monitoring or AF or AFL documented by a 12-lead ECG was detected among 7 patients (2.1%) between 31 days after randomization and end of study follow-up (eTable 7 in Supplement 2). The complete list of secondary outcomes is available in eTable 8 in Supplement 2.

### Subgroup Analysis and Per-Protocol Analysis

The treatment effect of the study intervention was similar between the prespecified subgroups. Evaluation of effect modification could not be assessed between men and women because there were 0 events among women in the control group (eFigure in Supplement 2). Results of the per-protocol analysis were similar to the intent-to-treat analysis (eTable 9 in Supplement 2). An exploratory analysis examining the association between clinical factors and the primary end point is provided in eTable 10 in Supplement 2. Results of 2 additional exploratory analyses (detection of AF with 30 days of randomization according to occurrence of POAF before randomization and according to type of wearable sensor used) are provided in eTable 11 and eTable 12 in Supplement 2.

## Discussion

In this randomized clinical trial, we found that continuous monitoring during the first 30 days after hospitalization for cardiac surgery detected significantly more POAF than usual care among patients at high risk of stroke with no presurgical history of AF who had AF for less than 24 hours while hospitalized. Similar findings were observed for detection of longer AF durations of 6 hours or longer and 24 hours or longer. Most patients in this study had no POAF detected during hospitalization after cardiac surgery. These findings demonstrate that POAF after cardiac surgery is not confined to the hospitalization period. A high rate of AF can be detected in the first month after surgery.

POAF has long been considered an early, transient phenomenon after cardiac surgery because of pericardial inflammation, cardiac ischemia, hemodynamic fluctuations, and high adrenergic state, which is assumed to resolve in days.^19^ Although POAF after cardiac surgery is a common clinical problem, most studies^20,21^ have only described its incidence during hospitalization. The incidence of POAF occurring weeks to months after surgery is not well-defined; prior studies^3,4,5,6,7,8,9,10,11^ are few, retrospective, or nonrandomized. This represents an important unknown for clinicians managing these patients. In our study, continuous cardiac rhythm monitoring detected AF in nearly 1 of 5 patients within 30 days after discharge and was markedly higher than the rate of detected AF in the usual care group. This indicates that a substantial proportion of POAF is subclinical and would not be diagnosed without the use of continuous cardiac rhythm monitoring. In this study, patients had AF lasting less than 24 hours during hospitalization and would likely not have prompted any further monitoring or treatment under normal clinical circumstances. In this patient population, continuous ECG monitoring was essential to uncover the ongoing risk and extent of outpatient POAF. In particular, the occurrence of POAF was clustered within the first 2 weeks after discharge in our study cohort.

In our study, the rate of AF detection with continuous cardiac rhythm monitoring was substantially higher than that in patients in the nonsurgical setting and with a similar risk profile. The CHA_2_DS_2_-VASc score is associated with development of AF in the nonsurgical setting.^22,23^ Among patients with a CHA_2_DS_2_-VASc score similar to that for our cohort (median, 4.0 points), AF incidences of 0.9 to 1.5 cases per 100 patient-years were reported, using limited modalities to ascertain AF.^22,23^ In a study^24^ using continuous monitors in a nonsurgical population with a CHA_2_DS_2_-VASc score similar to that of our population, the detection rate of new-onset AF was 6.2% after 30 days. Compared with these studies, our 30-day AF detection rate of 19.6% was much higher than that in a general, matched population.

The rate of oral anticoagulation therapy in our patients was low. This was consistent with published studies^25,26^ showing that rates of oral anticoagulation therapy after cardiac surgery were low despite a high incidence of in-hospital POAF. Reluctance to prescribe anticoagulation therapy may be related to concerns over bleeding, the belief that POAF is self-limiting, or uncertainty regarding the duration of AF that would justify the use of oral anticoagulation for stroke prevention in this patient population. In our population with a median CHA_2_DS_2_-VASc score of 4 and AF lasting 6 minutes or longer, the annual stroke risk is estimated to be 1.3%,^27^ which would meet the threshold for oral anticoagulation therapy. Reluctance to prescribe oral anticoagulation therapy may reflect uncertainty in clinical practice guidelines as current recommendations for oral anticoagulation therapy after discharge are limited by lack of randomized trial data, especially if patients are in sinus rhythm.^15,16^ Our data may help inform these guidelines in terms of the optimal method for POAF detection after discharge.

Our findings suggest that the incidence of POAF may decrease rapidly over time because the rate of AF detection markedly declined after the first 2 weeks of hospital discharge. However, previous studies^1,13,14,20,28^ found an association between POAF and cardiovascular risk over long-term follow-up, when, presumably, POAF had largely resolved. Future research is needed to assess whether early anticoagulation may mitigate adverse short-term and long-term cardiovascular outcomes.^29^

### Limitations

This study has limitations that should be addressed. The primary end point is limited to 30 days and does not estimate the long-term, ongoing risk of POAF. Among patients with POAF lasting less than 24 hours, we did not collect the exact duration of AF and, hence, could not assess the association between in-hospital AF burden and the primary end point. Even with limited continuous monitoring, some patients did not complete it and reported a lack of interest as the primary reason. Our trial, by design, excluded patients with longer episodes of AF (≥24 hours) while hospitalized and those with prolonged hospital stay. The rates of AF occurrence and cardiovascular events might be higher if these patients were included in this study. We did not perform preoperative cardiac monitoring to rule out the presence of AF before surgery; subclinical AF could be prevalent among certain patient subsets, such as those with mitral valve disease. This was beyond the scope of this study. Lack of adherence with the monitoring by some patients led to underestimation of AF. The planned sample size and follow-up duration were not powered to detect for differences in major adverse cardiovascular outcomes, stroke rates in comparison with the presence of POAF, or whether oral anticoagulation therapy would alter the risk of such ischemic outcomes.

## Conclusions

Among cardiac surgical patients with an elevated risk of stroke and no history of preoperative or predischarge AF, a strategy of continuous cardiac rhythm monitoring unveiled a significant burden of unrecognized AF compared with usual care, specifically within the first 2 weeks after discharge. Future studies are required to assess whether oral anticoagulation therapy is safe and effective in this patient population.

## Notes

Supplement 1.
Trial Protocol

Supplement 2. **eTable 1.** SEARCH-AF Inclusion/Exclusion Criteria Inclusion Criteria **eTable 2.** SEARCH-AF Secondary Endpoints **eTable 3.** Reasons for Screen Failure in the SEARCH-AF Trial **eTable 4.** Reasons for Premature Termination of Monitoring **eTable 5.** Description of Detected AF Duration by Continuous Cardiac Rhythm Monitoring Within 30 Days of Randomization, Stratified by Week of Monitoring **eTable 6.** Rhythm-Based Outcomes Within 30 Days of Randomization **eTable 7.** Detection of Atrial Fibrillation/Flutter After 30 Days of Randomization **eTable 8.** Results of all Secondary Endpoints (Intent-to-Treat Cohort) **eTable 9.** Per Protocol Analysis **eTable 10.** Factors Associated with Occurrence of the Primary Endpoint **eTable 11.** Detection of Atrial Fibrillation Within 30 Days of Randomization in Relation to Presence of Post-Operative Atrial Fibrillation Occurring Prior to Randomization **eTable 12.** Detection of Atrial Fibrillation Within the First 30 Days After Randomization, According to Type of Wearable Sensor Employed **eFigure. **Primary Outcome According to Pre-Specified Subgroups

Supplement 3.
SEARCH AF CardioLink-1 Investigators

Supplement 4.
Data Sharing Statement
