# Supplementary material for: Effect of Continuous Electrocardiogram Monitoring on Detection of Undiagnosed Atrial Fibrillation After Hospitalization for Cardiac Surgery: A Randomized Clinical Trial
Source: JAMA Netw Open. 2021 Aug 27;4(8):e2121867. doi: 10.1001/jamanetworkopen.2021.21867 (PMC8397929; doi:10.1001/jamanetworkopen.2021.21867)
Supplement: Supplement 1. — Trial Protocol [file jamanetwopen-e2121867-s001.pdf]

Post-Surgical Enhanced Monitoring for Cardiac Arrhythmias and Atrial Fibrillation (SEARCH-AF): A randomized controlled trial

Protocol number: SEARCH-AF-001

Executive steering committee:
Andrew C. T. Ha, MD, MSc; Atul Verma, MD; C. David Mazer, MD; Bobby Yanagawa, MD, PhD; Dr. Subodh Verma, MD, PhD.

Coordinating centre:
Applied Health Research Centre, Li Ka Shing Knowledge Institute
St. Michael's Hospital
30 Bond Street, Toronto, Ontario, Canada.
M5B1W8

Version date:
December 18, 2015.

Study sponsor:
St. Michael's Hospital
30 Bond Street, Toronto, Ontario, Canada.
M5B1W8

INVESTIGATOR STATEMENT AND SIGNATURE

Protocol Number: SEARCH-AF-001

Post-Surgical Enhanced Monitoring for Cardiac Arrhythmias and Atrial Fibrillation (SEARCH-AF): A randomized controlled trial

I have read the protocol described herein. I agree to conduct this study in compliance with the protocol, Good Clinical Practice, and other applicable regulatory requirements.

Site Principal Investigator:

Signature: ____

Date: ____

Printed Name: ____________________________________

Site Name: _______________________________________

This protocol has been developed by the Principal Investigators and its contents are the intellectual property of this group. It is an offence to reproduce or use the information and data in this protocol for any purpose other than the SEARCH-AF trial without prior approval from the Principal Investigators.

Signature Page

Dr. Andrew C.T. Ha<br>Principal Investigator<br>St. Michael's Hospital	Name	2015-Dec-18<br>Date *(YYYY-MM-DD)*
Dr. Atul Verma<br>Principal Investigator<br>St. Michael's Hospital	Name	2015-Dec-18<br>Date *(YYYY-MM-DD)*
Dr. Subodh Verma<br>Principal Investigator<br>St. Michael's Hospital	Name	2015-Dec-18<br>Date *(YYYY-MM-DD)*

Table of Contents **Page**

1. Background and Rationale..11

2. Study Objectives and Hypothesis..15

2.1 Primary Objective
2.2 Secondary Objectives
2.3 Study Hypothesis

3. Ethical considerations…………………………………………………….……...16

3.1 Good Clinical Practice (GCP)
3.2 Research Ethics Board/Institutional Review Board
3.3 Informed Consent Process
3.4 Participant Confidentiality

4. Eligibility Criteria...17

4.1 Inclusion Criteria
4.2 Exclusion Criteria
4.3 Women of childbearing potential
4.4 Discontinuation of Subjects from Trial Participation

5. Study Design...19

5.1 Study Description
5.2 Executive Committee
5.3 Clinical Events Committee
5.4 Baseline Visit
5.5 Randomization
5.6 Proposed Study Intervention
5.6.1 30-day continuous cardiac rhythm monitor (Intervention group)
5.6.2 Usual care (control group)
5.6.3 Additional 14-day cardiac rhythm monitoring at 6±1 months
5.7 Methods to Protect against Potential Bias
5.8 Proposed Duration of the Intervention Period
5.9 Proposed Frequency and Duration of Follow-up
5.10 Study Flowchart
5.11 Primary Outcome
5.12 Secondary Outcomes
5.13 Prescription of Oral Anticoagulation
5.14 Other Considerations

6. Adverse events...29

6.1 Problem Reporting
6.2 Serious adverse events

7. Study Duration……...29

7.1 Expected Study Duration of Subject Participation

7.2 Expected Total Study Duration

8. Concomitant Medications/Natural Remedies/Foods...29

9. Prohibited Medications and Procedures..30

10. Study Evaluations/Procedures...30
11.1 Laboratory Evaluations and Specimen Collection
11.2 Questionnaires

11. Trial Management...30

12. Statistical considerations..31
12.1 Sample Size Calculation
12.2 Details of Statistical Analysis
12.3 Subgroup Analysis

13. References…………………………..…………………………………..……....34

14. Appendices…………………………………………………………………...38

LIST OF ABBREVIATIONS

ACC	American College of Cardiology
AE	Adverse event
AF	Atrial fibrillation
AFL	Atrial flutter
AHA	American Heart Association
ASSERT-II	Prevalence of sub-Clinical atrial fibrillation using an implantable cardiac monitor in patients with cardiovascular risk factors detected
BMI	Body mass index
CABG	Coronary artery bypass grafting surgery
CEC	Clinical events committee
CCS	Canadian Cardiovascular Society
CI	Confidence interval
CIHR	Canadian Institutes of Health Research
CNS	Central nervous system
COPD	Chronic obstructive pulmonary disease
CRYSTAL-AF	Study of continuous cardiac monitoring to assess atrial fibrillation after cryptogenic stroke
CVA	Cerebrovascular accident
ECG	Electrocardiogram
EMBRACE	30-day cardiac event monitor belt for recording atrial fibrillation after a cerebral ischemic event
ESC	European Society of Cardiology
HRS	Heart Rhythm Society
ICD	Implantable cardioverter defibrillator
ILR	Implantable loop recorder
INR	International normalized ratio
IRB	Institutional review board
LV	Left ventricular
MACE	Major adverse cardiac events
OAC	Oral anticoagulation
OR	Odds ratio
POAF/AFL	Post-operative atrial fibrillation / atrial flutter
PCI	Percutaneous coronary intervention
PI	Principal investigator
PVC	Premature ventricular contraction
RCT	Randomized controlled trial
REB	Research ethics board
SAE	Serious adverse event
TIA	Transient ischemic attack
WOCBP	Women of childbearing potential

Protocol Synopsis

**Project title**	**Post-Surgical Enhanced Monitoring for Cardiac Arrhythmias and Atrial Fibrillation (SEARCH-AF)**
**Short title**	SEARCH-AF
**Study sponsor**	St. Michael's Hospital, Toronto, Ontario, Canada.
**Recruitment and Participating sites**	A total of 396 subjects will be recruited from cardiac surgical centres in Canada.
**Phase / Regulatory status**	Phase IV / Randomized Controlled Trial.
**Study Hypothesis**	Among post-cardiac surgical subjects without a previous history of AF/AFL and with risk factors for stroke and POAF/AFL, a strategy of enhanced cardiac rhythm monitoring will result in a 3-fold or 7% absolute increase in the rate of AF/AFL detection (defined as a cumulative AF/AFL burden of ≥6 minutes or documentation of AF/AFL by a 12-lead ECG) when compared to usual care at 30 days after randomization.
**Study Objective**	The aim of SEARCH-AF is to evaluate a novel diagnostic tool for detecting POAF/AFL in cardiac surgical subjects during the early, sub-acute post-operative period.<br><br>***Primary objective:***<br>1) To test whether enhanced cardiac rhythm monitoring with an adhesive, continuous monitor results in higher rates of AF/AFL detection at 30 days after randomization for post-cardiac surgical subjects who are at risk for developing post-operative atrial arrhythmias, when compared to usual care.<br><br>***Secondary objectives:***<br>1) To assess the incidence of AF/AFL during the early, sub-acute post-operative period (defined as the first 30 days after randomization) and at 6±1 months after surgery.<br>2) To assess the proportion of subjects with continuous AF and/or AFL lasting ≥ 24 hours during the early, sub-acute post-operative period and at 6±1 months after surgery.<br>3) To assess subjects' adherence and incidence of adverse events from use of the Medtronic SEEQ™ system.

	4) To assess the proportion of subjects who are prescribed with oral anticoagulation at 45 days after discharge from cardiac surgery, at 6±1 months, and at 9±1 months after surgery.<br>5) To assess the proportion of subjects who experienced major bleeding events at 45 days after discharge from cardiac surgery.<br>6) To evaluate factors associated with development of POAF/AFL during the early, sub-acute post-operative period.
**Study Design**	Open-label, two-arm RCT comparing a strategy of enhanced cardiac rhythm monitoring vs. usual care in 396 post-cardiac surgical subjects who are at risk for developing POAF/AFL.
**Primary endpoint**	Proportion of subjects with a cumulative AF/AFL burden of ≥6 minutes or documentation of AF/AFL by a 12-lead ECG within 30 days after randomization. Clinical endpoints will be adjudicated by an independent committee of physicians.
**Sample size**	N=396 (198 per group, after accounting for a 2% attrition rate).
**Participants / Study Duration**	A total of 396 subjects will be enrolled over a 36-month period. The last subject enrolled will have a 9-month follow-up after hospital discharge, extending the total study duration to 45 months.
**Study Population**	Post-cardiac surgical subjects at high risk of stroke, defined as having a CHA_2DS_2-VASC score of ≥4 or CHA_2DS_2-VASC of ≥2 with additional risk factors for developing POAF/AFL. These subjects must not have a history of AF/AFL pre-operatively.
**Inclusion Criteria**	1) Male or female age ≥18 years.<br>2) Isolated CABG or valve replacement/repair ± CABG performed at the index surgical procedure.<br>3) At high risk of stroke and developing POAF/AFL, defined as having a CHA_2DS_2-VASC score of ≥4.<br>*or*<br>A CHA_2DS_2-VASC score of ≥2 with at least 1 of the following risk factors for developing POAF/AFL:<br>(i) Chronic obstructive pulmonary disease.<br>(ii) Sleep apnea.<br>(iii) Impaired renal function (defined as creatinine clearance <60 ml/min).<br>(iv) Echocardiographic evidence of at least mild left atrial

	enlargement (defined as ≥41 mm on M-mode, ≥59 ml or ≥29 mL/m^2 on biplane volume assessment).<br>(v) Elevated body mass index (defined as BMI ≥30).<br>(vi) Combined surgery (CABG + valve repair/replacement).
**Exclusion Criteria**	1) Atrial fibrillation or flutter at the time of randomization.<br>2) Known previous history of AF/AFL, diagnosed pre-operatively (note: documentation of a history of AF/AFL without accompanying rhythm proof will suffice).<br>3) Documentation of continuous AF/AFL for ≥24 hours during the in-hospital stay for the index cardiac surgery.<br>4) Subjects who, at the discretion of the treating cardiac surgery team, would be treated and discharged with oral anticoagulation due to POAF/AFL.<br>5) Mechanical valve replacement.<br>6) Current or anticipated treatment with oral anticoagulation for indications other than AF/AFL.<br>7) Hospitalization for ≥10 days (for the index cardiac surgery, with day #0 being the day of surgery).<br>8) Planned discharge from hospital with a type IC or III anti-arrhythmic drug.<br>9) Having received >5 grams of IV and/or oral amiodarone during hospitalization for the index cardiac surgical procedure.<br>10) Women of childbearing potential (WOCBP).<br>11) History of percutaneous or surgical left atrial ablation for AF.<br>12) Presence of a cardiac implantable electronic device with a functioning atrial lead (pacemaker, implantable cardioverter defibrillator, or cardiac resynchronization device).<br>13) Presence of an implantable loop recorder.<br>14) History of left atrial appendage ligation, removal, or occlusion.<br>15) Subjects with known allergies or hypersensitivities to adhesives or hydrogel.<br>16) Inability to provide written informed consent.<br>17) Current or anticipated participation in another randomized controlled trial in which the interventional drug or device is known to affect the incidence of the primary or secondary outcomes of this study.
**Intervention**	Subjects who meet inclusion criteria will be randomized in a 1:1 ratio to one of the following 2 arms:<br><br>***Enhanced cardiac rhythm monitoring (intervention group)***<br>▪ Starting on the day of randomization, subjects will undergo 30 days of

	continuous cardiac rhythm monitoring with the Medtronic SEEQ™ mobile cardiac telemetry system. They will receive another 14 days of continuous cardiac rhythm monitoring at 6±1 months after hospital discharge.<br><br>***Usual care (control group)***<br>▪ Subjects will not receive any form of protocol-mandated continuous cardiac rhythm monitoring until at 6±1 months after hospital discharge. Performance of ECG and/or Holter monitoring will be left at the discretion of the subjects' treating physicians.<br><br>At 6±1 months after surgery, subjects in both groups will undergo 14 days of continuous cardiac rhythm monitoring with the SEEQ™ mobile cardiac telemetry system.
**Follow-up**	All subjects will have a follow-up visit at 45-52 days after hospital discharge and at 6±1 months after surgery. A 12-lead ECG will be performed for all subjects at follow-up visits. A telephone follow-up will occur at 9±1 months after surgery.
**Study flow chart**	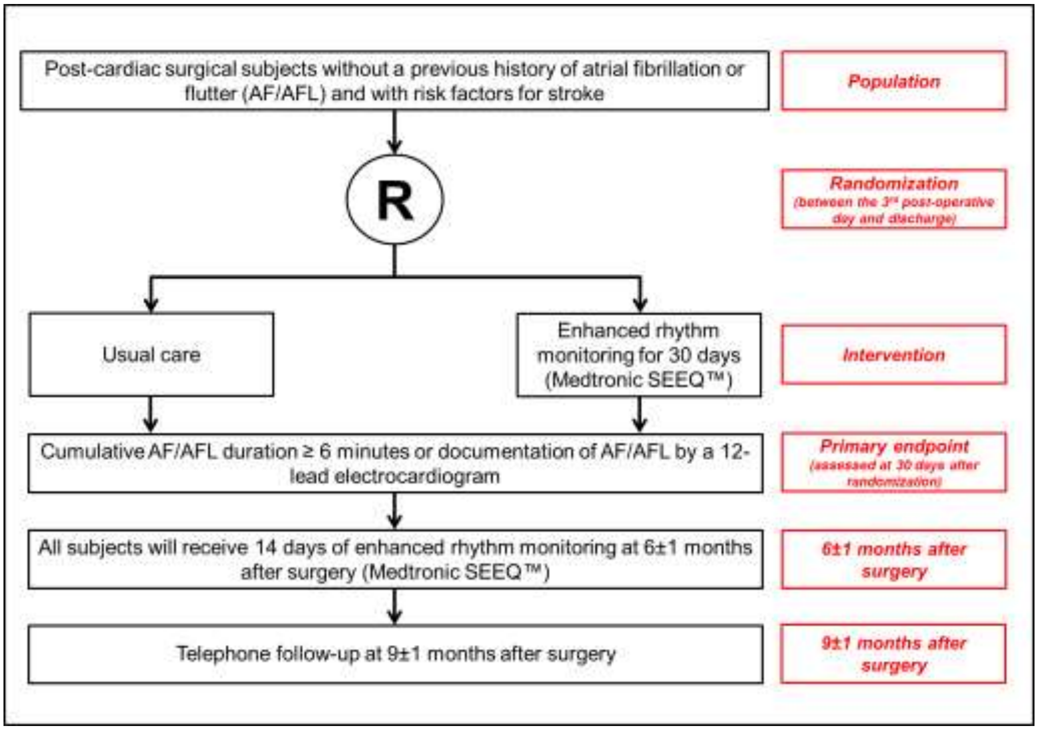

1. BACKGROUND AND RATIONALE

1.1 *The incidence of stroke after cardiac surgery is not trivial*

Stroke is a serious complication after cardiac surgery and its incidence in the post-operative phase has remained unchanged despite advances in surgical techniques. Data from administrative databases and observational registries suggest that the incidence of post-operative stroke after cardiac surgery ranges from 0.8 to 5.2% [1]. Given that millions of cardiac surgical procedures are performed worldwide, postoperative stroke poses significant functional and/or economic burden to patients, their families, and healthcare systems.

1.2 New-onset POAF is an important risk factor for stroke in cardiac surgical patients

Several risk factors have been identified for post-operative stroke in the cardiac surgical population. Older age, renal insufficiency, diabetes, left ventricular (LV) dysfunction, hypertension, low cardiac output syndrome, and atrial fibrillation (AF) had been found to be associated with increased stroke risk in the post-operative phase. In particular, new-onset post-operative atrial fibrillation (POAF) is an important risk factor for stroke. Administrative data from Ontario, Canada suggested that new-onset POAF was associated with a 50% increase in peri-operative stroke risk [2]. A systematic review of 11 cohort studies evaluating the clinical impact of new-onset POAF demonstrated a doubling of in-hospital, peri-operative stroke risk (OR 2.23 [95% CI, 1.78 to 2.80]) [3]. The presence of new-onset POAF is also associated with a greater risk of stroke beyond the peri-operative phase. In a retrospective Californian cohort derived from administrative claims data of ≈77,000 cardiac surgical patients, the risk of stroke at 1 year after hospitalization for the index surgery was higher in those with new-onset POAF than those without [4]. Finally, emerging data suggest that new-onset POAF may be a harbinger for future AF at >1 year after cardiac surgery, well beyond the post-operative phase [5]. Taken together, new-onset POAF is an important risk factor for stroke in the cardiac surgical population during the peri-operative phase and beyond. Given that POAF is so common after cardiac surgery (15-48%) [6.18], effective and timely diagnosis of AF in this population may expedite delivery of appropriate stroke prevention therapies. This is particularly relevant for those at high risk for stroke.

1.3 The incidence of POAF is not well-defined beyond hospital discharge

There have been many studies describing the incidence of POAF in the post-cardiac surgical setting, with reported rates ranging from 15-48% [6-18]. The incidence of POAF may be even higher for patients undergoing valve surgery, with reported rates of up to 60% [7,9,16]. However, it is important to note that the great majority of these studies reported ***in-hospital*** rates of POAF. **As such, there is very little data on the incidence of POAF in the sub-acute, post operative phase after hospital discharge (e.g. within the first month after hospital discharge).** Published reports suggest that the incidence of POAF within 30 days after cardiac surgery is low, in the range of 2-5% [19-22]. It should also be noted that most, if not all, of these studies assessed patients' rhythm status with a single 12-lead ECG on post-operative day #30. However, trying to detect for POAF/AFL in the sub-acute phase with a single ECG has poor sensitivity when compared to continuous cardiac rhythm monitoring. Accordingly, there is likely an element of ***detection bias*** in published reports which described the incidence of POAF in the post-cardiac surgical setting. The intensity of AF detection was much greater during hospitalization (the immediate post-operative phase) when compared to the post-discharge period. This may contribute to the marked decline in the reported incidence of POAF for cardiac

surgical patients as reported by various studies.

Evidence in favor of this argument comes from an observational study by Funk et al. in which cardiac surgical patients wore 14-day event recorders after hospital discharge [23]. To our knowledge, this was the only study which evaluated the rhythm status of cardiac surgical patients after hospital discharge with a more sensitive modality than a 12-lead ECG. Patients transmitted their cardiac rhythm status on a daily and symptom-based basis. With this enhanced cardiac rhythm monitoring strategy, the incidence of POAF was found to be 14% within 2 weeks after hospital discharge [23]. In this study, it should be noted that the rhythm status of participants was not continuously recorded but rather was only triggered by the patient in this study. Therefore, it was quite possible and likely that asymptomatic AF episodes would not have been detected by this study's monitoring approach. While it is generally accepted that new-onset POAF is a transient phenomenon which declines to negligible levels by 3 months, the true incidence of POAF in the sub-acute phase and beyond is not clearly defined. It may be higher than we traditionally assume, particularly during the first month after hospital discharge.

1.4 Many cardiac surgical patients are at high risk for AF-related stroke

The great majority of cardiac surgical patients have concomitant cardiovascular co-morbidities, including hypertension, diabetes, older age, kidney disease, peripheral artery disease, and cerebrovascular disease. Applying validated stroke risk prediction tools such as the CHA_2DS_2-VASC scoring schema, a substantial portion of cardiac surgical patients were found to be at high risk for stroke. In a study by Gialdini et al. which included 11,837 cardiac surgical patients with new onset POAF, 86.0% and 38.5% of them had a CHA_2DS_2-VASC score of ≥2 and ≥4, respectively [4]. Given that a sizeable proportion of cardiac surgical patients are at high risk for stroke when AF is diagnosed in the post-operative state, this highlights the need to develop strategies to effectively detect and diagnose AF in this population.

1.5 Oral anticoagulation may be underused in cardiac surgical patients with POAF

There is data to suggest that oral anticoagulation (OAC) may be under-prescribed for cardiac surgical patients with POAF. Amongst 445 post-CABG patients with POAF enrolled in the prospective Perioperative Cardiovascular Surgical Care (CAPS-Care) registry, a sub-study of the Society of Thoracic Surgery database, only 173 patients (39%) were discharged on warfarin in spite of the fact that 80% of them had a $CHADS_2$ score of ≥2 [24]. Factors such as concern over post-operative bleeding, absence of high-quality guideline recommendations on OAC use in the POAF setting, and the prevailing belief that POAF is a transient phenomenon likely contribute to OAC non-use in this otherwise high-risk population. If, however, we can demonstrate the POAF continues to be an ongoing, frequent, and recurrent issue for these patients soon after hospital discharge, this will support the concept of early OAC initiation after surgery if POAF is diagnosed.

1.6 Extended cardiac rhythm monitoring improves the rate of AF detection

There is considerable interest in using extended cardiac rhythm monitoring strategies to detect AF in selected patient subsets at high-risk for stroke. The EMBRACE trial randomized patients with cryptogenic stroke or TIA to extended cardiac rhythm monitoring with a wearable 30-day monitor or to usual care which consisted of a 24-hour Holter monitor [25]. The extended monitoring strategy resulted in a 5-fold increase in the detection of AF or AFL for ≥30 seconds

when compared to usual care. In the CRYSTAL-AF trial, patients with cryptogenic stroke were randomized to intensive cardiac rhythm monitoring with an implantable loop recorder (ILR) or to usual care. By 6 months, the rate of AF detection was 6 times higher in the ILR group when compared to usual care [26]. Using ILR technology, two trials (ASSERT-II, REVEAL-AF) are evaluating the rate of AF detection in subjects who are at risk for AF-related stroke but without a prior history of documented AF.

In the post-cardiac surgical population, we posit that the first month after cardiac surgery continues to represent a vulnerable period for development of new-onset POAF, and that a strategy of enhanced cardiac rhythm monitoring may be particularly important in detecting AF during this sub-acute period, particularly in those patients at high risk for stroke. Based on published studies in other patient subsets [25,27], an extended rhythm monitoring strategy may improve the rate of AF detection in the target population of this study.

This concept is unexplored in the cardiac surgical population and represents an important unanswered, actionable question. If we demonstrate a significant increase in detectable and actionable AF during this period, it will provide important information regarding the rhythm monitoring practices and anticoagulation needs for stroke prevention in this high-risk patient cohort.

1.7 A novel 30-day cardiac rhythm monitoring system to detect AF

Presently, extended cardiac rhythm monitoring can be achieved in three ways. First, the traditional approach involves the use of multiple 24h, 48h, 7-day or 14-day Holter monitors. This approach is limited due to poor patient compliance, in part related to skin irritation caused by adhesive skin contact electrodes. The second option is with an external event loop recorder, but most available devices are cumbersome for patients to wear and can only store a limited number and duration of episodes which may necessitate repeat clinic visits to download the recorded episodes. There is also inherent delay from the time of AF detection to diagnosis with this monitoring system since the episodes need to be transmitted, interpreted, and reported by separate, specialized personnel. As such, this system is limited in its user-friendliness and efficiency of data communication. A third option is insertion of an implantable loop recorder (ILR). However, this is an invasive procedure involving a much more expensive device. The indwelling nature of the ILR diminishes its attractiveness for patients who only require extended cardiac rhythm monitoring for a relatively short period of time, such as over several weeks to months.

This trial will afford the opportunity to evaluate a novel diagnostic tool for AF detection over a short-term period. The Medtronic SEEQ™ mobile cardiac telemetry system is a novel, wearable technology which can provide up to 30 days of continuous rhythm monitoring **(*appendix A-D*)**. The monitoring device is an adhesive, water-resistant patch which is applied over the patient's anterior chest wall. An attractive feature of this technology is that detected episodes will be directly transmitted from the patient to a centralized data centre where they can be quickly analyzed by trained personnel. This can potentially reduce the turnaround time from AF detection to delivery of vital clinical information to treating physicians.

1.8 Systematic reviews performed to date:

No systematic review has been performed examining the specific research question of our proposed trial (enhanced cardiac monitoring vs. usual care in the rate of POAF/AFL detection after cardiac surgery). We performed a series of literature reviews in PUBMED and www.clinicaltrials.gov to address a number of study issues. The PUBMED search strategy was described in ***appendix E*** and is current as of May 13 2015.

(1) Novelty of the present research question: We performed a PUBMED search using the following keywords ("MeSH" and "TIAB"): atrial fibrillation, cardiac surgery, randomized controlled trial, ambulatory electrocardiography. We did not identify any published RCT which examined the specific research question of our proposed trial (enhanced cardiac monitoring with a continuous rhythm monitoring modality vs. usual care in the rate of POAF/AFL detection after cardiac surgery). In the www.clinicaltrials.gov database, we did not identify any ongoing, planned, completed, or terminated RCT which addressed our specific research question. To our knowledge, our research question has not been addressed by any ongoing or completed RCT, suggesting that our proposed question is novel and merits justification for further pursuit.

(2) Ongoing / completed studies (non-RCTs) examining the role of enhanced cardiac rhythm monitoring in the detection of POAF/AFL after cardiac surgery: We employed a similar approach as above to search for ongoing or completed non-randomized studies evaluating the role of continuous cardiac rhythm monitoring to detect for POAF/AFL in cardiac surgical subjects. We identified 4 studies and they are described in appendix E. The only completed study (Funk M. et al.) employed an older, relatively cumbersome monitoring system [23]. Most importantly, rhythm monitoring was triggered by self-activation and therefore this system could not monitor cardiac rhythm on a continuous basis. The other 3 ongoing studies are small (50-100 subjects); 2 of them employed ILR technology for continuous rhythm monitoring and thus required a separate invasive procedure for placement of the device. The study by Lowres et al. utilizes an i-Phone application to evaluate for AF on a daily basis; hence subjects' rhythm status is not monitored on a continuous basis [30].

1.9 The need for a randomized controlled trial

Recent studies have shown that enhanced cardiac rhythm monitoring can substantially increase the rate of AF detection in patients with cryptogenic stroke, when compared to usual care which typically consisted of a single 24-hour Holter [26,27]. Since POAF is so common after cardiac surgery and given that many of these patients will be at high risk for stroke (and hence potentially eligible for treatment with OAC if AF is detected), a trial which similarly compares the diagnostic yield of enhanced AF detection to usual care in the sub-acute, post-operative period (ie: within the first month after surgery) will introduce new knowledge to a previously unexplored aspect in the peri-operative management of cardiac surgery patients. If enhanced cardiac rhythm monitoring can indeed detect a high rate of POAF which normally will not have been diagnosed with usual care, this can potentially change clinical practice in regards to the monitoring practices and anticoagulation choices for stroke prevention in these high-risk patients.

Presently, there is little data to inform clinicians on the optimal monitoring strategy for POAF in post-cardiac surgical patients, particularly after hospital discharge. This is reflected by the

paucity of recommendations in this area by AF guidelines from major cardiovascular societies such as the Canadian Cardiovascular Society, American Heart Association, and the European Society of Cardiology [31-33].

Accordingly, a RCT will introduce high-quality evidence to an important topic regarding the optimal cardiac rhythm monitoring strategy for post-cardiac surgical patients. Successful completion of a RCT may potentially change clinical practice in this arena.

2. Study Objectives

The aim of SEARCH-AF is to evaluate an enhanced monitoring strategy for detecting atrial fibrillation or flutter (AF/AFL) during the sub-acute, post-discharge period of cardiac surgical subjects who underwent CABG and/or valve surgery. The primary purpose of this study is to compare this enhanced monitoring strategy to usual care and determine if it results in greater rates of AF/AFL detection for high-risk subjects during the sub-acute, post-operative phase after cardiac surgery. The ultimate goal of this research is to potentially improve our ability to identify post-operative AF/AFL in this population, which in turn may facilitate delivery of appropriate stroke prevention therapies.

2.1 Primary Objective:

1) To test whether enhanced cardiac rhythm monitoring with an adhesive, continuous monitoring device results in higher rates of AF/AFL detection during the subacute, post-discharge period of cardiac surgical subjects who are at risk of developing post-operative atrial arrhythmias.

2.2 Secondary Objectives:

1) To assess the incidence of AF/AFL of cardiac surgical subjects during: (i) the subacute, post-discharge period and (ii) late period (6 months after surgery).
2) To assess the proportion of subjects with continuous AF and/or AFL lasting ≥ 24 hours during the subacute, post-discharge period of subjects after cardiac surgery.
3) To assess subjects' adherence and incidence of adverse events from use of the Medtronic SEEQ™ system.
4) To assess the proportion of subjects who are prescribed with oral anticoagulation at 45 days after discharge, at 6±1 months, and at 9±1 months from cardiac surgery.
5) To assess the proportion of subjects who experienced major bleeding events during the 45 day-period after discharge from cardiac surgery.
6) To evaluate factors associated with development of POAF/AFL during the subacute, post-discharge period of subjects after cardiac surgery.

2.3 Study Hypothesis

Among post-cardiac surgical subjects without a previous history of AF/AFL but with risk factors for developing POAF/AFL, a strategy of enhanced cardiac rhythm monitoring (using an adhesive continuous monitoring system) results in a 3-fold or a 7% absolute increase in the rate of AF/AFL detection (defined as a cumulative AF/AFL burden ≥6 minutes or documentation of AF/AFL by a 12-lead ECG) when compared to usual care within 30 days after discharge from surgery.

3. Ethical considerations

3.1 Good Clinical Practice (GCP)

This study will be conducted in accordance with Good Clinical Practice (GCP), as defined by the International Conference on Harmonisation (ICH) and in accordance with the ethical principles underlying European Union Directive 2001/20/EC and the United States Code of Federal Regulations, Title 21, Part 50 (21CFR50).

The study will be conducted in compliance with the protocol. The protocol, any amendments, and the subject informed consent will receive Institutional Review Board/Independent Ethics Committee (IRB/IEC) approval/favorable opinion before initiation of the study.

All potential serious breaches must be reported to the study sponsor (St. Michael's Hospital, Toronto, Canada) immediately. A serious breach is a breach of the conditions and principles of GCP in connection with the study or the protocol, which is likely to affect, to a significant degree, the safety or physical or mental integrity of the subjects of the study or the scientific value of the study.

Study personnel involved in conducting this study will be qualified by education, training, and experience to perform their respective tasks.

This study will not use the services of study personnel where sanctions have been invoked or where there has been scientific misconduct or fraud (eg, loss of medical licensure; debarment).

3.2 Research Ethics Board/Institutional Review Board

Before study initiation, the investigator must obtain written and dated approval from the REB/IRB for the following: protocol, consent form, subject recruitment materials/process (e.g. advertisements), and any other written information which will be provided to subjects.

The investigator or sponsor should also provide the REB/IRB with device information and a copy of any guidance documents or information about the device to be provided to subjects, and any updates.

The investigator should provide the REB/IRB with reports, updates, and other information (e.g. expedited safety reports, amendments, and administrative letters) according to regulatory requirements or institution procedures.

The principal site investigator will be responsible for obtaining REB/IRB approval and annual renewal throughout the duration of the study.

3.3 Informed Consent Process

Investigators must ensure that subjects or, in those situations where consent cannot be given by subjects, their legally acceptable representative are clearly and fully informed about the purpose, potential risks, and other critical issues regarding clinical studies in which they volunteer to participate.

Investigators must:

1) Provide a copy of the consent form and written information about the study in the language in which the subject is most proficient prior to clinical study participation. The language must be non-technical and easily understood.

2) Allow time necessary for subject or subject's legally acceptable representative to inquire about the details of the study.

3) Obtain an informed consent signed and personally dated by the subject or the subject's legally acceptable representative and by the person who conducted the informed consent discussion.

4) Obtain the REB/IRB's written approval of the written informed consent form and any other information to be provided to the subjects, prior to the beginning of the study, and after any revisions are completed for new information.

5) If informed consent is initially given by a subject's legally acceptable representative or legal guardian, and the subject subsequently becomes capable of making and communicating their informed consent during the study, then consent must additionally be obtained from the subject.

6) Revise the informed consent whenever important new information becomes available that is relevant to the subject's consent. The investigator, or a person designated by the investigator, should fully inform the subject or the subject's legally acceptable representative or legal guardian, of all pertinent aspects of the study and of any new information relevant to the subject's willingness to continue participation in the study. This communication should be documented.

Participants may withdraw consent at any time during the course of the trial. The informed consent form will be signed and dated by the participant and the person who conducted the informed consent discussion. The original signed informed consent form will be retained in the participant's study files and a copy of the signed form will be provided to the participant.

3.4 Participant Confidentiality

All subject related information including Case Report Forms, evaluation forms, reports, etc. will be kept strictly confidential. All records will be kept in a secure, locked location and only research staff will have access to the records. Subjects will be identified only by means of a coded number specific to each subject. All computerized databases will identify subjects by numeric codes only, and will be password protected.

4. Eligibility Criteria

4.1 Inclusion Criteria

1) Male or female age ≥18 years.
2) Isolated CABG or valve replacement/repair +/- CABG performed at the index surgical procedure.
3) At elevated risk of stroke and for having POAF/AFL, defined as having a CHA_2DS_2-VASC score of ≥4 *or* ≥2 with at least 1 of the following risk factors for developing POAF/AFL:
   (i) Chronic obstructive pulmonary disease.

(ii) Sleep apnea.
(iii) Impaired renal function (defined as creatinine clearance <60 ml/min/1.73m^2).
(iv) Echocardiographic evidence of at least mild left atrial enlargement (defined as ≥41 mm on M-mode, ≥59 ml or ≥29 mL/m^2 on biplane volume assessment [34] from an echocardiogram performed within 12 months of study enrollment).
(i) Elevated body mass index (defined as BMI ≥30).
(ii) Combined surgery (CABG + valve repair/replacement).

4) Able to provide written informed consent.

4.2 Exclusion Criteria

1) Atrial fibrillation or flutter at the time of randomization.
2) Known previous history of AF/AFL, diagnosed pre-operatively (note: documentation of a history of AF/AFL without accompanying rhythm proof will suffice).
3) Documentation of continuous AF/AFL for ≥24 hours during the in-hospital stay for the index cardiac surgery.
4) Subjects who, at the discretion of the treating cardiac surgery team, would be treated and discharged with oral anticoagulation due to POAF/AFL.
5) Mechanical valve replacement.
6) Current or anticipated treatment with oral anticoagulation for indications other than AF/AFL.
7) Hospitalization for ≥10 days (for the index cardiac surgery, with day #0 being the day of surgery).
8) Planned discharge from hospital with a type IC or III anti-arrhythmic drug.
9) Having received >5 grams of IV and/or oral amiodarone during hospitalization for the index cardiac surgical procedure.
10) Women of childbearing potential (WOCBP) (please refer to section 4.3 for definition).
11) History of percutaneous or surgical left atrial ablation for AF.
12) Presence of a cardiac implantable electronic device with a functioning atrial lead (pacemaker, implantable cardioverter defibrillator, or cardiac resynchronization device).
13) Presence of an implantable loop recorder.
14) History of left atrial appendage ligation, removal, or occlusion.
15) Subjects with known allergies or hypersensitivities to adhesives or hydrogel.
16) Inability to provide written informed consent.
17) Current or anticipated participation in another randomized controlled trial in which the interventional drug or device is known to affect the incidence of the primary or secondary outcomes of this study.

4.3 Women of childbearing potential

Women of childbearing potential (WOCBP) are excluded from the SEARCH-AF trial. A WOCBP is defined as any female who has experienced menarche and who has not undergone surgical sterilization (hysterectomy or bilateral oophorectomy) and is not postmenopausal. Menopause is defined as 12 months of amenorrhea in a woman over age 45 years in the absence of other biological or physiological causes. In addition, females under the age of 55 years must have a serum follicle stimulating hormone, (FSH) level > 40 mIU/mL to confirm menopause.

*Females treated with hormone replacement therapy, (HRT) are likely to have artificially suppressed FSH levels and may require a washout period in order to obtain a physiologic FSH level. The duration of the washout period is a function of the type of HRT used. The duration of the washout period below are suggested guidelines and the investigators should use their judgement in checking serum FSH levels. If the serum FSH level is >40 mIU/ml at any time during the washout period, the woman can be considered postmenopausal:

- 1 week minimum for vaginal hormonal products (rings, creams, gels).
- 4 week minimum for transdermal products.
- 8 week minimum for oral products.

Other parenteral products may require washout periods as long as 6 months.

4.4 Discontinuation of Subjects from Trial Participation

Subjects must discontinue use of the investigational device for any of the following reasons:

- Withdrawal of informed consent (subject's decision to withdraw for any reason).
- Any clinical adverse event, laboratory abnormality, or intercurrent illness which, in the opinion of the investigator, indicates that continued use of the device is not in the best interest of the subject.
- Loss of ability to freely provide consent through imprisonment or involuntary incarceration for treatment of either a psychiatric or physical (eg, infectious disease) illness.

All subjects who discontinue use of the device for any reason will be asked to continue to be followed until the final study visit, if they provide consent to do so.. If a subject declines to continue to participate in the study the reason for withdrawal must be documented appropriately.

All subjects are free to withdraw from participation in this study at any time, for any reason, specified or unspecified, and without penalty or loss of benefits to which the subject is otherwise entitled. Study patients will be informed of the possibility to withdraw consent without giving any reason. Subjects may be withdrawn for specific reasons during the study, which include: ineligibility, non-compliance or for administrative reasons (including study closure).

Before a subject is declared lost to follow-up, all efforts should have been made to contact the participant for a final assessment.

5. Study Design

5.1 Study Description

SEARCH-AF is a multicenter, parallel group (2-arm), unblinded randomized controlled trial (RCT). It will compare a strategy of enhanced cardiac rhythm monitoring to usual care in post-cardiac surgery subjects during sub-acute, post-operative period. Half of the study population will be randomly allocated to a 30-day adhesive cardiac event monitoring system (intervention group) and the other half will be randomly allocated to usual care (control group) in which no continuous cardiac rhythm monitoring will be mandated. The primary endpoint will be a cumulative atrial fibrillation/flutter (AF/AFL) burden of ≥6 minutes or documentation of

AF/AFL with a 12-lead electrocardiogram (ECG). In addition, all subjects will also receive 14 days of continuous cardiac rhythm monitoring at 6±1 months after surgery. Clinical endpoints will be adjudicated by an independent Clinical Events Committee.

5.2 Executive Committee

An academic Executive Committee, led by co-chairs Dr. Andrew Ha, Dr. Atul Verma, Dr. C. David Mazer, Dr. Bobby Yanagawa, and Dr. Subodh Verma participated in protocol development. This group will provide ongoing scientific and operational oversight to the study.

The Executive Committee will provide suggestions for potential investigators and site coordinators, monitor progress of study enrollment, make recommendations about the conduct of the study, and oversee the presentation and publication of the trial results. The Executive Committee will include clinical experts from specialties involved in management of cardiac surgical subjects with AF (cardiology, cardiac electrophysiology, cardiac surgery, cardiac anesthesiology) and who are experienced in clinical trial methodologies.

5.3 Clinical Events Committee (CEC)

The Clinical Events Committee, composed of experts in the relevant fields, will review reported study clinical outcomes (arrhythmia, MACE events, and bleeding) to provide consistency and validity in the assessment of these outcomes. They will be blinded to treatment assignment when adjudicating such outcomes. In addition, this committee will be responsible for over-reading all rhythm strips, ECGs, full-disclosure Holter results, and full disclosure SEEQ™ recordings (if necessary) to ascertain that AF/AFL was correctly diagnosed. A chairperson of the CEC will be nominated.

5.4 Baseline Visit

The baseline visit will occur at a time during hospitalization when the subject is clinically stable after his/her index cardiac surgery, according to the treating cardiac surgeon. During this visit, the following information will be collected:

Clinical information: demographics, medical history (including hypertension, diabetes, heart failure or LV dysfunction, previous history of stroke, TIA, or systemic embolism, renal disease, sleep apnea, peripheral vascular disease, COPD, alcohol use, smoking history), CHA_2DS_2-VASC score, HAS-BLED score, concomitant medications, and details pertaining to cardiac surgery.

Physical examination information: Height, weight, blood pressure, pulse rate.

Laboratory or imaging testing: Pre-operative and post-operative ECG, echocardiography (performed within 12 months from enrollment), complete blood count, creatinine, electrolytes, INR.

5.5 Randomization

After study eligibility has been confirmed and the baseline visit assessment is completed, subjects will be randomly allocated in a 1:1 ratio to the intervention arm (30 days of cardiac rhythm monitoring with an adhesive cardiac telemetry system, starting on the day of randomization) or to the control group (usual care). Randomization lists will be generated by

computer and employ random permuted blocks. Randomization will be stratified by centre and according to the type of cardiac surgery performed: (i) isolated CABG or (ii) valve replacement or repair +/- CABG. Randomization can occur at any time between the third post-operative day and discharge.

Stratification by surgery type is felt to be important for this trial for the following reasons. First, in the published literature, subjects who undergo valve replacement or repair were more likely to develop POAF with reported incidences of up to 60% during hospitalization [8,10,17]. These rates are higher than those who undergo isolated CABG, in which the highest rates are typically in the range of ≈40% [7-19]. Second, the case volume ratio of isolated CABG to valve surgery (±CABG) is in the range of 4:1. Therefore, stratification will provide balance to the proportion of subjects with isolated CABG vs. valve surgery ± CABG in both study arms.

If a subject is randomized to the intervention group, the device will be fitted onto the subject on the same day of randomization. If the subject is randomized to usual care, no formal continuous cardiac rhythm monitoring will be mandated. However, subjects in the usual care group may undergo rhythm assessment (e.g. ECG, Holter monitoring, event monitoring) if there is a clinical indication to do so, as per the discretion of their treating physicians.

5.6 Proposed Study Intervention

Subjects will be randomly allocated to one the following two arms: (i) Continuous cardiac rhythm monitoring for 30 days after randomization (intervention group) or (ii) Usual care (control group). In addition, all subjects will also receive 14 days of continuous cardiac rhythm monitoring at 6±1 months after surgery.

5.6.1 30-day continuous cardiac rhythm monitor early after surgery (Intervention group)

On the day of randomization, subjects in this group will receive 30 days of continuous cardiac rhythm monitoring with an adhesive monitor. The device that will be used is the Medtronic SEEQ™ mobile cardiac telemetry system *(appendix A-D).*

Wearable sensor

The SEEQ™ system consists of a wearable sensor which is a single-lead, low-profile, peel-and-stick device applied over the anterior left chest wall of the subject. It is water-resistant, which is a major difference than other forms of prolonged cardiac rhythm monitoring modalities such as the 14-day Holter monitor. The wearable patch is designed for one-time use only and cannot be re-applied if removed. Each sensor provides up to 7.5 days of monitoring. Therefore, the sensor will be applied on 4 separate occasions if 30 days of rhythm monitoring are required.

Data transmitter

The data transmitter is required to facilitate detected transmission of the detected arrhythmia episodes via a cellular network to a central data processing centre from Medtronic. Access to the cellular network is free of charge for the research participants and internet access is not required. The subject needs to be within 30 feet from the transmitter in order for the detected episodes to be sent wirelessly. In addition, the data transmitter is battery-operated and requires daily charging. The data transmitter also has a self-activated button which allows for transmission of subject-activated episodes to provide rhythm-symptom correlation.

Data reporting
All auto-detected and subject-triggered episodes which are successfully transmitted will be sent to a central Medtronic monitoring centre. Selection of rhythm strips for inclusion in the summary report is governed by a pre-specified set of rhythm/rate-based parameters *(appendix C)*. The report includes a number of arrhythmic diagnoses, including episodes of new-onset AF or AFL irrespective of the ventricular rate. A summary report with accompanying rhythm strips will be sent to the treating physician and the trial coordination centre on a weekly basis.

The transmitted data will be anonymized as each research participant in SEARCH-AF will be identified by a unique study code which does not contain any personal identifying information. If the subject is re-hospitalized or assessed in an ambulatory clinic during the intervention period (defined as the first 30 days after study randomization), all efforts will be made to obtain 12-lead ECGs or cardiac telemetry strips that were performed during those clinical visits.

Currently, the Medtronic SEEQ™ mobile cardiac telemetry system is not approved for clinical use in Canada. Approval for Investigational Testing (Medical Devices) from Health Canada will be obtained. If this device is approved for clinical use in Canada during the course of the trial, at which point sites will notify their REBs of this change.

Patient Education
Given that a new adhesive sensor needs to be applied every 7.5 days, each subject in the intervention group will need to remove and re-apply a new sensor on 3 additional occasions. The first application will be done during hospitalization, under the supervision of the research coordinator. During the first sensor application, the subject will be educated on the appropriate use of this device, including the following:

1) Proper placement of the adhesive sensor.
2) Use and maintenance of the data transmitter.
3) Self-activation of the sensor for symptomatic episodes.

For subjects randomized to the intervention group, the research coordinator will contact them on a weekly basis by telephone to reinforce education of the proper use of the device (if necessary). In addition, adverse outcomes related to the use of the study interventional device will be assessed and an in-person clinic visit may be arranged if required.

Studies of the SEEQ™ mobile cardiac telemetry system had shown high levels of patient compliance and satisfaction with this device. In these studies, 98% of patients were able to properly apply the SEEQ™ adhesive sensor, patient compliance throughout the prescribed monitoring period was reported to be 90%, and 85% of patients were satisfied with the SEEQ™ mobile cardiac telemetry system. (http://www.medtronicdiagnostics.com/us/cardiac-monitors/seeq-mct-system/evidence/index.htm, accessed July 31 2015)

5.6.2 Usual Care
Subjects randomized to the usual care arm will be discharged from hospital without protocol-mandated continuous cardiac rhythm monitoring. Within the first 30 days after randomization, no protocol-mandated continuous cardiac rhythm assessment is planned. This is consistent with the practice of the participating surgical sites in SEARCH-AF and the overall Canadian/global cardiac surgical community at large. Presently, there are no clinical guidelines or

recommendations to inform clinicians on how to monitor for POAF/AFL in the cardiac surgical population during the sub-acute, post-discharge phase, particularly within the first month after discharge. The 2010 Canadian Cardiovascular Society AF guidelines recommend that patients be reviewed at 6-12 weeks after surgery if they are treated with medications for their POAF/AFL [31]. However, the specific cardiac monitoring strategy is not described. The most recent iterations of the ACC/AHA/HRS or ESC AF guidelines do not contain specific recommendations on the detection or follow-up care of cardiac surgical patients with POAF/AFL after discharge [32,33].

However, subjects in the control group may undergo rhythm monitoring during the study period if their treating physicians deem that there is a clinical indication to so do. Typical rhythm monitoring modalities such as a 12-lead ECG, Holter monitoring, or event monitoring may be prescribed on clinical grounds. The test reports will be obtained along with full disclosure of rhythm strips. These will be independently overread by the CEC. If the subject is re-hospitalized or has an unscheduled medical visit during the first 30 days after randomization (the study period during which the primary endpoint will be evaluated), all cardiac rhythm tests which are performed will be obtained and overread.

5.6.3 Additional 14-day cardiac rhythm monitoring at 6±1 months

At the 6±1 month follow-up visit, all subjects (in the intervention group and the usual care group) will undergo 14 days of continuous cardiac rhythm monitoring with the SEEQ™ mobile cardiac telemetry system. This will involve application of 2 SEEQ™ monitors over the 14-day monitoring period. The first (of two) SEEQ™ monitor will be applied onto the subject at the 6±1 month visit. At this visit, subjects will be educated on the use and maintenance of the SEEQ™ monitor. Please refer to section 5.6.1 for details on this aspect.

5.7 Methods to Protect against Potential Bias

We have identified several potential sources of bias in this trial and have proposed methods to minimize them:

Non-blinded study design: SEARCH-AF is an open-label RCT since subjects and clinicians will be aware of the treatment assignment. Atrial arrhythmias detected by the SEEQ™ system will be initially interpreted by trained technicians in Medtronic's reading centre who are not involved in any part of the design and are unaware of the study design. Adhering to standardized protocols, they are trained to interpret and report whether AF/AFL is present or absent and the duration of these episodes. We do not expect Medtronic will play any role in biasing the results away from the null by preferentially "over-calling" AF/AFL episodes for participants in this study. In addition, full disclosure of the entire monitoring period can be retrieved. The generated rhythm strips and reports will then be sent to an independent group of adjudicators consisting of cardiologists and cardiac electrophysiologists. These adjudicators will over-read the reports with the attached rhythm strips and verify if the diagnosis is indeed correct. If needed, they will be able access full disclosure rhythm strips for further review. Additional rhythm monitoring with Holter, 12-ECG, in-patient telemetry strips, and cardiac event monitors will only be done on clinical grounds (ie: if deemed necessary by the treating physicians). Patient identification information will be removed and sent to the adjudicators for review, who will be blinded to the

randomization status of the subjects. We believe these measures will minimize/mitigate potential bias due to the open-label design of SEARCH-AF.

Detection bias: It can be argued that since no continuous cardiac rhythm monitoring is mandated in the control arm, the intervention arm will be favored due to the fact that it will be exposed to a more intense cardiac rhythm monitoring strategy, consequently allowing for a greater number of POAF/AFL episodes to be detected. While this is intuitively correct, the purpose of this study is to demonstrate that enhanced monitoring can substantially improve the rate POAF/AFL detection over usual care, by at least 3-fold, within the first month after surgery. This trial will be powered to detect an absolute difference of 7% in POAF/AFL detection between the two arms (based on an assumed event rate of 3% in the control group). The magnitude of this difference (3-fold increase or 7% absolute difference) is felt to be clinically relevant by the Steering Committee of SEARCH-AF, which consists of cardiac electrophysiologists who specialize in the care of AF patients, a neurologist who specialize in AF-related stroke care, cardiac surgeons and a cardiac anesthesiologist who manage this post-operative issue on a daily basis.

Misclassification bias: All forms of ambulatory cardiac rhythm monitoring are subject to artifact (e.g. motion artifact) which may prohibit accurate rhythm diagnosis. The presence of artifact may result in either under- or over-diagnosis of AF/AFL. To minimize this potential source of bias, a standard operating procedure manual for event adjudication will be developed. All detected AF/AFL episodes will be confirmed by the CEC. If there is disagreement in the diagnosis of the detected AF/AFL episode, it will be reviewed by the chair of the adjudication committee for determination of the final rhythm diagnosis.

5.8 Proposed Duration of the Intervention Period

There will be two intervention periods. For subjects randomized to the intervention group (enhanced cardiac rhythm monitoring), they will undergo 30 days of continuous cardiac rhythm monitoring with the Medtronic SEEQ™ mobile cardiac telemetry system starting on the day of randomization. At 6±1 months after discharge from the index cardiac surgery, they will undergo another 14 days of cardiac rhythm monitoring with the SEEQ™ device.

Subjects in the usual care group (control group) will only undergo protocol-mandated cardiac rhythm monitoring at 6±1 months after discharge. At that time, they will undergo 14 days of cardiac rhythm monitoring with the SEEQ™ device.

5.9 Proposed Frequency and Duration of Follow-up

Visit #1 (45-52 days after discharge): All subjects will be assessed during follow-up at 45-52 days after discharge from their index cardiac surgery. Details of MACE and major bleeding events which occurred after hospital discharge will be recorded. As well, subjects' list of medications will be updated. Specifically, a detailed history of OAC use will be obtained, including the date of initiation, prescriber, dose, and type.

In this visit, subjects in the intervention group will return their data transmitters, have their wearable sensors removed (if still worn by the participant), and undergo a 12-lead ECG. Subjects in the control group will undergo a 12-lead ECG during this follow-up visit as well.

Visit #2 (6±1 months after surgery): Subjects will be assessed during a second follow-up visit at 6±1 months after surgery. At this visit, all subjects will receive 14 days of continuous cardiac rhythm monitoring with the SEEQ™ device. All subjects will be educated on the proper use of the device. Details of MACE and bleeding events which occurred since the first follow-up visit will be recorded. As well, subjects' list of medications will be updated. A detailed history of OAC use will be obtained, including the date of initiation, prescriber, dose, type.

Visit #3 (9±1 months after surgery): This visit will be conducted by telephone. At this visit, a history of OAC use will be obtained (type, dose, prescriber).

5.10 Study flowchart

Evaluation	*Screening/ Baseline*	*Randomization (between the 3rd post-operative day and discharge)*	*Visit #1 (45-52 days after discharge)*	*Visit #2 (6±1 months after surgery)*	*Visit #3 (telephone-based) (9±1 months after surgery)*
**Inclusion/exclusion criteria**	X				
**Medical history**	X		X	X	
**Informed consent**	X				
**Physical exam**	X		X	X	
**Blood pressure + Heart Rate**	X		X	X	
**Medication review**	X		X	X	X
**12-lead ECG**	X		X	X	
**Randomization**		X			
**Education of device application and maintenance of data transmitter**		X *(intervention group only)*		X (all subjects)	
**Application of the SEEQ™ device**		X *(intervention group only)*		X (all subjects)	
**Primary and/or secondary outcomes**			X	X	X
**SAE**	X*	X	X	X	X

**SAE collection begins after consent is signed*

5.11 Primary Outcome

The primary endpoint of this trial is documentation of AF/AFL, defined as a cumulative AF/AFL duration of ≥6 minutes or documentation of AF/AFL by a 12-lead ECG within 30 days after randomization. Clinical endpoints will be adjudicated by an independent clinical events committee.

5.12 Secondary Outcomes

1) AF/AFL lasting for ≥24 hours during 30 days after randomization and at 6±1 months after surgery.
2) Cumulative AF/AFL burden during the 14-day monitoring period (with the SEEQ™ device) at 6±1 months after surgery.
3) Proportion of subjects who are prescribed with oral anticoagulation at the following timepoints: (i) 45 days after discharge from cardiac surgery; (ii) 6±1 months after discharge from cardiac surgery; (iii) 9±1 months after discharge from cardiac surgery.
4) Number of days during which the Medtronic SEEQ™ sensor was worn by subjects.
5) Reasons for premature removal of the Medtronic SEEQ™ sensor by subjects.
6) Incidence of adverse events related to use of the Medtronic SEEQ™ device.
7) Performance of non-protocol mandated Holter monitoring and/or event recorders during the 30-day period after randomization (for subjects in both groups).
8) Major adverse cardiac outcomes occurring within 45 days after hospital discharge, including the following: all-cause death, myocardial infarction, ischemic stroke, non-CNS systemic embolism (please refer to appendix F for endpoint definitions).
9) Proportion of subjects who experienced major bleeding events (please refer to appendix G for definitions of bleeding outcomes) within 45 days after discharge from cardiac surgery.

5.13 Prescription of Oral Anticoagulation

It is possible that greater rates of AF/AFL detection in the early post-operative period may lead to increased prescription of oral anticoagulation by the subjects' treating physicians. The rates of OAC use between the intervention and control groups at 45 days, 6±1 months, and 9±1 months after discharge from cardiac surgery will be recorded as secondary outcomes. In addition, the type (and dosing if applicable) of OAC used will be collected. The choice of OAC that is prescribed will be at the discretion of the subjects' treating physicians and is not mandated by protocol.

In SEARCH-AF, the decision to initiate oral anticoagulation is not mandated by protocol but rather is left at the discretion of the subjects' treating physicians. The decision to treat a subject with OAC for AF-related stroke prevention after cardiac surgery is governed by a number factors including: (i) the subject's risk of stroke, (ii) the subject's risk of bleeding in the early post-operative phase, (iii) the subject's AF burden, (iv) the subject's treatment preferences. The relative weights of these factors will vary from subject to subject. Therefore, the decision to initiate OAC in this patient population is individualized.

At the current moment, there is no high-quality data in inform clinicians on the specific AF burden upon which OAC use will be beneficial in the post-cardiac surgical population. In the 2010 CCS AF guidelines, there was a conditional recommendation of low-quality evidence

suggesting the use of OAC for post-cardiac surgical patients with continuous AF of >72 hours. Specifically, it emphasized an "individualized assessment of the risks of a thromboembolic event and the risk of postoperative bleeding" [29]. In the 2010 ESC AF guidelines, initiation of OAC is recommended for post-cardiac surgical patients with AF duration >48 hours in the absence of contraindications to OAC use (Class IIa recommendation, level of evidence "A") [32]. In the 2014 AHA/ACC/HRS AF practice guidelines, antithrombotic treatment for post-cardiac surgical patients who developed AF was considered to be "reasonable…as advised for non-surgical patients" (Class IIa recommendation, level of evidence "B") [33]. In the American guidelines, there was no mention of a specific AF duration threshold which will trigger prescription of OAC therapy. This highlights the existing variability in clinical practice as it pertains to the use of OAC for post-cardiac surgical patients who develop AF.

Each participating site in SEARCH-AF will be co-led by a cardiologist/cardiac electrophysiologist and a cardiac surgeon. We will encourage dialogue amongst the subject's treating physician, the subject's cardiac surgeon, and the local site PIs in the decision-making process of OAC use for subjects in SEARCH-AF. The local site PIs will provide consultative advice on whether a given subject should be considered for OAC use, after accounting for his/her risk of stroke, risk of bleeding, and AF burden. Ultimately, the final decision to initiate OAC for subjects in this trial will be at the discretion of their treating physicians.

5.14 Other Considerations

Loss to follow-up: Given that the primary endpoint will be assessed at a short time course after randomization (30 days), we anticipate the rate of loss to follow-up to be low (<1%). In addition, all efforts will be made to ensure that subjects are assessed at follow-up at 45-52 days after discharge from their index cardiac surgery.

Non-compliance: It is possible that subjects may stop wearing the SEEQ™ sensor given that a new sensor needs to be applied every week for a total of 4 weeks (to allow for 30 days of monitoring). To minimize this, research coordinators will contact (telephone or email) subjects on a weekly basis during the study period to ensure compliance with enhanced cardiac rhythm monitoring. The monitoring centre will also alert the research team if they do not receive transmitted data for a period of more than 48 hours. Previous studies had reported high compliance rates of wearing the SEEQ™ monitor for the prescribed monitoring period [35,36].

Crossover: Subjects randomized to the control group will be unlikely to crossover to the intervention group, since the costs of the adhesive system employed in this trial are not covered by government health plans and this device is not routinely used in clinical practice, and the device is not currently approved for use in Canada. On the other hand, it is possible for subjects in the intervention group to be crossed over to usual care if, for some reason, they no longer wear the adhesive monitor. However, we anticipate the rate of non-compliance to be low. If this unlikely situation is to occur, we do not anticipate that the results will significantly bias away from the null because (i) the anticipated occurrence rate of crossover is low (<1%), (ii) subjects will be crossed over to usual care in which the intensity of rhythm monitoring is less; (iii) analyses will be performed with the intent-to-treat principle.

6. Adverse events

6.1 Device Problem Reporting

Subject to section 59 of the Medical Device Regulations in Canada, any incident that comes to the attention of the investigator which meets the following conditions must be reported to the study sponsor within 24 hours of becoming aware of the incident:

(1) Incident is related to a failure of the device or a deterioration in its effectiveness, or any inadequacy in its labelling or in the directions for use;

AND

(2) Has led to the death or a serious deterioration in the state of health of a patient, user or other person, or could do so were it to recur.

In such a situation, the investigator must complete the SEARCH-AF Incident Report and submit the report to the coordinating centre within 1 business day of becoming aware of the incident. The following information will be required: date of the incident, details of the incident, course of action taken, and other relevant details.

Follow-up reports must be provided to the coordinating centre as any new information becomes available. The coordinating centre or device manufacturer will report these incidents to the Medical Devices Bureau of Health Canada within the timelines specified in section 60 of the regulations.

6.2 Serious adverse event collecting and reporting

Please refer to appendix H for details pertaining to serious adverse event (SAE) collection and reporting.

7. Study duration

7.1 Expected study duration of subject participation

The anticipated duration of subject participation will be 9 months. The first follow-up visit will occur at 45-52 days after discharge from cardiac surgery and the second follow-up visit will occur at 6±1 months after discharge from cardiac surgery. A final telephone-based follow-up will occur at 9±1 months after discharge from cardiac surgery.

7.2 Expected total study duration

We planned for a recruitment period of 36 months. The last subject enrolled will be followed up at 9 months after surgery, extending the total study duration to 45 months.

8. Concomitant Medications/Natural Remedies/Foods

There are no restrictions placed on medication use in this study. Prescription medications will be recorded on the concomitant medication form in case report forms at baseline and follow-up visits.

9. Prohibited Medications and Procedures

There are no restrictions placed on medication use or procedures mandated by this study. Subjects will continue to receive all usual medications, rehabilitation, procedures and interventions as prescribed or recommended by his/her health care providers.

10. Study Evaluations/Procedures

10.1 Laboratory Evaluations and Specimen Collection

Not applicable.

10.2 Questionnaires

Not applicable.

11. Trial Management

11.1 Study coordinating centre

The day-to-day management of the trial will be supported by the Applied Health Research Center (AHRC). AHRC is a clinical research methods and services group at the Li Ka Shing Knowledge Institute of St. Michael's Hospital (Toronto, Canada). The AHRC has established itself as a comprehensive Academic Research Organization (ARO) affiliated with the University of Toronto. The AHRC has experience in managing more than 50 multi-site, national and international clinical trials and observational studies, and has grown to be one of the largest academic research organizations in Canada. The AHRC will work closely with the study sponsor and Steering Committee(s) to manage this clinical trial, including protocol finalization, site section and management, training personnel, collecting and managing trial documents and assisting with ethics submissions. AHRC will coordinate data management activities and will be responsible for developing electronic case report forms, performing data validation activities, providing data outputs and performing statistical analyses. AHRC employs industry-leading web-based secure database technology which incorporates advanced data validation and reporting tools, and allow for faster access to trial data than traditional paper-based data collection tools. The electronic database has built-in web-based study randomization tools and can link with other data systems (e.g. laboratory information systems) to facilitate automatic upload of clinical data to minimize manual entry. The AHRC is using electronic data capture software to manage data for over 25 studies, including more than 15 CIHR-funded multi-centre trials. AHRC was the data coordinating and management centre for the EMBRACE trial [25].

11.2 Compliance with the Study Protocol

The study shall be conducted as described in this approved protocol. All revisions to the protocol must be discussed with, and be prepared by, the Study Sponsor and the coordinating (St. Michael's Hospital and the Applied Health Research Centre which is affiliated with St. Michael's Hospital). The investigator should not implement any deviation or change to the protocol without prior review and documented approval/favorable opinion from the REB/IRB of an amendment, except where necessary to eliminate an immediate hazard(s) to study subjects.

If a deviation or change to a protocol is implemented to eliminate an immediate hazard(s) prior to obtaining IRB approval/favorable opinion, as soon as possible the deviation or change will be submitted to:

- REB/IRB for review and approval/favorable opinion
- Study Sponsor.
- Regulatory Authority(ies), if required by local regulations

Documentation of approval signed by the chairperson or designee of the REB/IRB(s) must be sent to the study sponsor.

If an amendment substantially alters the study design or increases the potential risk to the subject: (1) the consent form must be revised and submitted to the REB/IRB(s) for review and approval/favorable opinion; (2) the revised form must be used to obtain consent from subjects currently enrolled in the study if they are affected by the amendment; and (3) the new form must be used to obtain consent from new subjects prior to enrollment.

If the revision is an administrative letter, investigators must inform their REB/IRB(s).

12. Statistical Considerations

12.1 Sample Size Calculation

In this trial, the primary endpoint is the occurrence of sustained POAF/AFL, defined as a cumulative burden of ≥6 minutes or documentation with a 12-lead ECG. Based on the existing literature, the rate of POAF/AFL in the sub-acute, post-operative phase (within 1 month after surgery) was reported to be 2-5% [20-23]. For this trial, we assume that the rate of AF/AFL detection in the usual care group (control group) to be 3% during the first 30 days after randomization. We seek to detect at least a 3-fold or a 7% absolute increase in the rate of POAF/AFL detection with enhanced cardiac rhythm monitoring. As such, we assume a POAF/AFL detection rate of 10% in the intervention group. A total of 388 subjects (n=194 in each arm) will be required to detect this difference with 80% power at a 2-sided alpha of 0.05. Assuming a 2% attrition rate (death, loss to follow-up, withdrawal of consent to participate), the final sample size will be **396** (n=198 in each arm).

The Steering Committee of SEARCH-AF has determined that the magnitude of the difference which this trial seeks to detect (a 3-fold or an absolute 7% increase in the rate of AF/AFL detection when compared to usual care) is clinically meaningful for the population of interest.

Randomized controlled trials evaluating the efficacy of enhanced AF/AFL detection in the cryptogenic stroke population had demonstrated comparable magnitudes in differences of the primary endpoint between the treatment and control groups. In EMBRACE, the rate of AF/AFL detection was 5 times greater (absolute difference in the rate of AF/AFL detection: 12.9%) in the enhanced monitoring group when compared to the control group over a study period of 90 days [26]. In CRYSTAL-AF, the rate of AF/AFL detection was 6 times greater than the control group by 6 months (absolute difference in the rate of AF/AFL detection: 7.5%) [27]. In both studies, an AF/AFL episode lasting ≥30 seconds was the primary endpoint. As such, we feel that the magnitude of difference which we seek to detect in SEARCH-AF is realistic and achievable.

12.2 Details of Statistical Analysis
Descriptive statistics will be computed. For continuous variables, mean ± standard deviation will be reported or median and interquartile range as appropriate. For categorical variables, counts ± percentages will be reported.

The primary analysis will be conducted with the intent-to-treat principle. The primary outcome will be displayed in a 2 x 2 contingency table, sorted according to randomization status (intervention/control group) and the presence/absence of the primary endpoint. A contingency table stratified by the surgery type stratification factor will also be examined for homogeneity of treatment effect. The number of AF/AFL episodes will be compared by means of a Poisson regression model (or possibly zero-inflated Poisson if indicated) and the treatment effect expressed as a rate ratio with 95% CI. The proportion of patients experiencing AF/AFL lasting for 24 hours or more will be compared with a Fisher's Exact test (because numbers of events and therefore expected cell counts are expected to be low). The treatment effect will be expressed as a difference in proportions with 95% CI. The proportions of patients prescribed with oral anticoagulants will be analyzed similarly.

Since outcomes related to the device itself do not have a comparison group, they will be analyzed using only descriptive statistics (means, proportions, etc.). All remaining secondary outcomes are binary and will be compared with a Fisher's Exact test (assuming the assumptions for the chi-square test are not met) and the treatment effects will be expressed as risk differences with 95% CIs. Given the expected very low attrition rate of 2%, any missing data resulting from attrition at that rate will be insufficient to substantially alter conclusions. Nevertheless some sensitivity analyses will be considered, especially given anticipated low event rates.

Logistic regression will be performed to obtain adjusted estimates of the treatment effect if feasible. However, given that the anticipated absolute event rates will be relatively low which will result in a small number of subjects having the primary endpoint, the choice of covariate inclusion will be carefully considered. Candidate covariates which may be included in the logistic regression model include: age, sex, heart failure/LV dysfunction, diabetes, hypertension, history of ischemic stroke, renal disease (defined as a creatinine clearance of <60 mL/min), and COPD. Selection of these covariates is based on prior knowledge and standards from published literature. Secondary outcomes will be similarly analyzed after adjusting for these variables.

In subjects in both arms, the timing of the occurrence of the first AF/AFL episode (e.g. day 1, day 2, day 15, etc.) during the 30-day period after study randomization will be documented. Survival analysis will be used to determine the time (in days) to the first recorded POAF/AFL event and displayed with a Kaplan-Meier curve. This analysis is undertaken to assess the optimal duration of continuous cardiac rhythm monitoring to capture POAF/AFL in the post-discharge period (e.g. 1, 2, 3 or 4 weeks after discharge from cardiac surgery).

If this study is able to demonstrate the pre-specified outcome difference (3-fold or 7% absolute difference in the rate of AF/AFL detection) between the two arms, then a separate economic analysis will be undertaken to evaluate the cost-effectiveness of the study intervention.

12.3 Subgroup Analysis

Isolated CABG vs. Valve replacement/repair ± CABG: A pre-specified sub-analysis for subjects who underwent isolated CABG or valve surgery ± CABG is planned. In published reports, patients who underwent valve surgery had higher rates of in-hospital POAF when compared to those who underwent isolated CABG. This analysis will provide insight on the rates of AF/AFL detection between these 2 major types of cardiac surgery in the subacute, post-discharge period. Typically, patients who undergo bioprosthetic valve replacement (the most common being aortic valve replacement) are not routinely treated with warfarin in the post-operative period unless there is another compelling reason to do so, such as the presence of AF/AFL. If high rates of AF/AFL are detected in the valve surgical subset during the sub-acute post-discharge period, this may have significant clinical implications on oral anticoagulation use for this subset of subjetcts.

13. References

1. Selnes OA, Goldsborough MA, Borowicz LM, McKhann GM. Neurobehavioural sequelae of cardiopulmonary bypass. *Lancet*. 1999;353:1601-1606.

2. Whitlock R, Healey JS, Connolly SJ, Wang J, Danter MR, Tu JV, Novick R, Fremes S, Teoh K, Khera V, Yusuf S. Predictors of early and late stroke following cardiac surgery. *CMAJ*. 2014;186:905-911.

3. Kaw R, Hernandez AV, Masood I, Gillinov AM, Saliba W, Blackstone EH.Short- and long-term mortality associated with new-onset atrial fibrillation after coronary artery bypass grafting: a systematic review and meta-analysis. *J Thorac Cardiovasc Surg*. 2011;141:1305-1312.

4. Gialdini G, Nearing K, Bhave PD, Bonuccelli U, Iadecola C, Healey JS, Kamel H. Perioperative atrial fibrillation and the long-term risk of ischemic stroke. *JAMA*. 2014;312:616-622.

5. Lee SH, Kang DR, Uhm JS, Shim J, Sung JH, Kim JY, Pak HN, Lee MH, Joung B. New-onset atrial fibrillation predicts long-term newly developed atrial fibrillation after coronary artery bypass graft. *Am Heart J*. 2014;167:593-600.

6. Villareal RP, Hariharan R, Liu BC, Kar B, Lee VV, Elayda M, Lopez JA, Rasekh A, Wilson JM, Massumi A. Postoperative atrial fibrillation and mortality after coronary artery bypass surgery. *J Am Coll Cardiol*. 2004;43:742-748.

7. Creswell LL, Schuessler RB, Rosenbloom M, Cox JL. Hazards of postoperative atrial arrhythmias. *Ann Thorac Surg*. 1993;56:539-549.

8. Aranki SF, Shaw DP, Adams DH, Rizzo RJ, Couper GS, VanderVliet M, Collins JJ Jr, Cohn LH, Burstin HR. Predictors of atrial fibrillation after coronary artery surgery. Current trends and impact on hospital resources. *Circulation.* 1996;94:390-397.

9. Almassi GH, Schowalter T, Nicolosi AC, Aggarwal A, Moritz TE, Henderson WG, Tarazi R, Shroyer AL, Sethi GK, Grover FL, Hammermeister KE. Atrial fibrillation after cardiac surgery: a major morbid event? *Ann Surg*. 1997;226:501-511.

10. Mathew JP, Fontes ML, Tudor IC, Ramsay J, Duke P, Mazer CD, Barash PG, Hsu PH, Mangano DT; Investigators of the Ischemia Research and Education Foundation; Multicenter Study of Perioperative Ischemia Research Group. A multicenter risk index for atrial fibrillation after cardiac surgery. *JAMA*. 2004;;291:1720-1729.

11. Banach M, Rysz J, Drozdz JA, Okonski P, Misztal M, Barylski M, Irzmanski R, Zaslonka J. Risk factors of atrial fibrillation following coronary artery bypass grafting: a preliminary report. *Circ J*. 2006;70:438-441.

12. Mariscalco G, Engström KG. Postoperative atrial fibrillation is associated with late mortality after coronary surgery, but not after valvular surgery. *Ann Thorac Surg*. 2009;88:1871-1876.

13. Ahlsson A, Fengsrud E, Bodin L, Englund A. Postoperative atrial fibrillation in patients undergoing aortocoronary bypass surgery carries an eightfold risk of future atrial fibrillation and a doubled cardiovascular mortality. *Eur J Cardiothorac Surg*. 2010;37:1353-1359.

14. Andrews TC, Reimold SC, Berlin JA, Antman EM. Prevention of supraventricular arrhythmias after coronary artery bypass surgery. A meta-analysis of randomized control trials. *Circulation*. 1991;84(5 Suppl):III236-44.

15. Frost L, Mølgaard H, Christiansen EH, Hjortholm K, Paulsen PK, Thomsen PE. Atrial fibrillation and flutter after coronary artery bypass surgery: epidemiology, risk factors and preventive trials. *Int J Cardiol*. 1992;36:253-261.

16. Maisel WH, Rawn JD, Stevenson WG. Atrial fibrillation after cardiac surgery. *Ann Intern Med*. 2001;135:1061-1073.

17. Auer J, Weber T, Berent R, Ng CK, Lamm G, Eber B. Risk factors of postoperative atrial fibrillation after cardiac surgery. *J Card Surg*. 2005 Sep-Oct;20(5):425-31.

18. Leitch JW, Thomson D, Baird DK, Harris PJ. The importance of age as a predictor of atrial fibrillation and flutter after coronary artery bypass grafting. *J Thorac Cardiovasc Surg*. 1990;100:338-342.

19. Guarnieri T, Nolan S, Gottlieb SO, Dudek A, Lowry DR. Intravenous amiodarone for the prevention of atrial fibrillation after open heart surgery: the Amiodarone Reduction in Coronary Heart (ARCH) trial. *J Am Coll Cardiol*. 1999;34:343-347.

20. Gu S, Su PX, Liu Y, Yan J, Zhang XT, Wang TY. Low-dose amiodarone for the prevention of atrial fibrillation after coronary artery bypass grafting in patients older than 70 years. *Chin Med J (Engl).* 2009;122:2928-2932.

21. Pfisterer ME, Klöter-Weber UC, Huber M, Osswald S, Buser PT, Skarvan K, Stulz PM. Prevention of supraventricular tachyarrhythmias after open heart operation by low-dose sotalol: a prospective, double-blind, randomized, placebo-controlled study. *Ann Thorac Surg*. 1997;64:1113-1119.

22. Yagdi T, Nalbantgil S, Ayik F, Apaydin A, Islamoglu F, Posacioglu H, Calkavur T, Atay Y, Buket S. Amiodarone reduces the incidence of atrial fibrillation after coronary artery bypass grafting. *J Thorac Cardiovasc Surg*. 2003125:1420-1425.

23. Funk M, Richards SB, Desjardins J, Bebon C, Wilcox H. Incidence, timing, symptoms, and risk factors for atrial fibrillation after cardiac surgery. *Am J Crit Care*. 2003 Sep;12(5):424-33.

24. Yadava M, Hughey AB, Crawford TC. Postoperative atrial fibrillation: incidence, mechanisms, and clinical correlates. *Cardiol Clin*. 2014;32::627-636.

25. Lo B, Fijnheer R, Nierich AP, Bruins P, Kalkman CJ. C-reactive protein is a risk indicator for atrial fibrillation after myocardial revascularization. *Ann Thorac Surg*. 2005;79:1530-1535.

26. Gladstone DJ, Spring M, Dorian P, Panzov V, Thorpe KE, Hall J, Vaid H, O'Donnell M, Laupacis A, Côté R, Sharma M, Blakely JA, Shuaib A, Hachinski V, Coutts SB, Sahlas DJ, Teal P, Yip S, Spence JD, Buck B, Verreault S, Casaubon LK, Penn A, Selchen D, Jin A, Howse D, Mehdiratta M, Boyle K, Aviv R, Kapral MK, Mamdani M; EMBRACE Investigators and Coordinators. Atrial fibrillation in patients with cryptogenic stroke. *N Engl J Med*. 2014;370:2467-2477.

27. Sanna T, Diener HC, Passman RS, Di Lazzaro V, Bernstein RA, Morillo CA, Rymer MM, Thijs V, Rogers T, Beckers F, Lindborg K, Brachmann J; CRYSTAL AF Investigators. Cryptogenic stroke and underlying atrial fibrillation. *N Engl J Med*. 2014;370:2478-2486.

28. Reiffel J, Verma A, Halperin JL, Gersh B, Tombul S, Carrithers J, Sherfesee L, Kowey P. Rationale and design of REVEALAF: a prospective study of previously undiagnosed atrial fibrillation as documented by an insertable cardiac monitor in high-risk patients. *Am Heart J*. 2014;167:22-27.

29. Mitchell LB; CCS Atrial Fibrillation Guidelines Committee. Canadian Cardiovascular Society atrial fibrillation guidelines 2010: prevention and treatment of atrial fibrillation following cardiac surgery. *Can J Cardiol*. 2011;27:91-97.

30. European Heart Rhythm Association; European Association for Cardio-Thoracic Surgery, Camm AJ, Kirchhof P, Lip GY, Schotten U, Savelieva I, Ernst S, Van Gelder IC, Al-Attar N, Hindricks G, Prendergast B, Heidbuchel H, Alfieri O, Angelini A, Atar D, Colonna P, De Caterina R, De Sutter J, Goette A, Gorenek B, Heldal M, Hohloser SH, Kolh P, Le Heuzey JY, Ponikowski P, Rutten FH. Guidelines for the management of atrial fibrillation: the Task Force for the Management of Atrial Fibrillation of the European Society of Cardiology (ESC). *Eur Heart J*. 2010;31:2369-2429.

31. January CT, Wann LS, Alpert JS, Calkins H, Cigarroa JE, Cleveland JC Jr, Conti JB, Ellinor PT, Ezekowitz MD, Field ME, Murray KT, Sacco RL, Stevenson WG, Tchou PJ, Tracy CM, Yancy CW; ACC/AHA Task Force Members. 2014 AHA/ACC/HRS guideline for the management of patients with atrial fibrillation: a report of the American College of Cardiology/American Heart Association Task Force on practice guidelines and the Heart Rhythm Society. *Circulation*. 2014;130:e199-267.

32. Lowres N, Freedman SB, Gallagher R, Kirkness A, Marshman D, Orchard J, Neubeck L. Identifying postoperative atrial fibrillation in cardiac surgical patients posthospital discharge, using iPhone ECG: a study protocol. *BMJ Open*. 2015;13:e006849.

33. Qaddoura A, Kabali C, Drew D, van Oosten EM, Michael KA, Redfearn DP, Simpson CS, Baranchuk A. Obstructive sleep apnea as a predictor of atrial fibrillation after coronary artery bypass grafting: a systematic review and meta-analysis. *Can J Cardiol*. 2014;30:1516-1522.

34. Lang RM, Bierig M, Devereux RB, Flachskampf FA, Foster E, Pellikka PA, Picard MH, Roman MJ, Seward J, Shanewise JS, Solomon SD, Spencer KT, Sutton MS, Stewart WJ; Chamber Quantification Writing Group; AmericanSociety of Echocardiography's Guidelines and Standards Committee; European Association of Echocardiography. Recommendations for chamber quantification: a report from the AmericanSociety of Echocardiography's Guidelines and Standards Committee and the Chamber Quantification Writing Group, developed in conjunction with the European Association of Echocardiography, a branch of the European Society of Cardiology. *J Am Soc Echocardiogr*. 2005;18:1440-1463.

35. Glotzer TV, Hellkamp AS, Zimmerman J, Sweeney MO, Yee R, Marinchak R, Cook J, Paraschos A, Love J, Radoslovich G, Lee KL, Lamas GA; MOST Investigators. Atrial high rate episodes detected by pacemaker diagnostics predict death and stroke: report of the Atrial Diagnostics Ancillary Study of the MOde Selection Trial (MOST).*Circulation.* 2003;107:1614-1619.

36. Healey JS, Connolly SJ, Gold MR, Israel CW, Van Gelder IC, Capucci A, Lau CP, Fain E, Yang S, Bailleul C, Morillo CA, Carlson M, Themeles E, Kaufman ES, Hohnloser SH; ASSERT Investigators. Subclinical atrial fibrillation and the risk of stroke. *N Engl J Med.* 2012;366:120-129.

37. Weber-Krüger M, Gelbrich G, Stahrenberg R, Liman J, Kermer P, Hamann GF, Seegers J, Gröschel K, Wachter R; Find-AF(RANDOMISED) investigators. Finding atrial fibrillation in stroke patients: Randomized evaluation of enhanced and prolonged Holter monitoring--Find-AF(RANDOMISED) --rationale and design. *Am Heart J*. 2014;168:438-445.

38. Shinbane JS, Merkert, M, Fogoros, R, Mehta, V, Cao, M, Saxon, LA. Wearable Wireless Arrhythmia Detection Patches: Diagnostic Arrhythmia Yield, Time to First Arrhythmia, and Patient Compliance. *Heart Rhythm* 2013;10(5S):S305.

39. Shrivastav M, Padte S, Sinha N. Patient experience with a novel patch-like external loop recorder for cardiac arrhythmia detection in India. *Expert Rev Med Devices*. 2014 May;11(3):259-264.

14. Appendices

Appendix A: Product information for the Medtronic SEEQ mobile cardiac telemetry system.

Appendix B: Validation data of the SEEQ™ mobile cardiac telemetry system
Appendix C: Rhythm diagnoses collected by the Medtronic SEEQ™ mobile cardiac telemetry system in the SEARCH-AF trial.

Appendix D: Sample summary and episode reports from data collected by the Medtronic SEEQ™ mobile cardiac telemetry system.

Appendix E: Systematic review of completed and ongoing observational studies addressing the incidence of post-operative atrial arrhythmias after cardiac surgery.

Appendix F: Adverse cardiovascular endpoint definitions.

Appendix G: Major bleeding outcomes definition.

Appendix H: Serious adverse event collecting and reporting.

Appendix I: The CHA_2DS_2-VASC score.

Appendix A: Product information for the Medtronic SEEQ mobile cardiac telemetry system[1]

SEEQ™ MCT Wearable Sensor

- Each sensor can be worn for 7.5 days – up to 30 days
- Adhesive-backed sensor is applied to chest
- Communicates with the wireless transmitter
- The Wearable Sensor automatically captures events and/or patient can press the button to mark the event

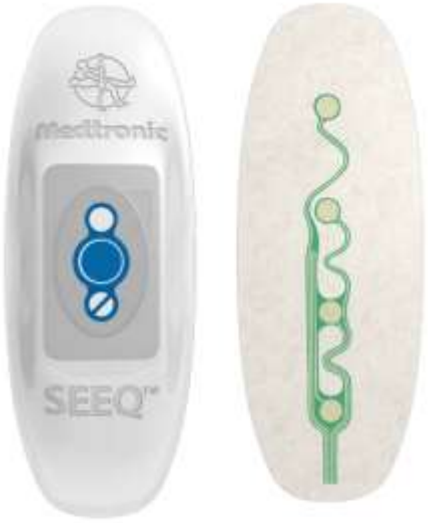

SEEQ™ MCT Transmitter

- Relays sensor data continuously via cellular signals to Medtronic Monitoring Center
- Rechargeable from standard electrical outlet
- Compact enough to tuck in purse or pocket
- Battery life is 12 hours
- Must be within 30 feet of the transmitter for successful data transmission

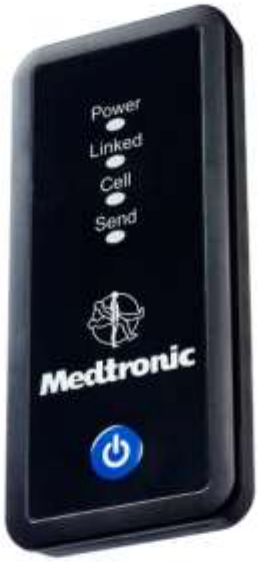

Appendix A: Product information for the Medtronic SEEQ mobile cardiac telemetry system[1]

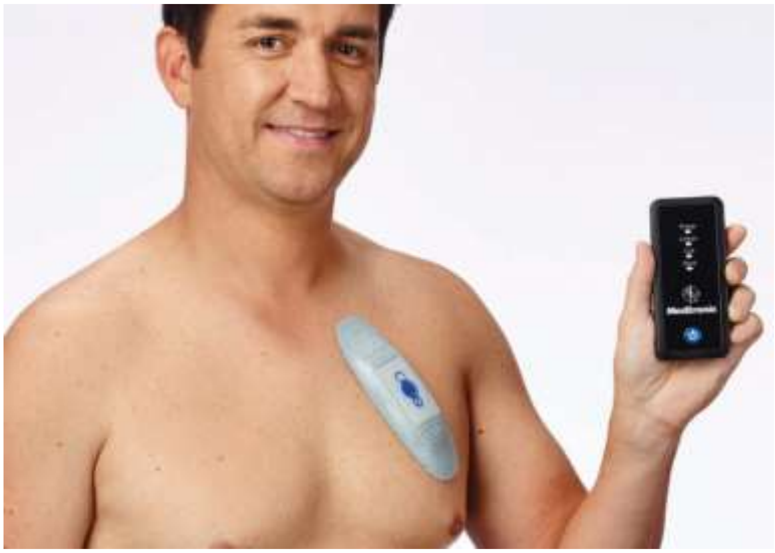

[1]http://www.medtronicdiagnostics.com/wcm/groups/mdtcom_sg/@mdt/documents/documents/seeq-system-overview.pdf. Accessed on January 18, 2015.

Appendix B: Validation data of the SEEQ™ mobile cardiac telemetry system

<u>Investigation of Monitoring Performance in Adherent Cardiac Telemetry (IMPACT) Study (www.clincial trials.gov NCT00919568)</u>

Note: This data is not published in a peer-reviewed journal. It was provided by Medtronic.

Objective: To compare the ambulatory arrhythmia monitoring performance of the Corventis NUVANT MCT System to a commercially available ambulatory cardiac monitoring system (Cardionet®)

Primary Endpoint

- Detection of clinically significant arrhythmias.

Study Design

- 20 patients undergoing concurrent monitoring with the Corventis NUVANT Mobile Cardiac Telemetry (MCT) System and a commercially available, clinically-approved MCT system (CardioNet® MCT; please refer to www.cardionet.com for details of the system). This system will be referred to as the "conventional" MCT system.
- The study duration was 30 days.
- Note: Medtronic had purchased the Corventis NUVANT MCT system; it is now marketed as the SEEQ™ mobile cardiac telemetry system.
- Patient reports from both systems evaluated by independent physician review panel consisting of 3 blinded physician reviewers.
- Reports were provided to each reviewer in random order.
- Final determination based on panel consensus.

Research Question: Were any of the arrhythmias clinically significant?

Results:

- Concordance between the NUVANT MCT system and conventional (Cardionet®) MCT was noted in 11 of 13 (85%) patients
- Discordance between the 2 systems was observed in 2 of 13 patients (15%). This was explained by difference in rhythm detection design (ie: differences in rhythm detection criteria) between the 2 systems.

Appendix C: Rhythm diagnoses collected by the Medtronic SEEQ™ mobile cardiac telemetry system in the SEARCH-AF trial.

For the SEARCH-AF trial, we intend the SEEQ™ mobile cardiac telemetry system to be used in a similar capacity as a 7-day Holter for post-cardiac surgical patients.

1) We will receive "End of Use Summary Reports" on a weekly basis. We do not require generation of Daily Reports for this trial. All Episode reports will be attached with the weekly End of Use Summary reports.

2) If there are arrhythmic events which met the "Notification Criteria", we do not require Medtronic to perform real-world notification of these events to the ordering physician or the research site or the research participant. Arrhythmic episodes meeting "Notification Criteria" will be included in the End of Use Summary report which are generated on a weekly basis.

Specific parameters of arrhythmic events under "Notification Criteria"

Arrhythmia	*Notification Criteria*
**VF**	*Yes*
**ICD discharge**	*Yes*
**VT**	*180 bpm and ≥10 beats*
**Wide complex tachycardia**	*180 bpm and ≥10 beats*
**PVCs**	*Never notified*
**Sinus bradycardia**	*≤30 bpm*
**Sinus tachycardia**	*≥150 bpm*
**SVT**	*≥150 bpm and ≥30 seconds*
**AF/AFL (fast)**	*≥150 bpm and ≥30 seconds*
**AF/AFL (slow)**	*≤30 bpm and ≥30 seconds*
**Pause**	*≥5 seconds*
**Mobitz I AV block**	*≤30 bpm*
**Mobitz II AV block**	*≤30 bpm*
**Isolated 2nd degree AV block**	*≤30 bpm*
**High degree AV block**	*≤30 bpm*
**3rd degree AV block**	*≤30 bpm*
**Patient-triggered ECG**	*None*
**Technicians discretion**	*Yes (Any)*

3) To assess AF burden in SEARCH-AF, we will have access on data pertaining to: (i) total of AF episodes; (ii) duration of each AF episode; and (iii) total duration of all AF episodes. In addition, we will have access to all rhythm strips which have been detected as AF by the device. This “raw data” will be provided by Medtronic. Please note that our adjudication results will not alter the Episode and End of Use reports as generated by Medtronic. In other words, if there is disagreement between our adjudicated events and Medtronic, the report as generated by Medtronic will not be altered. The results of the trial (as published in a scientific, peer-reviewed manuscript) will be based on the adjudicated results.

Appendix D: Sample summary and episode reports from data collected by the Medtronic SEEQ™ mobile cardiac telemetry system.

End Of Use Summary Report
Apr 19, 2014

Medtronic SEEQ™

Medtronic Monitoring Center
Phone 1-877-247-7449 Fax: 1-408-790-9375
E-mail medtronicmonitoring@medtronic.com

**Patient Name :**	Last, First
**Patient ID :**	8083784937297483927910
**DOB :**	Dec 12, 1965
**Gender :**	Male
**Address :**	Address Line 1<br>Address Line 2<br>Address Line 3
**Patient Phone :**	555-555-1212
**Phys. Diag. Code :**	427.31 : Atrial Fibrillation
**Medical record :**	

**Physician Name :**	Last, First
**Institution :**	Cardiology Clinic
**Address :**	Address Line 1<br>Address Line 2<br>Address Line 3
**Physician Phone :**	555-555-1213
**Physician Fax :**	555-555-1214

**System :**	SEEQ MCT	**Report ID :** 234
**Period :**	Apr 12, 2014 to Apr 19, 2014	

Heart Rate (bpm)

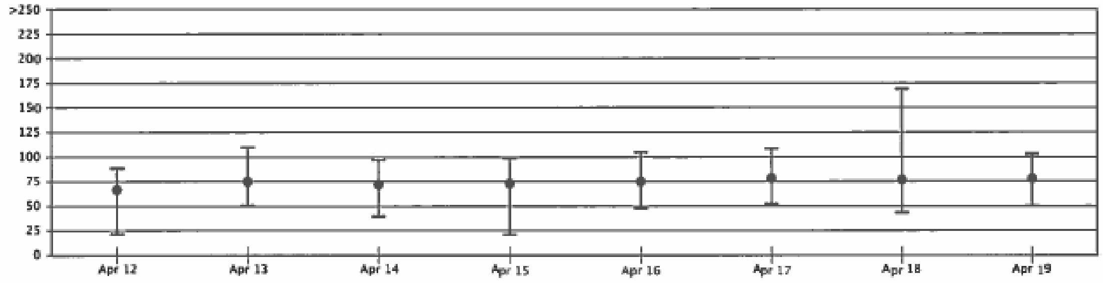

Atrial Fibrillation : Daily Burden (Hrs:Min) Episodes >= 5 min

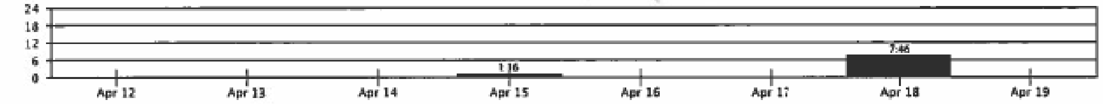

Atrial Fibrillation : Episode Overview (#)

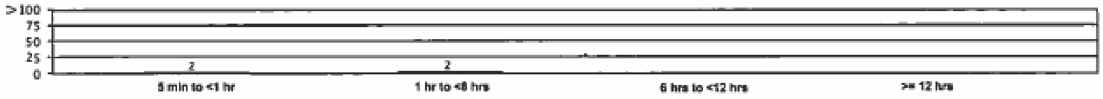

Episode Summary

Date	Time	Notify	Preliminary Observation	Trigger Type	Ref ID
Apr 12, 2014	10:06:45 EST		Rhythm: Bradycardia<br>HR Min 40bpm, Max: 50bpm, Avg: 45bpm	Baseline	1012320
Apr 12, 2014	18:58:26 EST	⚑	Rhythm:Bradycardia, Pause<br>HR: Min 20bpm, Max: 60bpm, Avg: 50bpm<br>Observations: Bradycardia with Pauses of 3.54 and 3.12 seconds.<br>Spoke to patient at 19.05 EST who reported feeling lightheaded	Patient	1012324
Apr 15, 2014	06:54:45 EST	⚑	Rhythm:Atrial Fibrillation, Bradycardia, Pause<br>HR: Min: 20bpm, Max: 110bpm, Avg: 65bpm<br>Observations: Pause of 4.82 seconds.	Auto	1013567
Apr 18, 2014	18:51:13 EST	⚑	Rhythm:Atrial Fibrillation<br>HR: Min: 130bpm, Max 170bpm, Avg. 150bpm	Auto	1013690
Apr 18, 2014	19:03:10 EST	⚑	Rhythm: Bradycardia<br>HR: Min; 25bpm, Max: 60bpm, Avg: 40bpm	Auto	1013711
Apr 18, 2014	19:28:33 EST	⚑	Rhythm:Atrial Fibrillation, Monomorphic VT, PACs/PJCs, PVCs<br>HR: Min: 90bpm, Max: 150bpm, Avg: 120bpm	Auto	1013834

The observations provided by Medtronic Monitoring, Inc. are preliminary and are intended for review and interpretation by the prescribing physician as part of the information used by the physician to make a diagnosis. Any observations in this report must be confirmed by a physician. Please login at www.medtronicmonitoring.com to review additional data related to this patient

Page 1 of 3

Copyright 2014 Medtronic Monitoring, Inc.
UC201502268 EN© Medtronic Inc. 2014. Minneapolis, MN. All Rights Reserved. Printed in USA. 08/2014

All patient and clinical data are fictitious and for demonstration purposes only

PRM00226 Rev C (08/2014)

Appendix D: Sample summary and episode reports from data collected by the Medtronic SEEQ™ mobile cardiac telemetry system

End Of Use Summary Report
Apr 19, 2014

Medtronic SEEQ™

Medtronic Monitoring Center
Phone: 1-877-247-7449 Fax: 1-408-790-9375
E-mail: medtronicmonitoring@medtronic.com

**Patient Name :** Last, First	**Physician Name:** Last, First

Episode Details

Apr 12, 2014 10:06:45 EST — Rhythm: Bradycardia; HR: Min: 40bpm, Max: 50bpm, Avg: 45bpm — Baseline — 1012320

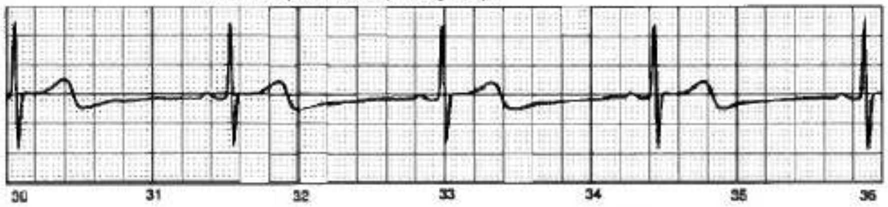

Apr 12, 2014 18:58:26 EST — Rhythm: Bradycardia, Pause; HR: Min: 20bpm, Max: 60bpm, Avg: 50bpm — Patient — 1012324
Observations: Bradycardia with Pauses of 3.54 seconds and 3.12 seconds. Spoke to patient at 19:05 EST who reported feeling lightheaded

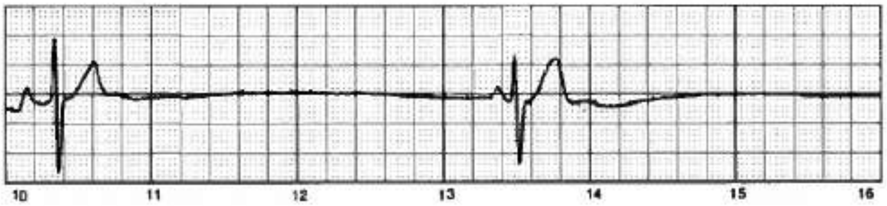

Apr 15, 2014 06:54:45 EST — Rhythm: Atrial Fibrillation, Bradycardia, Pause; HR: Min: 20bpm, Max: 110bpm, Avg: 65bpm — Auto — 1013567
Observations: Bradycardia with Pause of 4.82 seconds

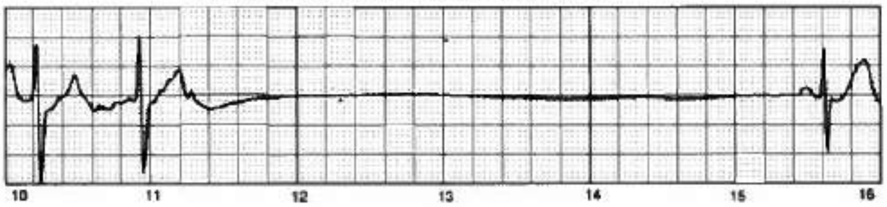

Apr 18, 2014 18:51:13 EST — Rhythm: Atrial Fibrillation; HR: Min: 130bpm, Max: 170bpm, Avg: 150bpm — Auto — 1013690

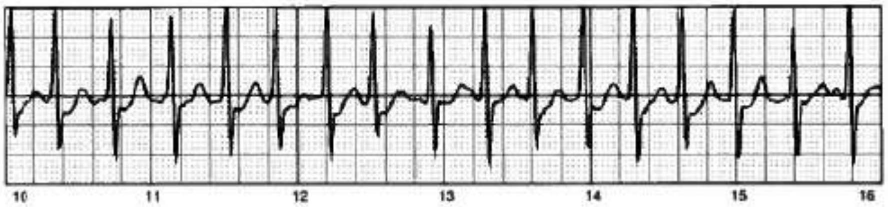

The observations provided by Medtronic Monitoring, Inc. are preliminary and are intended for review and interpretation by the prescribing physician as part of the information used by the physician to make a diagnosis. Any observations in this report must be confirmed by a physician. Please login at www.medtronicmonitoring.com to review additional data related to this patient.

Page 2 of 3

Copyright 2014 Medtrosic Monitoring, Inc.

UC201502269 EN © Medtronic, Inc. 2014. Minneapolis, MN. All Rights Reserved. Printed in USA. 08/2014

All patient and clinical data are fictitious and for demonstration purposes only

PRM00226 Rev C (08/2014)

Appendix D: Sample summary and episode reports from data collected by the Medtronic SEEQ™ mobile cardiac telemetry system

End Of Use Summary Report
Apr 19, 2014

Medtronic SEEQ™

Medtronic Monitoring Center
Phone: 1-877-247-7449 Fax: 1-408-790-9375
E-mail: medtronicmonitoring@medtronic.com

**Patient Name :** Last, First	**Physician Name:** Last, First

Apr 18, 2014 19:03:10 EST — Rhythm: Bradycardia — HR: Min: 25bpm, Max: 60bpm, Avg: 40bpm — Auto — 1013711

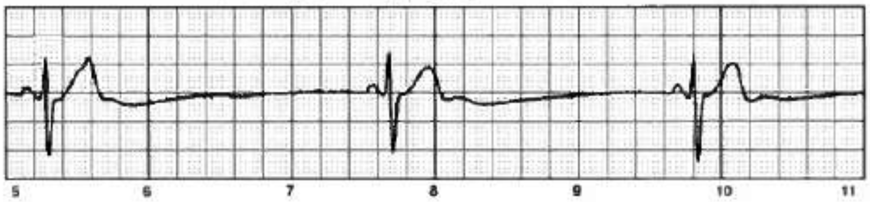

Apr 18, 2014 19:28:33 EST — Rhythm: Atrial Fibrillation, Monomorphic VT, PACs/PJCs, PVCs — HR: Min: 90bpm, Max: 150bpm, Avg: 120bpm — Auto — 1013834

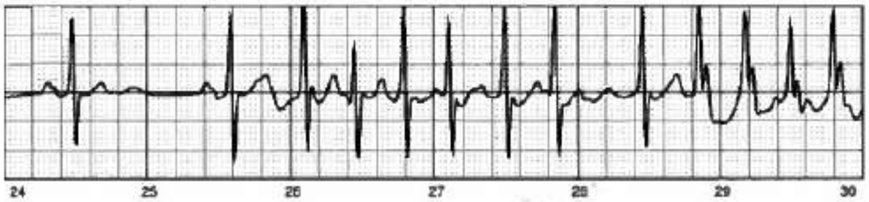

Bradycardia Statistics

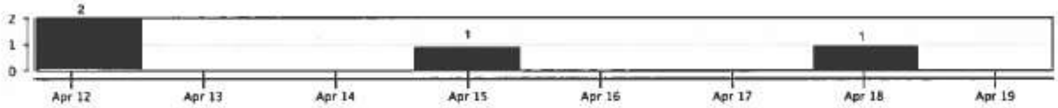

Pause Statistics

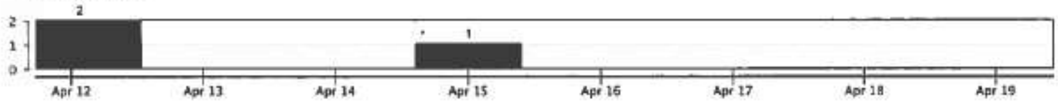

Physician Interpretation

Date ______________ Physician Signature ______________________________

The observations provided by Medtronic Monitoring, Inc. are preliminary and are intended for review and interpretation by the prescribing physician as part of the information used by the physician to make a diagnosis. Any observations in this report must be confirmed by a physician. Please login at www.medtronicmonitoring.com to review additional data related to this patient.

Copyright 2014 Medtronic Monitoring, Inc.

Page 3 of 3

All patient and clinical data are fictitious and for demonstration purposes only

PRM00226 Rev C (06/2014)

UC201502260 EN © Medtronic, Inc. 2014. Minneapolis, MN. All Rights Reserved. Printed in USA. 06/2014

Appendix D: Sample summary and episode reports from data collected by the Medtronic SEEQ™ mobile cardiac telemetry system

Episode Report
Feb 02, 2014 03:21:33

Medtronic SEEQ™

Medtronic Monitoring Center
Phone: 1-877-247-7449 Fax: 1-408-790-9375
E-mail: medtronicmonitoring@medtronic.com

**Patient Name:**	Last, First
**Patient ID:**	89628939743892
**DOB:**	Dec 11, 1959
**Gender:**	Male
**Address:**	Address<br>Address<br>City, State Zip
**Patient Phone:**	1-555-555-1212
**Phys. Diag. Code :**	427.31 : Atrial Fibrillation
**Medical Record:**	11902

**Physician:**	Last, First
**Institution:**	Cardiology Center
**Address:**	Address<br>Address<br>City, State Zip
**Physician Phone:**	1-555-555-1221
**Physician Fax:**	1-555-555-1222

System: SEEQ MCT **Ref ID:** 24246
Period: Feb 01, 2014 - Feb 14, 2014

DateTime: Feb 02, 2014 03:21:33 EST
Trigger Type: Automatic
Rhythm: Atrial Fibrillation
HR: Min: 80 bpm, Max: 110 bpm

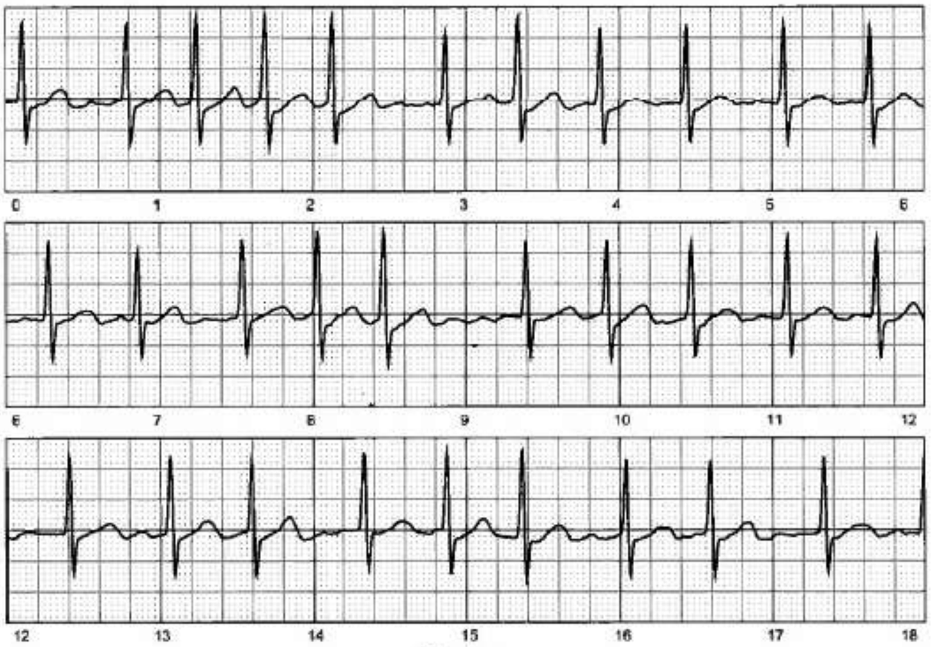

25 mm / sec

The observations provided by Medtronic Monitoring, Inc. are preliminary and are intended for review and interpretation by the prescribing physician as part of the information used by the physician to make a diagnosis. Any observations in this report must be confirmed by a physician. Please login at www.medtronicmonitoring.com to review additional data related to this patient.

Copyright 2014 Medtronic Monitoring, Inc.

All patient and clinical data are fictitious and for demonstration purposes only

Page 1 of 2
PRM60220 Rev B (08/2014)

Appendix E: Systematic review of completed and ongoing observational studies addressing the incidence of post-operative atrial arrhythmias after cardiac surgery (current as of May 13 2015).

Study	Study design	Sample size	Status	Rhythm monitoring modality	Comments
**Funk et al.**	Observational, prospective study	302	Published	Wearable cardiac event monitor (recording of heart rhythm will commence upon subject-based self-triggering)	Subjects wore 14-day rhythm monitors which are activated by self-triggering (ie: non-continuous rhythm monitoring).
**Lowres et al.**	Observational, prospective pilot study	50	Ongoing, study design has been published	An i-phone based application to monitor subjects' heart rhythm	Subjects will be asked to record their rhythm status with an i-Phone app on a daily basis for 30 days after discharge from cardiac surgery.
**MONITOR-AF (NCT01395836)**	Observational, prospective, 2-arm study	50	Ongoing	Implantable loop recorder (ILR)	Subjects' rhythm status will be monitored for 1 year to assess for AF occurrence.
**Medtronic Reveal XT study (NCT01526343)**	Observational, prospective study	100	Ongoing	Implantable loop recorder (ILR) and "Traditional monitoring" (e.g. Holter and ECG)	All subjects underwent surgical AF ablation (as a stand-alone or concomitant procedure). An ILR was implanted in each subject. All subjects underwent traditional monitoring (ECG and Holter). The primary outcome was the number and burden of atrial tachyarrhythmia detected during pre-specified post-operative time intervals. The comparison of interest will be ILR vs. traditional monitoring (each subject serves as his/her own comparator).

Appendix E: Systematic review of completed and ongoing observational studies addressing the incidence of post-operative atrial arrhythmias after cardiac surgery (current as of May 13 2015).

PUBMED search strategy: ("Cardiac Surgical Procedures"[mh] OR "Cardiac Surgical Procedures"[tiab] OR "Coronary Artery Bypass"[mh] OR "Coronary Artery Bypass"[tiab]) AND ("atrial fibrillation"[mh] OR "atrial fibrillation"[tiab]) AND "randomized controlled trial"[publication type].

Using this strategy, we did not find any published RCT on this particular topic. After omitting "AND "randomized controlled trial"[publication type]", we were able to identify 2 studies (Funk and Lowres).

Similar keywords used in the www.clinicaltrials.gov database

This yielded 2 more ongoing studies in the clinicaltrials.gov database: MONITOR-AF (NCT01395836) and Medtronic Reveal XT study (NCT01526343).

References:

Funk M, Richards SB, Desjardins J, Bebon C, Wilcox H. Incidence, timing, symptoms, and risk factors for atrial fibrillation after cardiac surgery. *Am J Crit Care*.2003 Sep;12(5):424-33.

Lowres N, Freedman SB, Gallagher R, Kirkness A, Marshman D, Orchard J, Neubeck L. Identifying postoperative atrial fibrillation in cardiac surgical patients post hospital discharge, using iPhone ECG: a study protocol. *BMJ Open*. 2015 Jan 13;5(1):e006849.

Appendix F: Major adverse cardiovascular endpoint (MACE) definition

Event	Definition
**All-cause mortality**	Death from any cause
**Myocardial infarction (third universal definition of myocardial infarction)[1]**	The term acute myocardial infarction (MI) should be used when there is evidence of myocardial necrosis in a clinical setting consistent with acute myocardial ischemia. Under these conditions any one of the following criteria meets the diagnosis for MI:<br><br>1) Detection of a rise and/or fall of cardiac biomarker values [preferably cardiac troponin (cTn)] with at least one value above the 99th percentile upper reference limit (URL) and with at least one of the following:<br><br>- Symptoms of ischaemia.<br>- New or presumed new significant ST-segment–T wave (ST–T) changes or new left bundle branch block (LBBB).<br>- Development of pathological Q waves in the ECG.<br>- Imaging evidence of new loss of viable myocardium or new regional wall motion abnormality.<br>- Identification of an intracoronary thrombus by angiography or autopsy.<br><br>2) Cardiac death with symptoms suggestive of myocardial ischemia and presumed new ischaemic ECG changes or new LBBB, but death occurred before cardiac biomarkers were obtained, or before cardiac biomarker values would be increased.<br><br>3) Percutaneous coronary intervention (PCI) related MI is arbitrarily defined by elevation of cTn values (>5 x 99th percentile URL) in patients with normal baseline values (≤99th percentile URL) or a rise of cTn values >20% if the baseline values are elevated and are stable or falling. In addition, either (i) symptoms suggestive of myocardial ischemia or (ii) new ischaemic ECG changes or (iii) angiographic findings consistent with a procedural complication or (iv) imaging

	demonstration of new loss of viable myocardium or new regional wall motion abnormality are required.<br><br>4) Stent thrombosis associated with MI when detected by coronary angiography or autopsy in the setting of myocardial ischemia and with a rise and/or fall of cardiac biomarker values with at least one value above the 99th percentile URL.<br><br>5) Coronary artery bypass grafting (CABG) related MI is arbitrarily defined by elevation of cardiac biomarker values (>10 x 99th percentile URL) in patients with normal baseline cTn values (≤99th percentile URL). In addition, either (i) new pathological Q waves or new LBBB, or (ii) angiographic documented new graft or new native coronary artery occlusion, or (iii) imaging evidence of new loss of viable myocardium or new regional wall motion abnormality.
**Stroke**	Diagnosis of stroke will require the abrupt onset of focal neurological symptoms lasting at least 24 hours. Accompanying neurovascular imaging information will be used for event adjudication, if it is performed.[2]
**Systemic embolism**	Clinical history consistent with an acute loss of blood flow to a peripheral artery (or arteries), which is supported by evidence of embolism from surgical specimens, autopsy, angiography, or other objective testing. [2]

[1] Thygesen K, Alpert JS, Jaffe AS, Simoons ML, Chaitman BR, White HD; Joint ESC/ACCF/AHA/WHF Task Force for the Universal Definition of Myocardial Infarction, Katus HA, Lindahl B, Morrow DA, Clemmensen PM, Johanson P, Hod H, Underwood R, Bax JJ, Bonow RO, Pinto F, Gibbons RJ, Fox KA, Atar D, Newby LK, Galvani M, Hamm CW, Uretsky BF, Steg PG, Wijns W, Bassand JP, Menasché P, Ravkilde J, Ohman EM, Antman EM, Wallentin LC, Armstrong PW, Simoons ML, Januzzi JL, Nieminen MS, Gheorghiade M, Filippatos G, Luepker RV, Fortmann SP, Rosamond WD, Levy D, Wood D, Smith SC, Hu D, Lopez-Sendon JL, Robertson RM, Weaver D, Tendera M, Bove AA, Parkhomenko AN, Vasilieva EJ, Mendis S. Third universal definition of myocardial infarction. *Circulation.* 2012 Oct 16;126(16):2020-35.

[2] http://www.nejm.org/doi/suppl/10.1056/NEJMoa1107039/suppl_file/nejmoa1107039_protocol.pdf Accessed July 29 2015.

Appendix G: Bleeding outcome definitions[1]

Acute clinically overt bleeding is defined as new onset, visible bleeding, signs, and/or symptoms suggestive of bleeding with confirmatory imaging techniques which can detect the presence of blood (e.g. ultrasound, computed tomography, magnetic resonance imaging)

The definition of ***major bleeding*** is adapted from the International Society on Thrombosis and Hemostasis (ISTH) definition[2].

Major bleeding is defined as a bleeding event which is:

- Acute clinically overt bleeding accompanied by one or more of the following:
  - A decrease in hemoglobin (Hgb) of 2 g/dL or more
  - A transfusion of 2 or more units of packed red blood cells
  - Bleeding that occurs in at least one of the following critical sites:
    - Intracranial
    - Intra-spinal
    - Intraocular (within the corpus of the eye; thus, a conjunctival bleed is not an intraocular bleed)
    - Pericardial
    - Intra-articular
    - Intramuscular with compartment syndrome
    - Retroperitoneal.
- Bleeding that is fatal.

Clinically relevant non-major bleeding event: The definition of clinical relevant non-major bleeding will be acute or sub-acute clinically overt bleeding that does not satisfy the criteria for major bleeding and that leads to one or more of the following:

1) hospital admission for bleeding.
2) physician-guided medical or surgical treatment for bleeding.
3) a change in antithrombotic therapy.

Minor bleeding events: All acute clinically overt bleeding events not meeting the criteria for either major bleeding or clinically relevant non-major bleeding will be classified as minor bleeding.

Fatal bleeding event is defined as a bleeding event that the adjudication committee determines to be the primary cause of death or contributes directly to death.

All acute clinically overt bleeding events will be adjudicated as a major bleeding event, or clinically relevant non-major bleeding event. Minor bleeding events will not be adjudicated.

[1] http://www.nejm.org/doi/suppl/10.1056/NEJMoa1107039/suppl_file/nejmoa1107039_protocol.pdf Accessed July 31 2015.

[2]Schulman S, Kearon C; Subcommittee on Control of Anticoagulation of the Scientific and Standardization Committee of the International Society on Thrombosis and Haemostasis.Definition of major bleeding in clinical investigations of antihemostatic medicinal products in non-surgical patients. *J Thromb Haemost*. 2005;3:692-694.

Appendix H: Serious adverse outcome collection and reporting.

ADVERSE EVENT REPORTING

All Serious Adverse Events (SAEs) that occur following the subject's written consent to participate in the study through 30 days of discontinuation of dosing must be reported to reported to the study sponsor.

Adverse Events

- An Adverse Event [AE] is defined as any new untoward medical occurrence or worsening of a pre-existing medical condition in a patient or clinical investigation subject administered a medicinal product and that does not necessarily have a causal relationship with this treatment. An AE can therefore be any unfavorable and unintended sign (including an abnormal laboratory finding, for example), symptom, or disease temporally associated with the use of a medicinal product, whether or not considered related to the investigational product.
- The causal relationship to a medicinal product is determined by a physician and should be used to assess all adverse events (AEs). The causal relationship can be one of the following:
  - Related: There is a reasonable causal relationship between a medicinal product and the AE.
  - Not Related: There is not a reasonable causal relationship between a medicinal product and the AE.
- The term "reasonable causal relationship" means there is evidence to suggest a causal relationship.
- Adverse events can be spontaneously reported or elicited during open-ended questioning, examination, or evaluation of a subject. (In order to prevent reporting bias, subjects should not be questioned regarding the specific occurrence of one or more adverse events).

Serious Adverse Events

A **Serious Adverse Event (SAE)** is any untoward medical occurrence at any dose that:

- results in death
- is life-threatening (defined as an event in which the subject was at risk of death at the time of the event; it does not refer to an event which hypothetically might have caused death if it were more severe)
- requires inpatient hospitalization or causes prolongation of existing hospitalization (see ***NOTE****: below for exceptions)
- results in persistent or significant disability/incapacity
- is a congenital anomaly/birth defect
- is an important medical event, defined as a medical event that may not be immediately life-threatening or result in death or hospitalization but, based on appropriate medical and

scientific judgment, may jeopardize the subject or may require intervention (e.g., medical, surgical) to prevent one of the other serious outcomes listed above. Examples of such events include but are not limited to intensive treatment in an emergency department or at home for allergic bronchospasm; blood dyscrasias or convulsions that do not result in hospitalization.

– Potential drug-induced liver injury (DILI) is also considered an important medical event--see the DILI section below for a definition of a potential DILI event.

Suspected transmission of an infectious agent (eg, pathogenic or non-pathogenic) via a medicinal product is an SAE.

Although pregnancy, overdose, and cancer are not always serious by regulatory definition, these events must be handled as SAEs.

***NOTE:** *The following hospitalizations are not considered SAEs:*

– A visit to the emergency room or other hospital department lasting less than 24 hours that does not result in admission (unless considered an "important medical event" or a life-threatening event)

– Elective surgery planned before signing consent

– Admissions as per protocol for a planned medical/surgical procedure

– Routine health assessment requiring admission for baseline/trending of health status (eg, routine colonoscopy)

– Medical/surgical admission other than remedying ill health state that was planned before study entry. Appropriate documentation is required in these cases

– Admission encountered for another life circumstance that carries no bearing on health status and requires no medical/surgical intervention (eg, lack of housing, economic inadequacy, caregiver respite, family circumstances, administrative reason)

Serious Adverse Event Collecting and Reporting

Following the subject's written consent to participate in the study, all SAEs, whether related or not related to a medicinal product, must be collected, including those thought to be associated with protocol-specified procedures. All SAEs must be collected that occur during the screening period and within 30 days of discontinuing dosing of a medicinal product. If applicable, SAEs must be collected that relate to any later protocol-specific procedure (such as follow-up skin biopsy).

The investigator should report any SAE occurring after these time periods that is believed to be related to a medicinal product or protocol-specified procedure.

An SAE report should be completed for any event where doubt exists regarding its status of seriousness.

If the investigator believes that an SAE is not related to a medicinal product, but is potentially related to the conditions of the study (such as withdrawal of previous therapy, or a complication of a study procedure), the relationship should be specified in the narrative section of the SAE Report Form.

SAEs, whether related or unrelated to a medicinal product, and pregnancies must be reported to the study sponsor within 1 business day. SAEs must be reported initially through the electronic case report form (eCRF); if necessary, the central coordinating centre will follow up to ask sites to record the SAEs on the CIOMS Report Form. Pregnancies will be reported on a Pregnancy Surveillance Form.

If only limited information is initially available, follow-up reports are required. (Note: Follow-up SAE reports should include the same investigator term(s) initially reported.)

If an ongoing SAE changes in its intensity or relationship to a medicinal product or if new information becomes available, a follow-up SAE report should be sent within 1 business day to the study sponsor (or designee) using the same procedure used for transmitting the initial SAE report.

All SAEs should be followed to resolution or stabilization.

SAE Reconciliation for SAEs related to BMS products

The sponsor will reconcile the clinical database SAE cases transmitted to BMS Global Pharmacovigilance (GPV&E). Frequency of reconciliation will be done every three months and once prior to study database lock. BMS GPV&E will e-mail upon request from the sponsor, the GPV&E reconciliation report. Requests for reconciliation should be sent to aepbusinessprocess@bms.com. The data elements listed on the GPV&E reconciliation report will be used for case identification purposes. If the investigator determines a case was not transmitted to BMS GPV&E, the case will be sent immediately.

Health Authority Reporting (Worldwide)

Investigators must adhere to local Health Authority Reporting Requirements and timelines. For studies conducted under a local health authority:

- Adverse drug reactions that are Serious, Unexpected, and at least Possibly Related to the drug (Suspected Unexpected Serious Adverse Reaction, SUSAR) and that have not previously been reported in the Investigators' Brochure, or reference safety information document will be reported promptly, within local reporting timelines, to the health authority in writing by the Investigator.
- A clear description of the suspected reaction should be provided along with an assessment as to whether the event is drug or disease related.
- The Investigator shall notify the health authority of any unexpected fatal or life threatening experience associated with the use of the drugs as soon as possible but no later than 7 calendar days after initial receipt of the information.

All SAEs should be reported to the study sponsor by entering the data into the SAE form of the eCRF. This will trigger an email notification to the coordination centre and sponsor. Site staff will also be required to print the SAE form and fax it to the Coordination Centre at 416-864-3016.

Non-Serious Adverse Events

A nonserious adverse event is an AE not classified as serious.

Non-Serious Event Collecting and Reporting

The collection of non-serious adverse event (NSAE) information should begin at initiation of a medicinal product. Nonserious adverse event information should also be collected from the start of a placebo lead-in period or other observational period intended to establish a baseline status for the subjects.

Nonserious AEs should be followed to resolution or stabilization, or reported as SAEs if they become serious. Follow-up is also required for nonserious AEs that cause interruption or discontinuation of a medicinal product, or those that are present at the end of study treatment as appropriate.

Nonserious Adverse Events related to any BMS product are provided to BMS via annual safety reports (if applicable), and interim or final study reports.

Laboratory Test Abnormalities

The following laboratory abnormalities should be captured and reported as appropriate:

- Any laboratory test result that is clinically significant or meets the definition of an SAE
- Any laboratory test result abnormality that required the subject to have a medicinal product discontinued or interrupted
- Any laboratory test result abnormality that required the subject to receive specific corrective therapy.

It is expected that wherever possible, the clinical rather than the laboratory term will be used by the reporting investigator (eg, use the term anemia rather than low hemoglobin value).

Laboratory test abnormalities are provided to the study sponsor via annual safety reports (if applicable), and interim or final study reports.

Pregnancy

If, following initiation of the investigational product, it is subsequently discovered that a study subject is pregnant or may have been pregnant at the time of medicinal product exposure, including during at least 5 half-lives after medicinal product administration, the medicinal product will be permanently discontinued in an appropriate manner (eg, dose tapering if necessary for subject safety).

The investigator must immediately notify the study sponsor of this event via the Pregnancy Surveillance Form within 24 hours and in accordance with SAE reporting procedures.

Follow-up information regarding the course of the pregnancy, including perinatal and neonatal outcome and, where applicable, offspring information must be reported on a Pregnancy Surveillance Form.

Any pregnancy that occurs in a female partner of a male study participant should be reported to the study sponsor. Information on this pregnancy may also be collected on the Pregnancy Surveillance Form.

Protocol-required procedures for study discontinuation and follow-up must be performed on the subject unless contraindicated by pregnancy (eg, x-ray studies). Other appropriate pregnancy follow-up procedures should be considered if indicated.

Overdose

Include any product-specific definition of overdose in addition to the following mandatory statement as the last sentence.

An overdose is defined as the accidental or intentional administration of any dose of a medicinal product that is considered both excessive and medically important. All occurrences of overdose must be reported as SAEs.

Drug Induced Liver Injury (DILI)

Wherever possible, timely confirmation of initial liver-related laboratory abnormalities should occur prior to the reporting of a potential DILI event. All occurrences of potential DILIs, meeting the defined criteria, must be reported as SAEs.

Potential drug induced liver injury is defined as

1. AT (ALT or AST) elevation > 3 times upper limit of normal (ULN)

AND

2. Total bilirubin > 2 times ULN, without initial findings of cholestasis (elevated serum alkaline phosphatase),

AND

3. No other immediately apparent possible causes of AT elevation and hyperbilirubinemia, including, but not limited to, viral hepatitis, pre-existing chronic or acute liver disease, or the administration of other drug(s) known to be hepatotoxic.

Other Safety Considerations

Any significant worsening noted during interim or final physical examinations, electrocardiograms, x-rays, and any other potential safety assessments, whether or not these procedures are required by the protocol, should also be recorded as a nonserious or serious adverse event, as appropriate, and reported accordingly.

Appendix I: The CHA_2DS_2-VASC score[1]

	Clinical feature	Score
C	Congestive heart failure **(or LVEF ≤40%)**	1
H	Hypertension	1
A	Age ≥ 75 years	2
D	Diabetes	1
S	Stroke / TIA / Thromboembolism	2
V	Vascular disease (MI, PAD, or aortic plaque)	1
A	Age between 65-74 years	1
Sc	Sex category: Female	1
	Maximum score	**10**

	CHA_2DS_2-VASc Score	Annual Stroke Risk %
"Low risk"	0	0
"Moderate risk"	1	1.3
"High risk"	2	2.2
	3	3.2
	4	4.0
	5	6.7
	6	9.8
	7	9.6
	8	12.5
	9	15.2

[1]European Heart Rhythm Association; European Association for Cardio-Thoracic Surgery, Camm AJ, Kirchhof P, Lip GY, Schotten U, Savelieva I, Ernst S, Van Gelder IC, Al-Attar N, Hindricks G, Prendergast B, Heidbuchel H, Alfieri O, Angelini A, Atar D, Colonna P, De Caterina R, De Sutter J, Goette A, Gorenek B, Heldal M, Hohloser SH, Kolh P, Le Heuzey JY, Ponikowski P, Rutten FH. Guidelines for the management of atrial fibrillation: the Task Force for the Management of Atrial Fibrillation of the European Society of Cardiology (ESC).*Eur Heart J.* 2010;31:2369-2429.

Post-Surgical Enhanced Monitoring for Cardiac Arrhythmias and Atrial Fibrillation (SEARCH-AF): A randomized controlled trial

Protocol number: SEARCH-AF-001

Executive steering committee:
Andrew C. T. Ha, MD, MSc; Atul Verma, MD; C. David Mazer, MD; Bobby Yanagawa, MD, PhD; Subodh Verma, MD, PhD.

Coordinating centre:
Applied Health Research Centre, Li Ka Shing Knowledge Institute
St. Michael's Hospital
30 Bond Street, Toronto, Ontario, Canada.
M5B1W8

Version date:
June 30 2020

Study sponsor:
St. Michael's Hospital
30 Bond Street, Toronto, Ontario, Canada.
M5B1W8

INVESTIGATOR STATEMENT AND SIGNATURE

Protocol Number: SEARCH-AF-001

Post-Surgical Enhanced Monitoring for Cardiac Arrhythmias and Atrial Fibrillation (SEARCH-AF): A randomized controlled trial

I have read the protocol described herein. I agree to conduct this study in compliance with the protocol, Good Clinical Practice, and other applicable regulatory requirements.

Site Principal Investigator:

Signature: ____

Date: ____

Printed Name: _____________________________________

Site Name: _______________________________________

This protocol has been developed by the Principal Investigators and its contents are the intellectual property of this group. It is an offence to reproduce or use the information and data in this protocol for any purpose other than the SEARCH-AF trial without prior approval from the Principal Investigators.

Signature Page

Dr. Andrew C.T. Ha<br>Principal Investigator	Name	2020-June-30<br>Date *(YYYY-MM-DD)*
Dr. Atul Verma<br>Principal Investigator	Name	2020-June-30<br>Date *(YYYY-MM-DD)*
Dr. Subodh Verma<br>Principal Investigator	Name	2020-June-30<br>Date *(YYYY-MM-DD)*

Table of Contents **Page**

1. Background and Rationale...11

2. Study Objectives and Hypothesis..15
2.1 Primary Objective
2.2 Secondary Objectives
2.3 Study Hypothesis

3. Ethical considerations……………………………………………….……...16
3.1 Good Clinical Practice (GCP)
3.2 Research Ethics Board/Institutional Review Board
3.3 Informed Consent Process
3.4 Participant Confidentiality

4. Eligibility Criteria..18
4.1 Inclusion Criteria
4.2 Exclusion Criteria
4.3 Women of childbearing potential
4.4 Discontinuation of Subjects from Trial Participation

5. Study Design...20
5.1 Study Description
5.2 Executive Committee
5.3 Clinical Events Committee
5.4 Baseline Visit
5.5 Randomization
5.6 Proposed Study Intervention
5.6.1 30-day continuous cardiac rhythm monitor (Intervention group)
5.6.2 Usual care (control group)
5.6.3 Additional 14-day cardiac rhythm monitoring at 6±1 months
5.7 Methods to Protect against Potential Bias
5.8 Proposed Duration of the Intervention Period
5.9 Proposed Frequency and Duration of Follow-up
5.10 Study Flowchart
5.11 Primary Outcome
5.12 Secondary Outcomes
5.13 Prescription of Oral Anticoagulation
5.14 Other Considerations

6. Adverse events...30
6.1 Problem Reporting
6.2 Serious adverse events

7. Study Duration……...30
7.1 Expected Study Duration of Subject Participation

7.2 Expected Total Study Duration

8. Concomitant Medications/Natural Remedies/Foods..30

9. Prohibited Medications and Procedures...31

10. Study Evaluations/Procedures..31
11.1 Laboratory Evaluations and Specimen Collection
11.2 Questionnaires

11. Trial Management..31

12. Statistical considerations..32
12.1 Sample Size Calculation
12.2 Details of Statistical Analysis
12.3 Subgroup Analysis

13. References…………………………..……………………………………..……....35

14. Appendices…………………………………………………………………...39

LIST OF ABBREVIATIONS

ACC	American College of Cardiology
AE	Adverse event
AF	Atrial fibrillation
AFL	Atrial flutter
AHA	American Heart Association
ASSERT-II	Prevalence of sub-clinical atrial fibrillation using an implantable cardiac monitor in patients with cardiovascular risk factors detected
BMI	Body mass index
CABG	Coronary artery bypass grafting surgery
CEC	Clinical events committee
CCS	Canadian Cardiovascular Society
CI	Confidence interval
CIHR	Canadian Institutes of Health Research
CNS	Central nervous system
COPD	Chronic obstructive pulmonary disease
CRYSTAL-AF	Study of continuous cardiac monitoring to assess atrial fibrillation after cryptogenic stroke
CVA	Cerebrovascular accident
ECG	Electrocardiogram
EMBRACE	30-day cardiac event monitor belt for recording atrial fibrillation after a cerebral ischemic event
ESC	European Society of Cardiology
HRS	Heart Rhythm Society
ICD	Implantable cardioverter defibrillator
ILR	Implantable loop recorder
INR	International normalized ratio
IRB	Institutional review board
LV	Left ventricular
MACE	Major adverse cardiac events
OAC	Oral anticoagulation
OR	Odds ratio
POAF/AFL	Post-operative atrial fibrillation / atrial flutter
PCI	Percutaneous coronary intervention
PI	Principal investigator
PVC	Premature ventricular contraction
RCT	Randomized controlled trial
REB	Research ethics board
SAE	Serious adverse event
TIA	Transient ischemic attack
WOCBP	Women of childbearing potential

Protocol Synopsis

**Project title**	**Post-Surgical Enhanced Monitoring for Cardiac Arrhythmias and Atrial Fibrillation (SEARCH-AF)**
**Short title**	SEARCH-AF
**Study sponsor**	St. Michael's Hospital, Toronto, Ontario, Canada.
**Recruitment and Participating sites**	A total of 396 subjects will be recruited from cardiac surgical centres in Canada.
**Phase / Regulatory status**	Phase IV / Randomized Controlled Trial.
**Study Hypothesis**	Among post-cardiac surgical subjects without a previous history of AF/AFL and with risk factors for stroke and POAF/AFL, a strategy of enhanced cardiac rhythm monitoring will result in a 3-fold or 7% absolute increase in the rate of AF/AFL detection (defined as a cumulative AF/AFL burden of ≥6 minutes or documentation of AF/AFL by a 12-lead ECG) when compared to usual care at 30 days after randomization.
**Study Objective**	The aim of SEARCH-AF is to evaluate a novel diagnostic tool for detecting POAF/AFL in cardiac surgical subjects during the early, sub-acute post-operative period.<br><br>***Primary objective:***<br>1) To test whether enhanced cardiac rhythm monitoring with an adhesive, continuous monitor results in higher rates of AF/AFL detection at 30 days after randomization for post-cardiac surgical subjects who are at risk for developing post-operative atrial arrhythmias, when compared to usual care.<br><br>***Secondary objectives:***<br>1) To assess the incidence of AF/AFL during the early, sub-acute post-operative period (defined as the first 30 days after randomization) and at 6±1 months after surgery.<br>2) To assess the proportion of subjects with continuous AF and/or AFL lasting ≥ 24 hours during the early, sub-acute post-operative period and at 6±1 months after surgery.<br>3) To assess subjects' adherence and incidence of adverse events from use of a wearable adhesive cardiac rhythm monitoring system.

	4) To assess the proportion of subjects who are prescribed with oral anticoagulation at 45 days after discharge from cardiac surgery, at 6±1 months, and at 9±1 months after surgery.<br>5) To assess the proportion of subjects who experienced major bleeding events at 45 days after discharge from cardiac surgery.<br>6) To evaluate factors associated with development of POAF/AFL during the early, sub-acute post-operative period.
**Study Design**	Open-label, two-arm RCT comparing a strategy of enhanced cardiac rhythm monitoring vs. usual care in 396 post-cardiac surgical subjects who are at risk for developing POAF/AFL.
**Primary endpoint**	Proportion of subjects with a cumulative AF/AFL burden of ≥6 minutes or documentation of AF/AFL by a 12-lead ECG within 30 days after randomization. Clinical endpoints will be adjudicated by an independent committee of physicians.
**Sample size**	N=396 (198 per group, after accounting for a 2% attrition rate).
**Participants / Study Duration**	A total of 396 subjects will be enrolled over a 36-month period. The last subject enrolled will have a 9-month follow-up after hospital discharge, extending the total study duration to 45 months.
**Study Population**	Post-cardiac surgical subjects at high risk of stroke, defined as having a CHA_2DS_2-VASC score of ≥4 or CHA_2DS_2-VASC score of ≥2 with additional risk factors for developing POAF/AFL. These subjects must not have a history of AF/AFL pre-operatively.
**Inclusion Criteria**	1) Male or female age ≥18 years.<br>2) Isolated CABG or valve replacement/repair ± CABG performed at the index surgical procedure.<br>3) At high risk of stroke and developing POAF/AFL, defined as having a CHA_2DS_2-VASC score of ≥4.<br>*or*<br>A CHA_2DS_2-VASC score of ≥2 with at least 1 of the following risk factors for developing POAF/AFL:<br>(i) Chronic obstructive pulmonary disease.<br>(ii) Sleep apnea.<br>(iii) Impaired renal function (defined as creatinine clearance <60 ml/min or <60 ml/min/1.73m^2).<br>(iv) Echocardiographic evidence of at least mild left atrial

	enlargement (defined as ≥41 mm on M-mode, ≥59 ml or ≥29 mL/m^2 on biplane volume assessment).<br>(v) Elevated body mass index (defined as BMI ≥30).<br>(vi) Combined surgery (CABG + valve repair/replacement).
**Exclusion Criteria**	1) Atrial fibrillation or flutter at the time of randomization.<br>2) Known previous history of AF/AFL, diagnosed pre-operatively (note: documentation of a history of AF/AFL without accompanying rhythm proof will suffice).<br>3) Documentation of continuous AF/AFL for ≥24 hours during the in-hospital stay for the index cardiac surgery.<br>4) Subjects who, at the discretion of the treating cardiac surgery team, would be treated and discharged with oral anticoagulation due to POAF/AFL.<br>5) Mechanical valve replacement.<br>6) Current or anticipated treatment with oral anticoagulation for indications other than AF/AFL.<br>7) Hospitalization for ≥10 days (for the index cardiac surgery, with day #0 being the day of surgery).<br>8) Planned discharge from hospital with a type IC or III anti-arrhythmic drug.<br>9) Having received >5 grams of IV and/or oral amiodarone during hospitalization for the index cardiac surgical procedure.<br>10) Women of childbearing potential (WOCBP).<br>11) History of percutaneous or surgical left atrial ablation for AF.<br>12) Presence of a cardiac implantable electronic device with a functioning atrial lead (pacemaker, implantable cardioverter defibrillator, or cardiac resynchronization device).<br>13) Presence of an implantable loop recorder.<br>14) History of left atrial appendage ligation, removal, or occlusion.<br>15) Subjects with known allergies or hypersensitivities to adhesives or hydrogel.<br>16) Inability to provide written informed consent.<br>17) Current or anticipated participation in another randomized controlled trial in which the interventional drug or device is known to affect the incidence of the primary or secondary outcomes of this study.
**Intervention**	Subjects who meet inclusion criteria will be randomized in a 1:1 ratio to one of the following 2 arms:<br><br>***Enhanced cardiac rhythm monitoring (intervention group)***<br>▪ Starting on the day of randomization, subjects will undergo a maximum

<table>
<tr>
<td></td>
<td>of 30 days of continuous cardiac rhythm monitoring with a wearable adhesive cardiac rhythm monitoring system. They will receive another 14 days of continuous cardiac rhythm monitoring at 6±1 months after hospital discharge.

Usual care (control group)
▪ Subjects will not receive any form of protocol-mandated continuous cardiac rhythm monitoring until at 6±1 months after hospital discharge. Performance of additional ECG and/or Holter monitoring will be left at the discretion of the subjects’ treating physicians.

At 6±1 months after surgery, subjects in both groups will undergo 14 days of continuous cardiac rhythm monitoring with a wearable adhesive cardiac rhythm monitoring system.</td>
</tr>
<tr>
<td>Follow-up</td>
<td>All subjects will have a follow-up visit at 31-90 days after hospital discharge and at 6±1 months after surgery. A 12-lead ECG will be performed for all subjects at follow-up visits. A telephone follow-up will occur at 9±1 months after surgery.</td>
</tr>
<tr>
<td>Study flow chart</td>
<td>
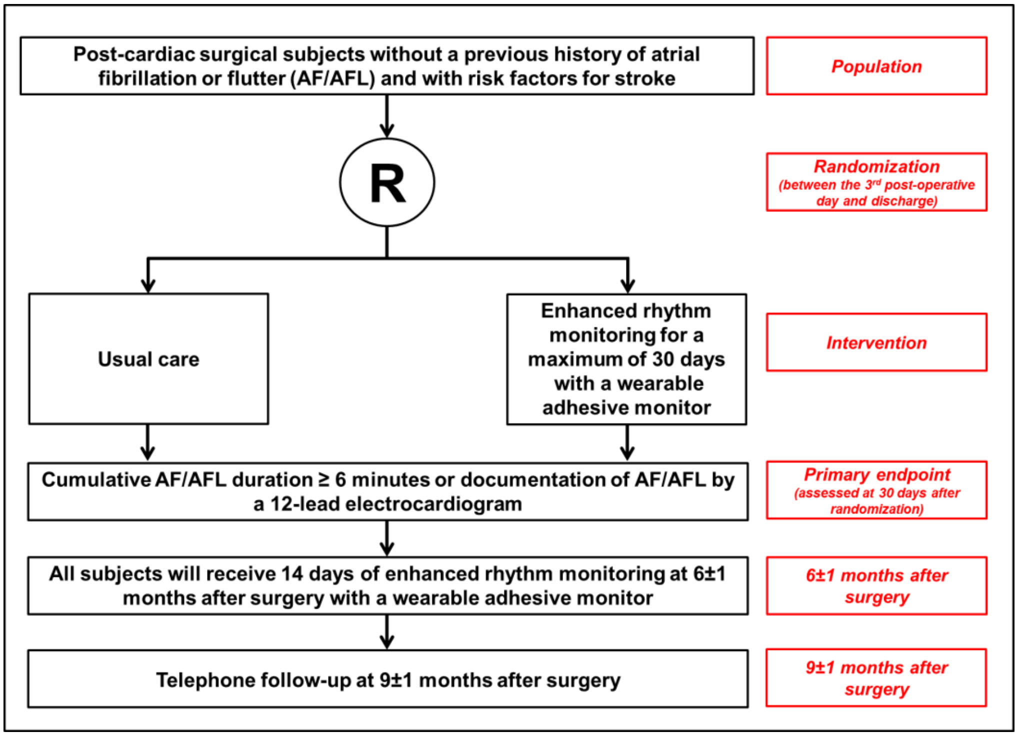

</td>
</tr>
</table>

1. BACKGROUND AND RATIONALE

1.1 *The incidence of stroke after cardiac surgery is not trivial*

Stroke is a serious complication after cardiac surgery and its incidence in the post-operative phase has remained unchanged despite advances in surgical techniques. Data from administrative databases and observational registries suggest that the incidence of post-operative stroke after cardiac surgery ranges from 0.8 to 5.2% [1]. Given that millions of cardiac surgical procedures are performed worldwide, postoperative stroke poses significant functional and/or economic burden to patients, their families, and healthcare systems.

1.2 New-onset POAF is an important risk factor for stroke in cardiac surgical patients

Several risk factors have been identified for post-operative stroke in the cardiac surgical population. Older age, renal insufficiency, diabetes, left ventricular (LV) dysfunction, hypertension, low cardiac output syndrome, and atrial fibrillation (AF) had been found to be associated with increased stroke risk in the post-operative phase. In particular, new-onset post-operative atrial fibrillation (POAF) is an important risk factor for stroke. Administrative data from Ontario, Canada suggested that new-onset POAF was associated with a 50% increase in peri-operative stroke risk [2]. A systematic review of 11 cohort studies evaluating the clinical impact of new-onset POAF demonstrated a doubling of in-hospital, peri-operative stroke risk (OR 2.23 [95% CI, 1.78 to 2.80]) [3]. The presence of new-onset POAF is also associated with a greater risk of stroke beyond the peri-operative phase. In a retrospective Californian cohort derived from administrative claims data of ≈77,000 cardiac surgical patients, the risk of stroke at 1 year after hospitalization for the index surgery was higher in those with new-onset POAF than those without [4]. Finally, emerging data suggest that new-onset POAF may be a harbinger for future AF at >1 year after cardiac surgery, well beyond the post-operative phase [5]. Taken together, new-onset POAF is an important risk factor for stroke in the cardiac surgical population during the peri-operative phase and beyond. Given that POAF is so common after cardiac surgery (15-48%) [6-20], effective and timely diagnosis of AF in this population may expedite delivery of appropriate stroke prevention therapies. This is particularly relevant for those at high risk for stroke.

1.3 The incidence of POAF is not well-defined beyond hospital discharge

There have been many studies describing the incidence of POAF in the post-cardiac surgical setting, with reported rates ranging from 15-48% [6-20]. The incidence of POAF may be even higher for patients undergoing valve surgery, with reported rates of up to 60% [7,9,16,19]. However, it is important to note that the great majority of these studies reported ***in-hospital*** rates of POAF. **As such, there is very little data on the incidence of POAF in the sub-acute, post operative phase after hospital discharge (e.g. within the first month after hospital discharge).** Published reports suggest that the incidence of POAF within 30 days after cardiac surgery is low, in the range of 2-5% [21-24]. It should also be noted that most, if not all, of these studies assessed patients' rhythm status with a single 12-lead ECG on post-operative day #30. However, trying to detect for POAF/AFL in the sub-acute phase with a single ECG has poor sensitivity when compared to continuous cardiac rhythm monitoring. Accordingly, there is likely an element of ***detection bias*** in published reports which described the incidence of POAF in the post-cardiac surgical setting. The intensity of AF detection was much greater during hospitalization (the immediate post-operative phase) when compared to the post-discharge period. This may contribute to the marked decline in the reported incidence of POAF for cardiac

surgical patients as reported by various studies.

Evidence in favor of this argument comes from an observational study by Funk et al. in which cardiac surgical patients wore 14-day event recorders after hospital discharge [25]. To our knowledge, this was the only published study which evaluated the rhythm status of cardiac surgical patients after hospital discharge with a more sensitive modality than a 12-lead ECG. Patients transmitted their cardiac rhythm status on a daily and symptom-based basis. With this enhanced cardiac rhythm monitoring strategy, the incidence of POAF was found to be 14% within 2 weeks after hospital discharge [25]. In this study, it should be noted that the rhythm status of participants was not continuously recorded but rather was only triggered by the subject. Therefore, it was quite possible and likely that asymptomatic AF episodes would not have been detected by this study's monitoring approach. While it is generally accepted that new-onset POAF is a transient phenomenon which declines to negligible levels by 3 months, the true incidence of POAF in the sub-acute phase and beyond is not clearly defined. It may be higher than we traditionally assume, particularly during the first month after hospital discharge.

1.4 Many cardiac surgical patients are at high risk for AF-related stroke

The great majority of cardiac surgical patients have concomitant cardiovascular co-morbidities, including hypertension, diabetes, older age, kidney disease, peripheral artery disease, and cerebrovascular disease. Applying validated stroke risk prediction tools such as the CHA_2DS_2-VASC scoring schema, a substantial portion of cardiac surgical patients were found to be at high risk for stroke if AF is diagnosed. In a study by Gialdini et al. which included 11,837 cardiac surgical patients with new onset POAF, 86.0% and 38.5% of them had a CHA_2DS_2-VASC score of ≥2 and ≥4, respectively [4]. Given that a sizeable proportion of cardiac surgical patients are at high risk for stroke when AF is diagnosed in the post-operative state, this highlights the need to develop strategies to effectively detect and diagnose AF in this population.

1.5 Oral anticoagulation may be underused in cardiac surgical patients with POAF

There is data to suggest that oral anticoagulation (OAC) may be under-prescribed for cardiac surgical patients with POAF. Amongst 445 post-CABG patients with POAF enrolled in the prospective Perioperative Cardiovascular Surgical Care (CAPS-Care) registry, a sub-study of the Society of Thoracic Surgery database, only 173 patients (39%) were discharged on warfarin in spite of the fact that 80% of them had a $CHADS_2$ score of ≥2 [26]. Factors such as concern over post-operative bleeding, absence of high-quality guideline recommendations on OAC use in the POAF setting, and the prevailing belief that POAF is a transient phenomenon likely contribute to OAC non-use in this otherwise high-risk population. If, however, we can demonstrate the POAF continues to be an ongoing, frequent, and recurrent issue for these patients soon after hospital discharge, this will support the concept of early OAC initiation after surgery if POAF is diagnosed.

1.6 Extended cardiac rhythm monitoring improves the rate of AF detection

There is considerable interest in using extended cardiac rhythm monitoring strategies to detect AF in selected patient subsets at high-risk for stroke. The EMBRACE trial randomized patients with cryptogenic stroke or TIA to extended cardiac rhythm monitoring with a wearable 30-day monitor or to usual care which consisted of a 24-hour Holter monitor [27]. The extended monitoring strategy resulted in a 5-fold increase in the detection of AF or AFL for ≥30 seconds

when compared to usual care. In the CRYSTAL-AF trial, patients with cryptogenic stroke were randomized to intensive cardiac rhythm monitoring with an implantable loop recorder (ILR) or to usual care. By 6 months, the rate of AF detection was 6 times higher in the ILR group when compared to usual care [28]. Using ILR technology, two trials (ASSERT-II; www.clinicaltrials.gov NCT01694394 and REVEAL-AF [29]) are evaluating the rate of AF detection in subjects who are at risk for AF-related stroke but without a prior history of documented AF.

In the post-cardiac surgical population, we posit that the first month after cardiac surgery continues to represent a vulnerable period for development of new-onset POAF, and that a strategy of enhanced cardiac rhythm monitoring may be particularly important in detecting AF during this sub-acute period, particularly in those patients at high risk for stroke. Based on published studies in other patient subsets [27,28], an extended rhythm monitoring strategy may improve the rate of AF detection in the target population of this study.

This concept is unexplored in the cardiac surgical population and represents an important unanswered, actionable question. If we demonstrate a significant increase in detectable and actionable AF during this period, it will provide important information regarding the rhythm monitoring practices and anticoagulation needs for stroke prevention in this high-risk patient cohort.

1.7 A novel 30-day cardiac rhythm monitoring system to detect AF

Presently, extended cardiac rhythm monitoring can be achieved in three ways. First, the traditional approach involves the use of multiple 24h, 48h, 7-day or 14-day Holter monitors. This approach is limited due to poor patient compliance, in part related to skin irritation caused by adhesive skin contact electrodes. The second option is with an external event loop recorder, but most available devices are cumbersome for patients to wear and can only store a limited number and duration of episodes which may necessitate repeat clinic visits to download the recorded episodes. There is also inherent delay from the time of AF detection to diagnosis with this monitoring system since episodes need to be transmitted, interpreted, and reported by separate, specialized personnel. As such, this system is limited in its user-friendliness and efficiency of data communication. A third option is insertion of an implantable loop recorder (ILR). However, this is an invasive procedure involving a much more expensive device. The indwelling nature of the ILR diminishes its attractiveness for patients who only require extended cardiac rhythm monitoring for a relatively short period of time, such as over several weeks to months.

This trial will afford the opportunity to evaluate a wearable adhesive cardiac rhythm monitoring system for AF detection over a short-term period. The Medtronic SEEQ™ mobile cardiac telemetry system is a novel, wearable technology which can provide up to 30 days of continuous rhythm monitoring (***appendix A-D***). The monitoring device is an adhesive, water-resistant patch which is applied over the patient's anterior chest wall. An attractive feature of this technology is that detected episodes will be directly transmitted from the patient to a centralized data centre where they can be readily analyzed by trained personnel. This can potentially reduce the turnaround time from AF detection to delivery of actionable data to clinicians.

As of October 1 2018, the CardioStat® cardiac rhythm monitoring system (Icentia Inc., Quebec, Canada) will replace the Medtronic SEEQ™ device when subjects undergo protocol-mandated cardiac rhythm monitoring procedures **(*appendix E-F*)**. This is a wearable adhesive cardiac rhythm monitoring device which is applied over the subject's anterior chest wall. Upon completion of the monitoring period, the device will be mailed back to the manufacturer (Icentia Inc., Quebec, Canada) for data extraction. Results will be available to the site investigator for review within 10 business days of receipt of the device.

1.8 Systematic reviews performed to date:

No systematic review has been performed examining the specific research question of our proposed trial (enhanced cardiac monitoring vs. usual care in the rate of POAF/AFL detection after cardiac surgery). We performed a series of literature reviews in PUBMED and www.clinicaltrials.gov to examine if similar studies are being conducted or had been published on this topic. The PUBMED search strategy was described in ***appendix G*** and is current as of May 13 2015.

(1) Novelty of the present research question: We performed a PUBMED search using the following keywords ("MeSH" and "TIAB"): atrial fibrillation, cardiac surgery, randomized controlled trial, ambulatory electrocardiography. We did not identify any published RCT which examined the specific research question of our proposed trial (enhanced cardiac monitoring with a continuous rhythm monitoring modality vs. usual care in the rate of POAF/AFL detection after cardiac surgery). In the www.clinicaltrials.gov database, we did not identify any ongoing, planned, completed, or terminated RCT which addressed our specific research question. To our knowledge, our research question has not been addressed by any ongoing or completed RCT, suggesting that our proposed question merits justification for further pursuit.

(2) Ongoing / completed studies (non-RCTs) examining the role of enhanced cardiac rhythm monitoring in the detection of POAF/AFL after cardiac surgery: We employed a similar approach as above to search for ongoing or completed non-randomized studies evaluating the role of continuous cardiac rhythm monitoring to detect for POAF/AFL in cardiac surgical subjects. We identified 4 studies and they are described in appendix G. The only completed study (Funk M. et al.) employed an older, relatively cumbersome monitoring system [25]. Most importantly, rhythm monitoring was triggered by self-activation and therefore this system could not monitor cardiac rhythm on a continuous basis. The other 3 ongoing studies are small (50-100 subjects); 2 of them employed ILR technology for continuous rhythm monitoring and thus required a separate invasive procedure for placement of the device. The study by Lowres et al. utilizes an i-Phone application to evaluate for AF on a daily basis; hence subjects' rhythm status is not monitored on a continuous basis [30].

1.9 The need for a randomized controlled trial

Recent studies have shown that enhanced cardiac rhythm monitoring can substantially increase the rate of AF detection in patients with cryptogenic stroke, when compared to usual care which typically consisted of a single 24-hour Holter [27,28]. Since POAF is so common after cardiac surgery and given that many of these patients will be at high risk for stroke (and hence potentially eligible for treatment with OAC if AF is detected), a trial which similarly compares the diagnostic yield of enhanced AF detection to usual care in the sub-acute, post-operative

period (ie: within the first month after surgery) will introduce new knowledge to a previously unexplored aspect in the peri-operative management of cardiac surgery patients. If enhanced cardiac rhythm monitoring can indeed detect a high rate of POAF which normally will not have been diagnosed with usual care, this can potentially change clinical practice in regards to the monitoring practices and anticoagulation choices for stroke prevention in these high-risk patients.

Presently, there is little data to inform clinicians on the optimal monitoring strategy for POAF in post-cardiac surgical patients, particularly after hospital discharge. This is reflected by the paucity of recommendations in this area by AF guidelines from major cardiovascular societies such as the Canadian Cardiovascular Society, American Heart Association, and the European Society of Cardiology [31-33].

Accordingly, a RCT will introduce high-quality evidence to an important topic regarding the optimal cardiac rhythm monitoring strategy for post-cardiac surgical patients. Successful completion of a RCT may potentially change clinical practice in this arena.

2. Study Objectives

The aim of SEARCH-AF is to evaluate an enhanced monitoring strategy for detecting atrial fibrillation or flutter (AF/AFL) during the sub-acute, post-discharge period of cardiac surgical subjects who underwent CABG and/or valve surgery. The primary purpose of this study is to compare this enhanced monitoring strategy to usual care and determine if it results in greater rates of AF/AFL detection for high-risk subjects during the sub-acute, post-operative phase after cardiac surgery. The ultimate goal of this research is to potentially improve our ability to identify post-operative AF/AFL in this population, which in turn may facilitate delivery of appropriate stroke prevention therapies.

2.1 Primary Objective:

1) To test whether enhanced cardiac rhythm monitoring with an adhesive, continuous monitoring device results in higher rates of AF/AFL detection during the subacute, post-discharge period of cardiac surgical subjects who are at risk of developing post-operative atrial arrhythmias.

2.2 Secondary Objectives:

1) To assess the incidence of AF/AFL of cardiac surgical subjects during: (i) the subacute, post-discharge period and (ii) late period (6±1 months after surgery).
2) To assess the proportion of subjects with continuous AF and/or AFL lasting ≥ 24 hours during the subacute, post-discharge period of subjects after cardiac surgery.
3) To assess subjects' adherence and incidence of adverse events from use of the Medtronic SEEQ™ system or the CardioStat® cardiac rhythm monitoring device.
4) To assess the proportion of subjects who are prescribed with oral anticoagulation at 45 days after discharge, at 6±1 months, and at 9±1 months from cardiac surgery.
5) To assess the proportion of subjects who experienced major bleeding events during the 45 day-period after discharge from cardiac surgery.

6) To evaluate factors associated with development of POAF/AFL during the subacute, post-discharge period of subjects after cardiac surgery.

2.3 Study Hypothesis
Among post-cardiac surgical subjects without a previous history of AF/AFL but with risk factors for developing POAF/AFL, a strategy of enhanced cardiac rhythm monitoring (using an adhesive continuous monitoring system) results in a 3-fold or a 7% absolute increase in the rate of AF/AFL detection (defined as a cumulative AF/AFL burden ≥6 minutes or documentation of AF/AFL by a 12-lead ECG) when compared to usual care within 30 days after randomization.

3. Ethical considerations

3.1 Good Clinical Practice (GCP)

This study will be conducted in accordance with Good Clinical Practice (GCP), as defined by the International Conference on Harmonisation (ICH) and in accordance with the ethical principles underlying European Union Directive 2001/20/EC and the United States Code of Federal Regulations, Title 21, Part 50 (21CFR50).

The study will be conducted in compliance with the protocol. The protocol, any amendments, and the subject informed consent will receive Institutional Review Board/Independent Ethics Committee (IRB/IEC) approval/favorable opinion before initiation of the study.

All potential serious breaches must be reported to the study sponsor (St. Michael's Hospital, Toronto, Canada) immediately. A serious breach is a breach of the conditions and principles of GCP in connection with the study or the protocol, which is likely to affect, to a significant degree, the safety or physical or mental integrity of the subjects of the study or the scientific value of the study.

Study personnel involved in conducting this study will be qualified by education, training, and experience to perform their respective tasks.

This study will not use the services of study personnel where sanctions have been invoked or where there has been scientific misconduct or fraud (eg, loss of medical licensure; debarment).

3.2 Research Ethics Board/Institutional Review Board

Before study initiation, the investigator must obtain written and dated approval from the REB/IRB for the following: protocol, consent form, subject recruitment materials/process (e.g. advertisements), and any other written information which will be provided to subjects.

The investigator or sponsor should also provide the REB/IRB with device information and a copy of any guidance documents or information about the device to be provided to subjects, and any updates.

The investigator should provide the REB/IRB with reports, updates, and other information (e.g. expedited safety reports, amendments, and administrative letters) according to regulatory requirements or institution procedures.

The principal site investigator will be responsible for obtaining REB/IRB approval and annual renewal throughout the duration of the study.

3.3 Informed Consent Process

Investigators must ensure that subjects or, in those situations where consent cannot be given by subjects, their legally acceptable representative are clearly and fully informed about the purpose, potential risks, and other critical issues regarding clinical studies in which they volunteer to participate.

Investigators must:

1) Provide a copy of the consent form and written information about the study in the language in which the subject is most proficient prior to clinical study participation. The language must be non-technical and easily understood.

2) Allow time necessary for subject or subject's legally acceptable representative to inquire about the details of the study.

3) Obtain an informed consent signed and personally dated by the subject or the subject's legally acceptable representative and by the person who conducted the informed consent discussion.

4) Obtain the REB/IRB's written approval of the written informed consent form and any other information to be provided to the subjects, prior to the beginning of the study, and after any revisions are completed for new information.

5) If informed consent is initially given by a subject's legally acceptable representative or legal guardian, and the subject subsequently becomes capable of making and communicating their informed consent during the study, then consent must additionally be obtained from the subject.

6) Revise the informed consent whenever important new information becomes available that is relevant to the subject's consent. The investigator, or a person designated by the investigator, should fully inform the subject or the subject's legally acceptable representative or legal guardian, of all pertinent aspects of the study and of any new information relevant to the subject's willingness to continue participation in the study. This communication should be documented.

Participants may withdraw consent at any time during the course of the trial. The informed consent form will be signed and dated by the participant and the person who conducted the informed consent discussion. The original signed informed consent form will be retained in the participant's study files and a copy of the signed form will be provided to the participant.

3.4 Participant Confidentiality

All subject related information including Case Report Forms, evaluation forms, reports, etc. will be kept strictly confidential. All records will be kept in a secure, locked location and only research staff will have access to the records. Subjects will be identified only by means of a coded number specific to each subject. All computerized databases will identify subjects by numeric codes only, and will be password protected.

4. Eligibility Criteria

4.1 Inclusion Criteria

1) Male or female age ≥18 years.
2) Isolated CABG or valve replacement/repair +/- CABG performed at the index surgical procedure.
3) At elevated risk of stroke and for having POAF/AFL, defined as having a CHA_2DS_2-VASC score of ≥4 *or* ≥2 with at least 1 of the following risk factors for developing POAF/AFL:
   (i) Chronic obstructive pulmonary disease.
   (ii) Sleep apnea [34].
   (iii) Impaired renal function (defined as creatinine clearance < 60 ml/min or <60 ml/min/1.73m^2).
   (iv) Echocardiographic evidence of at least mild left atrial enlargement (defined as ≥41 mm on M-mode, ≥59 ml or ≥29 mL/m^2 on biplane volume assessment [35] from an echocardiogram performed within 12 months of study enrollment).
   (i) Elevated body mass index (defined as BMI ≥30).
   (ii) Combined surgery (CABG + valve repair/replacement).
4) Able to provide written informed consent.

4.2 Exclusion Criteria

1) Atrial fibrillation or flutter at the time of randomization.
2) Known previous history of AF/AFL, diagnosed pre-operatively (note: documentation of a history of AF/AFL without accompanying rhythm proof will suffice).
3) Documentation of continuous AF/AFL for ≥24 hours during the in-hospital stay for the index cardiac surgery.
4) Subjects who, at the discretion of the treating cardiac surgery team, would be treated and discharged with oral anticoagulation due to POAF/AFL.
5) Mechanical valve replacement.
6) Current or anticipated treatment with oral anticoagulation for indications other than AF/AFL.
7) Hospitalization for ≥10 days (for the index cardiac surgery, with day #0 being the day of surgery).
8) Planned discharge from hospital with a type IC or III anti-arrhythmic drug.
9) Having received >5 grams of IV and/or oral amiodarone during hospitalization for the index cardiac surgical procedure.
10) Women of childbearing potential (WOCBP) (please refer to section 4.3 for definition).
11) History of percutaneous or surgical left atrial ablation for AF.
12) Presence of a cardiac implantable electronic device with a functioning atrial lead (pacemaker, implantable cardioverter defibrillator, or cardiac resynchronization device).
13) Presence of an implantable loop recorder.
14) History of left atrial appendage ligation, removal, or occlusion.
15) Subjects with known allergies or hypersensitivities to adhesives or hydrogel.
16) Inability to provide written informed consent.

17) Current or anticipated participation in another randomized controlled trial in which the interventional drug or device is known to affect the incidence of the primary or secondary outcomes of this study.

4.3 Women of childbearing potential

Women of childbearing potential (WOCBP) are excluded from the SEARCH-AF trial. A WOCBP is defined as any female who has experienced menarche and who has not undergone surgical sterilization (hysterectomy or bilateral oophorectomy) and is not postmenopausal. Menopause is defined as 12 months of amenorrhea in a woman over age 45 years in the absence of other biological or physiological causes. In addition, females under the age of 55 years must have a serum follicle stimulating hormone, (FSH) level > 40 mIU/mL to confirm menopause. *Females treated with hormone replacement therapy, (HRT) are likely to have artificially suppressed FSH levels and may require a washout period in order to obtain a physiologic FSH level. The duration of the washout period is a function of the type of HRT used. The duration of the washout period below are suggested guidelines and the investigators should use their judgement in checking serum FSH levels. If the serum FSH level is >40 mIU/ml at any time during the washout period, the woman can be considered postmenopausal:

- 1 week minimum for vaginal hormonal products (rings, creams, gels).
- 4 week minimum for transdermal products.
- 8 week minimum for oral products.

Other parenteral products may require washout periods as long as 6 months.

4.4 Discontinuation of Subjects from Trial Participation

Subjects must discontinue use of the investigational device for any of the following reasons:

- Withdrawal of informed consent (subject’s decision to withdraw for any reason).
- Any clinical adverse event, laboratory abnormality, or intercurrent illness which, in the opinion of the investigator, indicates that continued use of the device is not in the best interest of the subject.
- Loss of ability to freely provide consent through imprisonment or involuntary incarceration for treatment of either a psychiatric or physical (eg, infectious disease) illness.

All subjects who discontinue use of the device for any reason will be asked to continue to be followed until the final study visit, if they provide consent to do so.. If a subject declines to continue to participate in the study the reason for withdrawal must be documented appropriately.

All subjects are free to withdraw from participation in this study at any time, for any reason, specified or unspecified, and without penalty or loss of benefits to which the subject is otherwise entitled. Study patients will be informed of the possibility to withdraw consent without giving any reason. Subjects may be withdrawn for specific reasons during the study, which include: ineligibility, non-compliance or for administrative reasons (including study closure).

Before a subject is declared lost to follow-up, all efforts should have been made to contact the participant for a final assessment.

5. Study Design

5.1 Study Description

SEARCH-AF is a multicenter, parallel group (2-arm), unblinded randomized controlled trial (RCT). It will compare a strategy of enhanced cardiac rhythm monitoring to usual care in post-cardiac surgery subjects during sub-acute, post-operative period. Half of the study population will be randomly allocated to a wearable adhesive cardiac rhythm monitoring system for up to 30 days (intervention group) and the other half will be randomly allocated to usual care (control group) in which no continuous cardiac rhythm monitoring will be mandated during the first 30 days after randomization. The primary endpoint will be a cumulative atrial fibrillation/flutter (AF/AFL) burden of ≥6 minutes or documentation of AF/AFL with a 12-lead electrocardiogram (ECG). In addition, all subjects will also receive 14 days of continuous cardiac rhythm monitoring at 6±1 months after surgery. Clinical endpoints will be adjudicated by an independent Clinical Events Committee.

5.2 Executive Committee

An academic Executive Committee, led by co-chairs Dr. Andrew Ha, Dr. Atul Verma, Dr. C. David Mazer, Dr. Bobby Yanagawa, and Dr. Subodh Verma participated in protocol development. This group will provide ongoing scientific and operational oversight to the study.

The Executive Committee will provide suggestions for potential investigators and site coordinators, monitor progress of study enrollment, make recommendations about the conduct of the study, and oversee the presentation and publication of the trial results. The Executive Committee will include clinical experts from specialties involved in the clinical care of cardiac surgical subjects with AF (cardiology, cardiac electrophysiology, cardiac surgery, cardiac anesthesiology) and who are experienced in clinical trial methodologies.

5.3 Clinical Events Committee (CEC)

The Clinical Events Committee, composed of experts in the relevant fields, will review reported study clinical outcomes (arrhythmia, MACE events, and bleeding) to provide consistency and validity in the assessment of these outcomes. They will be blinded to treatment assignment when adjudicating such outcomes. In addition, this committee will be responsible for over-reading all rhythm strips, ECGs, full-disclosure Holter results, and full disclosure recordings from the wearable adhesive cardiac rhythm monitoring system (if necessary) to ascertain that AF/AFL was correctly diagnosed. A chairperson of the CEC will be nominated.

5.4 Baseline Visit

The baseline visit will occur at a time during hospitalization when the subject is clinically stable after his/her index cardiac surgery, according to the treating cardiac surgeon. During this visit, the following information will be collected:

Clinical information: demographics, medical history (including hypertension, diabetes, heart failure or LV dysfunction, previous history of stroke, TIA, or systemic embolism, renal disease,

sleep apnea, peripheral vascular disease, COPD, alcohol use, smoking history), CHA_2DS_2-VASC score, HAS-BLED score, concomitant medications, and details pertaining to cardiac surgery.

Physical examination information: Height, weight, blood pressure, pulse rate.

Laboratory or imaging testing: Pre-operative and post-operative ECG, echocardiography (performed within 12 months from enrollment), complete blood count, creatinine, electrolytes, INR.

5.5 Randomization

After study eligibility has been confirmed and the baseline visit assessment is completed, subjects will be randomly allocated in a 1:1 ratio to the intervention arm (up to 30 days of cardiac rhythm monitoring with a wearable adhesive cardiac rhythm monitoring system, starting on the day of randomization) or to the control group (usual care). Randomization lists will be generated by computer and employ random permuted blocks. Randomization will be stratified by centre and according to the type of cardiac surgery performed: (i) isolated CABG or (ii) valve replacement or repair +/- CABG. Randomization can occur at any time between the third post-operative day and discharge.

Stratification by surgery type is felt to be important for this trial for the following reasons. First, in the published literature, subjects who undergo valve replacement or repair were more likely to develop POAF with reported incidences of up to 60% during hospitalization [7,9,16,19] . These rates are higher than those who undergo isolated CABG, in which the highest rates are typically in the range of ≈40% [6-20]. Second, the case volume ratio of isolated CABG to valve surgery (±CABG) is in the range of 4:1. Therefore, stratification will provide balance to the proportion of subjects with isolated CABG vs. valve surgery ± CABG in both study arms.

If a subject is randomized to the intervention group, the device will be fitted onto the subject on within 24 hours of randomization. If the subject is randomized to usual care, no formal continuous cardiac rhythm monitoring will be mandated during the first 30 days after randomization. However, subjects in the usual care group may undergo rhythm assessment (e.g. ECG, Holter monitoring, event monitoring) if there is a clinical indication to do so, as per the discretion of their treating physicians.

5.6 Proposed Study Intervention

Subjects will be randomly allocated to one the following two arms: (i) Continuous cardiac rhythm monitoring for up to 30 days after randomization (intervention group) or (ii) Usual care (control group). In addition, all subjects will also receive 14 days of continuous cardiac rhythm monitoring at 6±1 months after surgery.

5.6.1 Continuous cardiac rhythm monitor early after surgery (Intervention group)

On the day of randomization, subjects in this group will receive 30 days of continuous cardiac rhythm monitoring with an adhesive monitor. From the start of the study to September 302018, the device to be used was the Medtronic SEEQ™ mobile cardiac telemetry system *(appendix A-D)*. Starting on October 1 2018, the CardioStat® system will be used for subjects who initiate protocol-mandated rhythm monitoring in this study (appendix E-F).

In May 2018, the SEARCH-AF team was informed by Medtronic that the SEEQ™ mobile cardiac telemetry system would no longer be available for research purposes as of September 30 2018. As a result, an alternative wearable adhesive cardiac rhythm monitoring system (CardioStat®, manufactured by Icentia Inc., Quebec, Canada) was chosen to replace the SEEQ™ monitoring system for all subsequent protocol-mandated cardiac rhythm monitoring events.

Wearable sensor
The SEEQ™ system consists of a wearable sensor which is a single-lead, low-profile, peel-and-stick device applied over the anterior left chest wall of the subject. It is water-resistant, which is a major difference than other forms of prolonged cardiac rhythm monitoring modalities such as the 14-day Holter monitor. The wearable patch is designed for one-time use only and cannot be re-applied if removed. Each sensor provides up to 7.5 days of monitoring. Therefore, the sensor will be applied on 4 separate occasions if 30 days of rhythm monitoring are required.

Starting on October 1 2018, the CardioStat® cardiac rhythm monitoring system (Icentia Inc., Quebec, Canada) was selected to replace the SEEQ™ system for all subsequent cardiac rhythm monitoring events as mandated by the protocol. The CardioStat® device is a wearable adhesive cardiac rhythm monitoring system which is low-profile, water-resistant, and is adhered onto the skin surface with 2 electrodes. This wearable adhesive device is designed for one-time use only. Each CardioStat® device will provide 14 days of cardiac rhythm monitoring. For subjects randomized to the intervention group who will be monitored by the CardioStat® device, they will receive 28 days of continuous cardiac rhythm monitoring.

Data transmitter
The data transmitter is required to facilitate detected transmission of the detected arrhythmia episodes via a cellular network to a central data processing centre from Medtronic. Access to the cellular network is free of charge for the research participants and internet access is not required. The subject needs to be within 30 feet from the transmitter in order for the detected episodes to be sent wirelessly. In addition, the data transmitter is battery-operated and requires daily charging. The data transmitter also has a self-activated button which allows for transmission of subject-activated episodes to provide rhythm-symptom correlation.

For subjects who undergo cardiac rhythm monitoring with the CardioStat® monitoring system, no transmitter will be required since all cardiac rhythm monitoring will be stored on the device itself.

Data reporting
<u>Medtronic SEEQ™ mobile cardiac telemetry system</u>
All auto-detected and subject-triggered episodes which are successfully transmitted will be sent to a central Medtronic monitoring centre. Selection of rhythm strips for inclusion in the summary report is governed by a pre-specified set of rhythm/rate-based parameters *(appendix C)*. The report includes a number of arrhythmic diagnoses, including episodes of new-onset AF or AFL irrespective of the ventricular rate. A summary report with accompanying rhythm strips will be sent to the treating physician and the trial coordination centre on a weekly basis as well as after the conclusion of the 30-day or 14-day monitoring period.

The transmitted data will be anonymized as each research participant in SEARCH-AF will be identified by a unique study code which does not contain any personal identifying information. If the subject is re-hospitalized or assessed in an ambulatory clinic during the study period, all efforts will be made to obtain 12-lead ECGs or cardiac telemetry strips that were performed during those clinical visits.

Currently, the Medtronic SEEQ™ mobile cardiac telemetry system is not approved for clinical use in Canada. Approval for Investigational Testing (Medical Devices) from Health Canada will be obtained. If this device is approved for clinical use in Canada during the course of the trial, investigational sites will notify their REBs of this change.

<u>CardioStat® cardiac rhythm monitoring system</u>
The cardiac rhythm data will be collected by the CardioStat® device and the data will not be wirelessly transmitted. Once the subject completes the monitoring period, the device will be mailed back to Icentia Inc. (Quebec, Canada) who will process the device and retrieve the rhythm data. Trained personnel will then examine the raw cardiac rhythm data and provide information on the presence of AF or AFL, the duration of each AF/AFL episode, as well as the total duration of AF/AFL during the monitoring period. A list of reportable cardiac rhythm parameters is shown in Appendix E-F. Once the cardiac rhythm data is analyzed, a summary report will be generated. This can be accessed by the investigators via a web-based, password-protected interface. Subjects will be identified by a unique study number which does not contain any personal identification information. The CardioStat® cardiac rhythm monitoring device is approved for clinical use in Canada.

Patient Education
<u>Medtronic SEEQ™ mobile cardiac telemetry system:</u>
Given that a new adhesive sensor needs to be applied every 7.5 days, each subject in the intervention group will need to remove and re-apply a new sensor on 3 additional occasions. The first application will be done during hospitalization, under the supervision of the research coordinator. During the first sensor application, the subject will be educated on the appropriate use of this device, including the following:

1) Proper placement of the adhesive sensor.
2) Use and maintenance of the data transmitter.
3) Self-activation of the sensor for symptomatic episodes.

For subjects randomized to the intervention group, the research coordinator will contact them on a weekly basis by telephone to reinforce education of the proper use of the device (if necessary). In addition, adverse outcomes related to the use of the study interventional device will be assessed and an in-person clinic visit may be arranged if required.

Studies of the SEEQ™ mobile cardiac telemetry system had shown high levels of patient compliance and satisfaction with this device. In these studies, 98% of patients were able to properly apply the SEEQ™ adhesive sensor, patient compliance throughout the prescribed monitoring period was reported to be 90%, and 85% of patients were satisfied with the SEEQ™

mobile cardiac telemetry system. (http://www.medtronicdiagnostics.com/us/cardiac-monitors/seeq-mct-system/evidence/index.htm, accessed July 31 2015)

CardioStat® cardiac rhythm monitoring system
Each wearable sensor will provide 14 days of continuous cardiac rhythm monitoring. As such, subjects who are randomized to the intervention arm will receive 2 CardioStat® devices. The first application will be done during hospitalization, under the supervision of the research coordinator. During the first sensor application, the subject will be educated on the appropriate use of this device, including the following:

1) Proper placement of the adhesive sensor.
2) Self-activation of the sensor for symptomatic episodes.

For subjects randomized to the intervention group, the research coordinator will contact them after 2 weeks by telephone to reinforce education of the proper use of the device (if necessary). Adverse outcomes related to the use of the study device will be assessed and an in-person clinic visit may be arranged if required.

5.6.2 Usual Care
Subjects randomized to the usual care arm will be discharged from hospital without protocol-mandated continuous cardiac rhythm monitoring within the first 30 days after randomization. This is consistent with the practice of the participating surgical sites in SEARCH-AF and the overall Canadian/global cardiac surgical community at large. Presently, there are no clinical guidelines or recommendations to inform clinicians on how to monitor for POAF/AFL in the cardiac surgical population during the sub-acute, post-discharge phase, particularly within the first month after discharge. The 2010 Canadian Cardiovascular Society AF guidelines recommend that patients be reviewed at 6-12 weeks after surgery if they are treated with medications for their POAF/AFL [31]. However, the specific cardiac monitoring strategy is not described. The most recent iterations of the ACC/AHA/HRS or ESC AF guidelines do not contain specific recommendations on the detection or follow-up care of cardiac surgical patients with POAF/AFL after discharge [32,33].

However, subjects in the control group may undergo rhythm monitoring during the study period if their treating physicians deem that there is a clinical indication to so do. Typical rhythm monitoring modalities such as a 12-lead ECG, Holter monitoring, or event monitoring may be prescribed on clinical grounds. The test reports will be obtained along with full disclosure of rhythm strips. These will be independently overread by the CEC. If the subject is re-hospitalized or has an unscheduled medical visit during the first 30 days after randomization (the study period during which the primary endpoint will be evaluated), all cardiac rhythm tests which are performed will be obtained and overread.

5.6.3 Additional 14-day cardiac rhythm monitoring at 6±1 months
At the 6±1 month follow-up visit, all subjects (in the intervention group and the usual care group) will undergo 14 days of continuous cardiac rhythm monitoring with a wearable adhesive cardiac rhythm monitoring device. This will involve application of 2 SEEQ™ monitors over the

14-day monitoring period. As of October 1 2018, the CardioStat® device will replace the SEEQ™ mobile cardiac telemetry system. **5.7 Methods to Protect against Potential Bias**
We have identified several potential sources of bias in this trial and have proposed methods to minimize them:

Non-blinded study design: SEARCH-AF is an open-label RCT since subjects and clinicians will be aware of the treatment assignment. Atrial arrhythmias detected by the SEEQ™ system will be initially interpreted by trained technicians in Medtronic's reading centre who are not involved in any part of the design and are unaware of the study design. Atrial arrhythmias detected by the CardioStat® cardiac rhythm monitoring system will be initially interpreted by trained technicians employed by Icentia Inc. who are not involved in any part of the study design. Adhering to standardized protocols, they are trained to interpret and report whether AF/AFL is present or absent and the duration of these episodes. We do not expect Medtronic or Icentia Inc. will play any role in biasing the results away from the null by preferentially "over-calling" AF/AFL episodes for participants in this study. In addition, full disclosure of the entire monitoring period can be retrieved. The generated rhythm strips and reports will then be sent to an independent group of adjudicators consisting of cardiologists and cardiac electrophysiologists. These adjudicators will over-read the reports with the attached rhythm strips and verify if the diagnosis is indeed correct. If needed, they will be able access full disclosure rhythm strips for further review. Additional rhythm monitoring with Holter, 12-ECG, in-patient telemetry strips, and cardiac event monitors will only be done on clinical grounds (ie: if deemed necessary by the treating physicians). Patient identification information will be removed and sent to the adjudicators for review, who will be blinded to the randomization status of the subjects. We believe these measures will minimize/mitigate potential bias due to the open-label design of SEARCH-AF.

Detection bias: It can be argued that since no continuous cardiac rhythm monitoring is mandated in the control arm during the first 30 days after randomization, the intervention arm will be favored due to the fact that it will be exposed to a more intense cardiac rhythm monitoring strategy, consequently allowing for a greater number of POAF/AFL episodes to be detected. While this is intuitively correct, the purpose of this study is to demonstrate that enhanced monitoring can substantially improve the rate POAF/AFL detection over usual care, by at least 3-fold, within the first month after surgery. This trial will be powered to detect an absolute difference of 7% in POAF/AFL detection between the two arms (based on an assumed event rate of 3% in the control group). The magnitude of this difference (3-fold increase or 7% absolute difference) is felt to be clinically relevant by the Steering Committee of SEARCH-AF, which consists of cardiac electrophysiologists who specialize in the care of AF patients, a neurologist who specialize in AF-related stroke care, cardiac surgeons and a cardiac anesthesiologist who manage this post-operative issue on a daily basis.

Misclassification bias: All forms of ambulatory cardiac rhythm monitoring are subject to artifact (e.g. motion artifact) which may prohibit accurate rhythm diagnosis. The presence of artifact may result in either under- or over-diagnosis of AF/AFL. To minimize this potential source of bias, a standard operating procedure manual for event adjudication will be developed. All detected AF/AFL episodes will be confirmed by the CEC. If there is disagreement in the

diagnosis of the detected AF/AFL episode, it will be reviewed by the chair of the adjudication committee for determination of the final rhythm diagnosis.

5.8 Proposed Duration of the Intervention Period

There will be two cardiac rhythm monitoring periods. For subjects randomized to the intervention group (enhanced cardiac rhythm monitoring), they will undergo up to 30 days of continuous cardiac rhythm monitoring with a wearable adhesive device starting on the day of randomization. At 6±1 months after discharge from the index cardiac surgery, they will undergo another 14 days of cardiac rhythm monitoring with a wearable adhesive device.

Subjects in the usual care group (control group) will only undergo protocol-mandated cardiac rhythm monitoring at 6±1 months after discharge. At that time, they will undergo 14 days of cardiac rhythm monitoring a wearable adhesive cardiac rhythm monitoring device.

5.9 Proposed Frequency and Duration of Follow-up

Visit #1 (31-90 days after discharge): All subjects will be assessed during follow-up at 31-90 days after discharge from their index cardiac surgery. Details of MACE and major bleeding events which occurred after hospital discharge will be recorded. As well, subjects' list of medications will be updated. Specifically, a detailed history of OAC use will be obtained, including the date of initiation, prescriber, dose, and type.

In this visit, subjects in the intervention group will return their data transmitters, have their wearable sensors removed (if still worn by the participant), and undergo a 12-lead ECG. Subjects in the control group will undergo a 12-lead ECG during this follow-up visit as well.

Visit #2 (6±1 months after surgery): Subjects will be assessed during a second follow-up visit at 6±1 months after surgery. At this visit, all subjects will receive 14 days of continuous cardiac rhythm monitoring with a wearable adhesive cardiac monitoring device. All subjects will be educated on the proper use of the device. Details of MACE and bleeding events which occurred since the first follow-up visit will be recorded. As well, subjects' list of medications will be updated. A detailed history of OAC use will be obtained, including the date of initiation, prescriber, dose, type. At this visit, all subjects will undergo a 12-lead ECG.

Visit #3 (9±1 months after surgery): This visit will be conducted by telephone. At this visit, a history of OAC use will be obtained (type, dose, prescriber).

5.10 Study flowchart

Evaluation	*Screening/Baseline*	*Randomization (between the 3rd post-operative day and discharge)*	*Visit #1 (31-90 days after discharge)*	*Visit #2 (6±1 months after surgery)*	*Visit #3 (telephone-based) (9±1 months after surgery)*
**Inclusion/exclusion criteria**	**X**				
**Medical history**	**X**		**X**	**X**	
**Informed consent**	**X**				
**Physical exam**	**X**		**X**	**X**	
**Blood pressure + Heart Rate**	**X**		**X**	**X**	
**Medication review**	**X**		**X**	**X**	**X**
**12-lead ECG**	**X**		**X**	**X**	
**Randomization**		**X**			
**Education of device application (and maintenance of data transmitter for subjects allocated to the SEEQ™ device)**		**X *(intervention group only)***		**X *(all subjects)***	
**Application of the wearable adhesive cardiac rhythm monitoring device**		**X *(intervention group only)***		**X *(all subjects)***	
**Primary and/or secondary outcomes**			**X**	**X**	**X**
**SAE**	**X***	**X**	**X**	**X**	**X**

**SAE collection begins after consent is signed*

5.11 Primary Outcome

The primary endpoint of this trial is documentation of AF/AFL, defined as a cumulative AF/AFL duration of ≥6 minutes or documentation of AF/AFL by a 12-lead ECG within 30 days after randomization. Clinical endpoints will be adjudicated by an independent clinical events committee.

5.12 Secondary Outcomes

1) AF/AFL lasting for ≥24 hours during 30 days after randomization and between day 31 and the last follow-up date.
2) Cumulative AF/AFL burden during the 14-day monitoring period (recorded by the wearable adhesive cardiac monitoring device) between day 31 and the last follow-up date.
3) Proportion of subjects who are prescribed with oral anticoagulation at the following timepoints: (i) 45 days after discharge from cardiac surgery; (ii) between day 46 and the last follow-up date.
4) Number of days during which the wearable adhesive cardiac rhythm monitor sensor was worn by subjects.
5) Reasons for premature removal of the wearable adhesive cardiac rhythm monitor by subjects.
6) Incidence of adverse events related to use of the wearable adhesive cardiac rhythm monitor.
7) Performance of non-protocol mandated Holter monitoring and/or event recorders during the 30-day period after randomization (for subjects in both groups).
8) Major adverse cardiac outcomes occurring within 45 days after hospital discharge, including the following: all-cause death, myocardial infarction, ischemic stroke, non-CNS systemic embolism (please refer to appendix H for endpoint definitions).
9) Proportion of subjects who experienced major bleeding events (please refer to appendix I for definitions of bleeding outcomes) within 45 days after discharge from cardiac surgery.

5.13 Prescription of Oral Anticoagulation

It is possible that greater rates of AF/AFL detection in the early post-operative period may lead to increased prescription of oral anticoagulation by the subjects' treating physicians. The rates of OAC use between the intervention and control groups at 45 days, 6±1 months, and 9±1 months after discharge from cardiac surgery will be recorded as secondary outcomes. In addition, the type (and dosing if applicable) of OAC used will be collected. The choice of OAC that is prescribed will be at the discretion of the subjects' treating physicians and is not mandated by protocol.

In SEARCH-AF, the decision to initiate oral anticoagulation is not mandated by protocol but rather is left at the discretion of the subjects' treating physicians. The decision to treat a subject with OAC for AF-related stroke prevention after cardiac surgery is influenced by a number factors including: (i) the subject's risk of stroke, (ii) the subject's risk of bleeding in the early post-operative phase, (iii) the subject's AF burden, (iv) the subject's treatment preferences. The relative weights of these factors will vary from subject to subject. Therefore, the decision to initiate OAC in this patient population is individualized.

At the current moment, there is no high-quality data in inform clinicians on the specific AF burden upon which OAC use will be beneficial in the post-cardiac surgical population. In the 2010 CCS AF guidelines, there was a conditional recommendation of low-quality evidence suggesting the use of OAC for post-cardiac surgical patients with continuous AF of >72 hours [31]. Specifically, it emphasized an "individualized assessment of the risks of a thromboembolic

event and the risk of postoperative bleeding" [31]. In the 2010 ESC AF guidelines, initiation of OAC is recommended for post-cardiac surgical patients with AF duration >48 hours in the absence of contraindications to OAC use (Class IIa recommendation, level of evidence "A") [32]. In the 2014 AHA/ACC/HRS AF practice guidelines, antithrombotic treatment for post-cardiac surgical patients who developed AF was considered to be "reasonable…as advised for non-surgical patients" (Class IIa recommendation, level of evidence "B") [33]. In the American guidelines, there was no mention of a specific AF duration threshold which will trigger prescription of OAC therapy. This highlights the existing variability in clinical practice as it pertains to the use of OAC for post-cardiac surgical patients who develop AF.

Each participating site in SEARCH-AF will be led by a cardiac surgeon and/or a cardiologist/cardiac electrophysiologist. We will encourage dialogue amongst the subject's treating physician, the subject's cardiac surgeon, and the local site PIs in the decision-making process of OAC use for subjects in SEARCH-AF. The local site PIs will provide consultative advice on whether a given subject should be considered for OAC use, after accounting for his/her risk of stroke, risk of bleeding, and AF burden. Ultimately, the final decision to initiate OAC for subjects in this trial will be at the discretion of their treating physicians.

5.14 Other Considerations

Loss to follow-up: Given that the primary endpoint will be assessed at a short time course after randomization (30 days), we anticipate the rate of loss to follow-up to be low (<1%). In addition, all efforts will be made to ensure that subjects are assessed at follow-up at 31-90 days after discharge from their index cardiac surgery.

Non-compliance: It is possible that subjects may stop wearing the wearable adhesive cardiac rhythm monitoring device given that a new sensor needs to be applied every week (for the SEEQ™ device) and every 2 weeks (for the CardioStat® device) for a maximum monitoring period of up to 30 days To minimize this, research coordinators will contact (telephone or email) subjects on a weekly (for SEEQ™) or biweekly (for CardioStat®) basis during the study period to ensure compliance with enhanced cardiac rhythm monitoring. The monitoring centre will also alert the research team if they do not receive transmitted data for a period of more than 48 hours. Previous studies had reported high compliance rates of wearing the SEEQ™ monitor for the prescribed monitoring period [36,37].

Crossover: Subjects randomized to the control group will be unlikely to crossover to the intervention group, since the costs of the adhesive system employed in this trial are not covered by government health plans and this device is not routinely used in clinical practice, and the SEEQ ™ device is not currently approved for use in Canada. On the other hand, it is possible for subjects in the intervention group to be crossed over to usual care if, for some reason, they no longer wear the adhesive monitor. However, we anticipate the rate of non-compliance to be low. If this unlikely situation is to occur, we do not anticipate that the results will significantly bias away from the null because (i) the anticipated occurrence rate of crossover is low (<1%), (ii) subjects will be crossed over to usual care in which the intensity of rhythm monitoring is less; (iii) analyses will be performed with the intent-to-treat principle.

6. Adverse events

6.1 Device Problem Reporting

Subject to section 59 of the Medical Device Regulations in Canada, any incident that comes to the attention of the investigator which meets the following conditions must be reported to the study sponsor within 24 hours of becoming aware of the incident:

(1) Incident is related to a failure of the device or a deterioration in its effectiveness, or any inadequacy in its labelling or in the directions for use;

AND

(2) Has led to the death or a serious deterioration in the state of health of a patient, user or other person, or could do so were it to recur.

In such a situation, the investigator must complete the SEARCH-AF Incident Report and submit the report to the coordinating centre within 1 business day of becoming aware of the incident. The following information will be required: date of the incident, details of the incident, course of action taken, and other relevant details.

Follow-up reports must be provided to the coordinating centre as any new information becomes available. The coordinating centre or device manufacturer will report these incidents to the Medical Devices Bureau of Health Canada within the timelines specified in section 60 of the regulations.

6.2 Serious adverse event collecting and reporting

Please refer to appendix J for details pertaining to serious adverse event (SAE) collection and reporting.

7. Study duration

7.1 Expected study duration of subject participation

The anticipated duration of subject participation will be 9 months. The first follow-up visit will occur at 31-90 days after discharge from cardiac surgery and the second follow-up visit will occur at 6±1 months after discharge from cardiac surgery. A final telephone-based follow-up will occur at 9±1 months after discharge from cardiac surgery.

7.2 Expected total study duration

We planned for a recruitment period of 36 months. The last subject enrolled will be followed up at 9 months after surgery, extending the total study duration to 45 months.

8. Concomitant Medications/Natural Remedies/Foods

There are no restrictions placed on medication use in this study. Prescription medications will be recorded on the concomitant medication form in case report forms at baseline and follow-up visits.

9. Prohibited Medications and Procedures

There are no restrictions placed on medication use or procedures mandated by this study. Subjects will continue to receive all usual medications, rehabilitation, procedures and interventions as prescribed or recommended by his/her health care providers.

10. Study Evaluations/Procedures

10.1 Laboratory Evaluations and Specimen Collection

Not applicable.

10.2 Questionnaires

Not applicable.

11. Trial Management

11.1 Study coordinating centre

The day-to-day management of the trial will be supported by the Applied Health Research Center (AHRC). AHRC is a clinical research methods and services group at the Li Ka Shing Knowledge Institute of St. Michael's Hospital (Toronto, Canada). The AHRC is a comprehensive Academic Research Organization (ARO) affiliated with the University of Toronto. The AHRC has experience in managing more than 50 multi-site, national and international clinical trials and observational studies, and has grown to be one of the largest academic research organizations in Canada. The AHRC will work closely with the study sponsor and Steering Committee to manage this clinical trial, including protocol finalization, site section and management, training personnel, collecting and managing trial documents and assisting with ethics submissions. AHRC will coordinate data management activities and will be responsible for developing electronic case report forms, performing data validation activities, providing data outputs and performing statistical analyses. AHRC employs industry-leading web-based secure database technology which incorporates advanced data validation and reporting tools, and allow for faster access to trial data than traditional paper-based data collection tools. The electronic database has built-in web-based study randomization tools and can link with other data systems (e.g. laboratory information systems) to facilitate automatic upload of clinical data to minimize manual entry. The AHRC is using electronic data capture software to manage data for over 25 studies, including more than 15 CIHR-funded multi-centre trials. AHRC was the data coordinating and management centre for the EMBRACE trial [27].

11.2 Compliance with the Study Protocol

The study shall be conducted as described in this approved protocol. All revisions to the protocol must be discussed with, and be prepared by, the Study Sponsor and the coordinating (St. Michael's Hospital and the Applied Health Research Centre which is affiliated with St. Michael's Hospital). The investigator should not implement any deviation or change to the protocol without prior review and documented approval/favorable opinion from the REB/IRB of an amendment, except where necessary to eliminate an immediate hazard(s) to study subjects.

If a deviation or change to a protocol is implemented to eliminate an immediate hazard(s) prior to obtaining IRB approval/favorable opinion, as soon as possible the deviation or change will be submitted to:

- REB/IRB for review and approval/favorable opinion
- Study Sponsor.
- Regulatory Authority(ies), if required by local regulations

Documentation of approval signed by the chairperson or designee of the REB/IRB(s) must be sent to the study sponsor.

If an amendment substantially alters the study design or increases the potential risk to the subject: (1) the consent form must be revised and submitted to the REB/IRB(s) for review and approval/favorable opinion; (2) the revised form must be used to obtain consent from subjects currently enrolled in the study if they are affected by the amendment; and (3) the new form must be used to obtain consent from new subjects prior to enrollment.

If the revision is an administrative letter, investigators must inform their REB/IRB(s).

12. Statistical Considerations

12.1 Sample Size Calculation

In this trial, the primary endpoint is the occurrence of sustained POAF/AFL, defined as a cumulative burden of ≥6 minutes or documentation with a 12-lead ECG. Based on the existing literature, the rate of POAF/AFL in the sub-acute, post-operative phase (within 1 month after surgery) was reported to be 2-5% [21-24]. For this trial, we assume that the rate of AF/AFL detection in the usual care group (control group) to be 3% during the first 30 days after randomization. We seek to detect at least a 3-fold or a 7% absolute increase in the rate of POAF/AFL detection with enhanced cardiac rhythm monitoring. As such, we assume a POAF/AFL detection rate of 10% in the intervention group. A total of 388 subjects (n=194 in each arm) will be required to detect this difference with 80% power at a 2-sided alpha of 0.05. Assuming a 2% attrition rate (death, loss to follow-up, withdrawal of consent to participate), the final sample size will be **396** (n=198 in each arm).

The Steering Committee of SEARCH-AF has determined that the magnitude of the difference which this trial seeks to detect (a 3-fold or an absolute 7% increase in the rate of AF/AFL detection when compared to usual care) is clinically meaningful for the population of interest.

Randomized controlled trials evaluating the efficacy of enhanced AF/AFL detection in the cryptogenic stroke population had demonstrated comparable magnitudes in differences of the primary endpoint between the treatment and control groups. In EMBRACE, the rate of AF/AFL detection was 5 times greater (absolute difference in the rate of AF/AFL detection: 12.9%) in the enhanced monitoring group when compared to the control group over a study period of 90 days [27]. In CRYSTAL-AF, the rate of AF/AFL detection was 6 times greater than the control group by 6 months (absolute difference in the rate of AF/AFL detection: 7.5%) [28]. In both studies, an AF/AFL episode lasting ≥30 seconds was the primary endpoint. As such, we feel that the magnitude of difference which we seek to detect in SEARCH-AF is realistic and achievable.

12.2 Details of Statistical Analysis

Descriptive statistics will be computed. For continuous variables, mean ± standard deviation will be reported or median and interquartile range as appropriate. For categorical variables, counts ± percentages will be reported.

The primary analysis will be conducted with the intent-to-treat principle. The primary outcome will be displayed in a 2 x 2 contingency table, sorted according to randomization status (intervention/control group) and the presence/absence of the primary endpoint. A contingency table stratified by the surgery type stratification factor will also be examined for homogeneity of treatment effect. The number of AF/AFL episodes will be compared by means of a Poisson regression model (or possibly zero-inflated Poisson if indicated) and the treatment effect expressed as a rate ratio with 95% CI. The proportion of patients experiencing AF/AFL lasting for 24 hours or more will be compared with a Fisher's Exact test (because numbers of events and therefore expected cell counts are expected to be low). The treatment effect will be expressed as a difference in proportions with 95% CI. The proportions of patients prescribed with oral anticoagulants will be analyzed similarly.

Since outcomes related to the device itself do not have a comparison group, they will be analyzed using only descriptive statistics (means, proportions, etc.). All remaining secondary outcomes are binary and will be compared with a Fisher's Exact test (assuming the assumptions for the chi-square test are not met) and the treatment effects will be expressed as risk differences with 95% CIs. Given the expected very low attrition rate of 2%, any missing data resulting from attrition at that rate will be insufficient to substantially alter conclusions. Nevertheless some sensitivity analyses will be considered, especially given anticipated low event rates.

Logistic regression will be performed to obtain adjusted estimates of the treatment effect if feasible. However, given that the anticipated absolute event rates will be relatively low which will result in a small number of subjects having the primary endpoint, the choice of covariate inclusion will be carefully considered. Candidate covariates which may be included in the logistic regression model include: age, sex, heart failure/LV dysfunction, diabetes, hypertension, history of ischemic stroke, renal disease, and COPD. Selection of these covariates is based on prior knowledge and standards from published literature. Secondary outcomes will be similarly analyzed after adjusting for these variables.

In subjects in both arms, the timing of the occurrence of the first AF/AFL episode (e.g. day 1, day 2, day 15, etc.) during the 30-day period after study randomization will be documented. Survival analysis will be used to determine the time (in days) to the first recorded POAF/AFL event and displayed with a Kaplan-Meier curve. This analysis is undertaken to assess the optimal duration of continuous cardiac rhythm monitoring to capture POAF/AFL in the post-discharge period (e.g. 1, 2, 3 or 4 weeks after discharge from cardiac surgery).

If this study is able to demonstrate the pre-specified outcome difference (3-fold or 7% absolute difference in the rate of AF/AFL detection) between the two arms, then a separate economic analysis will be undertaken to evaluate the cost-effectiveness of the study intervention.

12.3 Subgroup Analysis

Isolated CABG vs. Valve replacement/repair ± CABG: A pre-specified sub-analysis for subjects who underwent isolated CABG or valve surgery ± CABG is planned. In published reports, patients who underwent valve surgery had higher rates of in-hospital POAF when compared to those who underwent isolated CABG. This analysis will provide insight on the rates of AF/AFL detection between these 2 major types of cardiac surgery in the subacute, post-discharge period. Typically, patients who undergo bioprosthetic valve replacement (the most common being aortic valve replacement) are not routinely treated with warfarin in the post-operative period unless there is another compelling reason to do so, such as the presence of AF/AFL. If high rates of AF/AFL are detected in the valve surgical subset during the sub-acute post-discharge period, this may have relevant clinical implications on oral anticoagulation use for this subset of subjects.

13. References

1. Selnes OA, Goldsborough MA, Borowicz LM, McKhann GM. Neurobehavioural sequelae of cardiopulmonary bypass. *Lancet*. 1999;353:1601-1606.

2. Whitlock R, Healey JS, Connolly SJ, Wang J, Danter MR, Tu JV, Novick R, Fremes S, Teoh K, Khera V, Yusuf S. Predictors of early and late stroke following cardiac surgery. *CMAJ*. 2014;186:905-911.

3. Kaw R, Hernandez AV, Masood I, Gillinov AM, Saliba W, Blackstone EH.Short- and long-term mortality associated with new-onset atrial fibrillation after coronary artery bypass grafting: a systematic review and meta-analysis. *J Thorac Cardiovasc Surg*. 2011;141:1305-1312.

4. Gialdini G, Nearing K, Bhave PD, Bonuccelli U, Iadecola C, Healey JS, Kamel H. Perioperative atrial fibrillation and the long-term risk of ischemic stroke. *JAMA*. 2014;312:616-622.

5. Lee SH, Kang DR, Uhm JS, Shim J, Sung JH, Kim JY, Pak HN, Lee MH, Joung B. New-onset atrial fibrillation predicts long-term newly developed atrial fibrillation after coronary artery bypass graft. *Am Heart J*. 2014;167:593-600.

6. Villareal RP, Hariharan R, Liu BC, Kar B, Lee VV, Elayda M, Lopez JA, Rasekh A, Wilson JM, Massumi A. Postoperative atrial fibrillation and mortality after coronary artery bypass surgery. *J Am Coll Cardiol*. 2004;43:742-748.

7. Creswell LL, Schuessler RB, Rosenbloom M, Cox JL. Hazards of postoperative atrial arrhythmias. *Ann Thorac Surg*. 1993;56:539-549.

8. Aranki SF, Shaw DP, Adams DH, Rizzo RJ, Couper GS, VanderVliet M, Collins JJ Jr, Cohn LH, Burstin HR. Predictors of atrial fibrillation after coronary artery surgery. Current trends and impact on hospital resources. *Circulation*. 1996;94:390-397.

9. Almassi GH, Schowalter T, Nicolosi AC, Aggarwal A, Moritz TE, Henderson WG, Tarazi R, Shroyer AL, Sethi GK, Grover FL, Hammermeister KE. Atrial fibrillation after cardiac surgery: a major morbid event? *Ann Surg*. 1997;226:501-511.

10. Mathew JP, Fontes ML, Tudor IC, Ramsay J, Duke P, Mazer CD, Barash PG, Hsu PH, Mangano DT; Investigators of the Ischemia Research and Education Foundation; Multicenter Study of Perioperative Ischemia Research Group. A multicenter risk index for atrial fibrillation after cardiac surgery. *JAMA*. 2004;;291:1720-1729.

11. Banach M, Rysz J, Drozdz JA, Okonski P, Misztal M, Barylski M, Irzmanski R, Zaslonka J. Risk factors of atrial fibrillation following coronary artery bypass grafting: a preliminary report. *Circ J*. 2006;70:438-441.

12. Mariscalco G, Engström KG. Postoperative atrial fibrillation is associated with late mortality after coronary surgery, but not after valvular surgery. *Ann Thorac Surg*. 2009;88:1871-1876.

13. Ahlsson A, Fengsrud E, Bodin L, Englund A. Postoperative atrial fibrillation in patients undergoing aortocoronary bypass surgery carries an eightfold risk of future atrial fibrillation and a doubled cardiovascular mortality. *Eur J Cardiothorac Surg*. 2010;37:1353-1359.

14. Andrews TC, Reimold SC, Berlin JA, Antman EM. Prevention of supraventricular arrhythmias after coronary artery bypass surgery. A meta-analysis of randomized control trials. *Circulation*. 1991;84(5 Suppl):III236-44.

15. Frost L, Mølgaard H, Christiansen EH, Hjortholm K, Paulsen PK, Thomsen PE. Atrial fibrillation and flutter after coronary artery bypass surgery: epidemiology, risk factors and preventive trials. *Int J Cardiol*. 1992;36:253-261.

16. Maisel WH, Rawn JD, Stevenson WG. Atrial fibrillation after cardiac surgery. *Ann Intern Med*. 2001;135:1061-1073.

17. Auer J, Weber T, Berent R, Ng CK, Lamm G, Eber B. Risk factors of postoperative atrial fibrillation after cardiac surgery. *J Card Surg*. 2005;20:425-31.

18. Leitch JW, Thomson D, Baird DK, Harris PJ. The importance of age as a predictor of atrial fibrillation and flutter after coronary artery bypass grafting. *J Thorac Cardiovasc Surg*. 1990;100:338-342.

19. Yadava M, Hughey AB, Crawford TC. Postoperative atrial fibrillation: incidence, mechanisms, and clinical correlates. *Cardiol Clin*. 2014;32::627-636.

20. Lo B, Fijnheer R, Nierich AP, Bruins P, Kalkman CJ. C-reactive protein is a risk indicator for atrial fibrillation after myocardial revascularization. *Ann Thorac Surg*. 2005;79:1530-1535.

21. Guarnieri T, Nolan S, Gottlieb SO, Dudek A, Lowry DR. Intravenous amiodarone for the prevention of atrial fibrillation after open heart surgery: the Amiodarone Reduction in Coronary Heart (ARCH) trial. *J Am Coll Cardiol*. 1999;34:343-347.

22. Gu S, Su PX, Liu Y, Yan J, Zhang XT, Wang TY. Low-dose amiodarone for the prevention of atrial fibrillation after coronary artery bypass grafting in patients older than 70 years. *Chin Med J (Engl)*. 2009;122:2928-2932.

23. Pfisterer ME, Klöter-Weber UC, Huber M, Osswald S, Buser PT, Skarvan K, Stulz PM. Prevention of supraventricular tachyarrhythmias after open heart operation by low-dose sotalol: a prospective, double-blind, randomized, placebo-controlled study. *Ann Thorac Surg*. 1997;64:1113-1119.

24. Yagdi T, Nalbantgil S, Ayik F, Apaydin A, Islamoglu F, Posacioglu H, Calkavur T, Atay Y, Buket S. Amiodarone reduces the incidence of atrial fibrillation after coronary artery bypass grafting. *J Thorac Cardiovasc Surg*. 2003125:1420-1425.

25. Funk M, Richards SB, Desjardins J, Bebon C, Wilcox H. Incidence, timing, symptoms, and risk factors for atrial fibrillation after cardiac surgery. *Am J Crit Care*. 2003;12:424-33.

26. Steinberg BA, Zhao Y, He X, Hernandez AF, Fullerton DA, Thomas KL, Mills R, Klaskala W, Peterson ED, Piccini JP. Management of postoperative atrial fibrillation and subsequent outcomes in contemporary patients undergoing cardiac surgery: insights from the Society of Thoracic Surgeons CAPS-Care Atrial Fibrillation Registry. *Clin Cardiol*. 2014;37:7-13.

27. Gladstone DJ, Spring M, Dorian P, Panzov V, Thorpe KE, Hall J, Vaid H, O'Donnell M, Laupacis A, Côté R, Sharma M, Blakely JA, Shuaib A, Hachinski V, Coutts SB, Sahlas DJ, Teal P, Yip S, Spence JD, Buck B, Verreault S, Casaubon LK, Penn A, Selchen D, Jin A, Howse D, Mehdiratta M, Boyle K, Aviv R, Kapral MK, Mamdani M; EMBRACE Investigators and Coordinators. Atrial fibrillation in patients with cryptogenic stroke. *N Engl J Med*. 2014;370:2467-2477.

28. Sanna T, Diener HC, Passman RS, Di Lazzaro V, Bernstein RA, Morillo CA, Rymer MM, Thijs V, Rogers T, Beckers F, Lindborg K, Brachmann J; CRYSTAL AF Investigators. Cryptogenic stroke and underlying atrial fibrillation. *N Engl J Med*. 2014;370:2478-2486.

29. Reiffel J, Verma A, Halperin JL, Gersh B, Tombul S, Carrithers J, Sherfesee L, Kowey P. Rationale and design of REVEALAF: a prospective study of previously undiagnosed atrial fibrillation as documented by an insertable cardiac monitor in high-risk patients. *Am Heart J*. 2014;167:22-27.

30. Lowres N, Freedman SB, Gallagher R, Kirkness A, Marshman D, Orchard J, Neubeck L. Identifying postoperative atrial fibrillation in cardiac surgical patients posthospital discharge, using iPhone ECG: a study protocol. *BMJ Open*. 2015;13:e006849.

31. Mitchell LB; CCS Atrial Fibrillation Guidelines Committee. Canadian Cardiovascular Society atrial fibrillation guidelines 2010: prevention and treatment of atrial fibrillation following cardiac surgery. *Can J Cardiol*. 2011;27:91-97.

32. European Heart Rhythm Association; European Association for Cardio-Thoracic Surgery, Camm AJ, Kirchhof P, Lip GY, Schotten U, Savelieva I, Ernst S, Van Gelder IC, Al-Attar N, Hindricks G, Prendergast B, Heidbuchel H, Alfieri O, Angelini A, Atar D, Colonna P, De Caterina R, De Sutter J, Goette A, Gorenek B, Heldal M, Hohloser SH, Kolh P, Le Heuzey JY, Ponikowski P, Rutten FH. Guidelines for the management of

atrial fibrillation: the Task Force for the Management of Atrial Fibrillation of the European Society of Cardiology (ESC). *Eur Heart J.* 2010;31:2369-2429.

33. January CT, Wann LS, Alpert JS, Calkins H, Cigarroa JE, Cleveland JC Jr, Conti JB, Ellinor PT, Ezekowitz MD, Field ME, Murray KT, Sacco RL, Stevenson WG, Tchou PJ, Tracy CM, Yancy CW; ACC/AHA Task Force Members. 2014 AHA/ACC/HRS guideline for the management of patients with atrial fibrillation: a report of the American College of Cardiology/American Heart Association Task Force on practice guidelines and the Heart Rhythm Society. *Circulation*. 2014;130:e199-267.

34. Qaddoura A, Kabali C, Drew D, van Oosten EM, Michael KA, Redfearn DP, Simpson CS, Baranchuk A. Obstructive sleep apnea as a predictor of atrial fibrillation after coronary artery bypass grafting: a systematic review and meta-analysis. *Can J Cardiol*. 2014;30:1516-1522.

35. Lang RM, Bierig M, Devereux RB, Flachskampf FA, Foster E, Pellikka PA, Picard MH, Roman MJ, Seward J, Shanewise JS, Solomon SD, Spencer KT, Sutton MS, Stewart WJ; Chamber Quantification Writing Group; AmericanSociety of Echocardiography's Guidelines and Standards Committee; European Association of Echocardiography. Recommendations for chamber quantification: a report from the AmericanSociety of Echocardiography's Guidelines and Standards Committee and the Chamber Quantification Writing Group, developed in conjunction with the European Association of Echocardiography, a branch of the European Society of Cardiology. *J Am Soc Echocardiogr*. 2005;18:1440-1463.

36. Shinbane JS, Merkert, M, Fogoros, R, Mehta, V, Cao, M, Saxon, LA. Wearable Wireless Arrhythmia Detection Patches: Diagnostic Arrhythmia Yield, Time to First Arrhythmia, and Patient Compliance. *Heart Rhythm* 2013;10(5S):S305.

37. Shrivastav M, Padte S, Sinha N. Patient experience with a novel patch-like external loop recorder for cardiac arrhythmia detection in India. *Expert Rev Med Devices*. 2014 May;11(3):259-264.

14. Appendices

Appendix A: Product information for the Medtronic SEEQ™ mobile cardiac telemetry system.

Appendix B: Validation data of the SEEQ™ mobile cardiac telemetry system

Appendix C: Rhythm diagnoses collected by the Medtronic SEEQ™ mobile cardiac telemetry system in the SEARCH-AF trial.

Appendix D: Sample summary and episode reports from data collected by the Medtronic SEEQ™ mobile cardiac telemetry system.

Appendix E: The CardioStat® cardiac rhythm monitoring device.

Appendix F: Rhythm diagnoses reported by the CardioStat® cardiac rhythm monitoring system and sample summary report.

Appendix G: Systematic review of completed and ongoing observational studies addressing the incidence of post-operative atrial arrhythmias after cardiac surgery.

Appendix H: Adverse cardiovascular endpoint definitions.

Appendix I: Major bleeding outcomes definition.

Appendix J: Serious adverse event collecting and reporting.

Appendix K: The CHA_2DS_2-VASC score.

Appendix A: Product information for the Medtronic SEEQ mobile cardiac telemetry system[1]

SEEQ™ MCT Wearable Sensor

- Each sensor can be worn for 7.5 days – up to 30 days
- Adhesive-backed sensor is applied to chest
- Communicates with the wireless transmitter
- The Wearable Sensor automatically captures events and/or patient can press the button to mark the event

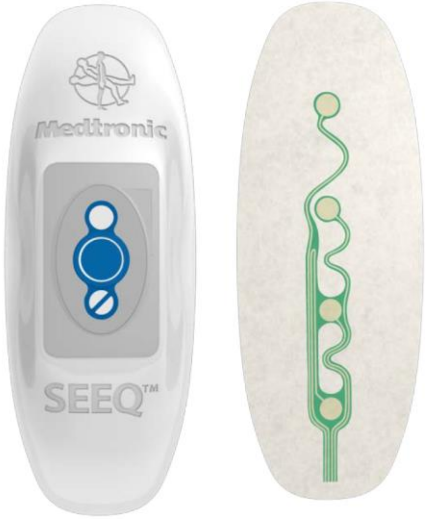

SEEQ™ MCT Transmitter

- Relays sensor data continuously via cellular signals to Medtronic Monitoring Center
- Rechargeable from standard electrical outlet
- Compact enough to tuck in purse or pocket
- Battery life is 12 hours
- Must be within 30 feet of the transmitter for successful data transmission

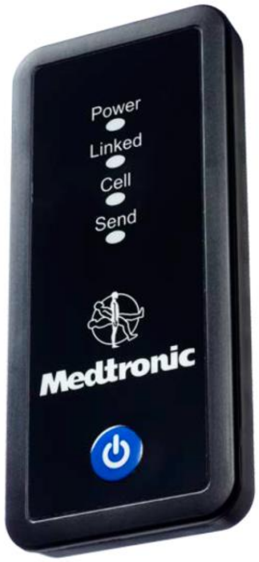

Appendix A: Product information for the Medtronic SEEQ mobile cardiac telemetry system[1]

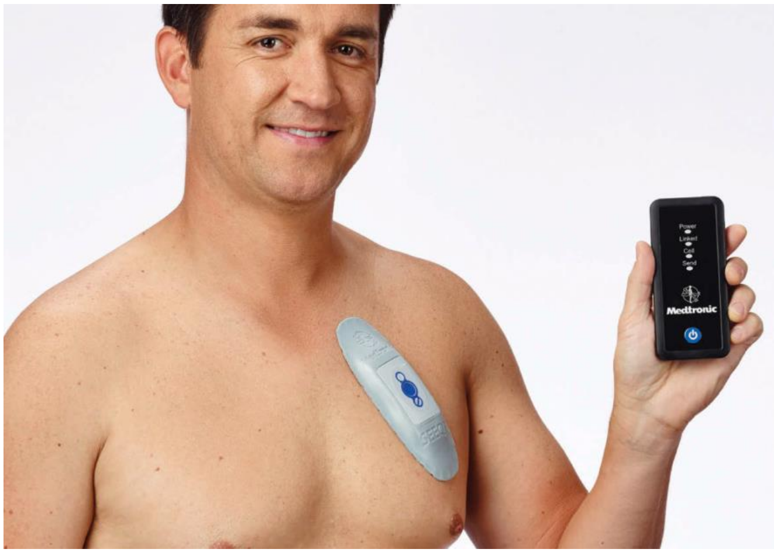

[1]http://www.medtronicdiagnostics.com/wcm/groups/mdtcom_sg/@mdt/documents/documents/seeq-system-overview.pdf. Accessed on January 18, 2015.

Appendix B: Validation data of the SEEQ™ mobile cardiac telemetry system

<u>Investigation of Monitoring Performance in Adherent Cardiac Telemetry (IMPACT) Study (www.clincialtrials.gov NCT00919568)</u>

Note: Data provided by Medtronic.

Objective: To compare the ambulatory arrhythmia monitoring performance of the Corventis NUVANT MCT System to a commercially available ambulatory cardiac monitoring system (Cardionet®)

Primary Endpoint

- Detection of clinically significant arrhythmias.

Study Design

- 20 patients undergoing concurrent monitoring with the Corventis NUVANT Mobile Cardiac Telemetry (MCT) System and a commercially available, clinically-approved MCT system (CardioNet® MCT; please refer to www.cardionet.com for details of the system). This system will be referred to as the "conventional" MCT system.
- The study duration was 30 days.
- Note: Medtronic had purchased the Corventis NUVANT MCT system; it is now marketed as the SEEQ™ mobile cardiac telemetry system.
- Patient reports from both systems evaluated by independent physician review panel consisting of 3 blinded physician reviewers.
- Reports were provided to each reviewer in random order.
- Final determination based on panel consensus.

Research Question: Were any of the arrhythmias clinically significant?

Results:

- Concordance between the NUVANT MCT system and conventional (Cardionet®) MCT was noted in 11 of 13 (85%) patients
- Discordance between the 2 systems was observed in 2 of 13 patients (15%). This was explained by difference in rhythm detection design (ie: differences in rhythm detection criteria) between the 2 systems.

Appendix C: Rhythm diagnoses collected by the Medtronic SEEQ™ mobile cardiac telemetry system in the SEARCH-AF trial.

For the SEARCH-AF trial, we intend the SEEQ™ mobile cardiac telemetry system to be used in a similar capacity as a 7-day Holter for post-cardiac surgical patients.

1) We will receive "End of Use Summary Reports" on a weekly basis as well as after the conclusion of the 30-day or 14-day monitoring period. All episode reports will be attached with the weekly End of Use Summary reports.

2) If there are arrhythmic events which met the "Notification Criteria", we do not require Medtronic to perform real-world notification of these events to the ordering physician or the research site or the research participant. Arrhythmic episodes meeting "Notification Criteria" will be included in the End of Use Summary report which is generated on a weekly basis as well as after the conclusion of the 30-day or 14-day monitoring period.

Specific parameters of arrhythmic events under "Notification Criteria"

Arrhythmia	*Notification Criteria*
**VF**	*Yes*
**ICD discharge**	*Yes*
**VT**	*180 bpm and ≥10 beats*
**Wide complex tachycardia**	*180 bpm and ≥10 beats*
**PVCs**	*Never notified*
**Sinus bradycardia**	*≤30 bpm*
**Sinus tachycardia**	*≥150 bpm*
**SVT**	*≥150 bpm and ≥30 seconds*
**AF/AFL (fast)**	*≥150 bpm and ≥30 seconds*
**AF/AFL (slow)**	*≤30 bpm and ≥30 seconds*
**Pause**	*≥5 seconds*
**Mobitz I AV block**	*≤30 bpm*
**Mobitz II AV block**	*≤30 bpm*
**Isolated 2nd degree AV block**	*≤30 bpm*
**High degree AV block**	*≤30 bpm*
**3rd degree AV block**	*≤30 bpm*
**Patient-triggered ECG**	*None*
**Technicians discretion**	*Yes (Any)*

3) To assess AF burden in SEARCH-AF, we will have access on data pertaining to: (i) total of AF episodes; (ii) duration of each AF episode; and (iii) total duration of all AF episodes. In addition, we will have access to all rhythm strips which have been detected as AF by the device. This “raw data” will be provided by Medtronic. Please note that our adjudication results will not alter the Episode and End of Use reports as generated by Medtronic. In other words, if there is disagreement between our adjudicated events and Medtronic, the report as generated by Medtronic will not be altered. The results of the trial (as published in a scientific, peer-reviewed manuscript) will be based on the adjudicated results.

Appendix D: Sample summary and episode reports from data collected by the Medtronic SEEQ™ mobile cardiac telemetry system.

SEEQ™ MCT EPISODE REPORT

Medtronic

Full Name Patient 1 August 09, 2017

Report ID 616734

PATIENT INFORMATION

**Birthdate**	Oct 01, 1936
**Gender**	Male
**Phone**	1-000-000-0000
**Address**	Street Address<br>City, State ZIP Code

PHYSICIAN INFORMATION

**Physician**	Doctor, Name
**Institution**	Cardiology Clinic
**Phone**	1-000-000-0000
**Address**	Street Address<br>City, State ZIP Code

PRESCRIPTION SUMMARY

**System**	SEEQ MCT	**Prescription**	14 days
**Monitored**	Since Dec 03, 2016 (9 days; 9 days worn)		
**Physician's Diagnostic Codes**	-R55 Syncope<br>-		

PHYSICIAN INTERPRETATION

SIGNATURE: ______________________ DATE: ______________________

© Copyright 2016 Medtronic Monitoring Inc. The observations provided are preliminary and are intended for review and interpretation by the prescribing physician as part of the information used by the physician to make a diagnosis. Any observations in this report must be confirmed by a physician. Please log in at www.medtronicmonitoring.com to review additional data related to this patient or call Medtronic Monitoring at 1-877-247-7449.

Appendix D: Sample summary and episode reports from data collected by the Medtronic SEEQ™ mobile cardiac telemetry system

SEEQ™ MCT EPISODE REPORT

Medtronic

Full Name Patient 1

Auto-Triggered **A**

December 11, 2016 04:43:05 EST (Day 9, Sunday)

**Rhythm**	Atrial Fibrillation, IVCD, Pause	**Ref ID**	616734
**HR**	Min: 45 bpm; Max: 50 bpm; Avg: 43 bpm	Notifiable	
**Observations**	Atrial Fibrillation with IVCD and 3.1 second Pause / Asymptomatic / Doctor Notified at 0359 PST		

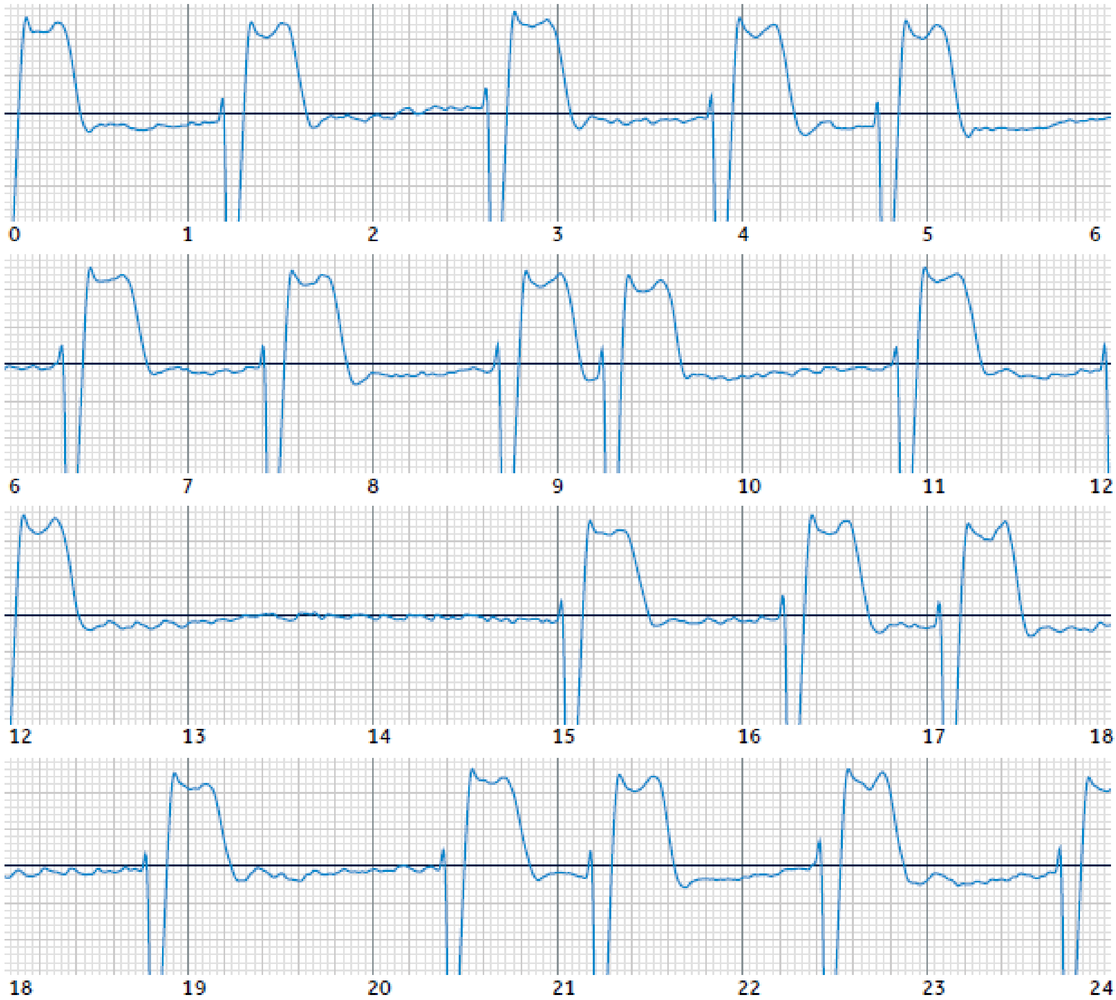

© Copyright 2016 Medtronic Monitoring Inc. The observations provided are preliminary and are intended for review and interpretation by the prescribing physician as part of the information used by the physician to make a diagnosis. Any observations in this report must be confirmed by a physician. Please log in at www.medtronicmonitoring.com to review additional data related to this patient or call Medtronic Monitoring at 1-877-247-7449.

Appendix D: Sample summary and episode reports from data collected by the Medtronic SEEQ™ mobile cardiac telemetry system

SEEQ™ MCT
EPISODE REPORT

Medtronic

Full Name Patient 1

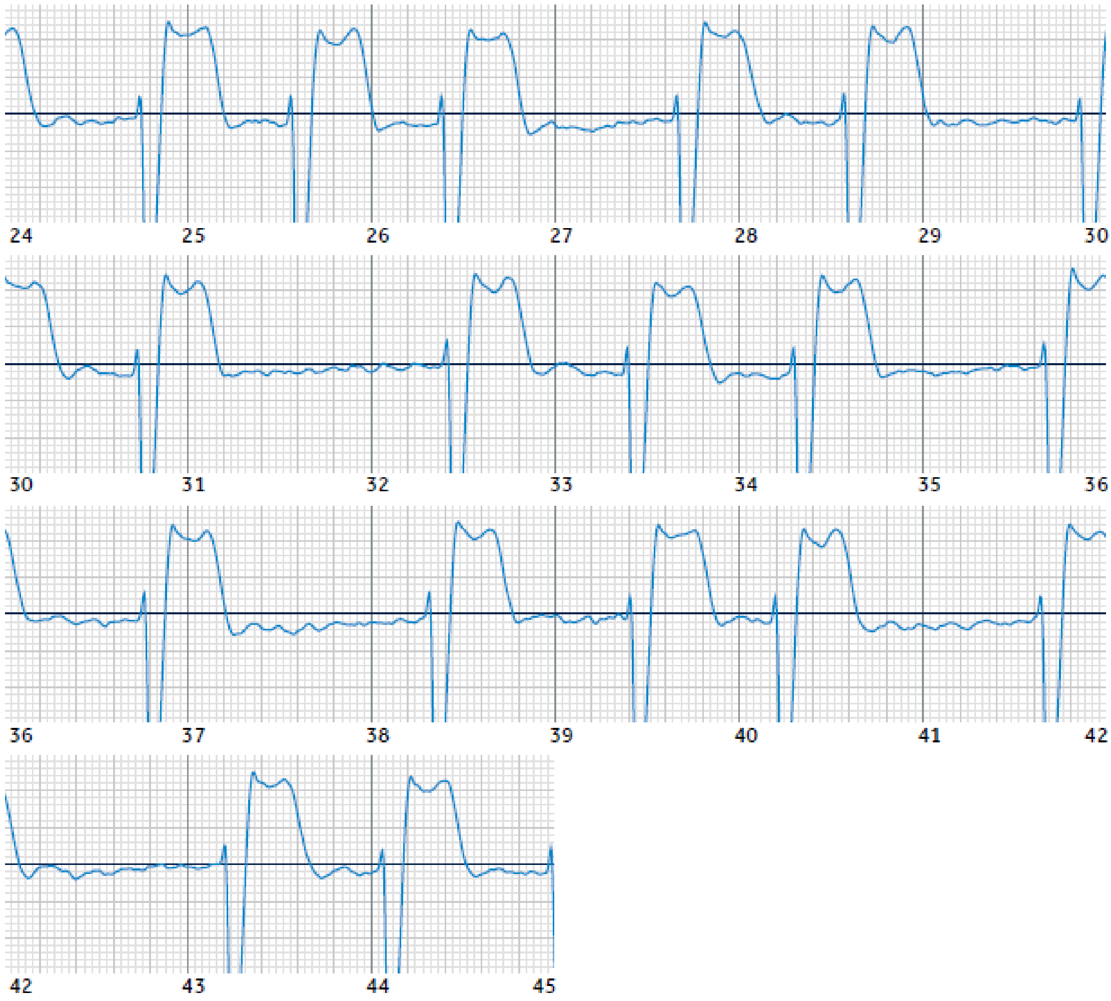

© Copyright 2016 Medtronic Monitoring Inc. The observations provided are preliminary and are intended for review and interpretation by the prescribing physician as part of the information used by the physician to make a diagnosis. Any observations in this report must be confirmed by a physician. Please log in at www.medtronicmonitoring.com to review additional data related to this patient or call Medtronic Monitoring at 1-877-247-7449.

Appendix D: Sample summary and episode reports from data collected by the Medtronic SEEQ™ mobile cardiac telemetry system

SEEQ™ MCT
END-OF-USE SUMMARY REPORT

Medtronic

Full Name Patient 1 December 16, 2016

Report ID 513

PATIENT INFORMATION

**Birthdate**	Oct 01, 1936
**Gender**	Male
**Phone**	1-000-000-0000
**Address**	Street Address<br>City, State ZIP Code

PHYSICIAN INFORMATION

**Physician**	Doctor, Name
**Institution**	Cardiology Clinic
**Phone**	1-000-000-0000
**Address**	Street Address<br>City, State ZIP Code

Heart Rate

(min-max-avg of 5-minute averages)

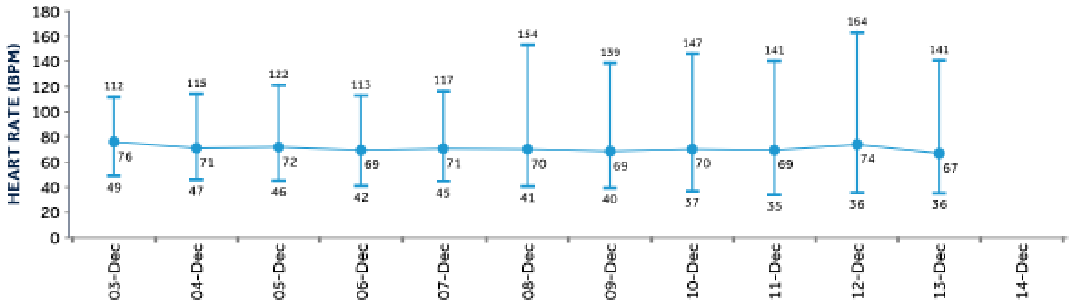

PRESCRIPTION SUMMARY

**System**	SEEQ MCT	**Prescription**	14 days
**Monitored**	Dec 03, 2016 - Dec 13, 2016 (11 days; 11 days worn)		
**Physician's Diagnostic Codes**	-R55 Syncope		

CARDIAC SUMMARY

(5-minute averages)

**Min Heart Rate**	35 bpm
**Max Heart Rate**	164 bpm
**Avg Heart Rate**	71 bpm

PHYSICIAN INTERPRETATION

SIGNATURE: ______________________ **DATE:** ______________

© Copyright 2016 Medtronic Monitoring Inc. The observations provided are preliminary and are intended for review and interpretation by the prescribing physician as part of the information used by the physician to make a diagnosis. Any observations in this report must be confirmed by a physician. Please log in at www.medtronicmonitoring.com to review additional data related to this patient or call Medtronic Monitoring at 1-877-247-7449.

Appendix D: Sample summary and episode reports from data collected by the Medtronic SEEQ™ mobile cardiac telemetry system

SEEQ™ MCT
END-OF-USE SUMMARY REPORT

Medtronic

ARRHYTHMIA SUMMARY

Total ECGs Reviewed 441

Total Reportable ECGs 22

- -Total Auto-Triggered Episodes 16
- -Total Patient-Triggered 6
- -Total Patient-Triggered w/Arrhythmia 6

Total Notifiable ECGs 2

Ventricular

PAGE

Wide Complex Tachy	0 episodes		
Monomorphic VT	0 episodes		
Polymorphic VT/VF	0 episodes		
Idioventricular	0 episodes		

Ectopy

Total PVCs	13,937		
Average Daily PVC Burden	1.3%		
Highest Daily PVCs	5,451	Day 10	4
Highest Daily PVC Burden	4.5%		

AV Blocks/Pause

Pause	13 episodes
AV Block	0 episodes

Atrial

PAGE

Atrial Fibrillation 379 episodes

Atrial Fibrillation Burden
(episodes ≥ 30 sec)

Average V rate in AF	79 bpm		
Highest Daily AF Burden	21h 8m	Day 2	3
Average Daily AF Burden	14h 29m		
Total Time in AF	6d 15h 21m	67.4%	

Atrial Fibrillation: Episode Overview (#)

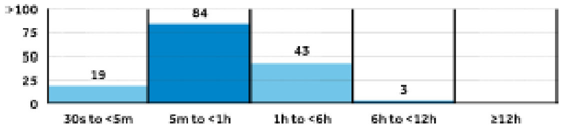

Atrial Flutter	3 episodes
SVT	0 episodes

Junctional Rhythm 0 episodes

Normal

Sinus Brady	0 episodes
Sinus Tachy	1 episode

© Copyright 2016 Medtronic Monitoring Inc. The observations provided are preliminary and are intended for review and interpretation by the prescribing physician as part of the information used by the physician to make a diagnosis. Any observations in this report must be confirmed by a physician. Please log in at www.medtronicmonitoring.com to review additional data related to this patient or call Medtronic Monitoring at 1-877-247-7449.

Appendix D: Sample summary and episode reports from data collected by the Medtronic SEEQ™ mobile cardiac telemetry system

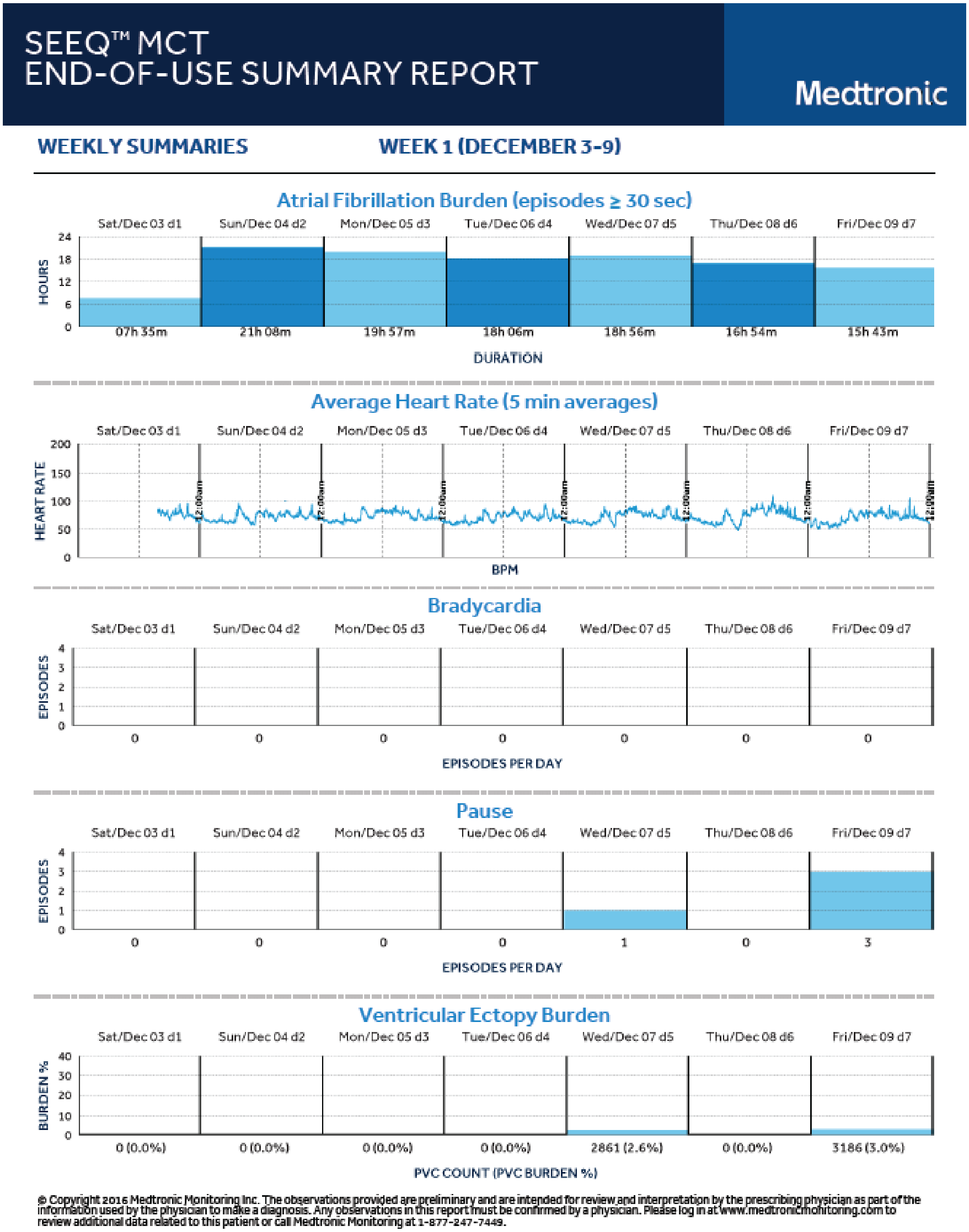

Appendix D: Sample summary and episode reports from data collected by the Medtronic SEEQ™ mobile cardiac telemetry system

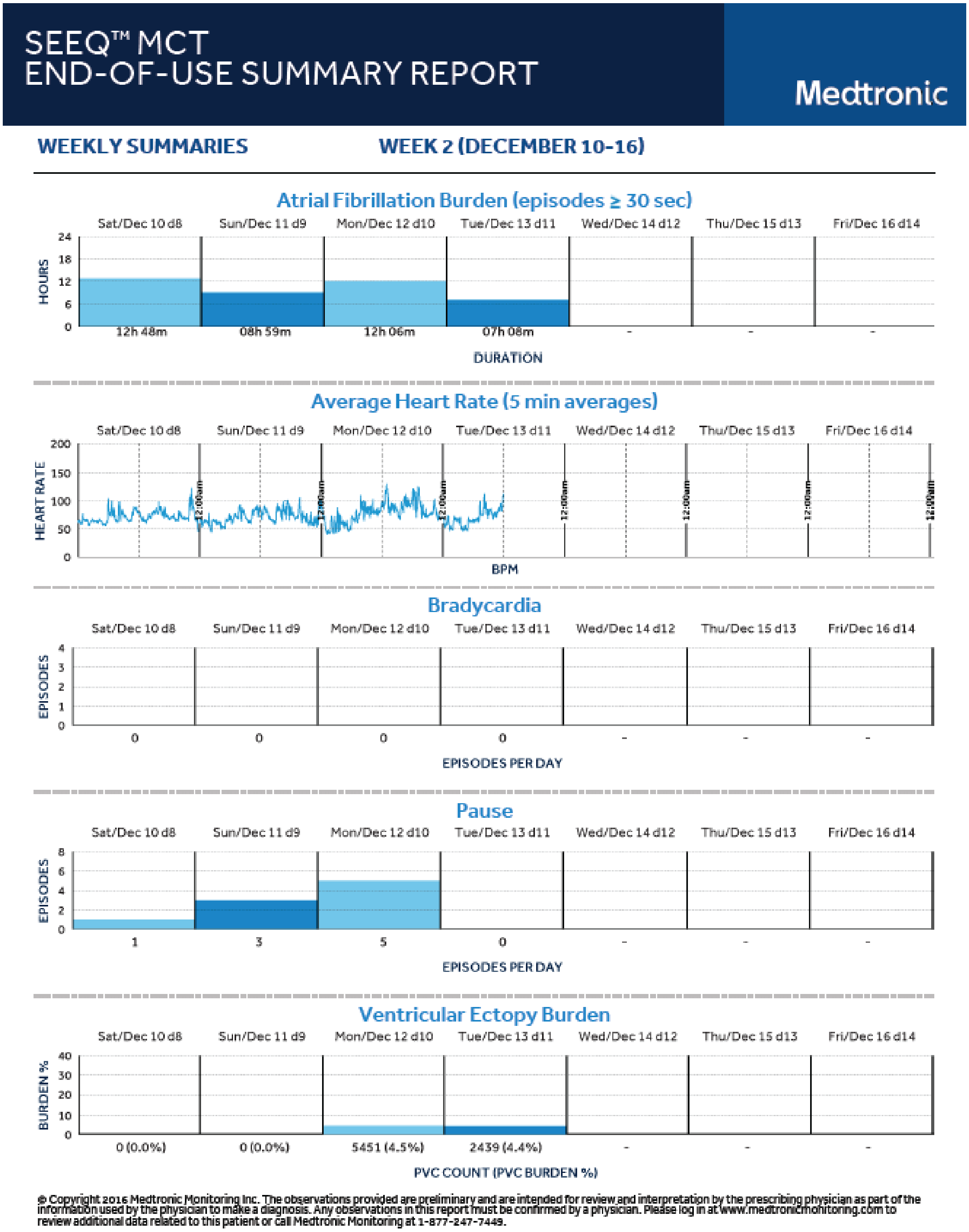

Appendix D: Sample summary and episode reports from data collected by the Medtronic SEEQ™ mobile cardiac telemetry system

SEEQ™ MCT
END-OF-USE SUMMARY REPORT

Medtronic

SENSOR 1 **DECEMBER 3-8**

Baseline **B**

December 03, 2016 15:48:01 EST (Day 1, Saturday)

**Rhythm**	Atrial Fibrillation, IVCD	**Ref ID**	616254
**HR**	Min: 90 bpm, Max: 110 bpm, Avg: 100 bpm		
**Observations**			

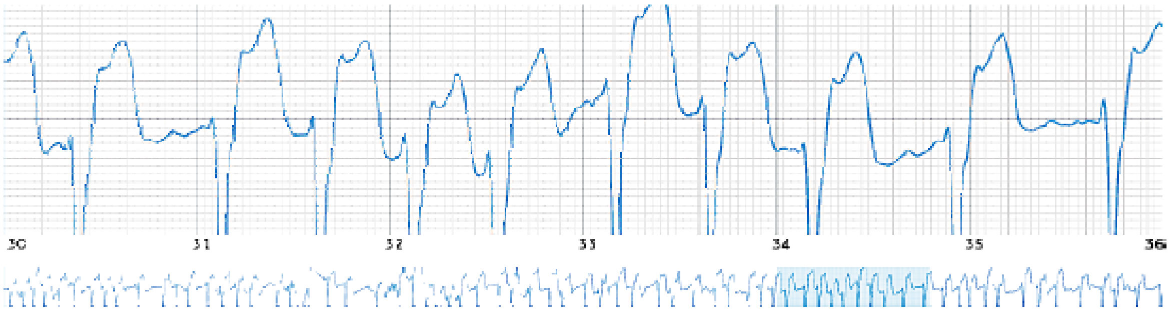

Patient-Triggered **P**

December 04, 2016 08:39:00 EST (Day 2, Sunday)

**Rhythm**	Atrial Fibrillation, IVCD, Artifact	**Ref ID**	616268
**HR**	Min: 60 bpm, Max: 90 bpm, Avg: 75 bpm		
**Observations**	Atrial Fibrillation with IVCD / Palpitations while Sitting / Spoke with Patient at 0553 PST		

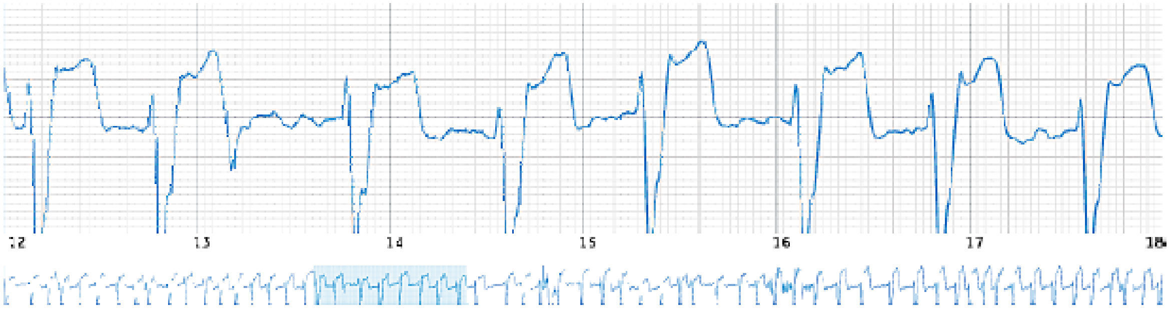

© Copyright 2016 Medtronic Monitoring Inc. The observations provided are preliminary and are intended for review and interpretation by the prescribing physician as part of the information used by the physician to make a diagnosis. Any observations in this report must be confirmed by a physician. Please log in at www.medtronicmonitoring.com to review additional data related to this patient or call Medtronic Monitoring at 1-877-247-7449.

Appendix D: Sample summary and episode reports from data collected by the Medtronic SEEQ™ mobile cardiac telemetry system

SEEQ™ MCT
END-OF-USE SUMMARY REPORT

Medtronic

SENSOR 1 **DECEMBER 3-8 (CONTINUED)**

Patient-Triggered P

December 05, 2016 08:49:33 EST (Day 3, Monday)

**Rhythm**	Atrial Fibrillation, IVCD	**Ref ID**	616310
**HR**	Min: 70 bpm, Max: 110 bpm, Avg: 90 bpm		
**Observations**	Atrial Fibrillation with IVCD / Dizzy while Standing / Spoke with Patient at 08:00 PST		

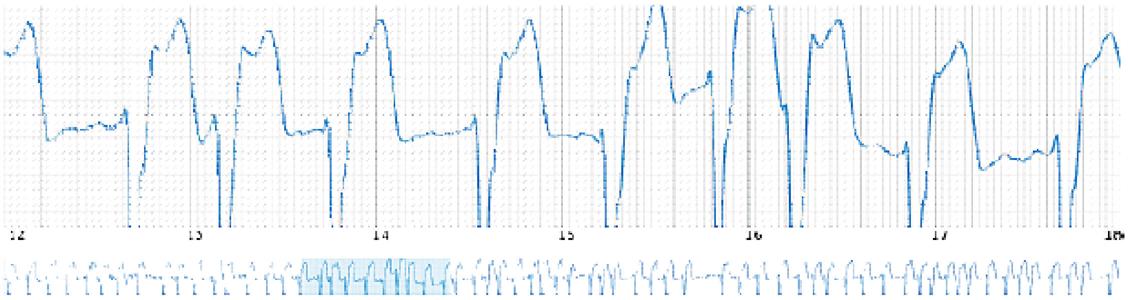

Patient-Triggered P

December 06, 2016 08:57:54 EST (Day 4, Tuesday)

**Rhythm**	Atrial Fibrillation, IVCD	**Ref ID**	616325
**HR**	Min: 60 bpm, Max: 80 bpm, Avg: 70 bpm		
**Observations**	Atrial Fibrillation with IVCD /Lightheaded while Sitting / Spoke with Patient at 0624 PST		

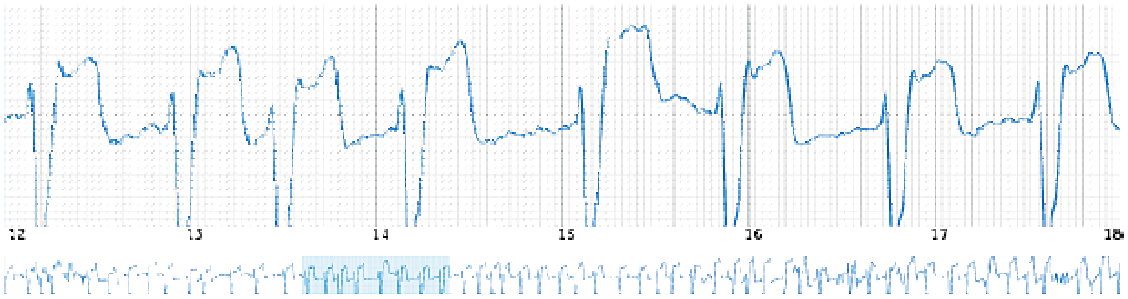

© Copyright 2016 Medtronic Monitoring Inc. The observations provided are preliminary and are intended for review and interpretation by the prescribing physician as part of the information used by the physician to make a diagnosis. Any observations in this report must be confirmed by a physician. Please log in at www.medtronicmonitoring.com to review additional data related to this patient or call Medtronic Monitoring at 1-877-247-7449.

Appendix D: Sample summary and episode reports from data collected by the Medtronic SEEQ™ mobile cardiac telemetry system

SEEQ™ MCT
END-OF-USE SUMMARY REPORT

Medtronic

SENSOR 1 DECEMBER 3-8 (CONTINUED)

Auto-Triggered A

December 07, 2016 04:19:19 EST (Day 5, Wednesday)

Rhythm	Atrial Fibrillation, IVCD, Pause	Ref ID	616341
HR	Min: 60 bpm, Max: 85 bpm, Avg: 72 bpm		
Observations	Atrial Fibrillation, IVCD and 2.0 second Pause.		

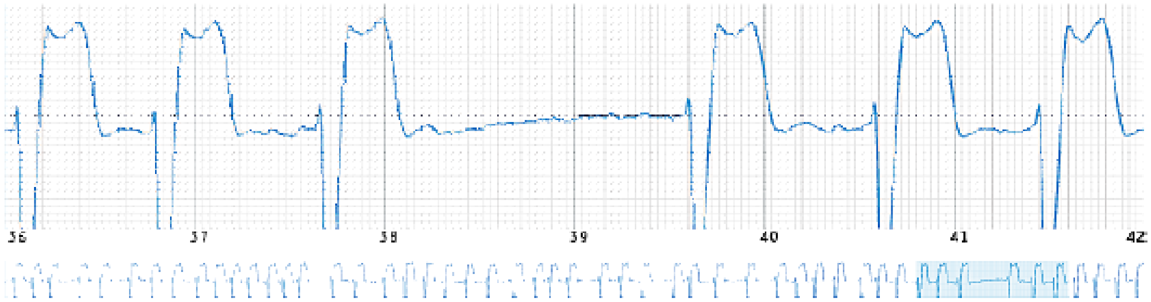

Patient-Triggered P

December 07, 2016 10:13:01 EST (Day 5, Wednesday)

Rhythm	Atrial Fibrillation, PVCs, IVCD	Ref ID	616346
HR	Min: 80 bpm, Max: 90 bpm, Avg: 85 bpm		
Observations	Atrial Fibrillation with IVCD and PVC / Other-Heartburn While Standing / Spoke with Patient at 0727 PST		

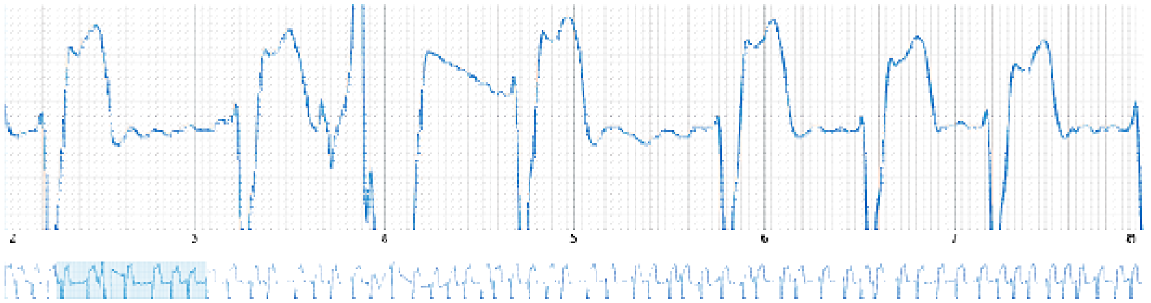

© Copyright 2016 Medtronic Monitoring Inc. The observations provided are preliminary and are intended for review and interpretation by the prescribing physician as part of the information used by the physician to make a diagnosis. Any observations in this report must be confirmed by a physician. Please log in at www.medtronicmonitoring.com to review additional data related to this patient or call Medtronic Monitoring at 1-877-247-7449.

Appendix E: The CardioStat® cardiac rhythm monitoring device

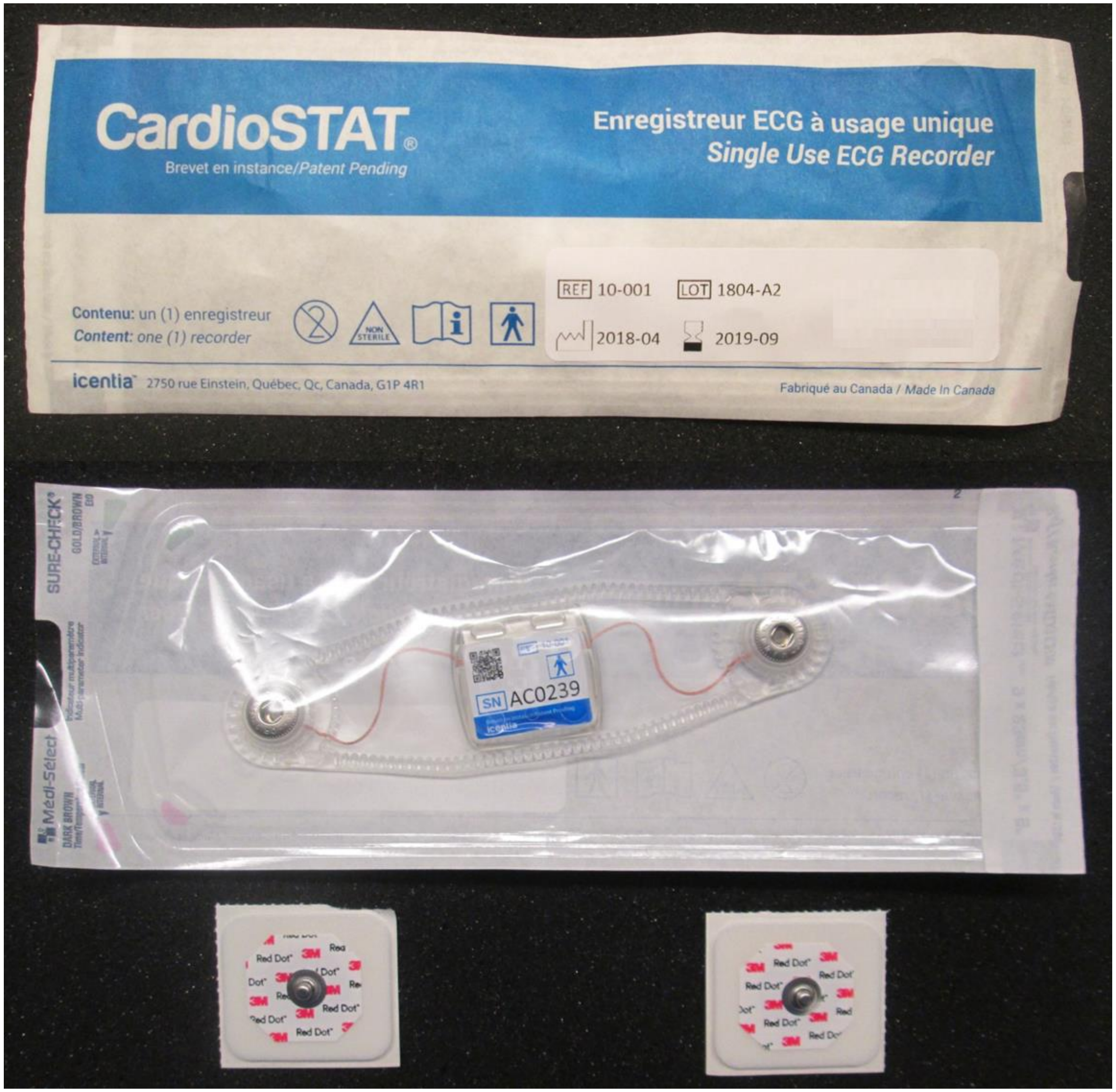

Device dimensions: Weight = 20 grams; Length = 140 mm (5.5 inches); Thickness = 9 mm (0.35 inches).

Health Canada Class 2 Medical Device License Number 93860.

The CardioStat® cardiac rhythm monitoring device is attached to 2 skin electrodes. These electrodes will adhere onto the anterior left chest wall to provide up to 14 days of continuous heart rhythm monitoring.

Appendix F: Example of rhythm diagnoses reported by the CardioStat® cardiac rhythm monitoring system and summary report.

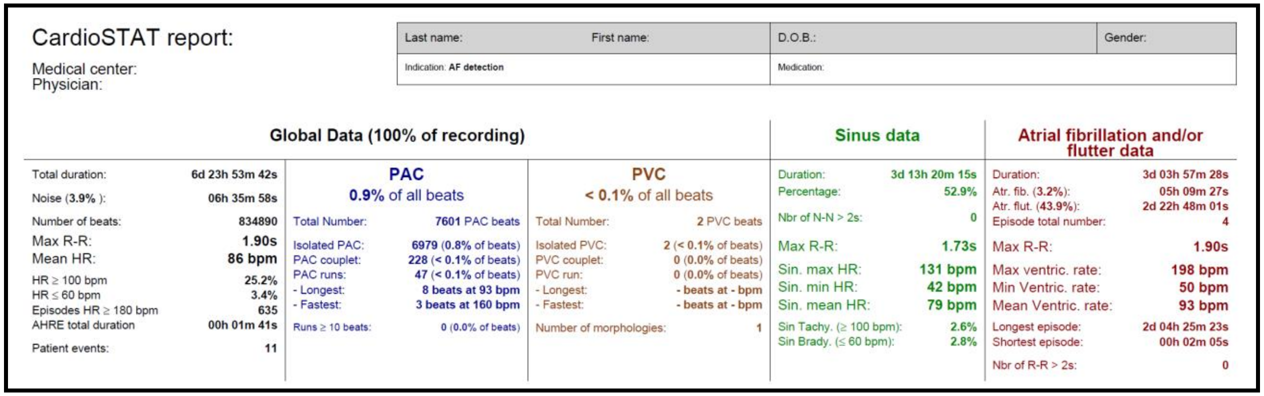

CardioSTAT report:

Medical center:
Physician:

Last name:	First name:	D.O.B.:	Gender:
Indication: **AF detection**		Medication:	

Global Data (100% of recording)

Total duration:	6d 23h 53m 42s
Noise (3.9%):	06h 35m 58s
Number of beats:	834890
Max R-R:	1.90s
Mean HR:	86 bpm
HR ≥ 100 bpm	25.2%
HR ≤ 60 bpm	3.4%
Episodes HR ≥ 180 bpm	635
AHRE total duration	00h 01m 41s
Patient events:	11

PAC
0.9% of all beats

Total Number:	7601 PAC beats
Isolated PAC:	6979 (0.8% of beats)
PAC couplet:	228 (< 0.1% of beats)
PAC runs:	47 (< 0.1% of beats)
- Longest:	8 beats at 93 bpm
- Fastest:	3 beats at 160 bpm
Runs ≥ 10 beats:	0 (0.0% of beats)

PVC
< 0.1% of all beats

Total Number:	2 PVC beats
Isolated PVC:	2 (< 0.1% of beats)
PVC couplet:	0 (0.0% of beats)
PVC run:	0 (0.0% of beats)
- Longest:	- beats at - bpm
- Fastest:	- beats at - bpm
Number of morphologies:	1

Sinus data

Duration:	3d 13h 20m 15s
Percentage:	52.9%
Nbr of N-N > 2s:	0
Max R-R:	1.73s
Sin. max HR:	131 bpm
Sin. min HR:	42 bpm
Sin. mean HR:	79 bpm
Sin Tachy. (≥ 100 bpm):	2.6%
Sin Brady. (≤ 60 bpm):	2.8%

Atrial fibrillation and/or flutter data

Duration:	3d 03h 57m 28s
Atr. fib. (3.2%):	05h 09m 27s
Atr. flut. (43.9%):	2d 22h 48m 01s
Episode total number:	4
Max R-R:	1.90s
Max ventric. rate:	198 bpm
Min Ventric. rate:	50 bpm
Mean Ventric. rate:	93 bpm
Longest episode:	2d 04h 25m 23s
Shortest episode:	00h 02m 05s
Nbr of R-R > 2s:	0

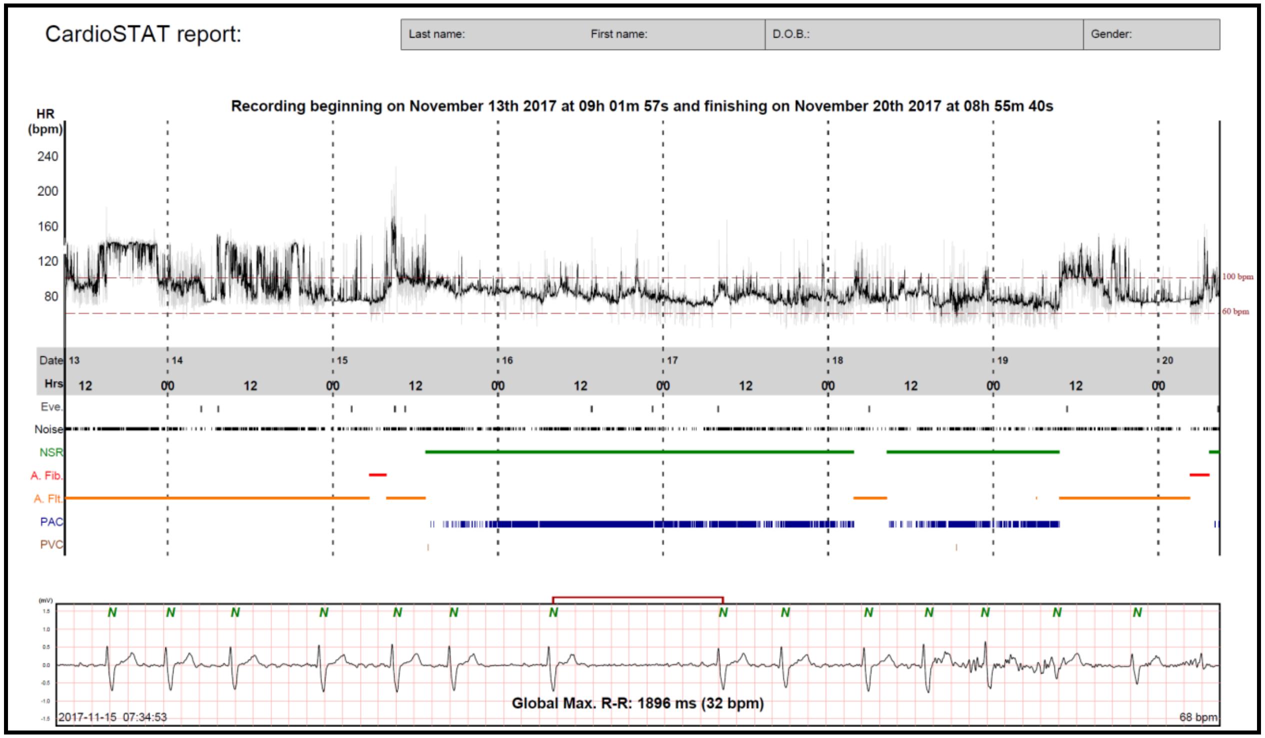

Appendix F: Example of rhythm diagnoses reported by the CardioStat® cardiac rhythm monitoring system and summary report.

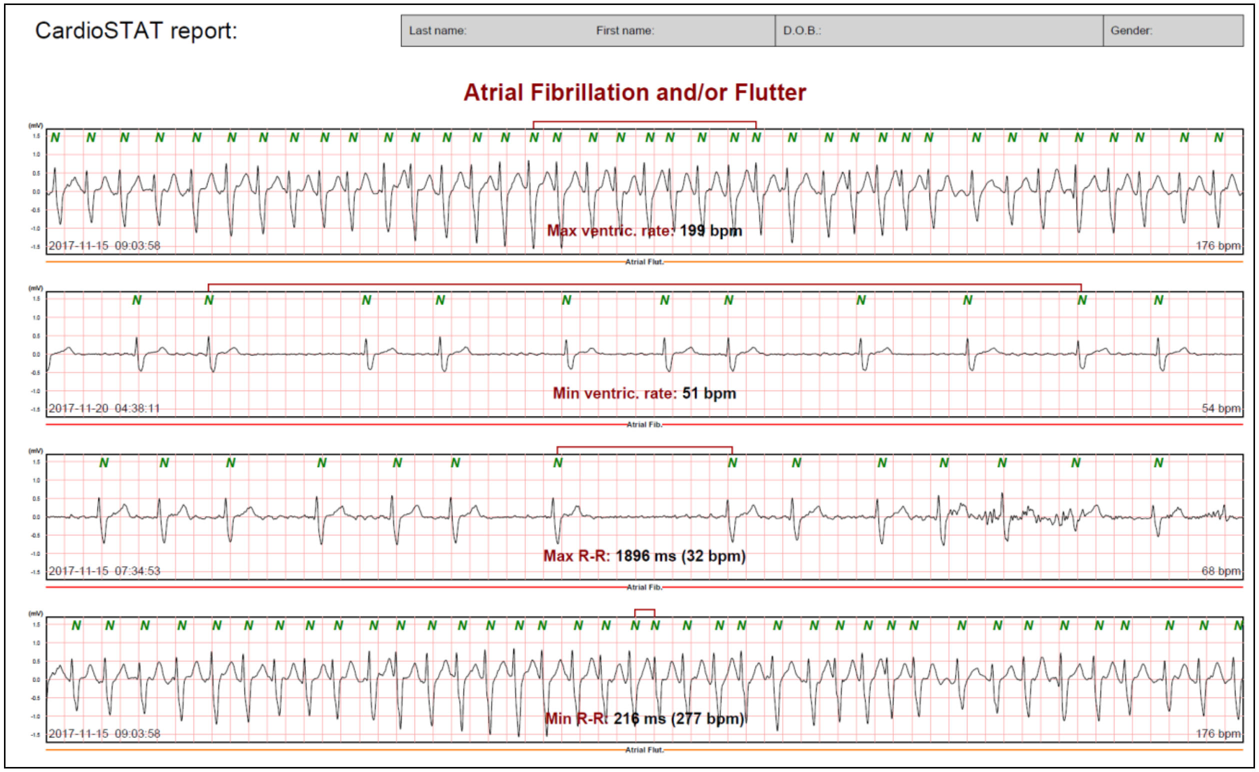
CardioSTAT report:
Last name:
First name:
D.O.B.:
Gender:
Atrial Fibrillation and/or Flutter
Max ventric. rate: 199 bpm
2017-11-15 09:03:58
176 bpm
Atrial Flut.
Min ventric. rate: 51 bpm
2017-11-20 04:38:11
54 bpm
Atrial Fib.
Max R-R: 1896 ms (32 bpm)
2017-11-15 07:34:53
68 bpm
Atrial Fib.
Min R-R: 216 ms (277 bpm)
2017-11-15 09:03:58
176 bpm
Atrial Flut.

Appendix F: List of cardiac rhythm findings which will be included in the final summary CardioStat® report in the SEARCH-AF trial.

List of cardiac rhythm findings which will be included in the final summary CardioStat® report in the SEARCH-AF trial
Atrial fibrillation (AF) burden
Atrial flutter (AFL) burden
Number of AF or AFL episodes lasting >30 seconds
Duration of the shortest and longest AF / AFL episode
Hourly distribution of AF and AFl episodes
Maximal R-R interval during AF and/or AFL
Maximal R-R interval during sinus rhythm
Maximal, minimal, and average ventricular rate
Presence of atrioventricular block and/or pauses
Presence of supraventricular ectopic beats

Presence of ventricular ectopic beats
Ventricular fibrillation
Sustained ventricular tachycardia (defined as lasting longer than 30 seconds with ventricular rates of >100 beats per minute, unless the subject has a known QRS duration of >120 ms and sinus tachycardia)
Any sinus interval >3.05 seconds or RR interval >5.05 seconds
Second degree (type II) and third-degree AV blocks
Ventricular rate of less than 30 bpm lasting for at least 1 minute

Appendix G: Systematic review of completed and ongoing observational studies addressing the incidence of post-operative atrial arrhythmias after cardiac surgery (current as of May 13 2015).

Appendix G: Systematic review of completed and ongoing observational studies addressing the incidence of post-operative

Study	Study design	Sample size	Status	Rhythm monitoring modality	Comments
**Funk et al.**	Observational, prospective study	302	Published	Wearable cardiac event monitor (recording of heart rhythm will commence upon subject-based self-triggering)	Subjects wore 14-day rhythm monitors which are activated by self-triggering (ie: non-continuous rhythm monitoring).
**Lowres et al.**	Observational, prospective pilot study	50	Ongoing, study design has been published	An i-phone based application to monitor subjects' heart rhythm	Subjects will be asked to record their rhythm status with an i-Phone app on a daily basis for 30 days after discharge from cardiac surgery.
**MONITOR-AF (NCT01395836)**	Observational, prospective, 2-arm study	50	Ongoing	Implantable loop recorder (ILR)	Subjects' rhythm status will be monitored for 1 year to assess for AF occurrence.
**Medtronic Reveal XT study (NCT01526343)**	Observational, prospective study	100	Ongoing	Implantable loop recorder (ILR) and "Traditional monitoring" (e.g. Holter and ECG)	All subjects underwent surgical AF ablation (as a stand-alone or concomitant procedure). An ILR was implanted in each subject. All subjects underwent traditional monitoring (ECG and Holter). The primary outcome was the number and burden of atrial tachyarrhythmia detected during pre-specified post-operative time intervals. The comparison of interest will be ILR vs. traditional monitoring (each subject serves as his/her own comparator).

atrial arrhythmias after cardiac surgery (current as of May 13 2015).

PUBMED search strategy: ("Cardiac Surgical Procedures"[mh] OR "Cardiac Surgical Procedures"[tiab] OR "Coronary Artery Bypass"[mh] OR "Coronary Artery Bypass"[tiab]) AND ("atrial fibrillation"[mh] OR "atrial fibrillation"[tiab]) AND "randomized controlled trial"[publication type].

Using this strategy, we did not find any published RCT on this particular topic. After omitting "AND "randomized controlled trial"[publication type]", we were able to identify 2 studies (Funk and Lowres).

Similar keywords used in the www.clinicaltrials.gov database

This yielded 2 more ongoing studies in the clinicaltrials.gov database: MONITOR-AF (NCT01395836) and Medtronic Reveal XT study (NCT01526343).

References:

Funk M, Richards SB, Desjardins J, Bebon C, Wilcox H. Incidence, timing, symptoms, and risk factors for atrial fibrillation after cardiac surgery. *Am J Crit Care*.2003 Sep;12(5):424-33.

Lowres N, Freedman SB, Gallagher R, Kirkness A, Marshman D, Orchard J, Neubeck L. Identifying postoperative atrial fibrillation in cardiac surgical patients post hospital discharge, using iPhone ECG: a study protocol. *BMJ Open*. 2015 Jan 13;5(1):e006849.

Appendix H: Major adverse cardiovascular endpoint (MACE) definition

Event	Definition
**All-cause mortality**	Death from any cause
**Myocardial infarction (third universal definition of myocardial infarction)**[1]	The term acute myocardial infarction (MI) should be used when there is evidence of myocardial necrosis in a clinical setting consistent with acute myocardial ischemia. Under these conditions any one of the following criteria meets the diagnosis for MI:<br><br>1) Detection of a rise and/or fall of cardiac biomarker values [preferably cardiac troponin (cTn)] with at least one value above the 99th percentile upper reference limit (URL) and with at least one of the following:<br><br>- Symptoms of ischaemia.<br>- New or presumed new significant ST-segment–T wave (ST–T) changes or new left bundle branch block (LBBB).<br>- Development of pathological Q waves in the ECG.<br>- Imaging evidence of new loss of viable myocardium or new regional wall motion abnormality.<br>- Identification of an intracoronary thrombus by angiography or autopsy.<br><br>2) Cardiac death with symptoms suggestive of myocardial ischemia and presumed new ischaemic ECG changes or new LBBB, but death occurred before cardiac biomarkers were obtained, or before cardiac biomarker values would be increased.<br><br>3) Percutaneous coronary intervention (PCI) related MI is arbitrarily defined by elevation of cTn values (>5 x 99th percentile URL) in patients with normal baseline values (≤99th percentile URL) or a rise of cTn values >20% if the baseline values are elevated and are stable or falling. In addition, either (i) symptoms suggestive of myocardial ischemia or (ii) new ischaemic ECG changes or (iii) angiographic findings consistent with a procedural complication or (iv) imaging

	demonstration of new loss of viable myocardium or new regional wall motion abnormality are required.<br><br>4) Stent thrombosis associated with MI when detected by coronary angiography or autopsy in the setting of myocardial ischemia and with a rise and/or fall of cardiac biomarker values with at least one value above the 99th percentile URL.<br><br>5) Coronary artery bypass grafting (CABG) related MI is arbitrarily defined by elevation of cardiac biomarker values (>10 x 99th percentile URL) in patients with normal baseline cTn values (≤99th percentile URL). In addition, either (i) new pathological Q waves or new LBBB, or (ii) angiographic documented new graft or new native coronary artery occlusion, or (iii) imaging evidence of new loss of viable myocardium or new regional wall motion abnormality.
**Stroke**	Diagnosis of stroke will require the abrupt onset of focal neurological symptoms lasting at least 24 hours. Accompanying neurovascular imaging information will be used for event adjudication, if it is performed.[2]
**Systemic embolism**	Clinical history consistent with an acute loss of blood flow to a peripheral artery (or arteries), which is supported by evidence of embolism from surgical specimens, autopsy, angiography, or other objective testing. [2]

[1] Thygesen K, Alpert JS, Jaffe AS, Simoons ML, Chaitman BR, White HD; Joint ESC/ACCF/AHA/WHF Task Force for the Universal Definition of Myocardial Infarction, Katus HA, Lindahl B, Morrow DA, Clemmensen PM, Johanson P, Hod H, Underwood R, Bax JJ, Bonow RO, Pinto F, Gibbons RJ, Fox KA, Atar D, Newby LK, Galvani M, Hamm CW, Uretsky BF, Steg PG, Wijns W, Bassand JP, Menasché P, Ravkilde J, Ohman EM, Antman EM, Wallentin LC, Armstrong PW, Simoons ML, Januzzi JL, Nieminen MS, Gheorghiade M, Filippatos G, Luepker RV, Fortmann SP, Rosamond WD, Levy D, Wood D, Smith SC, Hu D, Lopez-Sendon JL, Robertson RM, Weaver D, Tendera M, Bove AA, Parkhomenko AN, Vasilieva EJ, Mendis S. Third universal definition of myocardial infarction. *Circulation.* 2012 Oct 16;126(16):2020-35.

[2] http://www.nejm.org/doi/suppl/10.1056/NEJMoa1107039/suppl_file/nejmoa1107039_protocol.pdf Accessed July 29 2015.

Appendix I: Bleeding outcome definitions[1]

Acute clinically overt bleeding is defined as new onset, visible bleeding, signs, and/or symptoms suggestive of bleeding with confirmatory imaging techniques which can detect the presence of blood (e.g. ultrasound, computed tomography, magnetic resonance imaging)

The definition of ***major bleeding*** is adapted from the International Society on Thrombosis and Hemostasis (ISTH) definition[2].

Major bleeding is defined as a bleeding event which is:

- Acute clinically overt bleeding accompanied by one or more of the following:
  - A decrease in hemoglobin (Hgb) of 2 g/dL or more
  - A transfusion of 2 or more units of packed red blood cells
  - Bleeding that occurs in at least one of the following critical sites:
    - Intracranial
    - Intra-spinal
    - Intraocular (within the corpus of the eye; thus, a conjunctival bleed is not an intraocular bleed)
    - Pericardial
    - Intra-articular
    - Intramuscular with compartment syndrome
    - Retroperitoneal.
- Bleeding that is fatal.

Clinically relevant non-major bleeding event: The definition of clinical relevant non-major bleeding will be acute or sub-acute clinically overt bleeding that does not satisfy the criteria for major bleeding and that leads to one or more of the following:

1) hospital admission for bleeding.
2) physician-guided medical or surgical treatment for bleeding.
3) a change in antithrombotic therapy.

Minor bleeding events: All acute clinically overt bleeding events not meeting the criteria for either major bleeding or clinically relevant non-major bleeding will be classified as minor bleeding.

Fatal bleeding event is defined as a bleeding event that the adjudication committee determines to be the primary cause of death or contributes directly to death.

All acute clinically overt bleeding events will be adjudicated as a major bleeding event, or clinically relevant non-major bleeding event. Minor bleeding events will not be adjudicated.

[1]http://www.nejm.org/doi/suppl/10.1056/NEJMoa1107039/suppl_file/nejmoa1107039_protocol.pdf Accessed July 31 2015.

[2]Schulman S, Kearon C; Subcommittee on Control of Anticoagulation of the Scientific and Standardization Committee of the International Society on Thrombosis and Haemostasis.Definition of major bleeding in clinical investigations of antihemostatic medicinal products in non-surgical patients. *J Thromb Haemost*. 2005;3:692-694.

Appendix J: Serious adverse outcome collection and reporting.

ADVERSE EVENT REPORTING

All Serious Adverse Events (SAEs) that occur following the subject's written consent to participate in the study through 30 days of discontinuation of dosing must be reported to reported to the study sponsor.

Adverse Events

- An Adverse Event [AE] is defined as any new untoward medical occurrence or worsening of a pre-existing medical condition in a patient or clinical investigation subject administered a medicinal product and that does not necessarily have a causal relationship with this treatment. An AE can therefore be any unfavorable and unintended sign (including an abnormal laboratory finding, for example), symptom, or disease temporally associated with the use of a medicinal product, whether or not considered related to the investigational product.
- The causal relationship to a medicinal product is determined by a physician and should be used to assess all adverse events (AEs). The causal relationship can be one of the following:
  - Related: There is a reasonable causal relationship between a medicinal product and the AE.
  - Not Related: There is not a reasonable causal relationship between a medicinal product and the AE.
- The term "reasonable causal relationship" means there is evidence to suggest a causal relationship.
- Adverse events can be spontaneously reported or elicited during open-ended questioning, examination, or evaluation of a subject. (In order to prevent reporting bias, subjects should not be questioned regarding the specific occurrence of one or more adverse events).

Serious Adverse Events

A **Serious Adverse Event (SAE)** is any untoward medical occurrence at any dose that:

- results in death
- is life-threatening (defined as an event in which the subject was at risk of death at the time of the event; it does not refer to an event which hypothetically might have caused death if it were more severe)
- requires inpatient hospitalization or causes prolongation of existing hospitalization (see ***NOTE****: below for exceptions)
- results in persistent or significant disability/incapacity
- is a congenital anomaly/birth defect
- is an important medical event, defined as a medical event that may not be immediately life-threatening or result in death or hospitalization but, based on appropriate medical and

scientific judgment, may jeopardize the subject or may require intervention (e.g., medical, surgical) to prevent one of the other serious outcomes listed above. Examples of such events include but are not limited to intensive treatment in an emergency department or at home for allergic bronchospasm; blood dyscrasias or convulsions that do not result in hospitalization.

Suspected transmission of an infectious agent (eg, pathogenic or non-pathogenic) via a medicinal product is an SAE.

Although pregnancy, overdose, adverse events of special interest, and cancer are not always serious by regulatory definition, these events must be handled as SAEs.

Any component of a study endpoint that is considered related to study therapy should be reported as an SAE (eg, death is an endpoint, if death occurred due to anaphylaxis, anaphylaxis must be reported).

***NOTE:** *The following hospitalizations are not considered SAEs:*

- A visit to the emergency room or other hospital department lasting less than 24 hours that does not result in admission (unless considered an "important medical event" or a life-threatening event)
- Elective surgery planned before signing consent
- Admissions as per protocol for a planned medical/surgical procedure
- Routine health assessment requiring admission for baseline/trending of health status (eg, routine colonoscopy)
- Medical/surgical admission other than remedying ill health state that was planned before study entry. Appropriate documentation is required in these cases
- Admission encountered for another life circumstance that carries no bearing on health status and requires no medical/surgical intervention (eg, lack of housing, economic inadequacy, caregiver respite, family circumstances, administrative reason)

Adverse Events of Special Interest

In this study, the following adverse events are to be reported to BMS as serious events, regardless of whether these reports are classified as serious or unexpected:

Potential or suspected cases of liver injury including but not limited to liver test abnormalities, jaundice, hepatitis or cholestasis.

Serious Adverse Event Collecting and Reporting

Following the subject's written consent to participate in the study, all SAEs, whether related or not related to a medicinal product, must be collected, including those thought to be associated with protocol-specified procedures. SAEs must be recorded on CIOMS Form and reported to BMS within 3 business days to comply with regulatory requirements. A form should be completed for any event where doubt exists regarding its status of seriousness. Although overdose and cancer are not always serious by regulatory definition, these events should be recorded on a form and reported to BMS within 3 business days. All SAEs must be reported by confirmed facsimile (fax) transmission or reported via electronic mail to:

SAE Email Address: Worldwide.Safety@BMS.com
SAE Facsimile Number: 1-609-818-3804

If only limited information is initially available, follow-up reports may be required.

All SAEs must be collected that occur during the screening period and within 30 days of discontinuing dosing of a medicinal product. If applicable, SAEs must be collected that relate to any later protocol-specific procedure (such as follow-up skin biopsy).
The investigator should report any SAE occurring after these time periods that is believed to be related to a medicinal product or protocol-specified procedure.

An SAE report should be completed for any event where doubt exists regarding its status of seriousness.

If the investigator believes that an SAE is not related to a medicinal product, but is potentially related to the conditions of the study (such as withdrawal of previous therapy, or a complication of a study procedure), the relationship should be specified in the narrative section of the SAE Report Form.

SAEs, whether related or unrelated to a medicinal product, and pregnancies must be reported to the study sponsor within 1 business day. SAEs must be reported initially through the electronic case report form (eCRF); if necessary, the central coordinating centre will follow up to ask sites to record the SAEs on the CIOMS Report Form. Pregnancies will be reported on a Pregnancy Surveillance Form.

If only limited information is initially available, follow-up reports are required. (Note: Follow-up SAE reports should include the same investigator term(s) initially reported.)

If an ongoing SAE changes in its intensity or relationship to a medicinal product or if new information becomes available, a follow-up SAE report should be sent within 1 business day to the study sponsor (or designee) using the same procedure used for transmitting the initial SAE report.

All SAEs should be followed to resolution or stabilization.

SAE Reconciliation for SAEs related to BMS products

The sponsor will reconcile the clinical database SAE cases transmitted to BMS Global Pharmacovigilance (GPV&E). Frequency of reconciliation will be done every three months and once prior to study database lock. BMS GPV&E will e-mail upon request from the sponsor, the GPV&E reconciliation report. Requests for reconciliation should be sent to aepbusinessprocess@bms.com. The data elements listed on the GPV&E reconciliation report will be used for case identification purposes. If the investigator determines a case was not transmitted to BMS GPV&E, the case will be sent immediately.

Health Authority Reporting (Worldwide)

Investigators must adhere to local Health Authority Reporting Requirements and timelines. For studies conducted under a local health authority:

- Adverse drug reactions that are Serious, Unexpected, and at least Possibly Related to the drug (Suspected Unexpected Serious Adverse Reaction, SUSAR) and that have not previously been reported in the Investigators' Brochure, or reference safety information document will be reported promptly, within local reporting timelines, to the health authority in writing by the Investigator.
- A clear description of the suspected reaction should be provided along with an assessment as to whether the event is drug or disease related.
- The Investigator shall notify the health authority of any unexpected fatal or life threatening experience associated with the use of the drugs as soon as possible but no later than 7 calendar days after initial receipt of the information.

All SAEs should be reported to the study sponsor by entering the data into the SAE form of the eCRF. This will trigger an email notification to the coordination centre and sponsor. Site staff will also be required to print the SAE form and fax it to the Coordination Centre at 416-864-3016.

Unusual failure in efficacy
Any unusual failure in efficacy of any BMS marketed drug product prescribed to the patient must be reported to the coordination centre within 1 business day of becoming aware of the event using a CIOMS form (https://cioms.ch/wp-content/uploads/2017/05/cioms-form1.pdf). The coordinating centre will report these events to BMS Pharmacovigilance within 2 business days of becoming aware to:

SAE Email Address: Worldwide.Safety@BMS.com
SAE Facsimile Number: 1-609-818-3804

To further define an unusual failure in efficacy: The underlying principle is that if a health product fails to produce the expected intended effect, there may be an adverse outcome for the patient, including an exacerbation of the condition for which the health product is being used. Clinical judgment should be exercised to determine if the problem reported is related to the product itself, rather than one of treatment selection or disease progression, since health products cannot be expected to be effective in 100% of the patients. One example of unusual failure in efficacy is a previously well-stabilized condition that deteriorates when the patient changes to a different brand or receives a new prescription. Another example of a case that should be reported on an expedited basis is a life-threatening infection where the failure in efficacy seems to be due to the development of a newly resistant strain of bacterium previously regarded as susceptible.

Non-Serious Adverse Events

A nonserious adverse event is an AE not classified as serious.

Non-Serious Event Collecting and Reporting

The collection of non-serious adverse event (NSAE) information should begin at initiation of a medicinal product. Nonserious adverse event information should also be collected from the start of a placebo lead-in period or other observational period intended to establish a baseline status for the subjects.

Nonserious AEs should be followed to resolution or stabilization, or reported as SAEs if they become serious. Follow-up is also required for nonserious AEs that cause interruption or discontinuation of a medicinal product, or those that are present at the end of study treatment as appropriate.

Nonserious Adverse Events related to any BMS product are provided to BMS via annual safety reports (if applicable), and interim or final study reports.

Laboratory Test Abnormalities

The following laboratory abnormalities should be captured and reported as appropriate:

- Any laboratory test result that is clinically significant or meets the definition of an SAE
- Any laboratory test result abnormality that required the subject to have a medicinal product discontinued or interrupted
- Any laboratory test result abnormality that required the subject to receive specific corrective therapy.

It is expected that wherever possible, the clinical rather than the laboratory term will be used by the reporting investigator (eg, use the term anemia rather than low hemoglobin value).

Laboratory test abnormalities are provided to the study sponsor via annual safety reports (if applicable), and interim or final study reports.

Pregnancy

If, following initiation of the investigational product, it is subsequently discovered that a study subject is pregnant or may have been pregnant at the time of medicinal product exposure, including during at least 5 half-lives after medicinal product administration, the medicinal product will be permanently discontinued in an appropriate manner (eg, dose tapering if necessary for subject safety).

The investigator must immediately notify the study sponsor of this event via the Pregnancy Surveillance Form within 24 hours and in accordance with SAE reporting procedures.

Follow-up information regarding the course of the pregnancy, including perinatal and neonatal outcome and, where applicable, offspring information must be reported on a Pregnancy Surveillance Form.

Any pregnancy that occurs in a female partner of a male study participant should be reported to the study sponsor. Information on this pregnancy may also be collected on the Pregnancy Surveillance Form.

Protocol-required procedures for study discontinuation and follow-up must be performed on the subject unless contraindicated by pregnancy (eg, x-ray studies). Other appropriate pregnancy follow-up procedures should be considered if indicated.

Overdose

Include any product-specific definition of overdose in addition to the following mandatory statement as the last sentence.

An overdose is defined as the accidental or intentional administration of any dose of a medicinal product that is considered both excessive and medically important. All occurrences of overdose must be reported as SAEs.

Other Safety Considerations

Any significant worsening noted during interim or final physical examinations, electrocardiograms, x-rays, and any other potential safety assessments, whether or not these procedures are required by the protocol, should also be recorded as a nonserious or serious adverse event, as appropriate, and reported accordingly.

Appendix K: The CHA_2DS_2-VASC score[1]

	Clinical feature	Score
C	Congestive heart failure (or LVEF ≤40%)	1
H	Hypertension	1
A	Age ≥ 75 years	2
D	Diabetes	1
S	Stroke / TIA / Systemic arterial embolism	2
V	Vascular disease (e.g. CAD, PAD, aortic plaque)	1
A	Age between 65-74 years	1
Sc	Sex category: Female	1
	**Maximum score**	**10**

CAD = coronary artery disease; LVEF = left ventricular ejection fraction; PAD = peripheral arterial disease; TIA = transient ischemic attack.

[1]European Heart Rhythm Association; European Association for Cardio-Thoracic Surgery, Camm AJ, Kirchhof P, Lip GY, Schotten U, Savelieva I, Ernst S, Van Gelder IC, Al-Attar N, Hindricks G, Prendergast B, Heidbuchel H, Alfieri O, Angelini A, Atar D, Colonna P, De Caterina R, De Sutter J, Goette A, Gorenek B, Heldal M, Hohloser SH, Kolh P, Le Heuzey JY, Ponikowski P, Rutten FH. Guidelines for the management of atrial fibrillation: the Task Force for the Management of Atrial Fibrillation of the European Society of Cardiology (ESC).*Eur Heart J.* 2010;31:2369-2429.

MEMO

SEARCH-AF

To: All site investigators and coordinators of the SEARCH-AF study

Subject: PROTOCOL CLARIFICATION

Date: Friday December 16, 2016

The purpose of this memorandum is to update the following in the SEARCH-AF study protocol (Version Number: 1.0, Version Date: 18 December, 2015):

1. Page 22 – Section 5.6.1, under the sub-heading "Data Reporting", states that "*A summary report with accompanying rhythm strips will be sent to the treating physician and the trial coordination centre* ***on a weekly basis.***"

   AND

   Page 42 – Appendix C states "*We will receive "End of Use Summary Reports"* ***on a weekly basis***" and "*Arrhythmic episodes meeting "Notification Criteria" will be included in the End of Use Summary report which is generated* ***on a weekly basis.***"

   In place of "on a weekly basis", these sentences should instead read "at the end of the 30-day or 14-day monitoring period." Medtronic will provide summary reports at the end of each study subject's monitoring period.

   Please note that episodes of atrial fibrillation or atrial flutter which are detected by the SEEQ™ device will be transmitted to the investigational and central coordinating sites within 4 hours of receipt by the Medtronic monitoring center. This means that this information (detected atrial fibrillation/flutter) will be received by the investigational site before receipt of the summary report/End of Use report.

2. A discrepancy exists in the creatinine clearance value of Inclusion Criterion 3)(iii):

   On page 8 it is stated as: "**<60 ml/min**"

   On page 18 it is stated as: "**<60 ml/min/1.73m^{2}**"

   Please note that either value is acceptable. The former is the Cockcroft-Gault formula and the latter is derived from the CKD-EPI formula. Please use whichever calculation is preferred by your institution's laboratory.

Please file this memo with your SEARCH-AF regulatory files. The above information will be incorporated into the next version of the protocol when a full amendment is forthcoming.

MEMO

SEARCH-AF

To: All site investigators and coordinators of the SEARCH-AF study

Subject: PROTOCOL CLARIFICATION

Date: Monday February 6, 2017

The purpose of this memorandum is to clarify the following in the SEARCH-AF study protocol (Version Number: 1.0, Version Date: 18 December, 2015):

The primary and secondary study '*objectives*' are listed in two areas of the protocol:

- Page 7
- Page 15

The primary and secondary study '*outcomes*' are listed on:

- Page 27

Please note that there are slight wording differences between the listed '*objectives*' and '*outcomes*', and that **all study endpoints will be measured in accordance to the definitions on page 27.**

Please file this memo with your SEARCH-AF regulatory files.

SEARCH-AF Protocol Summary of Changes (Document date: 02-Feb-2018)

Amendment 1: Version 1.0 (18-Dec-2015) vs. Version 2.0 (02-Feb-2018)

Protocol Section		Description of Changes	Reason / Rationale
Page 8	Protocol Synopsis - Inclusion Criteria 3iii	**Previous wording:** "Impaired renal function (defined as creatinine clearance <60 ml/min)"<br><br>**Revised wording:** "Impaired renal function (defined as creatinine clearance <60 ml/min or <60 ml/min/1.73m^2)."	Both types of creatinine clearance values have been added for clarity. The first calculation is the Cockcroft-Gault formula and the second is derived from the CKD-EPI formula. Both calculations are considered acceptable.
Page 10	Protocol Synopsis – Follow Up	**Previous wording:** "All subjects will have a follow-up visit at 45-52 days after hospital discharge and at 6±1 months after surgery.<br><br>**Revised wording**: "All subjects will have a follow-up visit at 31-90 days after hospital discharge and at 6±1 months after surgery."	The Follow-Up Visit 1 window has expanded from 45-52 days after hospital discharge to 31-90 days after hospital discharge to better accommodate patients and facilitate visit compliance.
Page 18	Eligibility Criteria – Inclusion Criteria 3iii	**Previous wording:** "Impaired renal function (defined as creatinine clearance <60 ml/min/1.73m^2)"<br><br>**Revised wording:** "Impaired renal function (defined as creatinine clearance <60 ml/min or <60 ml/min/1.73m^2)."	Both types of creatinine clearance values have been added for clarity. The first calculation is the Cockcroft-Gault formula and the second is derived from the CKD-EPI formula. Both calculations are considered acceptable.
Page 21	Study Design - Randomization	**Previous wording:** "If a subject is randomized to the intervention group, the device will be fitted onto the subject on the same day of randomization."<br><br>**Revised wording:** "If a subject is randomized to the intervention group, the device will be fitted onto the subject within 24 hours of randomization."	The time frame has been expanded to better accommodate for patient discharge from the hospital, and allows added flexibility for research coordinators to ensure adequate time to meet with the patient.
Page 22	Study Design – Proposed Study Intervention – *Data Reporting*	**Previous wording:** "A summary report with accompanying rhythm strips will be sent to the treating physician and the trial coordination centre on a weekly basis.<br><br>**Revised wording:** "A summary report with accompanying rhythm strips will be sent to the treating physician and the trial coordination	Medtronic will provide weekly summary reports in addition to End of Use reports at the end of each study subject's monitoring period.

Protocol Section		Description of Changes	Reason / Rationale
		centre on a weekly basis as well as after the conclusion of the 30-day or 14-day monitoring period.”	
Page 25	Proposed Frequency and Duration of Follow Up – Visit #1	**Previous wording:** “Visit #1 (45-52 days after discharge): All subjects will be assessed during follow-up at 31-90 days after discharge from their index cardiac surgery.”<br><br>**Revised wording:** “Visit #1 (31-90 days after discharge): All subjects will be assessed during follow-up at 31-90 days after discharge from their index cardiac surgery.”	The Follow-Up Visit 1 window has expanded from 45-52 days after hospital discharge to 31-90 days after hospital discharge to better accommodate patients and facilitate visit compliance.
Page 26	Study Flowchart	**Previous wording:** “Visit #1 (45-52 days after discharge)”<br><br>**Revised wording:** “Visit #1 (31-90 days after discharge)”	The Follow-Up Visit 1 window has expanded from 45-52 days after hospital discharge to 31-90 days after hospital discharge to better accommodate patients and facilitate visit compliance.
Page 28	Other Considerations	**Previous wording:** “In addition, all efforts will be made to ensure that subjects are assessed at follow-up at 45-52 days after discharge from their index cardiac surgery.”<br><br>**Revised wording:** “In addition, all efforts will be made to ensure that subjects are assessed at follow-up at 31-90 days after discharge from their index cardiac surgery.”	The Follow-Up Visit 1 window has expanded from 45-52 days after hospital discharge to 31-90 days after hospital discharge to better accommodate patients and facilitate visit compliance.
Page 29	Study duration – Expected study duration of subject population	**Previous wording:** “The first follow-up visit will occur at 45-52 days after discharge from cardiac surgery and the second follow-up visit will occur at 6±1 months after discharge from cardiac surgery.”<br><br>**Revised wording:** “The first follow-up visit will occur at 31-90 days after discharge from cardiac surgery and the second follow-up visit will occur at 6±1 months after discharge from cardiac surgery.”	The Follow-Up Visit 1 window has expanded from 45-52 days after hospital discharge to 31-90 days after hospital discharge to better accommodate patients and facilitate visit compliance.

SEARCH-AF Protocol Summary of Changes (Document date: 02-Feb-2018)

Amendment 1: Version 1.0 (18-Dec-2015) vs. Version 2.0 (02-Feb-2018)

Protocol Section		Description of Changes	Reason / Rationale
Page 42	Appendix C	**Previous wording:** "We will receive "End of Use Summary Reports" on a weekly basis."<br>a*nd*<br>"Arrhythmic episodes meeting "Notification Criteria" will be included in the End of Use Summary report which is generated on a weekly basis."<br><br>**Revised wording:** "We will receive "End of Use Summary Reports" on a weekly basis as well as after the conclusion of the 30-day or 14-day monitoring period."<br>a*nd*<br>"Arrhythmic episodes meeting "Notification Criteria" will be included in the End of Use Summary report which is generated on a weekly basis as well as after the conclusion of the 30-day or 14-day monitoring period."	Medtronic will provide weekly summary reports in addition to End of Use reports at the end of each study subject's monitoring period.
Pages 44-53	Appendix D	The formatting of the End of Use Summary Report and Episode Report has been updated.	Medtronic has updated the formatting of their reports. Sample reports are provided.
Page 61	Appendix H	A section on 'Adverse Events of Special Interest' has been added to replace language regarding Drug Induced Liver Injury (DILI).	This change was made to better align with BMS' global reporting requirements and incorporates reporting of potential or suspected cases of liver injury.

Protocol Section		Description of Changes	Reason / Rationale
Page 63	Appendix H	A section on the reporting of any ‘Unusual Failure in Efficacy’ has been added to the protocol.	This section was added in order to comply with Health Canada regulations (Part C, Division 8 of the Food and Drug Regulations)
Page 66	Appendix I	CHA_2DS_2-VASC score table has been updated.	The “S” feature of the CHA_2DS_2-VASC score has been clarified to include “Stroke, TIA and Systemic arterial embolism”.<br><br>The “V” feature of the CHA_2DS_2-VASC score has been updated with specific vascular diseases “CAD, PAD and aortic plaque”.

SEARCH-AF Protocol Summary of Changes (Document date: Oct 1 2018)

Amendment 2: Version 2.0 (02-Feb-2018) vs. Version 3.0 (01-Oct-2018)

Medtronic's SEEQ™ monitoring program will be phased out in order for the company to focus on other rhythm monitoring initiatives. This program will end on October 1, 2018.

CardioSTAT is a patch-based rhythm monitor developed and manufactured by Icentia Inc. This device is licensed and commercially available in Canada. Given its high-quality recordings as well as it's similarities to the SEEQ™ devices, CardioSTAT was selected as the new monitoring device to be used for the remainder of the SEARCH-AF trial. Below is a list of amendments that have been made to the protocol to reflect this change:

Protocol Section		Description of Changes	Reason / Rationale
Page 7	Protocol Synopsis – Study Objective	**Previous wording:** "To assess subjects' adherence and incidence of adverse events from use of the Medtronic SEEQ system"<br><br>**Revised wording:** "To assess subjects' adherence and incidence of adverse events from use of a wearable adhesive cardiac rhythm monitoring system."	Medtronic's SEEQ program is being phased out.
Page 9-10	Protocol Synopsis – Intervention	**Previous wording:** "Starting on the day of randomization, subjects will undergo 30 days of continuous cardiac rhythm monitoring with the Medtronic SEEQ™ mobile cardiac telemetry system."<br><br>**Revised wording**: "Starting on the day of randomization, subjects will undergo a maximum of 30 days of continuous cardiac rhythm monitoring with a wearable adhesive cardiac rhythm monitoring system."	Each CardioSTAT device records 14 days of data. During the initial 4-week monitoring, patients will be provided 2 CardioSTAT devices for 28 days of monitoring.
Page 10	Protocol Synopsis – Intervention	**Previous wording:** "At 6±1 months after surgery, subjects in both groups will undergo 14 days of continuous cardiac rhythm monitoring with the SEEQ™ mobile cardiac telemetry system."<br><br>**Revised wording**: "At 6±1 months after surgery, subjects in both groups will undergo 14 days of continuous cardiac rhythm monitoring with a wearable adhesive cardiac rhythm monitoring system."	Medtronic's SEEQ program is being phased out.
Page 10	Protocol Synopsis – Study flow chart	**Previous wording:**	Medtronic's SEEQ program is being phased out.<br><br>Each CardioSTAT device records 14 days of data. During the initial 4-week monitoring,

SEARCH-AF Protocol Summary of Changes (Document date: Oct 1 2018)

Amendment 2: Version 2.0 (02-Feb-2018) vs. Version 3.0 (01-Oct-2018)

<table>
<tr><th colspan="2">Protocol Section</th><th>Description of Changes</th><th>Reason / Rationale</th></tr>
<tr><td></td><td></td><td>
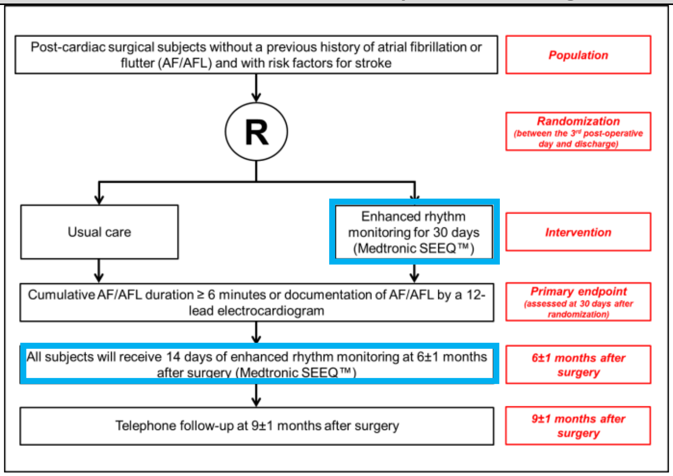

Revised wording:

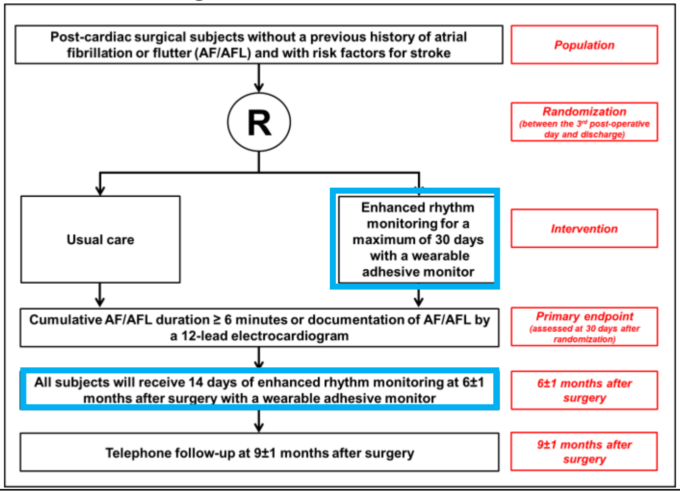

</td><td>patients will be provided 2 CardioSTAT devices for a maximum of 28 days of monitoring.</td></tr>
</table>

SEARCH-AF Protocol Summary of Changes (Document date: Oct 1 2018)

Amendment 2: Version 2.0 (02-Feb-2018) vs. Version 3.0 (01-Oct-2018)

Protocol Section		Description of Changes	Reason / Rationale
Page 13	Background and Rationale – Section 1.7	**Previous wording:** "This trial will afford the opportunity to evaluate a novel diagnostic tool for AF detection over a short-term period."<br><br>**Revised wording:** "This trial will afford the opportunity to evaluate a wearable adhesive cardiac rhythm monitoring system for AF detection over a short-term period."	The text has been updated to provide more specificity and clarify.
Page 14	Background and Rationale – Section 1.7	**New text: "**As of October 1 2018, the CardioStat cardiac rhythm monitoring system (Icentia Inc., Quebec, Canada) will replace the Medtronic SEEQ™ device when subjects undergo protocol-mandated cardiac rhythm monitoring procedures (appendix E-F). This is a wearable adhesive cardiac rhythm monitoring device which is applied over the subject's anterior chest wall. Upon completion of the monitoring period, the device will be mailed back to the manufacturer (Icentia Inc., Quebec, Canada) for data extraction. Results will be available to the site investigator for review within 10 business days upon receipt of the device."	Medtronic's SEEQ program is being phased out. New text has been added to describe Icentia's CarioSTAT device.
Page 14	Background and Rationale – Section 1.8	**Previous wording:** "The PUBMED search strategy was described in appendix E and is current as of May 13 2015."<br><br>**Revised wording:** "The PUBMED search strategy was described in appendix G and is current as of May 13 2015."	List of Appendices have been updated.
Page 14	Background and Rationale – Section 1.8	**Previous wording:** "We identified 4 studies and they are described in appendix E."<br><br>**Revised wording:** "We identified 4 studies and they are described in appendix G."	List of Appendices have been updated.
Page 20	Section 5.1 - Study Description	**Previous wording:** "Half of the study population will be randomly allocated to a 30-day adhesive cardiac event monitoring system (intervention group)…"<br><br>**Revised wording:** "Half of the study population will be randomly allocated to a wearable adhesive cardiac rhythm monitoring system for up to 30 days (intervention group)…"	Each CardioSTAT device records 14 days of data. During the initial 4-week monitoring, patients will be provided 2 CardioSTAT devices for a maximum of 28 days of monitoring.

SEARCH-AF Protocol Summary of Changes (Document date: Oct 1 2018)

Amendment 2: Version 2.0 (02-Feb-2018) vs. Version 3.0 (01-Oct-2018)

Protocol Section		Description of Changes	Reason / Rationale
Page 20	Section 5.3 - Clinical Events Committee	**Previous wording:** "…full-disclosure SEEQ™ recordings (if necessary)…"<br><br>**Revised wording:** "…full-disclosure recordings from the wearable adhesive cardiac rhythm monitoring system (if necessary)…"	Medtronic's SEEQ program is being phased out.
Page 21	Section 5.5 - Randomization	**Previous wording:** "…subjects will be randomly allocated in a 1:1 ratio to the intervention arm (30 days of cardiac rhythm monitoring with an adhesive cardiac telemetry system…)"<br><br>**Revised wording:** "…subjects will be randomly allocated in a 1:1 ratio to the intervention arm (up to 30 days of cardiac rhythm monitoring with a wearable adhesive cardiac rhythm monitoring system…)"	Each CardioSTAT device records 14 days of data. During the initial 4-week monitoring, patients will be provided 2 CardioSTAT devices for a maximum of 28 days of monitoring.
Page 21	Section 5.6 – Proposed Study Intervention	**Previous wording:** "Continuous cardiac rhythm monitoring for 30 days…"<br><br>**Revised wording:** "Continuous cardiac rhythm monitoring for up to 30 days…"	Each CardioSTAT device records 14 days of data. During the initial 4-week monitoring, patients will be provided 2 CardioSTAT devices for a maximum of 28 days of monitoring.
Page 21	Section 5.6.1 heading	**Previous wording:** "30-day continuous cardiac rhythm monitor early after surgery (Intervention group)"<br><br>**Revised wording:** "Continuous cardiac rhythm monitor early after surgery (Intervention group)"	Each CardioSTAT device records 14 days of data. During the initial 4-week monitoring, patients will be provided 2 CardioSTAT devices for a maximum of 28 days of monitoring.
Page 21	Section 5.6.1	**New text:** "From the start of the study to September 30 2018, the device to be used was the Medtronic SEEQ™ mobile cardiac telemetry system (appendix A-D). Starting on October 1 2018, the CardioStat system will be used for subjects who initiate protocol-mandated rhythm monitoring in this study (appendix E-F).<br><br>In May 2018, the SEARCH-AF team was informed by Medtronic that the SEEQ™ mobile cardiac telemetry system would no longer be available for research purposes as of September 30 2018. As a result, an alternative wearable adhesive cardiac rhythm monitoring system (CardioStat, manufactured by Icentia Inc., Quebec, Canada) was chosen to replace the SEEQ™ monitoring system for all subsequent protocol-mandated cardiac rhythm monitoring events.	Text has been added to indicate the start and end dates of when the Medtronic SEEQ devices were used for this trial.

SEARCH-AF Protocol Summary of Changes (Document date: Oct 1 2018)

Amendment 2: Version 2.0 (02-Feb-2018) vs. Version 3.0 (01-Oct-2018)

Protocol Section		Description of Changes	Reason / Rationale
Page 22	Section 5.6.1	**New text:** "Starting on October 1 2018, the CardioStat cardiac rhythm monitoring system (Icentia Inc., Quebec, Canada) was selected to replace the SEEQ™ system for all subsequent cardiac rhythm monitoring events as mandated by the protocol. The CardioStat device is a wearable adhesive cardiac rhythm monitoring system which is low-profile, water-resistant, and is adhered onto the skin surface with 2 electrodes. This wearable adhesive device is designed for one-time use only. Each CardioStat device will provide 14 days of cardiac rhythm monitoring. For subjects randomized to the intervention group who will be monitored by the CardioStat device, they will receive 28 days of continuous cardiac rhythm monitoring."	Text has been added to describe the CardioStat device.
Page 22	Section 5.6.1	**New text:** "For subjects who undergo cardiac rhythm monitoring with the CardioStat monitoring system, no transmitter will be required since all cardiac rhythm monitoring will be stored on the device itself."	Text has been added to indicate that CardioStat will not require the transmitter.
Page 22	Section 5.6.1	**New text:** "Medtronic SEEQ™ mobile cardiac telemetry system"	Subsection heading added for clarity.
Page 23	Section 5.6.1	**New text:** "<u>CardioStat cardiac rhythm monitoring system</u><br>The cardiac rhythm data will be collected by the CardioStat device and the data will not be wirelessly transmitted. Once the subject completes the monitoring period, the device will be mailed back to Icentia Inc. (Quebec, Canada) who will process the device and retrieve the rhythm data. Trained personnel will then examine the raw cardiac rhythm data and provide information on the presence of AF or AFL, the duration of each AF/AFL episode, as well as the total duration of AF/AFL during the monitoring period. A list of reportable cardiac rhythm parameters is shown in Appendix E-F. Once the cardiac rhythm data is analyzed, a summary report will be generated. This can be accessed by the investigators via a web-based, password-protected interface. Subjects will be identified by a unique study number which does not contain any personal identification information. The CardioStat cardiac rhythm monitoring device is approved for clinical use in Canada."	Text has been added to describe cardiac monitor by the CardioStat device.
Page 23	Section 5.6.1	**New text:** "Medtronic SEEQ™ mobile cardiac telemetry system:"	Subsection heading added for clarity.

SEARCH-AF Protocol Summary of Changes (Document date: Oct 1 2018)

Amendment 2: Version 2.0 (02-Feb-2018) vs. Version 3.0 (01-Oct-2018)

Protocol Section		Description of Changes	Reason / Rationale
Page 24	Section 5.6.1	**Next text:** "CardioStat cardiac rhythm monitoring system<br>Each wearable sensor will provide 14 days of continuous cardiac rhythm monitoring. As such, subjects who are randomized to the intervention arm will receive 2 CardioStat devices. The first application will be done during hospitalization, under the supervision of the research coordinator. During the first sensor application, the subject will be educated on the appropriate use of this device, including the following:<br>1) Proper placement of the adhesive sensor.<br>2) Self-activation of the sensor for symptomatic episodes.<br><br>For subjects randomized to the intervention group, the research coordinator will contact them after 2 weeks by telephone to reinforce education of the proper use of the device (if necessary). Adverse outcomes related to the use of the study device will be assessed and an in-person clinic visit may be arranged if required."	Text has been added to describe patient education around the CardioStat device.
Page 24	Section 5.6.3	**Previous wording:** "At the 6±1 month follow-up visit, all subjects (in the intervention group and the usual care group) will undergo 14 days of continuous cardiac rhythm monitoring with the SEEQ™ mobile cardiac telemetry system. This will involve application of 2 SEEQ™ monitors over the 14-day monitoring period. The first (of two) SEEQ™ monitor will be applied onto the subject at the 6±1 month visit. At this visit, subjects will be educated on the use and maintenance of the SEEQ™ monitor. Please refer to section 5.6.1 for details on this aspect."<br><br>**Revised wording:** "At the 6±1 month follow-up visit, all subjects (in the intervention group and the usual care group) will undergo 14 days of continuous cardiac rhythm monitoring with a wearable adhesive cardiac rhythm monitoring device. This will involve application of 2 SEEQ™ monitors over the 14-day monitoring period. As of October 1 2018, the CardioStat device will replace the SEEQ™ mobile cardiac telemetry system."	Wording around the 14-day monitoring period has been revised to also describe the use of the CardioStat device.
Page 25	Section 5.7	**New text:** "Atrial arrhythmias detected by the CardioStat cardiac rhythm monitoring system will be initially interpreted by trained technicians employed by Icentia Inc. who are not involved in any part of the study design."	Text has been added to indicate that Icentia technicians who review monitoring reports were not part of the design of the study

Protocol Section		Description of Changes	Reason / Rationale
		**New text:** "Medtronic or Icentia Inc. …"	
Page 26	Section 5.8	**Previous wording:** "…they will undergo 30 days of continuous cardiac rhythm monitoring with the Medtronic SEEQ™ mobile cardiac telemetry system starting on the day of randomization. At 6±1 months after discharge from the index cardiac surgery, they will undergo another 14 days of cardiac rhythm monitoring with the SEEQ™ device.<br>Subjects in the usual care group (control group) will only undergo protocol-mandated cardiac rhythm monitoring at 6±1 months after discharge. At that time, they will undergo 14 days of cardiac rhythm monitoring with the SEEQ™ device."<br><br>**Revised wording:** "…they will undergo up to 30 days of continuous cardiac rhythm monitoring with a wearable adhesive device starting on the day of randomization. At 6±1 months after discharge from the index cardiac surgery, they will undergo another 14 days of cardiac rhythm monitoring with a wearable adhesive device.<br>Subjects in the usual care group (control group) will only undergo protocol-mandated cardiac rhythm monitoring at 6±1 months after discharge. At that time, they will undergo 14 days of cardiac rhythm monitoring with a wearable adhesive cardiac rhythm monitoring device. "	Medtronic's SEEQ program is being phased out.<br><br>Each CardioSTAT device records 14 days of data. During the initial 4-week monitoring, patients will be provided 2 CardioSTAT devices for a maximum of 28 days of monitoring.
Page 26	Section 5.9	**Previous wording:** "At this visit, all subjects will receive 14 days of continuous cardiac rhythm monitoring with the SEEQ™ device.<br><br>**Revised wording:** "At this visit, all subjects will receive 14 days of continuous cardiac rhythm monitoring with a wearable adhesive cardiac monitoring device."	Medtronic's SEEQ program is being phased out.
Page 27	Section 5.10 – Study flowchart	**New text: "**Education of device application (and maintenance of data transmitter for subjects allocated to the SEEQ™ device)"	Text has been added to clarify tasks to be completed when using the SEEQ device versus the CardioStat device.

SEARCH-AF Protocol Summary of Changes (Document date: Oct 1 2018)

Amendment 2: Version 2.0 (02-Feb-2018) vs. Version 3.0 (01-Oct-2018)

Protocol Section		Description of Changes	Reason / Rationale
Page 27	Section 5.10 –Study flowchart	**Previous wording:** "Application of the SEEQ™ device."<br><br>**Revised wording:** "Application of the wearable adhesive cardiac rhythm monitoring device."	Medtronic's SEEQ program is being phased out.
Page 28	Section 5.12 – Secondary Outcomes	**Previous wording:**<br>**"**2) Cumulative AF/AFL burden during the 14-day monitoring period (with the SEEQ™ device) at 6±1 months after surgery.<br><br>4) Number of days during which the Medtronic SEEQ™ sensor was worn by subjects.<br>5) Reasons for premature removal of the Medtronic SEEQ™ sensor by subjects.<br>6) Incidence of adverse events related to use of the Medtronic SEEQ™ device.<br><br>8) … (please refer to appendix F for endpoint definitions).<br>9) … (please refer to appendix G for definitions of bleeding outcomes). "<br><br>**Revised wording:**<br>"2) Cumulative AF/AFL burden during the 14-day monitoring period (recorded by the wearable adhesive cardiac monitoring device) at 6±1 months after surgery.<br><br>4) Number of days during which the wearable adhesive cardiac rhythm monitor sensor was worn by subjects.<br>5) Reasons for premature removal of the wearable adhesive cardiac rhythm monitor by subjects.<br>6) Incidence of adverse events related to use of the wearable adhesive cardiac rhythm monitor.<br><br>8) … (please refer to appendix H for endpoint definitions).<br>9) … (please refer to appendix I for definitions of bleeding outcomes)."	Wording has been revised to reflect the discontinuation of Medtronic's SEEQ program.<br><br>List of Appendices have been updated.

SEARCH-AF Protocol Summary of Changes (Document date: Oct 1 2018)

Amendment 2: Version 2.0 (02-Feb-2018) vs. Version 3.0 (01-Oct-2018)

Protocol Section		Description of Changes	Reason / Rationale
Page 29	Section 5.14	**Previous wording:** "It is possible that subjects may stop wearing the SEEQ™ sensor given that a new sensor needs to be applied every week for a total of 4 weeks (to allow for 30 days of monitoring). To minimize this, research coordinators will contact (telephone or email) subjects on a weekly basis…"<br><br>**Revised wording:** "It is possible that subjects may stop wearing the wearable adhesive cardiac rhythm monitoring device given that a new sensor needs to be applied every week (for the SEEQ™ device) and every 2 weeks (for the CardioStat device) for a maximum monitoring period of up to 30 days. To minimize this, research coordinators will contact (telephone or email) subjects on a weekly (for SEEQ™) or biweekly (for CardioStat)…"<br><br>**New text:** "…and the SEEQ™ device is not currently approved for use in Canada"	Wording has been revised to reflect cardiac monitoring using the SEEQ device versus the CardioStat device.
Page 30	Section 6.2	**Previous wording:** "Please refer to appendix H for details…"<br><br>**Revised wording:** "Please refer to appendix J for details…"	List of Appendices have been updated.
Page 39	Section 14 – Appendices	Information regarding Icentia's CardioStat device has been added as Appendix E and Appendix F.	List of Appendices have been updated.
Page 55	Appendix E	**New Appendix**:<br>The CardioStat cardiac rhythm monitoring device	
Page 56	Appendix F	**New Appendix:**<br>Rhythm diagnoses reported by the CardioStat cardiac rhythm monitoring system and sample summary report	
Page 59-71	Appendix G-K	Subsequent Appendices have been renamed.	

SEARCH-AF Protocol Summary of Changes (Document date: June 30 2020)

Amendment 3: Version 3.0 (01-Oct-2018) vs. Version 4.0 (30-Jun-2020)

Protocol Section		Description of Changes	Reason / Rationale
Page 15	Secondary Objectives (#3)	Previous wording: "To assess subjects' adherence and incidence of adverse events from use of the Medtronic SEEQ™ system."<br><br>Revised wording: "To assess subjects' adherence and incidence of adverse events from use of the Medtronic SEEQ™ system or the CardioStat® cardiac rhythm monitoring device."	Use of the CardioSTAT® cardiac rhythm monitoring device in this trial was implemented on October 1 2018 as the Medtronic SEEQ™ device was phased out by the company. Wording pertaining to CardioSTAT® was included in protocol version 3.0 (dated 01-Oct-2018). Due to oversight, inclusion of this wording was not done for this specific secondary objective. Accordingly, this has been included in protocol version 4.0.
Page 28	Secondary Outcomes (#1)	Previous wording: "AF/AFL lasting for ≥24 hours during 30 days after randomization and at 6±1 months after surgery."<br><br>Revised wording: "AF/AFL lasting for ≥24 hours during 30 days after randomization and between day 31 and the last follow-up date."	Due to the COVID-19 pandemic, study visits could not be conducted at the specified time windows for some of the study participants. Therefore, wording has been revised to account for the fact that study visits and collection of outcomes during follow-up visits could have been conducted outside the pre-specified time windows for some participants.
Page 28	Secondary Outcomes (#2)	Previous wording: "Cumulative AF/AFL burden during the 14-day monitoring period (recorded by the wearable adhesive cardiac monitoring device) at 6±1 months after surgery."<br><br>Revised wording: "Cumulative AF/AFL burden during the 14-day monitoring period (recorded by the wearable adhesive cardiac monitoring device) between day 31 and the last follow-up date."	Due to the COVID-19 pandemic, study visit #2 (originally planned at 6±1 months after surgery) could not be conducted at the specified time window for some of the study participants. As a result, some subjects did not undergo cardiac rhythm monitoring at 6±1 months after surgery. Therefore, wording has been revised to account for the fact that cardiac rhythm monitoring was conducted outside the 6±1 month window for some participants.
Page 28	Secondary Outcomes (#3)	Previous wording: "Proportion of subjects who are prescribed with oral anticoagulation at the following timepoints: (i) 45 days after discharge from cardiac surgery; (ii) 6±1 months after discharge from cardiac surgery; (iii) 9±1 months after discharge from cardiac surgery. "	Due to the COVID-19 pandemic, study visits could not be conducted at the specified time windows for some of the study participants. Therefore, wording has been revised to account for the fact that study visits and collection of outcomes during follow-up visits could have been conducted outside the pre-specified time

Protocol Section		Description of Changes	Reason / Rationale
		Revised wording: "Proportion of subjects who are prescribed with oral anticoagulation at the following timepoints: (i) 45 days after discharge from cardiac surgery; (ii) between day 46 and the last follow-up date."	windows for some participants.
Page 28	Secondary Outcomes (#4)	Previous wording: "Number of days during which the wearble adhesive cardiac rhythm monitor sensor was worn by subjects.<br><br>Revised wording: **"**Number of days during which the wearable adhesive cardiac rhythm monitor sensor was worn by subjects."	There was a typo ("wearble") which has been corrected.

STATISTICAL ANALYSIS PLAN

Protocol Title: Post-Surgical Enhanced Monitoring for Cardiac Arrhythmias and Atrial Fibrillations (SEARCH-AF): A Randomized Controlled Trial

Short Title: SEARCH-AF

ClinicalTrials.gov Identifier: NCT02793895

Principal Investigators: Dr. Andrew C.T. Ha[a], Dr. Atul Verma[b], C. David Mazer[c], and Dr. Subodh Verma[c]

[a]Toronto General Hospital, University Health Network
[b]Southlake Regional Health Centre
[c]St. Michael's Hospital, Unity Health Toronto

Last Revision Date: July 14 2020
Version: 1.1
Final Sign-off Date: July 14 2020
Archive Date: July 14 2020

The information in this document is confidential. It is understood that information in this document shall not be disclosed to any third party, in any form, without prior written consent of an authorized officer of St. Michael's Hospital.

Study Biostatistician:
Fei Zuo
Research Biostatistician
Applied Health Research Centre (AHRC)
The HUB, Li Ka Shing Knowledge Institute
St. Michael's Hospital, Unity Health Toronto

July 14 2020

Signature **Date**

Peer Review Biostatistician:
Kevin E. Thorpe
Head of Biostatistics
Applied Health Research Centre (AHRC)
The HUB, Li Ka Shing Knowledge Institute
St. Michael's Hospital, Unity Health Toronto

July 14 2020

Signature **Date**

Approved by:
Dr. Andrew C.T. Ha
Cardiac Electrophysiology
Peter Munk Cardiac Center
Toronto General Hospital, University Health Network

July 14 2020

Signature **Date**

Approved by:
Dr. Atul Verma
Cardiac ElectrophysiologySouthlake Regional Health Centre

July 14 2020

Signature **Date**

Approved by:
Dr. C. David Mazer
Anesthesiology and Scientist
Keenan Research Centre for Biomedical Science
St. Michael's Hospital, Unity Health Toronto

July 14 2020

Signature **Date**

Study Biostatistician:

Fei Zuo
Research Biostatistician
Applied Health Research Centre (AHRC)
The HUB, Li Ka Shing Knowledge Institute
St. Michael's Hospital, Unity Health Toronto

	July 14 2020
**Signature**	**Date**

Peer Review Biostatistician:

Kevin E. Thorpe
Head of Biostatistics
Applied Health Research Centre (AHRC)
The HUB, Li Ka Shing Knowledge Institute
St. Michael's Hospital, Unity Health Toronto

Kevin Thorpe	July 14 2020
**Signature**	**Date**

Approved by:

Dr. Andrew C.T. Ha
Cardiac Electrophysiology
Peter Munk Cardiac Center
Toronto General Hospital, University Health Network

	July 14 2020
**Signature**	**Date**

Approved by:

Dr. Atul Verma
Cardiac ElectrophysiologySouthlake Regional Health Centre

	July 14 2020
**Signature**	**Date**

Approved by:

Dr. C. David Mazer
Anesthesiology and Scientist
Keenan Research Centre for Biomedical Science
St. Michael's Hospital, Unity Health Toronto

	July 14 2020
**Signature**	**Date**

**Study Biostatistician:**	Fei Zuo<br>Research Biostatistician<br>Applied Health Research Centre (AHRC)<br>The HUB, Li Ka Shing Knowledge Institute<br>St. Michael's Hospital, Unity Health Toronto	
	**Signature**	July 14 2020<br>**Date**
**Peer Review Biostatistician:**	Kevin E. Thorpe<br>Head of Biostatistics<br>Applied Health Research Centre (AHRC)<br>The HUB, Li Ka Shing Knowledge Institute<br>St. Michael's Hospital, Unity Health Toronto	
	**Signature**	July 14 2020<br>**Date**
**Approved by:**	Dr. Andrew C.T. Ha<br>Cardiac Electrophysiology<br>Peter Munk Cardiac Center<br>Toronto General Hospital, University Health Network	
	**Signature**	July 14 2020<br>**Date**
**Approved by:**	Dr. Atul Verma<br>Cardiac ElectrophysiologySouthlake Regional Health Centre	
	**Signature**	July 14 2020<br>**Date**
**Approved by:**	Dr. C. David Mazer<br>Anesthesiology and Scientist<br>Keenan Research Centre for Biomedical Science<br>St. Michael's Hospital, Unity Health Toronto	
	**Signature**	July 14 2020<br>**Date**

**Study Biostatistician:**	Fei Zuo<br>Research Biostatistician<br>Applied Health Research Centre (AHRC)<br>The HUB, Li Ka Shing Knowledge Institute<br>St. Michael's Hospital, Unity Health Toronto

July 14 2020

Signature **Date**

**Peer Review Biostatistician:**	Kevin E. Thorpe<br>Head of Biostatistics<br>Applied Health Research Centre (AHRC)<br>The HUB, Li Ka Shing Knowledge Institute<br>St. Michael's Hospital, Unity Health Toronto

July 14 2020

Signature **Date**

**Approved by:**	Dr. Andrew C.T. Ha<br>Cardiac Electrophysiology<br>Peter Munk Cardiac Center<br>Toronto General Hospital, University Health Network

July 14 2020

Signature **Date**

**Approved by:**	Dr. Atul Verma<br>Cardiac ElectrophysiologySouthlake Regional Health Centre

July 14 2020

Signature **Date**

**Approved by:**	Dr. C. David Mazer<br>Anesthesiology and Scientist<br>Keenan Research Centre for Biomedical Science<br>St. Michael's Hospital, Unity Health Toronto

July 14 2020

Signature **Date**

Study Biostatistician: Fei Zuo
Research Biostatistician
Applied Health Research Centre (AHRC)
The HUB, Li Ka Shing Knowledge Institute
St. Michael's Hospital, Unity Health Toronto

	July 14 2020
**Signature**	**Date**

Peer Review Biostatistician: Kevin E. Thorpe
Head of Biostatistics
Applied Health Research Centre (AHRC)
The HUB, Li Ka Shing Knowledge Institute
St. Michael's Hospital, Unity Health Toronto

	July 14 2020
**Signature**	**Date**

Approved by: Dr. Andrew C.T. Ha
Cardiac Electrophysiology
Peter Munk Cardiac Center
Toronto General Hospital, University Health Network

	July 14 2020
**Signature**	**Date**

Approved by: Dr. Atul Verma
Cardiac ElectrophysiologySouthlake Regional Health Centre

	July 14 2020
**Signature**	**Date**

Approved by: Dr. C. David Mazer
Anesthesiology and Scientist
Keenan Research Centre for Biomedical Science
St. Michael's Hospital, Unity Health Toronto

	July 14 2020
**Signature**	**Date**

Approved by:

Dr. Subodh Verma
Cardiac Surgery and Scientist
Keenan Research Centre for Biomedical Science
St. Michael's Hospital, Unity Health Toronto

**Signature**	July 14 2020<br>**Date**

Approved by:

Dr. Peter Jüni
Director
Applied Health Research Centre (AHRC)
The HUB, Li Ka Shing Knowledge Institute
St. Michael's Hospital, Unity Health Toronto

**Signature**	July 14 2020<br>**Date**

Approved by: Dr. Subodh Verma
Cardiac Surgery and Scientist
Keenan Research Centre for Biomedical Science
St. Michael's Hospital, Unity Health Toronto

July 14 2020

Signature **Date**

Approved by: Dr. Peter Jüni
Director
Applied Health Research Centre (AHRC)
The HUB, Li Ka Shing Knowledge Institute
St. Michael's Hospital, Unity Health Toronto

July 14 2020

Signature **Date**

1. INTRODUCTION

This statistical analysis plan (SAP) outlines the planned statistical methods for the summary and analysis of data collected within the scope of the Post-Surgical Enhanced Monitoring for Cardiac Arrhythmias and Atrial Fibrillation (SEARCH-AF) trial protocol version 4.0 dated June 30 2020.

The SAP should be read in conjunction with the study protocol and the electronic Case Report Forms (eCRFs). This version of the SAP has been developed using the final version of the protocol mentioned above and eCRFs version 8.5.11 dated September 5, 2019.

All statistical analyses will be performed using R version 3.6.2, or higher.

2. STUDY OBJECTIVES

2.1 Primary Objective

To test whether enhanced cardiac rhythm monitoring with an adhesive, continuous monitoring device results in higher rates of atrial fibrillation/atrial flutter (AF/AFL) detection during the subacute, post-discharge period of cardiac surgical subjects who are at risk of developing post-operative atrial arrhythmias, when compared to usual care.

Primary Outcome and Endpoint

The primary outcome of this trial is the difference in proportions of participants with a cumulative atrial fibrillation/flutter (AF/AFL) duration of ≥ 6 minutes or documentation of AF/AFL by a 12-lead electrocardiogram (ECG) between groups. The primary endpoint of this trial is documentation of sustained AF/AFL, defined as a cumulative AF/AFL duration of ≥ 6 minutes or documentation of AF/AFL by a 12-lead ECG within 30 days after randomization. Clinical endpoints will be adjudicated by an independent clinical events committee.

2.2 Secondary Objectives

To compare the following secondary outcomes between the enhanced cardiac rhythm monitoring group and the usual care group:

2.2.1 Secondary Outcomes

1) Proportion of subjects with detection of cumulative AF/AFL lasting for ≥24 hours at the following time points:
   i. within the first 30 days after randomization
   ii. between day 31 and the last follow-up date

2) Duration of cumulative AF/AFL burden detected during the 14-day monitoring period (recorded by the wearable adhesive cardiac monitoring device) among subjects:

i. between day 31 and the last follow-up date

3) Proportion of subjects who are prescribed oral anticoagulation at the following time points:
   i. within the first 45 days after discharge from surgery
   ii. between day 46 and the last follow-up date

4) Number of days during which the protocol-mandated wearable cardiac rhythm monitor sensor was worn by subjects.
   i. within the first 30 days after randomization (note: applicable for enhanced cardiac monitoring group only)
   ii. between day 31 and the last follow-up date

5) Reasons for premature removal of protocol-mandated wearable adhesive cardiac rhythm monitors by subjects.

6) Proportion of subjects who experienced adverse events related to the use of protocol-mandated wearable adhesive cardiac rhythm monitors at the following time points:
   i. within the first 30 days after randomization
   ii. between day 31 and the last follow-up date

7) Performance of non-protocol mandated Holter monitoring and/or event recorders during the 30-day period after randomization.
   i. within the first 30 days after randomization

8) Proportion of subjects who experienced major adverse cardiac event (MACE) outcomes (ALL-cause death, myocardial infarction, ischemic stroke, non-CNS system embolism) at the following time points:
   i. within the first 45 days after discharge from surgery
   ii. between day 46 and the last follow-up date

9) Proportion of subjects with major bleeding events at the following time points:
   i. within the first 45 days after discharge from surgery
   ii. between day 46 and the last follow-up date

10) Hospitalization or emergency room visits.
   i. within the first 45 days after hospital discharge from index cardiac surgery
   ii. between day 46 and the last follow-up date

(note: This secondary outcome was listed in the original clinicaltrials.gov submission dated on June 2 2016 but was not explicitly listed in the protocol).

11) Proportion of subjects with cumulative AF/AFL lasting for ≥6 hours at the following time points:
    i. within the first 30 days after randomization
    ii. between day 31 and the last follow-up date

(note: this secondary outcome was pre-specified before data lock and data analysis but was not explicitly listed in the protocol).

To describe the following outcomes in the enhanced cardiac rhythm monitoring group and/or the usual care group:

2.2.2 Descriptive Outcomes

1) Proportion of subjects who are prescribed oral anticoagulation at the following time points:
    i. within the first 30 days after randomization

2) Proportion of subjects with cumulative AF/AFL lasting for ≥6 minutes at the following time points:
    i. within the first 30 days after randomization
    ii. between 31 days and the last follow-up date

3) Proportion of subjects with detection of at least 1 episode of AF/AFL lasting for ≥30 seconds:
    i. within the first 30 days after randomization

4) Proportion of subjects with detection of at least 1 episode of AF/AFL lasting for ≥6 minutes on a continuous basis at the following time points:
    i. within the first 30 days after randomization
    ii. between 31 days and the last follow-up date

5) Proportion of subjects with detection of at least 1 episode of AF/AFL lasting for ≥6 hours on a continuous basis at the following time points:
    i. within the first 30 days after randomization
    ii. between 31 days and the last follow-up date

6) Proportion of subjects with detection of at least 1 episode of AF/AFL lasting for ≥24 hours on a continuous basis at the following time points:
    i. within the first 30 days after randomization
    ii. between 31 days and the last follow-up date

7) For each subject, the total number of AF/AFL episodes lasting for ≥6 minutes on a continuous basis at the following time points:
    i. within the first 30 days after randomization
    ii. between 31 days and the last follow-up date

8) For each subject, the total number of AF/AFL episodes lasting for ≥6 hours on a continuous basis at the following time points:
    i. within the first 30 days after randomization
    ii. between 31 days and the last follow-up date

9) For each subject, the total number of AF/AFL episodes lasting for ≥24 hours on a continuous basis at the following time points:
    i. within the first 30 days after randomization
    ii. between 31 days and the last follow-up date

10) Proportion of subjects with at least one 12-lead ECG demonstrating AF/AFL at the following time points:
    i. within the first 30 days after randomization
    ii. between 31 days and the last follow-up date

11) Proportion of subjects who underwent non-protocol mandated continuous cardiac rhythm Holter monitoring and/or event recorders at the following time points:
    i. between day 31 and the last follow-up date

12) Histogram of compliance percentages (protocol-mandated wearable adhesive cardiac rhythm monitoring sensor use)
    i. Within the first 30 days after randomization
    ii. between day 31 and the last follow-up date

13) Daily burden of AF/AFL burden against time (Figure): This will be calculated on a per-subject basis.
    i. within the first 30 days
    ii. between day 31 and the last follow-up date

14) Cumulative burden of AF/AFL: this metric quantifies the percentage (%) of AF/AFL detected during time periods at which protocol-mandated continuous cardiac rhythm monitoring is performed (AF/AFL burden (%) = Cumulative duration of AF/AFL detected ÷ Total amount of time during which protocol-mandated cardiac rhythm monitoring was performed). This will be calculated on a per-subject basis.
    i. within the first 30 days after randomization
    ii. between day 31 and last follow-up date

15) Number of subjects who prematurely removed protocol-mandated adhesive cardiac rhythm monitoring sensors at the following time periods:
    i. within the first 30 days after randomization
    ii. between day 31 and the last follow-up date

3. STUDY DESIGN

3.1 Study Design

This is an open-label, two-arm RCT comparing a strategy of enhanced cardiac rhythm monitoring to usual care (control group) for detecting post-operative AF/AFL among post-cardiac surgical subjects at risk for developing post-operative atrial arrhythmias.

3.2 Study Cohort

The study cohort is consisted of randomized post-cardiac surgical subjects at high risk of stroke, defined as having a CHA_2DS_2-VASC score of ≥4 or a CHA_2DS_2-VASC score of ≥2 with additional risk factors for developing POAF/AFL. These subjects must not have a history of AF/AFL pre-operatively.

3.2.1 Intent-to-Treat (ITT) Cohort

The Intent-to-Treat (ITT) cohort will consist of all subjects who were randomized to the enhanced cardiac rhythm monitoring group or the control group. The primary analysis for outcomes is based on the ITT cohort. Analyses will be conducted on the ITT cohort according to the treatment group to which the subjects were randomized.

3.2.2 Per-Protocol (PP) Cohort

The Per-Protocol (PP) cohort will consist of all randomized subjects who received their allocated intervention without significant deviations in the assigned treatment during the first 30 days after randomization:

- For subjects randomized to the enhanced cardiac rhythm monitoring group, the per-protocol (PP) cohort is defined by subjects who wore the sensor for ≥24 hours within the first 30 days after randomization.
- For subjects randomized to the usual care group, the PP cohort is defined by subjects who did not wear a continuous cardiac rhythm monitor (SEEQ™ or CardioSTAT® monitor) during the first 30 days after randomization.

3.3 Treatment Definition

There are two treatment groups in the study. Patients are randomized (1:1) to receive (i) usual care (control group) or (ii) enhanced cardiac rhythm monitoring (intervention group).

4. STATISTICAL ANALYSIS

Categorical data will be summarized by counts and percentages, and continuous variables by means, medians and standard deviations. All outcome comparisons will include a treatment effect estimate along with a 95% confidence interval. All outcome comparisons will include a p-value calculated using an appropriate test. The adjusted estimates of the treatment effect and their 95% confidence intervals will also be reported for all relevant outcomes. Candidate covariates will include age, sex, heart failure/left ventricular (LV) dysfunction, diabetes, hypertension, history of ischemic stroke, renal disease, chronic obstructive pulmonary disease (COPD), isolated CABG vs. valve replacement/repair ± CABG, CHA_2DS_2-VASC score, and left atrial diameter.

4.1 Primary Analysis

The primary analysis will compare the proportions of subjects with cumulative AF/AFL lasting ≥6 minutes detected by continuous cardiac rhythm monitoring or having at least one 12-lead ECG demonstrating AF/AFL within 30 days after randomization between the 2 treatment arms (enhanced cardiac rhythm monitoring group or usual care). Difference in proportions and the 95% confidence interval will be reported. A Chi-squared test of independence will be conducted to obtain the p-value.

4.2 Secondary Analysis

For the secondary outcomes outlined in section 2.2.1, a chi-squared test of independence will be used to compare the proportions between groups where appropriate. Difference in proportions, the 95% confidence intervals, and p values will be reported.

For the descriptive outcomes outlined in section 2.2.2, only descriptive summaries will be presented. Mean and standard deviations or median with interquartile range (where appropriate) will be reported for continuous outcomes. Counts and percentages will be reported for categorical outcomes. No inferential analyses of these outcomes are planned unless they are specifically requested during the peer-review process and deemed appropriate by the statistical team.

4.3 Adjusted Analysis

A logistic regression will be conducted on the primary outcome and secondary outcomes with binary responses, adjusting for candidate covariates. Performance of each of these analyses will be contingent on the number of outcomes accrued. A given regression analysis may not be performed if the number of outcomes is small.

4.4 Subgroup Analysis

Sub-analyses for the following will be conducted. The subgroups are as follows:

- Isolated CABG vs. Valve replacement/repair ± CABG
- Sex (Male vs. Female)
- Age
- CHA_2DS_2-VASC score
- Left atrial diameter

4.5 Sensitivity analysis

Sensitivity analysis will be performed for the per-protocol cohort.

4.6 Missing data

In the presence of missing data in the primary and secondary outcomes, sensitivity analyses will be carried out. Based on the type or amount of missing data, this may include multiple imputation and inverse probability-weighted models.
