# Supplementary material for: Effect of Continuous Electrocardiogram Monitoring on Detection of Undiagnosed Atrial Fibrillation After Hospitalization for Cardiac Surgery: A Randomized Clinical Trial
Source: JAMA Netw Open. 2021 Aug 27;4(8):e2121867. doi: 10.1001/jamanetworkopen.2021.21867 (PMC8397929; doi:10.1001/jamanetworkopen.2021.21867)
Supplement: Supplement 2. — eTable 1. SEARCH-AF Inclusion/Exclusion Criteria Inclusion Criteria eTable 2. SEARCH-AF Secondary Endpoints eTable 3. Reasons for Screen Failure in the SEARCH-AF Trial eTable 4. Reasons for Premature Termination of Monitoring eTable 5. Description of Detected AF Duration by Continuous Cardiac Rhythm Monitoring Within 30 Days of Randomization, Stratified by Week of Monitoring eTable 6. Rhythm-Based Outcomes Within 30 Days of Randomization eTable 7. Detection of Atrial Fibrillation/Flutter After 30 Days of Randomization eTable 8. Results of all Secondary Endpoints (Intent-to-Treat Cohort) eTable 9. Per Protocol Analysis eTable 10. Factors Associated with Occurrence of the Primary Endpoint eTable 11. Detection of Atrial Fibrillation Within 30 Days of Randomization in Relation to Presence of Post-Operative Atrial Fibrillation Occurring Prior to Randomization eTable 12. Detection of Atrial Fibrillation Within the First 30 Days After Randomization, According to Type of Wearable Sensor Employed eFigure. Primary Outcome According to Pre-Specified Subgroups [file jamanetwopen-e2121867-s002.pdf]

Supplemental Online Content

Ha ACT, Verma S, Mazer CD, et al; SEARCH AF CardioLink-1 Investigators. Effect of continuous electrocardiogram monitoring on detection of undiagnosed atrial fibrillation after hospitalization for cardiac surgery: a randomized clinical trial. *JAMA Netw Open*. 2021;4(8):e2121867. doi:10.1001/jamanetworkopen.2021.21867

eTable 1. SEARCH-AF Inclusion/Exclusion Criteria Inclusion Criteria

eTable 2. SEARCH-AF Secondary Endpoints

eTable 3. Reasons for Screen Failure in the SEARCH-AF Trial

eTable 4. Reasons for Premature Termination of Monitoring

eTable 5. Description of Detected AF Duration by Continuous Cardiac Rhythm Monitoring Within 30 Days of Randomization, Stratified by Week of Monitoring

eTable 6. Rhythm-Based Outcomes Within 30 Days of Randomization

eTable 7. Detection of Atrial Fibrillation/Flutter After 30 Days of Randomization

eTable 8. Results of all Secondary Endpoints (Intent-to-Treat Cohort)

eTable 9. Per Protocol Analysis

eTable 10. Factors Associated with Occurrence of the Primary Endpoint

eTable 11. Detection of Atrial Fibrillation Within 30 Days of Randomization in Relation to Presence of Post-Operative Atrial Fibrillation Occurring Prior to Randomization

eTable 12. Detection of Atrial Fibrillation Within the First 30 Days After Randomization, According to Type of Wearable Sensor Employed

eFigure. Primary Outcome According to Pre-Specified Subgroups

This supplemental material has been provided by the authors to give readers additional information about their work.

eTable 1. SEARCH-AF Inclusion/Exclusion Criteria Inclusion Criteria

1. Male or female age ≥18 years.
2. Isolated CABG or valve replacement/repair +/- CABG performed at the index surgical procedure.
3. At elevated risk of stroke and for having POAF/AFL, defined as having a CHA_2DS_2-VASC score of ≥4 or ≥2 with at least 1 of the following risk factors for developing POAF/AFL:
    a. Chronic obstructive pulmonary disease.
    b. Sleep apnea.
    c. Impaired renal function (defined as creatinine clearance <60 ml/min or <60 ml/min/1.73 m^2).
    d. Echocardiographic evidence of at least mild left atrial enlargement (defined as ≥41 mm on M-mode, ≥59 ml or ≥29 mL/m^2 on biplane volume assessment from an echocardiogram performed within 12 months of study enrollment)
    e. Elevated body mass index (defined as BMI ≥30).
    f. Combined surgery (CABG + valve repair/replacement).
4. Able to provide written informed consent.

Exclusion Criteria

1. Atrial fibrillation or flutter at the time of randomization.
2. Known previous history of AF/AFL, diagnosed pre-operatively (note: documentation of a history of AF/AFL without accompanying rhythm proof will suffice).
3. Documentation of continuous AF/AFL for ≥24 hours during the in-hospital stay for the index cardiac surgery.
4. Subjects who, at the discretion of the treating cardiac surgery team, would be treated and discharged with oral anticoagulation due to POAF/AFL.
5. Mechanical valve replacement.
6. Current or anticipated treatment with oral anticoagulation for indications other than AF/AFL.
7. Hospitalization for ≥10 days (for the index cardiac surgery, with day #0 being the day of surgery).
8. Planned discharge from hospital with a type IC or III anti-arrhythmic drug.
9. Having received >5 grams of IV and/or oral amiodarone during hospitalization for the index cardiac surgical procedure.
10. Women of childbearing potential.
11. History of percutaneous or surgical left atrial ablation for AF.
12. Presence of a cardiac implantable electronic device with a functioning atrial lead (pacemaker, implantable cardioverter defibrillator, or cardiac resynchronization device).
13. Presence of an implantable loop recorder.
14. History of left atrial appendage ligation, removal, or occlusion.
15. Subjects with known allergies or hypersensitivities to adhesives or hydrogel.
16. Inability to provide written informed consent.
17. Current or anticipated participation in another randomized controlled trial in which the interventional drug or device is known to affect the incidence of the primary or secondary outcomes of this study.

Abbreviations: AF/AFL, atrial fibrillation/flutter; BMI, body mass index; CABG, coronary artery bypass grafting; CHA_2DS_2-VASc, congestive heart failure, hypertension, age ≥75, diabetes, prior stroke or transient ischemic attack, vascular disease, age ≥65, female sex (1 point each except age >75 and stroke which are 2 points); IV, intravenous; POAF/AFL, post-operative atrial fibrillation/flutter.

© 2021 Ha ACT et al. *JAMA Network Open.*

eTable 2. SEARCH-AF Secondary Endpoints

Secondary endpoint		Included in this report?
**1**	Proportion of subjects with detection of cumulative AF/AFL lasting for ≥24 hours at the following time points:	
	i. within the first 30 days after randomization	**Main manuscript**
	ii. between day 31 and the last follow-up date	**eTable8**
**2**	Duration of cumulative AF/AFL burden detected during the 14-day monitoring period (recorded by the wearable adhesive cardiac monitoring device) among subjects between day 31 and the last follow-up date	**eTable8**
**3**	Proportion of subjects who are prescribed oral anticoagulation at the following time points:	
	i. within the first 45 days after discharge from surgery	**Main manuscript**
	ii. between day 46 and the last follow-up date	**Main manuscript**
**4**	Number of days during which the protocol-mandated wearable cardiac rhythm monitor sensor was worn by subjects.	
	i. within the first 30 days after randomization (note: applicable for enhanced cardiac monitoring group only)	**eTable8**
	i. between day 31 and the last follow-up date	**eTable8**
**5**	Reasons for premature removal of protocol-mandated wearable adhesive cardiac rhythm monitors by subjects.	**eTable4**
**6**	Proportion of subjects who experienced adverse events related to the use of protocol-mandated wearable adhesive cardiac rhythm monitors at the following time points:	
	i. within the first 30 days after randomization	**Main manuscript**
	ii. between day 31 and the last follow-up date	**eTable8**
**7**	Performance of non-protocol mandated Holter monitoring and/or event recorders during the 30-day period after randomization.	
	i. within the first 30 days after randomization	**eTable8**
**8**	Proportion of subjects who experienced major adverse cardiac event (MACE) outcomes (All-cause death, myocardial infarction, ischemic stroke, non-CNS system embolism) at the following time points:	
	i. within the first 45 days after discharge from surgery	**Main manuscript**
	ii. between day 46 and the last follow-up date	**Main manuscript**
**9**	Proportion of subjects with major bleeding events at the following time points:	
	i. within the first 45 days after discharge from surgery	**Main manuscript**
	ii. between day 46 and the last follow-up date	**Main manuscript**
**10**	Hospitalization or emergency room visits	
	i. within the first 45 days after discharge from surgery	**eTable8**
	ii. between day 46 and the last follow-up date	**eTable8**
**11**	Proportion of subjects with cumulative AF/AFL lasting for ≥6 hours at the following time points:	
	i. within the first 30 days after randomization	**Main manuscript**
	ii. between day 31 and the last follow-up date	**eTable8**

Abbreviations: AF/AFL, atrial fibrillation/flutter; non-CNS, non-central nervous system

© 2021 Ha ACT et al. *JAMA Network Open.*

eTable 3. Reasons for Screen Failure in the SEARCH-AF Trial

In the SEARCH-AF trial, 2803 patients were screened for eligibility and 2467 patients were not enrolled into the study. Below is a detailed list of reasons of why these patients were screened but not enrolled into the SEARCH-AF trial.

Specific reason for not enrolled into the study	***Number of patients***
**Declined participation**	**840**
**Did not meet study eligibility criteria**	**1204**
Not eligible due to low CHA_2DS_2-VASc score	211
Atrial fibrillation	207
Unable to provide consent	189
Prolonged hospitalization (more than 10 days)	155
Participating in another study	139
Anticoagulated	110
Treated with amiodarone	91
Pacemaker / ICD / ILR	38
Allergy to adhesive	27
Mechanical valve	14
Women of childbearing age	8
No CABG or valve surgery performed	6
Not specified	5
Left atrial appendage ligation	2
Unable to apply wearable sensor	2
**Other reasons**	**423**
Patient was not approached due to unavailability of research personnel	168
Not specified	104
Unable to attend follow-up	87
Investigator decision to not enrol patient into the study	31
Wearable sensor not available at time of screening	19
Discharged to another facility (therefore the patient was unable to complete study visits)	9
Death	3
Unable to approach the patient for study participation	2

Abbreviations: CABG, coronary artery bypass grafting; CHA_2DS_2-VASc, congestive heart failure, hypertension, age ≥75, diabetes, prior stroke or transient ischemic attack, vascular disease, age ≥65, female sex (1 point each except age >75 and stroke which are 2 points); ICD, implantable cardioverter defibrillator; ILR, implantable loop recorder.

© 2021 Ha ACT et al. *JAMA Network Open.*

eTable 4. Reasons for Premature Termination of Monitoring

Number of patients in the intervention group (n=163)

	**Number of patients**
Skin irritation	13
Patient preference	18
Wearable sensor was dislodged during shower	1
Wearable sensor malfunction	1
Patient forgot to reapply the wearable sensor	1
Patient lost the wearable sensors	1
Patient was readmitted to hospital and the wearable sensor was not worn during re-hospitalization	1
Patient required reoperation and wearable sensor device was removed	1
Not specified	13
**Total**	50

Note: In total, 17 patients in the intervention group reported skin irritation related to the adhesive material of the wearable patch-based device within 30 days of randomization. Of these 17 patients, 13 of them had premature termination of continuous cardiac rhythm monitoring.

© 2021 Ha ACT et al. *JAMA Network Open.*

eTable 5. Description of Detected AF Duration by Continuous Cardiac Rhythm Monitoring Within 30 Days of Randomization, Stratified by Week of Monitoring.

Week of monitoring	Summary of key findings
First	▪ Cumulative AF/AFL lasting for ≥6 minutes was detected in 22 patients. The median duration of cumulative detected AF/AFL was 97.6 minutes (25th percentile: 2.6 minutes, 75th percentile: 559.1 minutes).<br>▪ Cumulative AF/AFL lasting for ≥24 hours (≥1440 minutes) was detected in 3 patients in the first week.
Second	▪ Cumulative AF/AFL lasting for ≥6 minutes was detected in 13 patients. The median duration of cumulative detected AF/AFL was 0.20 minutes (25th percentile: 0 minutes, 75th percentile: 120.5 minutes).<br>▪ Cumulative AF/AFL lasting for ≥24 hours was detected in 3 patients while 15 patients had no detected AF/AFL.
Third	▪ The median duration of detected AF/AFL was 0 minutes.<br>▪ Cumulative AF/AFL lasting for ≥6 minutes was detected in 5 patients.
Fourth	▪ The median duration of detected AF/AFL was 0 minutes.<br>▪ Cumulative AF/AFL lasting for ≥6 minutes (108 minutes) was detected in 1 patient.

Abbreviations: AF/AFL, atrial fibrillation/flutter.

eTable 6. Rhythm-Based Outcomes Within 30 Days of Randomization

	Intervention (n=163)	Usual care (n=173)	Rate difference (%) and 95% CI
Patients with at least 1 episode of AF/AFL lasting ≥30 seconds within 30 days after randomization	34 (20.9)	0 (0.0)	20.9 (14.6 to 27.1)
Patients with a cumulative duration of AF/AFL lasting ≥6 minutes within 30 days after randomization	30 (18.4)	0 (0.0)	18.4 (12.5 to 24.4)
Patients with at least 1 episode of AF/AFL lasting ≥6 minutes within 30 days after randomization	30 (18.4)	0 (0.0)	18.4 (12.5 to 24.4)
Patients with at least 1 episode of AF/AFL lasting ≥6 hours within 30 days after randomization	12 (7.4)	0 (0.0)	7.4 (3.4 to 11.4)
Patients with at least 1 episode of AF/AFL lasting ≥24 hours within 30 days after randomization	1 (0.6)	0 (0.0)	0.6 (-0.6 to 1.8)

Abbreviations: AF/AFL, atrial fibrillation/flutter; CI, confidence interval.

Note: These rhythm-based outcomes were listed as “descriptive outcomes” in the Statistical Analysis Plan. Comparative analysis between the two randomization groups was performed on an exploratory basis.

© 2021 Ha ACT et al. *JAMA Network Open.*

eTable 7: Detection of Atrial Fibrillation/Flutter After 30 Days of Randomization

**Timing of AF occurrence in relation to randomization date**	
**Patient #1**	37 days (with 12-lead ECG)
**Patient #2**	51 days (with 12-lead ECG)
**Patient #3**	75 days (with 12-lead ECG)<br>202 days (with continuous cardiac rhythm monitoring)
**Patient #4**	93 days (with 12-lead ECG)
**Patient #5**	170 days (with continuous cardiac rhythm monitoring)
**Patient #6**	173 days (with continuous cardiac rhythm monitoring)
**Patient #7**	206 days (with 12-lead ECG)

At 6 months, 189/336 (56%) of patients underwent protocol-mandated continuous cardiac rhythm monitoring with a 2-week patch-based monitor. Among the 189 patients who underwent continuous cardiac rhythm monitoring at 6-month follow-up, cumulative AF lasting for ≥6 minutes was detected in 3 (1.6%) patients.

In the overall cohort, cumulative AF/AFL lasting for ≥6 minutes or AF/AFL detected by a 12-lead electrocardiogram (ECG) occurred in a total of 7 (2.1%) patients between 31 days after randomization and the end of follow-up. Atrial fibrillation was detected in 4 patients by a 12-lead ECG, cumulative AF/AFL lasting for ≥6 minutes was detected in 2 patients, and 1 patient had AF/AFL detected by both modalities. The median follow-up period of the study was cohort was 9 months.

Abbreviations: AF/AFL, atrial fibrillation/flutter; ECG, electrocardiogram.

© 2021 Ha ACT et al. *JAMA Network Open.*

eTable 8. Results of all Secondary Endpoints (Intent-to-Treat Cohort)

Secondary outcome		Intervention (n-163)	Usual care (n=173)	Estimate and 95% CI
1	Proportion of subjects with detection of cumulative AF/AFL lasting for ≥24 hours at the following time points:			
	i. within the first 30 days after randomization – no. (%)	5 (3.1)	0 (0.0)	3.1 (0.4 to 5.7)+
	ii. between day 31 and the last follow-up date – no. (%)	0 (0)	1 (0.6)	-0.6 (-1.7 to 0.6)
2	Duration of cumulative AF/AFL burden detected during the 14-day monitoring period (recorded by the wearable adhesive cardiac monitoring device) among subjects between day 31 and the last follow-up date - % (SD)	0 (0)	0.1 (1.1)	-0.1 (-0.3 to 0.1)
3	Proportion of subjects who are prescribed oral anticoagulation at the following time points:			
	i. within the first 45 days after discharge from surgery – no. (%)	7 (4.3)	4 (2.3)	2.0 (-1.9 to 5.8)
	ii. between day 46 and the last follow-up date – no. (%)	6 (3.7)	5 (2.9)	0.8 (-3.0 to 4.6)
4	Number of days during which the protocol-mandated wearable cardiac rhythm monitor sensor was worn by subjects.			
	i. within the first 30 days after randomization (note: applicable for enhanced cardiac monitoring group only) – mean number of days (SD)	21.2 (9.9)	0.0 (0.0)	21.2 (19.7 to 22.6)*
	ii. between day 31 and the last follow-up date – mean number of days (SD)	7.4 (7.3)	8.1 (6.8)	-0.7 (-2.2 to 0.8)
5	Reasons for premature removal of protocol-mandated wearable adhesive cardiac rhythm monitors by subjects.	See eTable4	See eTable4	not applicable
6	Proportion of subjects who experienced adverse events related to the use of protocol-mandated wearable adhesive cardiac rhythm monitors at the following time points:			
	i. within the first 30 days after randomization – no. (%)	17 (10.4)	0 (0)	10.4 (5.7 to 15.1)*
	ii. between day 31 and the last follow-up date – no. (%)	2 (1.2)	4 (2.3)	-1.1 (-3.9 to 1.7)
7	Performance of non-protocol mandated Holter monitoring and/or event recorders during the 30-day period after randomization.			

© 2021 Ha ACT et al. *JAMA Network Open.*

	i. within the first 30 days after randomization – no. (%)	5 (3.1)	4 (2.3)	0.8<br>(-2.7 to 4.2)
**8**	Proportion of subjects who experienced major adverse cardiac event (MACE) outcomes (ALL-cause death, myocardial infarction, ischemic stroke, non-CNS system embolism) at the following time points:			
	i. within the first 45 days after discharge from surgery – no. (%)	1 (0.6)	1 (0.6)	0.0<br>(-1.6 to 1.7)
	ii. between day 46 and the last follow-up date – no. (%)	1 (0.6)	3 (1.7)	-1.1<br>(-3.4 to 1.2)
**9**	Proportion of subjects with major bleeding events at the following time points:			
	i. within the first 45 days after discharge from surgery – no. (%)	0 (0)	1 (0.6)	-0.6<br>(-1.7 to 0.6)
	ii. between day 46 and the last follow-up date – no. (%)	1 (0.6)	3 (1.7)	-1.1<br>(-3.4 to 1.2)
**10**	Hospitalization or emergency room visits			
	i. within the first 45 days after discharge from surgery – no. (%)	13 (8.0)	7 (4.0)	3.9<br>(-1.2 to 9.0)
	ii. between day 46 and the last follow-up date – no. (%)	10 (6.1)	16 (9.2)	-3.1<br>(-8.8 to 2.6)
**11**	Proportion of subjects with cumulative AF/AFL lasting for ≥6 hours at the following time points:			
	i. within the first 30 days after randomization – no. (%)	14 (8.6)	0 (0)	8.6<br>(4.3 to 12.9)*
	ii. between day 31 and the last follow-up date – no. (%)	0 (0)	1 (0.6)	-0.6<br>(-1.7 to 0.6)

+ P=.06; * P <.001

Cumulative burden of AF/AFL: This metric quantified the percentage (%) of AF/AFL detected during time periods at which protocol-mandated continuous cardiac rhythm monitoring was performed (AF/AFL burden (%) = Cumulative duration of AF/AFL detected ÷ Total amount of time during which protocol-mandated cardiac rhythm monitoring was performed). This was calculated on a per-patient basis.

Abbreviations: AF/AFL, atrial fibrillation/flutter, CI, confidence interval, no., number; non-CNS, non-central nervous system; SD, standard deviation.

© 2021 Ha ACT et al. *JAMA Network Open.*

eTable 9. Per Protocol Analysis

The Per-Protocol cohort consisted of all randomized patients who received their allocated intervention without significant deviations in the assigned treatment during the first 30 days after randomization. As pre-specified in the statistical analysis plan, this was defined as the following:

- For patients randomized to the enhanced cardiac rhythm monitoring group, the per-protocol cohort was defined by patients who wore the sensor for ≥24 hours within the first 30 days after randomization.
- For patients randomized to the usual care group, the PP cohort was defined by subjects who did not wear a continuous cardiac rhythm monitor (SEEQ® or CardioSTAT™ monitor) during the first 30 days after randomization.

	**Continuous Monitoring (n=153)**	**Usual care (n=173)**	**Rate difference and 95% CI**
Primary outcome			
Patients with a cumulative duration of AF/AFL lasting ≥6 minutes or 12-lead ECG demonstrating AF/AFL detected in first 30 days – no. (%)	32 (20.9)	3 (1/7)	19.2 (12.4 to 25.9)*
Secondary outcomes (rhythm-based)			
Patients with cumulative duration of AF/AFL lasting ≥6 hours in first 30 days – no. (%)	14 (9.2)	0 (0)	9.2 (4.6 to 13.7)*
Patients with cumulative duration of AF/AFL lasting ≥24 hours in first 30 days – no. (%)	5 (3.3)	0 (0)	3.3 (0.5 to 6.1)+

* P <0.001; + P=0.052

Abbreviations: AF/AFL, atrial fibrillation/flutter; ECG, electrocardiogram; no., number.

© 2021 Ha ACT et al. *JAMA Network Open.*

eTable 10: Factors Associated with Occurrence of the Primary Endpoint

Variable	Odds ratio (95% confidence interval)
CHA_2DS_2-VASc score (per point increase)	1.46 (1.02-2.13)
Valve repair or replacement +/- CABG vs. isolated CABG	2.20 (0.93-5.10)
Occurrence of post-operative AF/AFL (lasting for <24 hours) prior to randomization (yes vs. no)	0.60 (0.03-4.23)

The primary endpoint was defined as detection of cumulative AF/AFL lasting for ≥6 minutes by continuous cardiac rhythm monitoring or AF/AFL detected on a 12-lead ECG. This analysis was conducted among the 163 patients randomized to the intervention group. A logistic regression analysis was conducted to examine between the variables of interest and the primary outcome. Three variables were tested: (i) CHA_2DS_2-VASc score (per point increase), (ii) valve repair or replacement +/- CABG vs. isolated CABG, and (iii) occurrence of post-operative AF/AFL prior to randomization. Selection of these 3 candidate variables was based on face validity and standards from the published literature as potential factors which could be associated with development of post-operative AF after cardiac surgery. In addition, 3 variables were selected for this analysis since the primary endpoint occurred in 32 patients in the intervention group.

Abbreviations: AF/AFL, atrial fibrillation/flutter; CABG, coronary artery bypass grafting, CHA_2DS_2-VASc, congestive heart failure, hypertension, age ≥75, diabetes, prior stroke or transient ischemic attack, vascular disease, age ≥65, female sex (1 point each except age ≥75 and stroke which are 2 points), ECG, electrocardiogram.

© 2021 Ha ACT et al. *JAMA Network Open.*

eTable 11. Detection of Atrial Fibrillation Within 30 Days of Randomization in Relation to Presence of Post-Operative Atrial Fibrillation Occurring Prior to Randomization

Among the 336 patients randomized in the SEARCH-AF trial, 18 (5.6%) patients had AF occurring after cardiac surgery but prior to randomization into the study. These patients did not have atrial fibrillation or flutter at the time of randomization. Below is a table describing the incidence of AF detected within the first 30 days after randomization in relation to the presence or absence of post-operative AF (lasting for <24 hours) observed prior to randomization.

	**Atrial fibrillation occurring after cardiac surgery and before randomization**	
	**Continuous cardiac rhythm monitoring (n=6)**	**Usual care (n=12)**
**Cumulative AF/AFL lasting ≥6 minutes or AF/AFL documented by a 12-lead ECG within 30 Days of Randomization**	1	1

Abbreviations: AF/AFL, atrial fibrillation/flutter; ECG, electrocardiogram.

© 2021 Ha ACT et al. *JAMA Network Open.*

eTable 12. Detection of Atrial Fibrillation Within the First 30 Days After Randomization, According to Type of Wearable Sensor Employed

As of October 1 2018, all patients who required protocol-mandated continuous cardiac rhythm monitoring were monitored with the CardioSTAT™ device instead of the Medtronic SEEQ® device as the SEEQ® program was ceased by Medtronic. Of the 163 patients who were randomized to receive continuous cardiac rhythm monitoring with a wearable sensor, 153 completed monitoring along with a final report that was available for analysis. Below is a table listing the detection rate of cumulative AF/AFL lasting for ≥6 minutes, according to the type of monitoring device employed.

	**SEEQ® (n=119)**	**CardioSTAT™ (n=34)**
**Number of patients in whom cumulative AF/AFL lasting ≥6 minutes was detected by continuous cardiac rhythm monitoring with a wearable sensor**	26 (21.8%)	4 (11.8%)

Abbreviation: AF/AFL, atrial fibrillation/flutter.

© 2021 Ha ACT et al. *JAMA Network Open.*

eFigure: Primary Outcome According to Pre-Specified Subgroups.

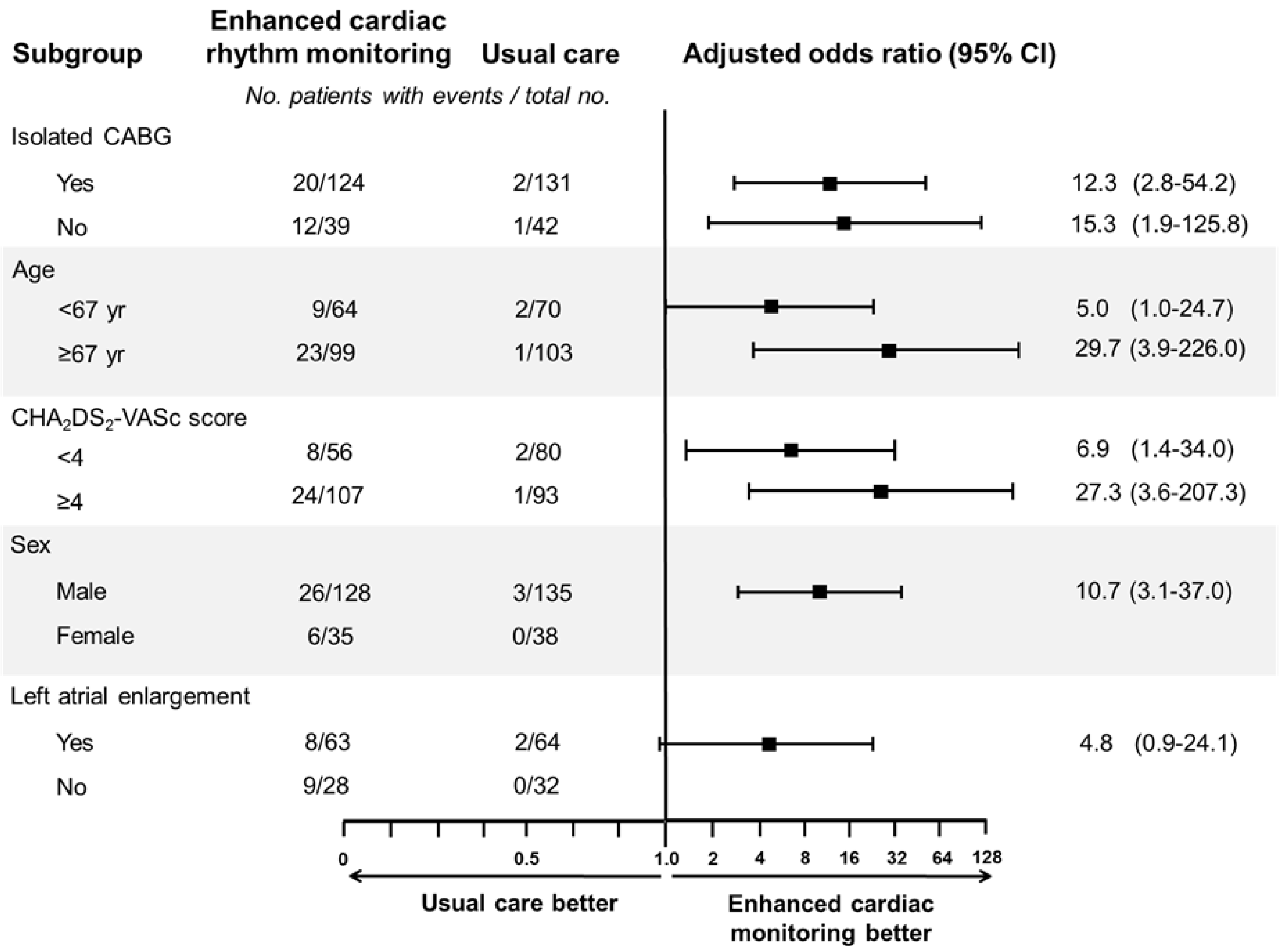

Squares represented point estimates of adjusted odd ratios calculated by logistic regression analysis with lines representing the 95% confidence interval (CI). Of the 336 patients, 187 had an echocardiogram which reported left atrial size. Left atrial enlargement was defined as having one of the following: left atrial size of ≥41 mm on M-mode or ≥59 ml or ≥29 mL/m^2 on biplane volume assessment from an echocardiogram performed within 12 months of study enrollment.

Abbreviations: CABG, coronary artery bypass surgery; CHA_2DS_2-VASc, congestive heart failure, hypertension, age ≥75, diabetes, prior stroke or transient ischemic attack, vascular disease, age ≥65, female sex (1 point each except age >75 and stroke which are 2 points); CI, confidence interval; yr, year.

© 2021 Ha ACT et al. *JAMA Network Open.*
