# Supplementary material for: Effect of Continuous Electrocardiogram Monitoring on Detection of Undiagnosed Atrial Fibrillation After Hospitalization for Cardiac Surgery: A Randomized Clinical Trial
Source: JAMA Netw Open. 2021 Aug 27;4(8):e2121867. doi: 10.1001/jamanetworkopen.2021.21867 (PMC8397929; doi:10.1001/jamanetworkopen.2021.21867)
Supplement: Supplement 3. — SEARCH AF CardioLink-1 Investigators [file jamanetwopen-e2121867-s003.pdf]

Supplemental Online Content: Nonauthor Collaborators

*Indicates required information. Only first name, last name, and suffix will appear in PubMed.

*Group Name(s): SEARCH AF CardioLink-1 Investigators							
***First Name and Middle Initial(s)**	***Last Name**	***Suffix (eg, Jr, III)**	Academic Degrees	Institution	Location (city, state/province, country)	Role or Contribution, eg, chair, principal investigator	Group (if more than 1 Group listed in the byline) and/or Subgroup (eg, Steering Committee)
Andrew C.T.	Ha		MD, MSc	Toronto General Hospital	Toronto, Ontario, Canada	Principal investigator	Steering Committee
Subodh	Verma		MD, PhD	St. Michael's Hospital	Toronto, Ontario, Canada	Co-principal investigator, Study co-chair	Steering Committee
C. David	Mazer		MD	St. Michael's Hospital	Toronto, Ontario, Canada	Co-principal investigator	Steering Committee
Adrian	Quan		MPhil	St. Michael's Hospital	Toronto, Ontario, Canada	Co-investigator, Co-author	Steering Committee
Bobby	Yanagawa		MD, PhD	St. Michael's Hospital	Toronto, Ontario, Canada	Co-principal investigator	Steering Committee
David A.	Latter		MD	St. Michael's Hospital	Toronto, Ontario, Canada	Co-investigator, Co-author	
Terrence M.	Yau		MD, PhD	Toronto General Hospital	Toronto, Ontario, Canada	Co-investigator, Co-author	
Frédéric	Jacques		MD	University Institute of Cardiology and Respirology of Québec	Québec City, Québec, Canada	Co-investigator, Co-author	
Craig D.	Brown		MD	Saint John Regional Hospital	Saint John, New Brunswick, Canada	Co-investigator, Co-author	
Rohit K.	Singal		MD, MSc	St Boniface General Hospital	Winnipeg, Manitoba, Canada	Co-investigator, Co-author	
Michael H.	Yamashita		MD, MPH	St Boniface General Hospital	Winnipeg, Manitoba, Canada	Co-investigator, Co-author	
Tahit	Saha		MD	Kingston General Hospital	Kingston, Ontario, Canada	Co-investigator, Co-author	
Kevin H.	Teoh		MD, MSc	Southlake Regional Health Centre	Newmarket, Ontario, Canada	Co-investigator, Co-author	
Buu-Khanh	Lam		MD, MPH	University of Ottawa Heart Institute	Ottawa, Ontario, Canada	Co-investigator, Co-author	

Supplemental Online Content: Nonauthor Collaborators

*Indicates required information. Only first name, last name, and suffix will appear in PubMed.

*First Name and Middle Initial(s)	*Last Name	*Suffix (eg, Jr, III)	Academic Degrees	Institution	Location (city, state/province, country)	Role or Contribution, eg, chair, principal investigator	Group (if more than 1 Group listed in the byline) and/or Subgroup (eg, Steering Committee)
Marc W.	Deyell		MD, MSc	St. Paul's Hospital	Vancouver, British Columbia, Canada	Co-author	
Marnee	Wilson		RN	St. Michael's Hospital	Toronto, Ontario, Canada	Co-author	
Makoto	Hibino		MD, PhD	St. Michael's Hospital	Toronto, Ontario, Canada	Co-author	
Christopher C.	Cheung		MD	St. Paul's Hospital	Vancouver, British Columbia, Canada	Co-author	
Andrew	Kosmopoulos		BHSc	St. Michael's Hospital	Toronto, Ontario, Canada	Co-author	
Vinay	Garg		MD, MSc	St. Michael's Hospital	Toronto, Ontario, Canada	Co-author	
Shira	Brodutch		MSc	St. Michael's Hospital	Toronto, Ontario, Canada	Co-author	
Hwee	Teoh		PhD	St. Michael's Hospital	Toronto, Ontario, Canada	Co-author	
Fei	Zuo		MPH	Li Ka Shing Knowledge Institute of St Michael's Hospital	Toronto, Ontario, Canada	Co-author	
Kevin E.	Thorpe		MMath	Li Ka Shing Knowledge Institute of St Michael's Hospital	Toronto, Ontario, Canada	Co-author	
Peter	Jüni		MD	Li Ka Shing Knowledge Institute of St Michael's Hospital	Toronto, Ontario, Canada	Co-author	
Deepak L.	Bhatt		MD, MPH	Brigham and Women's Hospital	Boston, Massachusetts, USA	Co-author	Steering Committee
Atul	Verma		MD	Southlake Regional Health Centre	Newmarket, Ontario, Canada	Co-principal investigator, Study co-chair	Steering Committee
