# Supplementary material for: Effect of Continuous Electrocardiogram Monitoring on Detection of Undiagnosed Atrial Fibrillation After Hospitalization for Cardiac Surgery: A Randomized Clinical Trial
Source: JAMA Netw Open. 2021 Aug 27;4(8):e2121867. doi: 10.1001/jamanetworkopen.2021.21867 (PMC8397929; doi:10.1001/jamanetworkopen.2021.21867)
Supplement: Supplement 4. — Data Sharing Statement [file jamanetwopen-e2121867-s004.pdf]

Data Sharing Statement

Ha. Effect of Continuous Electrocardiogram Monitoring on Detection of Undiagnosed Atrial Fibrillation After Hospitalization for Cardiac Surgery. *JAMA Netw Open*. Published August 27, 2021. doi:10.1001/jamanetworkopen.2021.21867

Data
Data available: No

Additional Information
Explanation for why data not available: Data will be available to external investigators by written request and approval of steering committee
